# Mind the Gap: Mapping Mass Spectral Databases in Genome-Scale Metabolic Networks Reveals Poorly Covered Areas

**DOI:** 10.3390/metabo8030051

**Published:** 2018-09-15

**Authors:** Clément Frainay, Emma L. Schymanski, Steffen Neumann, Benjamin Merlet, Reza M. Salek, Fabien Jourdan, Oscar Yanes

**Affiliations:** 1 Toxalim (Research Centre in Food Toxicology), Université de Toulouse, INRA, ENVT, INP-Purpan, UPS, 31555 Toulouse, France; Clement.Frainay@inra.fr (C.F.); Benjamin.Merlet@inra.fr (B.M.); 2 Eawag: Swiss Federal Institute for Aquatic Science and Technology, Überlandstrasse 133, 8600 Dübendorf, Switzerland; emma.schymanski@uni.lu; 3 Luxembourg Centre for Systems Biomedicine (LCSB), University of Luxembourg, 7, avenue des Hauts-Fourneaux, L-4362 Esch-sur-Alzette, Luxembourg; 4 Leibniz Institute of Plant Biochemistry, Department of Stress and Developmental Biology, Weinberg 3, 06120 Halle, Germany; Sneumann@ipb-halle.de; 5 German Centre for Integrative Biodiversity Research (iDiv), Halle-Jena-Leipzig Deutscher Platz 5e, 04103 Leipzig, Germany; 6 The International Agency for Research on Cancer (IARC), 150 Cours Albert Thomas, 69372 Lyon CEDEX 08, France; SalekR@iarc.fr; 7 Metabolomics Platform, IISPV, Department of Electronic Engineering, Universitat Rovira i Virgili, Avinguda Paisos Catalans 26, 43007 Tarragona, Spain; 8 Spanish Biomedical Research Center in Diabetes and Associated Metabolic Disorders (CIBERDEM), Monforte de Lemos 3-5, 28029 Madrid, Spain

**Keywords:** metabolic networks, mass spectral libraries, metabolite annotation, metabolomics data mapping

## Abstract

The use of mass spectrometry-based metabolomics to study human, plant and microbial biochemistry and their interactions with the environment largely depends on the ability to annotate metabolite structures by matching mass spectral features of the measured metabolites to curated spectra of reference standards. While reference databases for metabolomics now provide information for hundreds of thousands of compounds, barely 5% of these known small molecules have experimental data from pure standards. Remarkably, it is still unknown how well existing mass spectral libraries cover the biochemical landscape of prokaryotic and eukaryotic organisms. To address this issue, we have investigated the coverage of 38 genome-scale metabolic networks by public and commercial mass spectral databases, and found that on average only 40% of nodes in metabolic networks could be mapped by mass spectral information from standards. Next, we deciphered computationally which parts of the human metabolic network are poorly covered by mass spectral libraries, revealing gaps in the eicosanoids, vitamins and bile acid metabolism. Finally, our network topology analysis based on the betweenness centrality of metabolites revealed the top 20 most important metabolites that, if added to MS databases, may facilitate human metabolome characterization in the future.

## 1. Introduction

Metabolomics, or the comprehensive characterization and quantification of metabolites, complements upstream biochemical information obtained from genes, transcripts, and proteins, widening the current genomic reconstructions of metabolism and improving our understanding of biological and environmental processes [1]. Metabolomics is thus finding applications that span almost the full width of natural sciences, ranging from human [2,3], plant [4] and microbial biochemistry [5,6,7] to organism-environment interactions [8,9]. Despite the high research interest, identifying and characterizing the structure of metabolites has become a major obstacle for converting raw mass spectrometry (MS) data into biological knowledge. In this regard, open and commercial MS-based databases play an important role in identifying and characterizing the structure of metabolites by matching mass spectral features of the measured metabolites to curated spectra of reference standards [10]. Despite attempts to increase and improve the content of mass spectral databases in recent years, these are still far from containing experimental data of the known compounds. For instance, the widely used METLIN database [11] and the Human Metabolome Database (HMDB version 4.0) [12] now provide links and information for >900,000 and >110,000 compounds, respectively. However, barely 5% of these known small molecules have experimental spectral data from pure standards [13]. Equally important, the biochemical roles and metabolic activity of such small percentage of known and chemically well characterized metabolites is still lacking. Many compounds in mass spectral databases are exogenous drugs or chemical structures that are mainly laboratory based. Hence, it is important to elucidate how many and which compounds in mass spectral databases are involved in metabolic transformations encoded by the genome of prokaryotic and eukaryotic cells. Answering this question is central to investigate and improve the biochemical landscape of metabolomics databases, and assess their usability for reconstructing comprehensive mechanistic scenarios in cell metabolism.

Here, we use genome-based reconstructions of metabolism, also called genome-scale metabolic networks [14,15], to investigate their coverage by existing mass spectral libraries. Genome-scale metabolic networks are manually curated models that best describe our understanding of the metabolic processes occurring in an organism, acting as an indispensable tool to gain biological insight from metabolomic data. Genome-scale metabolic networks enable in-depth mechanistic interpretation through metabolic flux simulation and network analysis.

By analysing the coverage of metabolic networks, we have computationally deciphered which parts of the human metabolic network are poorly covered by mass spectral libraries and have identified metabolite gaps that, if added to MS databases, may enhance human metabolome characterization in the future, and consequently, provide a better understanding of cell metabolism.

## 2. Material and Methods

### 2.1. Chemical Library

Only compounds with measured mass spectra were used. *In silico* predicted MS/MS spectra available in certain public databases [12] were not considered in our study. A merged list of InChIKeys was initially created from public and commercial datasets published by Vinaixa et al*.* 2016 [13]. This list was further updated with new entries and resources [16,17] yielding: 9419 InChIKeys of compounds from the METLIN database [18] provided by Agilent Technologies; 399 InChIKeys from ReSpect [19]; 1171 InChIKeys from the Wiley MS for ID database provided by Herbert Oberacher; 3401 InChIKeys from the GNPS [20]; 11,009 InChIKeys from MassBank [21]; 3480 InChIKeys from mzCloud provided by Robert Mistrik (21 June 2016); 1034 InChIKeys from the HMDB [12] (downloaded on 21 June 2016); and 242,463 InChIKeys from NIST 14 provided by Stephen Stein and Dmitrii Tchekhovskoi. These InChIKey lists (which often contained duplicated entries) were merged for a total of 261,330 non-redundant InChIKey, containing 253,927 non-redundant InChIKey first-block. The InChIKey mapping was performed using the first block of the string, thus not taking into account charge or stereochemistry.

### 2.2. Human Metabolic Network and Graph Construction

**Figure 1:**
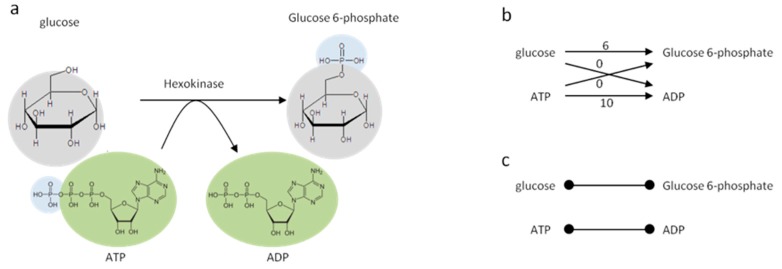
Graph reconstruction process. (**a**) Hexokinase reaction as described in the Recon2 database. Colored circles provide information on shared substructures between substrates and products. (**b**) Compound graph: each substrate is connected to each product of the reaction. Edges are weighted by the number of carbon atoms shared between each substrate to each product. (**c**) Final graph: transitions that do not involve the preservation of at least one carbon atom between the source and the target were removed.

Recon2 [22] was used to map our chemical library of 253,927 non-redundant first block InChIKeys [23]. The original Recon 2 network provided 968 InChIKeys, which was supplemented with additional InChIKeys from other compound identifiers in Recon2, using a combination of web services from PubChem [24], HMDB [25] and ChEBI [26] and home-made parsers (Supplementary File 1). We removed generic compounds (e.g., substrates denoting a set of possible compounds, often by using R-groups, such as an alcohol or sugar) with no proper structure or InChI, and peptides or other macro molecules that are too big to have their structure represented by a single string. We also discarded compounds without any external database reference, as the lack thereof prevents the retrieval of molecular descriptors through the aforementioned web services. Redundancy caused by compounds present in several compartments was avoided by merging all compartments into one single cell-scale model. We created a metabolite network (Compound graph, see Figure 1) where two metabolites are connected if there is at least one reaction producing one and consuming the other, with at least one carbon atom shared between the two metabolites. This allows not taking into account spurious connections involving side compounds like water. Inorganic carbonated compounds, such as CO_2_, were manually removed to complete this task. Some small sub-networks were disconnected from the rest of the network due to missing InChIs or incomplete annotations in Recon2 (network is provided in GML (Graph Modelling Language) format in Supplementary file 2).

### 2.3. Network Topology Analysis

After the creation of the undirected compound graph, we identified parts of the network that were less covered by mass spectral libraries. For this, we used the Label Propagation Algorithm (LPA) [27], which aims at finding communities within a network. The nodes in the network initially carry a label that denotes the two communities they belong to: the “well covered” (mapped metabolites in the chemical library) or the “poorly covered” (unmapped metabolites in the chemical library). The algorithm then diffuses the labels throughout the network by changing membership in both communities based on the labels that the neighbouring nodes (i.e., metabolites) possess. This process is applied to a network where the direction of metabolic reactions is not considered. In a biochemical context, this means that if a mapped metabolite is mostly surrounded by unmapped metabolites, the LPA will switch this metabolite from a ‘well covered’ to a ‘poorly covered’ community. The reasons for it being that measuring such metabolite will likely provide little biochemical information. In contrast, if one unmapped metabolite is mostly surrounded by mapped metabolites, the LPA will switch it from a ‘poorly covered’ to a ‘well covered’ community, so that the absence of this metabolite from mass spectral databases may be counterbalanced by the identification of its neighbouring metabolites. Consequently, densely connected groups of nodes reach a common community label quickly. Such steps were conducted iteratively until all label assignments were stable. We ran the analysis 1000 times and aggregated the results to obtain a final assignment taking into account different ties resolutions scenario (R code is provided in Supplementary File 3).

To identify key missing nodes (i.e., metabolites) in mass spectral libraries, we used a network topology measure called centrality. Centrality is a very well-studied field in network science which aims at identifying important actors in a network. Among the numerous centrality indices, we chose the betweenness as the criterion for metabolite prioritisation. The betweenness centrality quantifies the number of times a node acts as a bridge along the shortest path between two other nodes in the network [28]. The betweenness, therefore, provides a solution to identify metabolites with the greatest potential for bridging the gap between other metabolites, leading to a more cohesive view of the metabolism through metabolomics data.

### 2.4. Publication Mapping

Beside topological measure, we also characterised metabolites through their prominency in scientific literature. We used the PubChem REST API [29] to obtain PubChem identifiers (CID) from our InChIKey list. We then used the API to retrieve PubMed article identifiers (PMID) referenced from an entry accessed through its CID. We compared the number of associated articles between mapped and non-mapped metabolites using Wilcoxon rank sum test with continuity correction and a significance level of α = 0.001. We evaluated the association for a metabolite of having at least one associated article and being mapped using Fisher’s Exact Test, with a significance level of α = 0.001.

## 3. Results

### 3.1. Coverage of Genome-Scale Metabolic Networks by Mass Spectral Libraries

**Figure 2:**
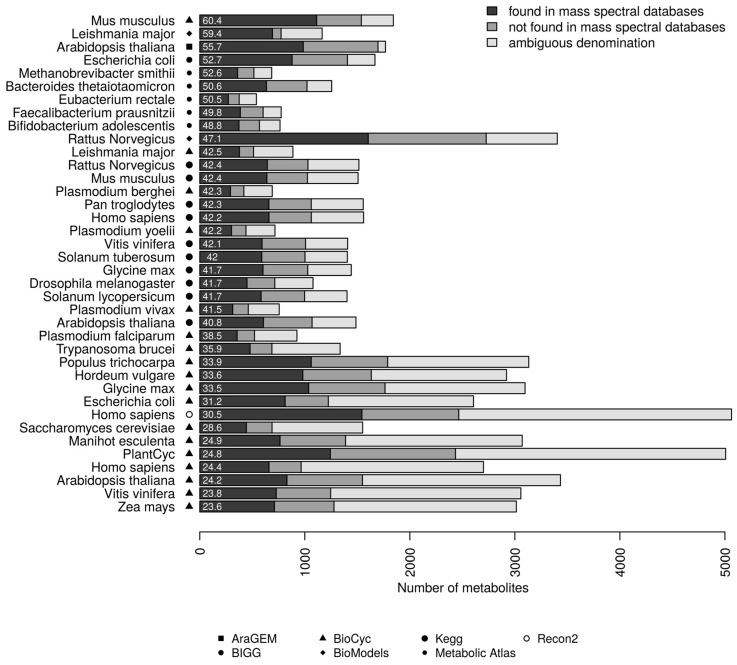
Coverage of prokaryotic and eukaryotic metabolic networks by mass spectral libraries. The genome-scale metabolic models are listed according to an increasing percentage of metabolites covered by mass spectral libraries. The percentage from 60.4 down to 23.6 is displayed to the left of each bar. “Found in mass spectral databases” refers to metabolites that can be mapped in at least one mass spectral database. “Not found in mass spectral databases” refers to compounds with an InChI from metabolic models that could not be matched with any compound in any mass spectral databases. “Ambiguous denomination” refers to compounds with undefined structures or insufficient information to retrieve the unambiguous InChIKey identifier; these compounds were not mapped.

We mapped the chemical library containing 253,927 non-redundant first block InChIKeys onto 38 different genome-scale metabolic networks, including relevant organisms such as *Escherichia coli*, *Arabidopsis thaliana*, *Saccharomyces cerevisiae* (yeast), *Mus musculus* (mouse) or *Homo sapiens* (human). Figure 2 shows the coverage of all the metabolic networks investigated (see Supplementary Files 4 and 5).

Two significant findings can be drawn from a closer analysis of Figure 2. First, the coverage of mapped metabolites in genome-scale metabolic models by mass spectral libraries is relatively low, and coverage varies from 20–60% depending on the species. In the case of model organisms, with extensively characterized genomes and annotated metabolic networks, such as *Mus musculus*, *Escherichia coli* and *Arabidopsis thaliana*, only 52–60% of their metabolomes could be potentially characterized by confronting MS data with all existing mass spectral information from pure standards (which are not currently accessible from a single resource). For human (*Homo sapiens*), this number drops to 42.2% and 30.5% in the case of the KEGG and Recon2 metabolic models, respectively. Second, the annotation level, i.e., specification of chemical identifiers, in genome-scale metabolic models is still very limited. Models such as *Homo sapiens* (Recon2 and HumanCyc) and different plants (PlantCyc) contain a large number of compounds with no compound identifier other than its name, resulting in fewer compounds than expected with associated InChIKey (an unambiguous identifier of chemical substances): 48.7% for Recon2, 48.6% for PlantCyc, and 35.7% for HumanCyc. On average, 63.2% of compounds in our metabolic models have InChIKey, which constitutes an obstacle for reliably mapping experimental metabolomics data onto metabolic models.

**Figure 3:**
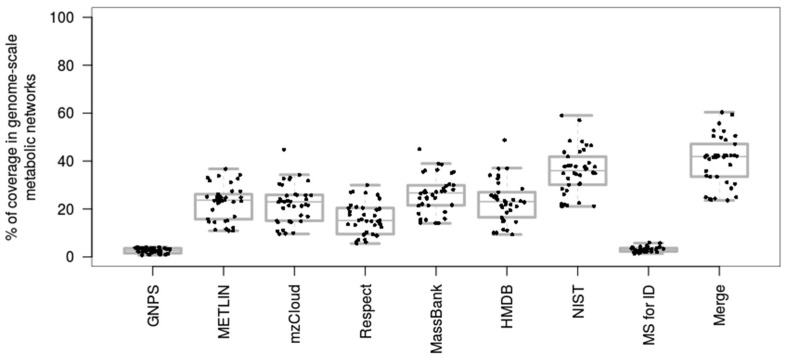
Coverage of prokaryotic and eukaryotic metabolic networks by individual mass spectral databases. HMDB and NIST include MS^2^ and electron ionization (EI)-MS spectral information. Box plots show the distribution of the percentages of coverage in 38 different genome-scale metabolic networks.

Additional to the above analysis, we have also assessed the coverage of individual mass spectral databases in metabolic models (see Supplementary Files 6 and 7). Figure 3 shows, for each spectral library, the percentage of compounds that could be mapped in each network. Overall, databases with the largest number of compounds (by InChIKey), such as NIST and MassBank, showed the best coverage, however these databases also include many exogenous compounds or chemical structures that could not be matched in the genome-scale metabolic models. GNPS covers the smallest percentage of metabolic networks since, at the date of the analysis, the database was mainly focused on secondary metabolites that are not well covered and annotated by genome-scale metabolic networks. The small coverage of MS for ID was also explained by its specificity towards forensic and toxicology related small molecules.

### 3.2. Deciphering Poorly Covered Parts of the Human Metabolic Network

**Figure 4:**
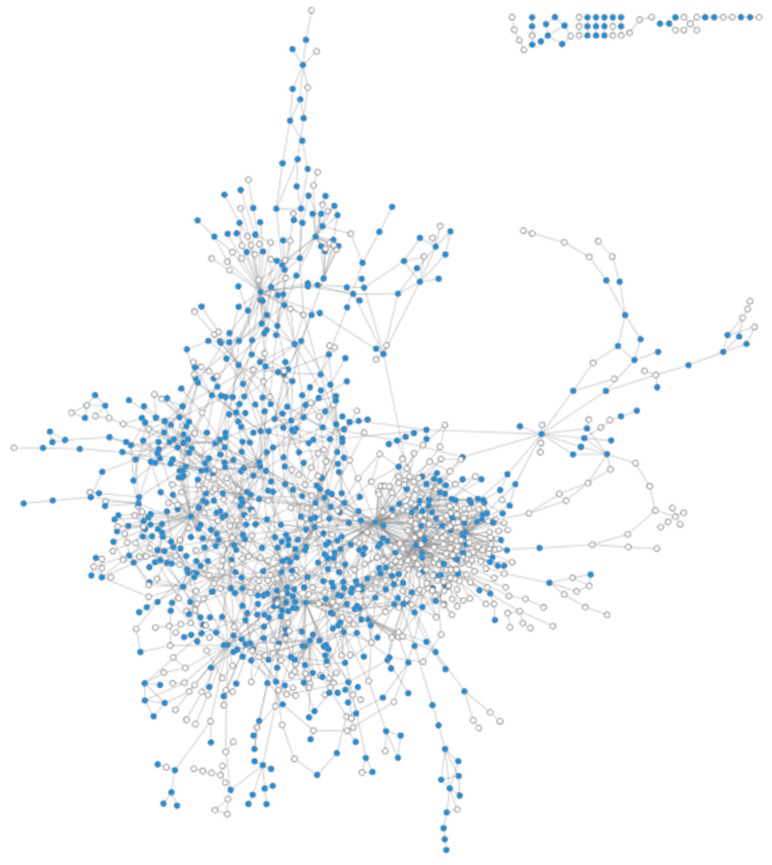
Coverage of the human metabolic network. Blue nodes: Covered by MS databases. White nodes: not covered by MS databases. Isolated nodes have been removed for easy viewing of the metabolic network.

As a priority, coverage of the human metabolic network by existing MS databases was investigated. Figure 4 shows the graph built based on Recon 2.03 human genome-scale metabolic network (see methods section), where the mapped and unmapped metabolites are represented as blue and white nodes, respectively. The number of nodes in the graph has been reduced by eliminating compounds without InChIs, compounds without carbons, and duplicated compounds in different cellular compartments. Inorganic compounds such as CO_2_ were manually removed. Out of 1597 resulting nodes in the metabolic network, 890 metabolites (55.7%) were mapped (see Supplementary File 8).

**Figure 5:**
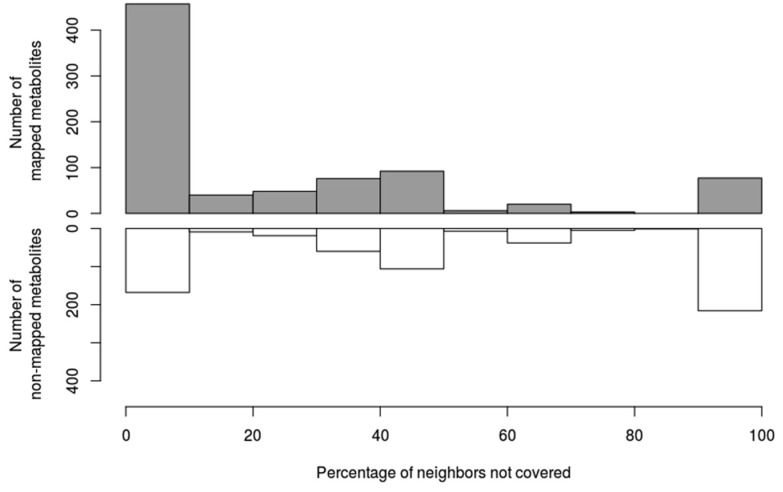
Relative coverage of metabolites’ neighborhood. Metabolites are categorized according to the coverage of their neighborhood, from fully covered to 90–100% uncovered. The *Y*-axis represents the number of metabolites in each category, with mapped metabolites displayed in grey, and non-mapped metabolites displayed in white.

**Figure 6:**
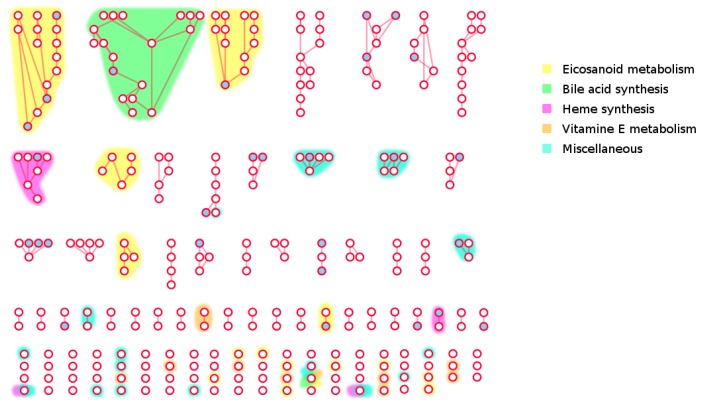
The ‘dark side’ of Human metabolism. The least covered subgraph of Recon 2.03 obtained from LPA using mapping status as the initial state. White circles: Non-mapped metabolites. Blue circles: mapped metabolites. Edges: Substrate-product relationships. Metabolites with ambiguous identifier have been removed. Colored Hulls: Pathways overrepresented in the poorly mapped area of the human metabolic network Recon 2.03. Right-tailed Fisher exact test with Benjamini-Hochberg correction, α = 0.05.

Next, we analysed which parts of the human network are poorly covered by experimental data present in MS databases. To do so, we used the LPA for community detection [27] (see Methods for details) and neighbourhood coverage analysis. The results reveal that 61% of connected metabolites in our network have at least half of their neighbours mapped in MS databases, and 80% have at least one mapped neighbour (Figure 5), which indicates that, despite the low coverage of genome-scale metabolic networks by MS databases, they can still broadly cover the human network without leaving large areas with uncovered metabolites. However, some poorly covered regions were evident in the network. About 293 compounds, of which 216 are not covered, have 90–100% of their neighbours not covered by MS databases either. This may be linked to the existence of metabolic gaps that represents around 18% of the overall network (considering only compounds annotated with InChIKeys). These poorly covered parts of the network identified by LPA are composed of small-size components (Figure 6), supporting the idea that most parts of the known human metabolism are covered in a broad sense. Some metabolic pathways nevertheless appear especially poorly covered, including eicosanoids, vitamins, heme and bile acid metabolism.

**Figure 7:**
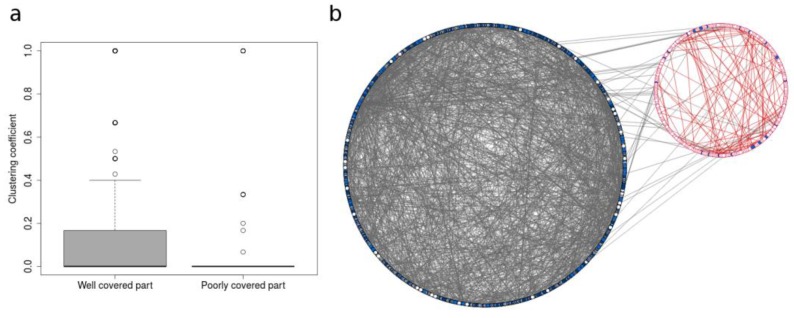
Topological analysis of the least covered areas. (**a**) Clustering coefficient distribution in well covered and poorly covered parts of human metabolism. Only the main component of the whole human metabolic network is considered. (**b**) Well-covered area vs. poorly-covered area in the human metabolic network. Blue nodes: mapped; white nodes: unmapped. Left: Well-covered group; right: poorly covered group. The poorly covered group appears quite small and sparsely connected compared to the well-covered one. Also, there are few connections (i.e., biochemical transformation with some carbon backbone conservation) between the two groups.

**Figure 8:**
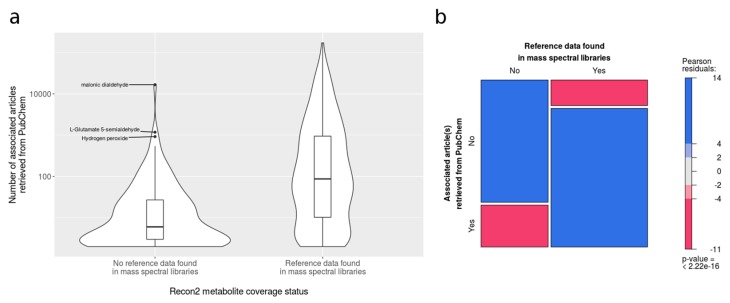
Relationship between the coverage status of Recon2 metabolites and the scientific literature. (**a**) Violin plots showing the distribution of the number of articles associated with mapped and non-mapped metabolites in Recon2. Y axis shows the number of articles (logarithmic scale) obtained from PubMed references in PubChem entries. Only metabolites with at least one associated article are considered. (**b**) Mosaic plot showing the proportion of Recon2 metabolites with PubMed references. Only metabolites with PubChem CID annotation were considered. The area of the tiles is proportional to the number of metabolites within each category. The color and shade of the tiles correspond to the sign and magnitude of the Pearson residuals. The Pearson residuals represent the contribution of the tile to the chi-squared statistics, assessing whether the two variables are independent or not. Red tiles indicate the proportion of under-represented metabolites, namely, metabolites with a smaller number of PubMed references than expected if the two variables (i.e., an entry in spectral libraries and a PubMed article in PubChem) were independent, while blue tiles indicate over-represented metabolites, namely, metabolites with a greater number of PubMed references than expected.

We also explored the topological characteristics of poorly covered parts in the human metabolic network. The most relevant aspect is a lower average clustering coefficient (i.e., nodes often have their neighbours poorly connected, as an indicator of low local density) in the poorly covered areas relative to metabolites from the well-covered areas (Figure 7A). The few links shared between the two parts (Figure 7B) also suggest that the poorly covered areas are virtually disconnected from the rest of the network. Overall, our results indicate that poorly covered areas tend to be located in sparsely connected spaces of the metabolic network. The sparsity of metabolic reactions in the poorly covered areas could describe few and very specific linear pathways, or it may also reveal missing metabolic reactions due to a lack of biochemical knowledge or sporadic activities in scientific investigation in those regions. We have attempted to tackle this issue by analysing the number of publications associated with each metabolite. We have linked metabolites in the networks to publications by retrieving the cross-referenced PubMed articles in their PubChem entry. The non-mapped metabolites (and the sparse regions in the network analysis) tend to have fewer publications than the mapped compounds (Figure 8a). The distribution of publications is heavily skewed, and as a result, we were not able to retrieve any article using PubMed CID query for 588 metabolites, while 53 metabolites exceeded 10,000 articles. The metabolites without associated publications are significantly enriched in non-mapped areas (Figure 8b). Note that 7% of the metabolites were excluded from our query in PubMed because no entry was found for them in PubChem. These missing compounds are also significantly enriched in poorly covered areas of the human network. Overall, this analysis suggests that metabolites not covered by spectral databases are less prominent in the scientific literature.

### 3.3. Filling Gaps in Poorly Covered Areas of Human Metabolism 

Recently, Aguilar-Mogas et al*.*, systematically demonstrated that neighbouring metabolites in a metabolic network share structural similarities and have similar MS/MS spectra [30]. On this basis, our network topology analysis provides an opportunity to identify the most important reference mass spectra to acquire in order to cover the largest number of structurally similar unmapped metabolites in the human metabolic network. Both machine learning algorithms for mass spectra prediction [31,32] and the biochemical interpretation of metabolomics results would benefit from filling these gaps. 

In order to identify the most important metabolites currently missing in the MS databases, we performed a centrality analysis. Table 1 shows the top 20 metabolites with the highest betweenness centrality (see the Methods section) from the poorly mapped areas of human metabolism. These high betweenness metabolites are key chemical structures [33], hence adding their mass spectra to reference libraries, as training data for machine learning algorithms and other identification approaches, will greatly improve prediction of the mass spectra of their unmapped neighbour metabolites. In turn, these metabolites are more likely biochemically affected by the propagation of metabolic perturbations due to their crossroad status, and therefore a must-have in metabolism monitoring.

## 4. Discussion

Here we have combined cheminformatics and network analysis methods to investigate the coverage of public and commercial mass spectral databases in the metabolism of prokaryotic and eukaryotic organisms, particularly taking a closer look at human metabolism. For this, we have used genome-scale metabolic reconstructions, which are considered the most comprehensive and annotated models of metabolism in multiple organisms. Genome-scale metabolic networks contain information both on metabolites and their reactions with corresponding genes and proteins. However, most genome-scale metabolic networks are reconstructed from genomic sequences and literature, and rarely incorporate new and rapidly evolving metabolomic data. This has resulted in some of the constraints and mismatches encountered in our study.

Our computational approach has revealed that many metabolites are missing from mass spectral libraries. For example, 44% of compounds with an InChIKey in Recon2 could not be matched in any mass spectral database. Our results, therefore, provide an essential resource to improve the biochemical landscape of mass spectral databases, and highlights the pressing need for standards to prioritise on to fill these gaps. However, the apparent “low metabolic content” of mass spectral libraries may also be a consequence of insufficient annotation of genome-scale metabolic models. These models (available in SBML format) were initially built for constraint-based computational studies (e.g., Flux Balance Analysis), where the chemical structure of small molecules is not necessarily required for computation. Therefore, most of these models contain a substantial number of metabolites with only short and ambiguous names but no other standard identifiers, which represent a serious obstacle for mapping metabolomics data onto genome-scale metabolic models. Metabolites without proper identifiers can result from the lack of cross-references during their annotation by the scientific community, making computational tools unable to reach the information needed to make correspondences between mass spectral libraries and metabolic networks. One common and useful identifier in this regard is the InChI, which is directly built from the chemical structure of compounds and the hash of the structure, the InChIKey, enabling both the computational analysis performed here, as well as much broader searching of other resources. Unfortunately, we have noticed that most metabolic models often refer to classes of compounds (instead of single chemical species with accurate structures) in order to represent the enzymatic promiscuity of substrates or to describe generic biochemical reactions. Consequently, when the metabolic networks are generated, nodes without chemical structures cannot be mapped on to the mass spectral libraries. Automated approaches to enumerate potentially matching structures to generic representations are required to capture these substances in future studies [34]. Metabolic models may also include some macromolecules that cannot be encoded into all resources due to its string length, although these are likely to be out of the mass range of mass spectrometry in a typical metabolomics experiment. Finally, metabolic models also often contain some entries that do not describe metabolites and therefore cannot be labelled with an InChIKey. For example, most prokaryotic models contain an entry named “biomass”, which provide a convenient way of defining an objective function for constraint-based modelling. The common lack of proper System Biology Ontology (SBO) term annotations and the rare usage of SBML packages allowing different entry types prevent the specific selection of metabolites in models.

The difficulty of mapping metabolomics data onto metabolic networks can also stem from the different scale between models and measures: different stereoisomers may be encoded in the network but are often indistinguishable in a MS experiment (see Figure 1 in Schymanski & Williams 2017 [34]). Furthermore, when no distinction is made between stereoisomers, or between the acid and base form of a compound, one of them can be arbitrarily chosen for setting the name and the annotations of the entry in the model. This could lead to false negatives in the coverage results. To overcome this issue, we used the first InChIKey block, which reduces the structures from the libraries and the networks to a “stereochemistry neutral” or a simplified version of the “MS-ready” form. This can lead to mismatches resulting from tautomers and other substances where different InChIKey first blocks can occur (e.g., monosaccharide compounds in networks, which can be labelled with both the cyclic (PubChem CID:5793) or the linear form (PubChem CID:10954115)). There is thus a strong need to coordinate cheminformaticians with the field of systems biology in order to improve the annotation of metabolic models and develop InChIs and InChIKeys for less defined structures. This would greatly facilitate data exchange and the integration of metabolomics data in the context of metabolic networks.

Eventually, comparing coverages between organisms can be misleading due to differences in size, quality, and completeness of metabolic models. Plant models, for instance, contain the largest number of metabolites among eukaryote organisms, yet they seem to have the poorest coverage by spectral data. While our work focused on human metabolism, the same workflow could be implemented by experts in plant metabolism to reveal metabolite gaps. On the other hand, incomplete and small metabolic models with a relatively good coverage may hide a ‘streetlight effect’, since these models are mainly annotated with well-known reactions and compounds, which are more likely to be present in mass spectral libraries. Since spectral databases and metabolic models are so dynamic, we present the data “as calculated” to describe the first use of LPA to detect dense blind spots in the coverage of a metabolic network. 

Also significant is the striking number of compounds in the spectral databases that did not match with any of our 38 genome-scale networks, namely 251.763 compounds, that is, ~99% in the merged database. Possible causes may include a very large number of exogenous compounds and secondary metabolites in spectral databases, synthetic compounds not found in nature, the annotation in other organisms that were not included in our list of genome-scale networks, and non-enzymatically produced compounds.

Finally, it should be emphasized the continuous growth of mass spectral databases with the addition of new spectra. Since performing this analysis, the latest NIST2017 has been released with spectra from 15,243 compounds, while mzCloud has grown to contain spectra from 7249 compounds (just to name two examples). The methods proposed in this article are sufficiently generic to be applied to updated datasets and/or in-house spectral libraries. It will also be possible to apply this approach to updated versions of metabolic networks. As a matter of fact, a new version of the human metabolic network Recon has been released concurrently to our work [35]. Our preliminary analysis indicates that Recon3D has considerably more annotated compounds with associated InChI than Recon2, however, the coverage of mapped metabolites remains roughly the same. We think, however, that further analyses and improvements of metabolic networks should be considered on the basis of Recon3D.

## Figures and Tables

**Table 1:** Top 20 metabolites with the highest overall betweenness centrality from the poorly mapped area of human network. PubChem CIDs were obtained using the Chemical Translation Service (http://cts.fiehnlab.ucdavis.edu/) with the name as presented in the first column.

Name (from Network)	PubChem CID	InChIKey
(25*R*)-3alpha,7alpha,12alpha-trihydroxy-5beta-cholestan-26-oyl-CoA(4-)	15942889	MNYDLIUNNOCPHG-FJWDCHQMSA-N
12-oxo-c-LTB3	122164853	ZFHPYBQKHVEFHO-LECUDPRGSA-N
3alpha,7alpha,12alpha-Trihydroxy-5beta-cholestanoate	440460	CNWPIIOQKZNXBB-SQZFNYHNSA-N
3alpha,7alpha,12alpha-trihydroxy-5beta-cholestan-26-al	193321	XJZGNVBLVFOSKJ-XZULNKEGSA-N
12-oxo-leukotriene B4	5280876	SJVWVCVZWMJXOK-NOJHDUNKSA-N
20-CoA-20-oxo-leukotriene B4	53481505	WLWKYZHFLKRKEU-WCOJVGLOSA-J
5beta-cholestane-3alpha,7alpha,12alpha,26-tetrol	439479	USFJGINJGUIFSY-XZULNKEGSA-N
(4*R*,5*S*)-4,5,6-trihydroxy-2,3-dioxohexanoate	440390	GJQWCDSAOUMKSE-STHAYSLISA-N
20-carboxy-leukotriene-B4	5280877	SXWGPVJGNOLNHT-VFLUTPEKSA-N
5beta-cholestane-3alpha,7alpha,12alpha-triol	160520	RIVQQZVHIVNQFH-XJZYBRFWSA-N
3-oxo-tetracosa-12,15,18,21-all-cis-tetraenoyl-CoA	131769900	HPMVBGKWFWCZAY-JDTXFHFDSA-N
6-pyruvoyl-5,6,7,8-tetrahydropterin	128973	WBJZXBUVECZHCE-UHFFFAOYSA-N
Hydroxymethylbilane	788	WDFJYRZCZIUBPR-UHFFFAOYSA-N
5beta-cholestane-3alpha,7alpha,12alpha,25-tetrol	160520	RIVQQZVHIVNQFH-XJZYBRFWSA-N
3(*S*)-hydroxy-tetracosa-12,15,18,21-all-cis-tetraenoyl-CoA	53477712	NTIXPPFPXLYJCT-OWOWEXKPSA-N
Uroporphyrinogen III	1179	HUHWZXWWOFSFKF-UHFFFAOYSA-N
12-oxo-20-hydroxy-leukotriene B4	53481459	CZWPUWRHQBAXJS-PABROBRYSA-N
3-oxo-all-cis-6,9,12,15,18-tetracosapentaenoyl-CoA	131769894	UQPANOGFYCZRAV-UWOIJHEUSA-N
all-cis-10,13,16,19-docosatetraenoyl-CoA	71627222	BEEQBBPNTYBGDP-BUSXXEPMSA-J
kinetensin	53481569	PANUJGMSOSQAAY-HAGIGRARSA-N

## Supplementary Materials

The following are available online at http://www.mdpi.com/2218-1989/8/3/51/s1, Supplementary File 1: Supp1-Recon2.v03_noCompartment_extra-annotations.xml. Supplementary File 2: Supp2-Recon2_compound-graph.gml. Supplementary File 3: Supp3-label-propagation-script.r. Supplementary File 4. Supp4-merge_mapping.csv. Supplementary File 5. Supp5-merge_mapping_barplot.r. Supplementary File 6: Supp6-model_vs_lib.csv. Supplementary File 7: Supp7-model_vs_lib_boxplot.r. Supplementary File 8: Supp8-recon_mapping_status.csv. Supplementary File 9: Supp9-recon_CID_and_publication_count.tab.
