# Supplementary material for: Mind the Gap: Mapping Mass Spectral Databases in Genome-Scale Metabolic Networks Reveals Poorly Covered Areas
Source: Metabolites. 2018 Sep 15;8(3):51. doi: 10.3390/metabo8030051 (PMC6161000; doi:10.3390/metabo8030051)
Supplement: Supplementary file 1 [file metabolites-08-00051-s001.zip › SupplementaryMaterialLegends.docx]

**<?xml version='1.0' encoding='UTF-8' standalone='no'?>**

**<sbml xmlns="http://www.sbml.org/sbml/level3/version1/core" level="3" version="1">**

**<model id="NA" name="Recon2v3, enriched with additional database refs, without compartments, without transports">**

**<listOfUnitDefinitions>**

**<unitDefinition id="mmol_per_gDW_per_hr" name="mmol_per_gDW_per_hr">**

**<listOfUnits>**

**<unit scale="0" exponent="-1" multiplier="0.00027777" kind="second"/>**

**<unit scale="-3" exponent="1" multiplier="1" kind="mole"/>**

**<unit scale="1" exponent="-1" multiplier="1" kind="gram"/>**

**</listOfUnits>**

**</unitDefinition>**

**</listOfUnitDefinitions>**

**<listOfCompartments>**

**<compartment id="metaComp" constant="true" spatialDimensions="3" name="metaComp" size="1"/>**

**</listOfCompartments>**

**<listOfSpecies>**

**<species id="M_phpyr" constant="false" hasOnlySubstanceUnits="false" name="keto-phenylpyruvate" metaid="cc4e72cb-7bb1-442c-8d8d-9d20e46f204f" boundaryCondition="false" compartment="metaComp">**

**<notes>**

**<body xmlns="http://www.w3.org/1999/xhtml">**

**<p>FORMULA: C9H7O3</p>**

**<p>CHARGE: 0</p>**

**<p>PUBCHEM.COMPOUND: 997</p>**

**<p>INCHIKEY: BTNMPGBKDVTSJY-UHFFFAOYSA-M</p>**

**<p>KEGG.COMPOUND: C00166</p>**

**<p>HMDB: HMDB00205</p>**

**<p>CHEBI: CHEBI:30851 || CHEBI:18005</p>**

**<p>INCHI: InChI=1S/C9H8O3/c10-8(9(11)12)6-7-4-2-1-3-5-7/h1-5H,6H2,(H,11,12) || InChI=1S/C9H8O3/c10-8(9(11)12)6-7-4-2-1-3-5-7/h1-5H,6H2,(H,11,12)/p-1</p>**

**</body>**

**</notes>**

**<annotation>**

**<rdf:RDF xmlns:rdf="http://www.w3.org/1999/02/22-rdf-syntax-ns#" xmlns:bqmodel="http://biomodels.net/model-qualifiers/" xmlns:bqbiol="http://biomodels.net/biology-qualifiers/">**

**<rdf:Description rdf:about="cc4e72cb-7bb1-442c-8d8d-9d20e46f204f">**

**<bqbiol:is>**

**<rdf:Bag>**

**<rdf:li rdf:resource="http://identifiers.org/PUBCHEM.COMPOUND/997"/></rdf:Bag></bqbiol:is><bqbiol:is>**

**<rdf:Bag>**

**<rdf:li rdf:resource="http://identifiers.org/INCHIKEY/BTNMPGBKDVTSJY-UHFFFAOYSA-M"/></rdf:Bag></bqbiol:is><bqbiol:is>**

**<rdf:Bag>**

**<rdf:li rdf:resource="http://identifiers.org/KEGG.COMPOUND/C00166"/></rdf:Bag></bqbiol:is><bqbiol:is>**

**<rdf:Bag>**

**<rdf:li rdf:resource="http://identifiers.org/HMDB/HMDB00205"/></rdf:Bag></bqbiol:is><bqbiol:is>**

**<rdf:Bag>**

**<rdf:li rdf:resource="http://identifiers.org/CHEBI/CHEBI:30851"/></rdf:Bag></bqbiol:is><bqbiol:is>**

**<rdf:Bag>**

**<rdf:li rdf:resource="http://identifiers.org/CHEBI/CHEBI:18005"/></rdf:Bag></bqbiol:is><bqbiol:is>**

**<rdf:Bag>**

**<rdf:li rdf:resource="http://identifiers.org/INCHI/InChI=1S/C9H8O3/c10-8(9(11)12)6-7-4-2-1-3-5-7/h1-5H,6H2,(H,11,12)"/></rdf:Bag></bqbiol:is><bqbiol:is>**

**<rdf:Bag>**

**<rdf:li rdf:resource="http://identifiers.org/INCHI/InChI=1S/C9H8O3/c10-8(9(11)12)6-7-4-2-1-3-5-7/h1-5H,6H2,(H,11,12)/p-1"/></rdf:Bag></bqbiol:is></rdf:Description></rdf:RDF>**

**</annotation>**

**</species>**

**<species id="M_acgam" constant="false" hasOnlySubstanceUnits="false" name="N-acetyl-D-glucosamine" metaid="3600c8e1-a929-4f3c-b2ac-e212cfc2037f" boundaryCondition="false" compartment="metaComp">**

**<notes>**

**<body xmlns="http://www.w3.org/1999/xhtml">**

**<p>FORMULA: C8H15NO6</p>**

**<p>CHARGE: 0</p>**

**<p>PUBCHEM.COMPOUND: 1201535 || 439174 || 24139 || 445246 || 1738118 || 644170 || 11861101 || 82313 || 343911 || 51403829 || 71464349 || 899 || 440552 || 71306821</p>**

**<p>INCHIKEY: OVRNDRQMDRJTHS-RTRLPJTCSA-N</p>**

**<p>KEGG.COMPOUND: C00140 || C05021 || C20328 || C02711 || C03878</p>**

**<p>HMDB: HMDB00803 || HMDB00215 || HMDB00853</p>**

**<p>CHEBI: CHEBI:506227 || CHEBI:28497 || CHEBI:41112 || CHEBI:44278 || CHEBI:28009 || CHEBI:50622</p>**

**<p>INCHI: InChI=1S/C8H15NO6/c1-3(11)9-5-7(13)6(12)4(2-10)15-8(5)14/h4-8,10,12-14H,2H2,1H3,(H,9,11)/t4-,5-,6-,7-,8?/m1/s1 || InChI=1S/C8H15NO6/c1-4(12)9-5(2-10)7(14)8(15)6(13)3-11/h2,5-8,11,13-15H,3H2,1H3,(H,9,12)/t5-,6+,7+,8+/m0/s1</p>**

**</body>**

**</notes>**

**<annotation>**

**<rdf:RDF xmlns:rdf="http://www.w3.org/1999/02/22-rdf-syntax-ns#" xmlns:bqmodel="http://biomodels.net/model-qualifiers/" xmlns:bqbiol="http://biomodels.net/biology-qualifiers/">**

**<rdf:Description rdf:about="_3600c8e1-a929-4f3c-b2ac-e212cfc2037f">**

**<bqbiol:is>**

**<rdf:Bag>**

**<rdf:li rdf:resource="http://identifiers.org/PUBCHEM.COMPOUND/1201535"/></rdf:Bag></bqbiol:is><bqbiol:is>**

**<rdf:Bag>**

**<rdf:li rdf:resource="http://identifiers.org/PUBCHEM.COMPOUND/439174"/></rdf:Bag></bqbiol:is><bqbiol:is>**

**<rdf:Bag>**

**<rdf:li rdf:resource="http://identifiers.org/PUBCHEM.COMPOUND/24139"/></rdf:Bag></bqbiol:is><bqbiol:is>**

**<rdf:Bag>**

**<rdf:li rdf:resource="http://identifiers.org/PUBCHEM.COMPOUND/445246"/></rdf:Bag></bqbiol:is><bqbiol:is>**

**<rdf:Bag>**

**<rdf:li rdf:resource="http://identifiers.org/PUBCHEM.COMPOUND/1738118"/></rdf:Bag></bqbiol:is><bqbiol:is>**

**<rdf:Bag>**

**<rdf:li rdf:resource="http://identifiers.org/PUBCHEM.COMPOUND/644170"/></rdf:Bag></bqbiol:is><bqbiol:is>**

**<rdf:Bag>**

**<rdf:li rdf:resource="http://identifiers.org/PUBCHEM.COMPOUND/11861101"/></rdf:Bag></bqbiol:is><bqbiol:is>**

**<rdf:Bag>**

**<rdf:li rdf:resource="http://identifiers.org/PUBCHEM.COMPOUND/82313"/></rdf:Bag></bqbiol:is><bqbiol:is>**

**<rdf:Bag>**

**<rdf:li rdf:resource="http://identifiers.org/PUBCHEM.COMPOUND/343911"/></rdf:Bag></bqbiol:is><bqbiol:is>**

**<rdf:Bag>**

**<rdf:li rdf:resource="http://identifiers.org/PUBCHEM.COMPOUND/51403829"/></rdf:Bag></bqbiol:is><bqbiol:is>**

**<rdf:Bag>**

**<rdf:li rdf:resource="http://identifiers.org/PUBCHEM.COMPOUND/71464349"/></rdf:Bag></bqbiol:is><bqbiol:is>**

**<rdf:Bag>**

**<rdf:li rdf:resource="http://identifiers.org/PUBCHEM.COMPOUND/899"/></rdf:Bag></bqbiol:is><bqbiol:is>**

**<rdf:Bag>**

**<rdf:li rdf:resource="http://identifiers.org/PUBCHEM.COMPOUND/440552"/></rdf:Bag></bqbiol:is><bqbiol:is>**

**<rdf:Bag>**

**<rdf:li rdf:resource="http://identifiers.org/PUBCHEM.COMPOUND/71306821"/></rdf:Bag></bqbiol:is><bqbiol:is>**

**<rdf:Bag>**

**<rdf:li rdf:resource="http://identifiers.org/INCHIKEY/OVRNDRQMDRJTHS-RTRLPJTCSA-N"/></rdf:Bag></bqbiol:is><bqbiol:is>**

**<rdf:Bag>**

**<rdf:li rdf:resource="http://identifiers.org/KEGG.COMPOUND/C00140"/></rdf:Bag></bqbiol:is><bqbiol:is>**

**<rdf:Bag>**

**<rdf:li rdf:resource="http://identifiers.org/KEGG.COMPOUND/C05021"/></rdf:Bag></bqbiol:is><bqbiol:is>**

**<rdf:Bag>**

**<rdf:li rdf:resource="http://identifiers.org/KEGG.COMPOUND/C20328"/></rdf:Bag></bqbiol:is><bqbiol:is>**

**<rdf:Bag>**

**<rdf:li rdf:resource="http://identifiers.org/KEGG.COMPOUND/C02711"/></rdf:Bag></bqbiol:is><bqbiol:is>**

**<rdf:Bag>**

**<rdf:li rdf:resource="http://identifiers.org/KEGG.COMPOUND/C03878"/></rdf:Bag></bqbiol:is><bqbiol:is>**

**<rdf:Bag>**

**<rdf:li rdf:resource="http://identifiers.org/HMDB/HMDB00803"/></rdf:Bag></bqbiol:is><bqbiol:is>**

**<rdf:Bag>**

**<rdf:li rdf:resource="http://identifiers.org/HMDB/HMDB00215"/></rdf:Bag></bqbiol:is><bqbiol:is>**

**<rdf:Bag>**

**<rdf:li rdf:resource="http://identifiers.org/HMDB/HMDB00853"/></rdf:Bag></bqbiol:is><bqbiol:is>**

**<rdf:Bag>**

**<rdf:li rdf:resource="http://identifiers.org/CHEBI/CHEBI:506227"/></rdf:Bag></bqbiol:is><bqbiol:is>**

**<rdf:Bag>**

**<rdf:li rdf:resource="http://identifiers.org/CHEBI/CHEBI:28497"/></rdf:Bag></bqbiol:is><bqbiol:is>**

**<rdf:Bag>**

**<rdf:li rdf:resource="http://identifiers.org/CHEBI/CHEBI:41112"/></rdf:Bag></bqbiol:is><bqbiol:is>**

**<rdf:Bag>**

**<rdf:li rdf:resource="http://identifiers.org/CHEBI/CHEBI:44278"/></rdf:Bag></bqbiol:is><bqbiol:is>**

**<rdf:Bag>**

**<rdf:li rdf:resource="http://identifiers.org/CHEBI/CHEBI:28009"/></rdf:Bag></bqbiol:is><bqbiol:is>**

**<rdf:Bag>**

**<rdf:li rdf:resource="http://identifiers.org/CHEBI/CHEBI:50622"/></rdf:Bag></bqbiol:is><bqbiol:is>**

**<rdf:Bag>**

**<rdf:li rdf:resource="http://identifiers.org/INCHI/InChI=1S/C8H15NO6/c1-3(11)9-5-7(13)6(12)4(2-10)15-8(5)14/h4-8,10,12-14H,2H2,1H3,(H,9,11)/t4-,5-,6-,7-,8?/m1/s1"/></rdf:Bag></bqbiol:is><bqbiol:is>**

**<rdf:Bag>**

**<rdf:li rdf:resource="http://identifiers.org/INCHI/InChI=1S/C8H15NO6/c1-4(12)9-5(2-10)7(14)8(15)6(13)3-11/h2,5-8,11,13-15H,3H2,1H3,(H,9,12)/t5-,6+,7+,8+/m0/s1"/></rdf:Bag></bqbiol:is></rdf:Description></rdf:RDF>**

**</annotation>**

**</species>**

**<species id="M_acgal" constant="false" hasOnlySubstanceUnits="false" name="N-acetyl-D-galactosamine" metaid="ac70be1b-9804-4a9e-a935-fd7aaa9644b2" boundaryCondition="false" compartment="metaComp">**

**<notes>**

**<body xmlns="http://www.w3.org/1999/xhtml">**

**<p>FORMULA: C8H15NO6</p>**

**<p>CHARGE: 0</p>**

**<p>PUBCHEM.COMPOUND: 84265 || 448896 || 44119931 || 343911 || 57455154 || 439174 || 35717 || 6971059 || 899 || 440552 || 92164</p>**

**<p>INCHIKEY: OVRNDRQMDRJTHS-KEWYIRBNSA-N</p>**

**<p>KEGG.COMPOUND: C00140 || C01074 || C05021 || C01132 || C02711</p>**

**<p>HMDB: HMDB00212 || HMDB00853</p>**

**<p>CHEBI: CHEBI:28037 || CHEBI:506227 || CHEBI:28497 || CHEBI:40356</p>**

**<p>INCHI: InChI=1S/C8H15NO6/c1-3(11)9-5-7(13)6(12)4(2-10)15-8(5)14/h4-8,10,12-14H,2H2,1H3,(H,9,11)/t4-,5-,6+,7-,8?/m1/s1 || InChI=1S/C8H15NO6/c1-3(11)9-5-7(13)6(12)4(2-10)15-8(5)14/h4-8,10,12-14H,2H2,1H3,(H,9,11)/t4-,5-,6+,7-,8+/m1/s1</p>**

**</body>**

**</notes>**

**<annotation>**

**<rdf:RDF xmlns:rdf="http://www.w3.org/1999/02/22-rdf-syntax-ns#" xmlns:bqmodel="http://biomodels.net/model-qualifiers/" xmlns:bqbiol="http://biomodels.net/biology-qualifiers/">**

**<rdf:Description rdf:about="ac70be1b-9804-4a9e-a935-fd7aaa9644b2">**

**<bqbiol:is>**

**<rdf:Bag>**

**<rdf:li rdf:resource="http://identifiers.org/PUBCHEM.COMPOUND/84265"/></rdf:Bag></bqbiol:is><bqbiol:is>**

**<rdf:Bag>**

**<rdf:li rdf:resource="http://identifiers.org/PUBCHEM.COMPOUND/448896"/></rdf:Bag></bqbiol:is><bqbiol:is>**

**<rdf:Bag>**

**<rdf:li rdf:resource="http://identifiers.org/PUBCHEM.COMPOUND/44119931"/></rdf:Bag></bqbiol:is><bqbiol:is>**

**<rdf:Bag>**

**<rdf:li rdf:resource="http://identifiers.org/PUBCHEM.COMPOUND/343911"/></rdf:Bag></bqbiol:is><bqbiol:is>**

**<rdf:Bag>**

**<rdf:li rdf:resource="http://identifiers.org/PUBCHEM.COMPOUND/57455154"/></rdf:Bag></bqbiol:is><bqbiol:is>**

**<rdf:Bag>**

**<rdf:li rdf:resource="http://identifiers.org/PUBCHEM.COMPOUND/439174"/></rdf:Bag></bqbiol:is><bqbiol:is>**

**<rdf:Bag>**

**<rdf:li rdf:resource="http://identifiers.org/PUBCHEM.COMPOUND/35717"/></rdf:Bag></bqbiol:is><bqbiol:is>**

**<rdf:Bag>**

**<rdf:li rdf:resource="http://identifiers.org/PUBCHEM.COMPOUND/6971059"/></rdf:Bag></bqbiol:is><bqbiol:is>**

**<rdf:Bag>**

**<rdf:li rdf:resource="http://identifiers.org/PUBCHEM.COMPOUND/899"/></rdf:Bag></bqbiol:is><bqbiol:is>**

**<rdf:Bag>**

**<rdf:li rdf:resource="http://identifiers.org/PUBCHEM.COMPOUND/440552"/></rdf:Bag></bqbiol:is><bqbiol:is>**

**<rdf:Bag>**

**<rdf:li rdf:resource="http://identifiers.org/PUBCHEM.COMPOUND/92164"/></rdf:Bag></bqbiol:is><bqbiol:is>**

**<rdf:Bag>**

**<rdf:li rdf:resource="http://identifiers.org/INCHIKEY/OVRNDRQMDRJTHS-KEWYIRBNSA-N"/></rdf:Bag></bqbiol:is><bqbiol:is>**

**<rdf:Bag>**

**<rdf:li rdf:resource="http://identifiers.org/KEGG.COMPOUND/C00140"/></rdf:Bag></bqbiol:is><bqbiol:is>**

**<rdf:Bag>**

**<rdf:li rdf:resource="http://identifiers.org/KEGG.COMPOUND/C01074"/></rdf:Bag></bqbiol:is><bqbiol:is>**

**<rdf:Bag>**

**<rdf:li rdf:resource="http://identifiers.org/KEGG.COMPOUND/C05021"/></rdf:Bag></bqbiol:is><bqbiol:is>**

**<rdf:Bag>**

**<rdf:li rdf:resource="http://identifiers.org/KEGG.COMPOUND/C01132"/></rdf:Bag></bqbiol:is><bqbiol:is>**

**<rdf:Bag>**

**<rdf:li rdf:resource="http://identifiers.org/KEGG.COMPOUND/C02711"/></rdf:Bag></bqbiol:is><bqbiol:is>**

**<rdf:Bag>**

**<rdf:li rdf:resource="http://identifiers.org/HMDB/HMDB00212"/></rdf:Bag></bqbiol:is><bqbiol:is>**

**<rdf:Bag>**

**<rdf:li rdf:resource="http://identifiers.org/HMDB/HMDB00853"/></rdf:Bag></bqbiol:is><bqbiol:is>**

**<rdf:Bag>**

**<rdf:li rdf:resource="http://identifiers.org/CHEBI/CHEBI:28037"/></rdf:Bag></bqbiol:is><bqbiol:is>**

**<rdf:Bag>**

**<rdf:li rdf:resource="http://identifiers.org/CHEBI/CHEBI:506227"/></rdf:Bag></bqbiol:is><bqbiol:is>**

**<rdf:Bag>**

**<rdf:li rdf:resource="http://identifiers.org/CHEBI/CHEBI:28497"/></rdf:Bag></bqbiol:is><bqbiol:is>**

**<rdf:Bag>**

**<rdf:li rdf:resource="http://identifiers.org/CHEBI/CHEBI:40356"/></rdf:Bag></bqbiol:is><bqbiol:is>**

**<rdf:Bag>**

**<rdf:li rdf:resource="http://identifiers.org/INCHI/InChI=1S/C8H15NO6/c1-3(11)9-5-7(13)6(12)4(2-10)15-8(5)14/h4-8,10,12-14H,2H2,1H3,(H,9,11)/t4-,5-,6+,7-,8?/m1/s1"/></rdf:Bag></bqbiol:is><bqbiol:is>**

**<rdf:Bag>**

**<rdf:li rdf:resource="http://identifiers.org/INCHI/InChI=1S/C8H15NO6/c1-3(11)9-5-7(13)6(12)4(2-10)15-8(5)14/h4-8,10,12-14H,2H2,1H3,(H,9,11)/t4-,5-,6+,7-,8+/m1/s1"/></rdf:Bag></bqbiol:is></rdf:Description></rdf:RDF>**

**</annotation>**

**</species>**

**<species id="M_HC00617" constant="false" hasOnlySubstanceUnits="false" name="Ferricytochrome b5" metaid="9599be1c-7e3f-4210-ad8e-139dbdf72e80" boundaryCondition="false" compartment="metaComp">**

**<notes>**

**<body xmlns="http://www.w3.org/1999/xhtml">**

**<p>CHARGE: 0</p>**

**<p>KEGG.COMPOUND: C00996</p>**

**<p>CHEBI: CHEBI:18097</p>**

**</body>**

**</notes>**

**<annotation>**

**<rdf:RDF xmlns:rdf="http://www.w3.org/1999/02/22-rdf-syntax-ns#" xmlns:bqmodel="http://biomodels.net/model-qualifiers/" xmlns:bqbiol="http://biomodels.net/biology-qualifiers/">**

**<rdf:Description rdf:about="_9599be1c-7e3f-4210-ad8e-139dbdf72e80">**

**<bqbiol:is>**

**<rdf:Bag>**

**<rdf:li rdf:resource="http://identifiers.org/KEGG.COMPOUND/C00996"/></rdf:Bag></bqbiol:is><bqbiol:is>**

**<rdf:Bag>**

**<rdf:li rdf:resource="http://identifiers.org/CHEBI/CHEBI:18097"/></rdf:Bag></bqbiol:is></rdf:Description></rdf:RDF>**

**</annotation>**

**</species>**

**<species id="M_limnen" constant="false" hasOnlySubstanceUnits="false" name="limonene" metaid="3c701b84-c6a8-4494-9986-5401321c2ba3" boundaryCondition="false" compartment="metaComp">**

**<notes>**

**<body xmlns="http://www.w3.org/1999/xhtml">**

**<p>FORMULA: C10H16</p>**

**<p>CHARGE: 0</p>**

**<p>PUBCHEM.COMPOUND: 439250 || 22311</p>**

**<p>INCHIKEY: XMGQYMWWDOXHJM-UHFFFAOYSA-N</p>**

**<p>KEGG.COMPOUND: C06078 || C00521</p>**

**<p>HMDB: HMDB03375</p>**

**<p>CHEBI: CHEBI:15384 || CHEBI:15383</p>**

**<p>INCHI: InChI=1S/C10H16/c1-8(2)10-6-4-9(3)5-7-10/h4,10H,1,5-7H2,2-3H3/t10-/m1/s1 || InChI=1S/C10H16/c1-8(2)10-6-4-9(3)5-7-10/h4,10H,1,5-7H2,2-3H3</p>**

**</body>**

**</notes>**

**<annotation>**

**<rdf:RDF xmlns:rdf="http://www.w3.org/1999/02/22-rdf-syntax-ns#" xmlns:bqmodel="http://biomodels.net/model-qualifiers/" xmlns:bqbiol="http://biomodels.net/biology-qualifiers/">**

**<rdf:Description rdf:about="_3c701b84-c6a8-4494-9986-5401321c2ba3">**

**<bqbiol:is>**

**<rdf:Bag>**

**<rdf:li rdf:resource="http://identifiers.org/PUBCHEM.COMPOUND/439250"/></rdf:Bag></bqbiol:is><bqbiol:is>**

**<rdf:Bag>**

**<rdf:li rdf:resource="http://identifiers.org/PUBCHEM.COMPOUND/22311"/></rdf:Bag></bqbiol:is><bqbiol:is>**

**<rdf:Bag>**

**<rdf:li rdf:resource="http://identifiers.org/INCHIKEY/XMGQYMWWDOXHJM-UHFFFAOYSA-N"/></rdf:Bag></bqbiol:is><bqbiol:is>**

**<rdf:Bag>**

**<rdf:li rdf:resource="http://identifiers.org/KEGG.COMPOUND/C06078"/></rdf:Bag></bqbiol:is><bqbiol:is>**

**<rdf:Bag>**

**<rdf:li rdf:resource="http://identifiers.org/KEGG.COMPOUND/C00521"/></rdf:Bag></bqbiol:is><bqbiol:is>**

**<rdf:Bag>**

**<rdf:li rdf:resource="http://identifiers.org/HMDB/HMDB03375"/></rdf:Bag></bqbiol:is><bqbiol:is>**

**<rdf:Bag>**

**<rdf:li rdf:resource="http://identifiers.org/CHEBI/CHEBI:15384"/></rdf:Bag></bqbiol:is><bqbiol:is>**

**<rdf:Bag>**

**<rdf:li rdf:resource="http://identifiers.org/CHEBI/CHEBI:15383"/></rdf:Bag></bqbiol:is><bqbiol:is>**

**<rdf:Bag>**

**<rdf:li rdf:resource="http://identifiers.org/INCHI/InChI=1S/C10H16/c1-8(2)10-6-4-9(3)5-7-10/h4,10H,1,5-7H2,2-3H3/t10-/m1/s1"/></rdf:Bag></bqbiol:is><bqbiol:is>**

**<rdf:Bag>**

**<rdf:li rdf:resource="http://identifiers.org/INCHI/InChI=1S/C10H16/c1-8(2)10-6-4-9(3)5-7-10/h4,10H,1,5-7H2,2-3H3"/></rdf:Bag></bqbiol:is></rdf:Description></rdf:RDF>**

**</annotation>**

**</species>**

**<species id="M_accoa" constant="false" hasOnlySubstanceUnits="false" name="acetyl-CoA(4-)" metaid="05efa58c-637b-4b2e-83aa-629e37325d31" boundaryCondition="false" compartment="metaComp">**

**<notes>**

**<body xmlns="http://www.w3.org/1999/xhtml">**

**<p>FORMULA: C23H34N7O17P3S</p>**

**<p>CHARGE: 0</p>**

**<p>PUBCHEM.COMPOUND: 444493</p>**

**<p>KEGG.COMPOUND: C00024</p>**

**<p>HMDB: HMDB01206</p>**

**<p>CHEBI: CHEBI:15351 || CHEBI:57288</p>**

**<p>INCHI: InChI=1S/C23H38N7O17P3S/c1-12(31)51-7-6-25-14(32)4-5-26-21(35)18(34)23(2,3)9-44-50(41,42)47-49(39,40)43-8-13-17(46-48(36,37)38)16(33)22(45-13)30-11-29-15-19(24)27-10-28-20(15)30/h10-11,13,16-18,22,33-34H,4-9H2,1-3H3,(H,25,32)(H,26,35)(H,39,40)(H,41,42)(H2,24,27,28)(H2,36,37,38)/p-4/t13-,16-,17-,18+,22-/m1/s1 || InChI=1S/C23H38N7O17P3S/c1-12(31)51-7-6-25-14(32)4-5-26-21(35)18(34)23(2,3)9-44-50(41,42)47-49(39,40)43-8-13-17(46-48(36,37)38)16(33)22(45-13)30-11-29-15-19(24)27-10-28-20(15)30/h10-11,13,16-18,22,33-34H,4-9H2,1-3H3,(H,25,32)(H,26,35)(H,39,40)(H,41,42)(H2,24,27,28)(H2,36,37,38)/t13-,16-,17-,18+,22-/m1/s1</p>**

**</body>**

**</notes>**

**<annotation>**

**<rdf:RDF xmlns:rdf="http://www.w3.org/1999/02/22-rdf-syntax-ns#" xmlns:bqmodel="http://biomodels.net/model-qualifiers/" xmlns:bqbiol="http://biomodels.net/biology-qualifiers/">**

**<rdf:Description rdf:about="_05efa58c-637b-4b2e-83aa-629e37325d31">**

**<bqbiol:is>**

**<rdf:Bag>**

**<rdf:li rdf:resource="http://identifiers.org/PUBCHEM.COMPOUND/444493"/></rdf:Bag></bqbiol:is><bqbiol:is>**

**<rdf:Bag>**

**<rdf:li rdf:resource="http://identifiers.org/KEGG.COMPOUND/C00024"/></rdf:Bag></bqbiol:is><bqbiol:is>**

**<rdf:Bag>**

**<rdf:li rdf:resource="http://identifiers.org/HMDB/HMDB01206"/></rdf:Bag></bqbiol:is><bqbiol:is>**

**<rdf:Bag>**

**<rdf:li rdf:resource="http://identifiers.org/CHEBI/CHEBI:15351"/></rdf:Bag></bqbiol:is><bqbiol:is>**

**<rdf:Bag>**

**<rdf:li rdf:resource="http://identifiers.org/CHEBI/CHEBI:57288"/></rdf:Bag></bqbiol:is><bqbiol:is>**

**<rdf:Bag>**

**<rdf:li rdf:resource="http://identifiers.org/INCHI/InChI=1S/C23H38N7O17P3S/c1-12(31)51-7-6-25-14(32)4-5-26-21(35)18(34)23(2,3)9-44-50(41,42)47-49(39,40)43-8-13-17(46-48(36,37)38)16(33)22(45-13)30-11-29-15-19(24)27-10-28-20(15)30/h10-11,13,16-18,22,33-34H,4-9H2,1-3H3,(H,25,32)(H,26,35)(H,39,40)(H,41,42)(H2,24,27,28)(H2,36,37,38)/p-4/t13-,16-,17-,18+,22-/m1/s1"/></rdf:Bag></bqbiol:is><bqbiol:is>**

**<rdf:Bag>**

**<rdf:li rdf:resource="http://identifiers.org/INCHI/InChI=1S/C23H38N7O17P3S/c1-12(31)51-7-6-25-14(32)4-5-26-21(35)18(34)23(2,3)9-44-50(41,42)47-49(39,40)43-8-13-17(46-48(36,37)38)16(33)22(45-13)30-11-29-15-19(24)27-10-28-20(15)30/h10-11,13,16-18,22,33-34H,4-9H2,1-3H3,(H,25,32)(H,26,35)(H,39,40)(H,41,42)(H2,24,27,28)(H2,36,37,38)/t13-,16-,17-,18+,22-/m1/s1"/></rdf:Bag></bqbiol:is></rdf:Description></rdf:RDF>**

**</annotation>**

**</species>**

**<species id="M_gln_L" constant="false" hasOnlySubstanceUnits="false" name="L-glutamine" metaid="3afe26de-b7be-4497-bc27-45bcdef98f58" boundaryCondition="false" compartment="metaComp">**

**<notes>**

**<body xmlns="http://www.w3.org/1999/xhtml">**

**<p>FORMULA: C5H10N2O3</p>**

**<p>CHARGE: 0</p>**

**<p>PUBCHEM.COMPOUND: 738 || 5961 || 25244553 || 6992086</p>**

**<p>INCHIKEY: ZDXPYRJPNDTMRX-VKHMYHEASA-N</p>**

**<p>KEGG.COMPOUND: C00303 || C00064</p>**

**<p>HMDB: HMDB00641</p>**

**<p>CHEBI: CHEBI:18050 || CHEBI:58359 || CHEBI:28300</p>**

**<p>INCHI: InChI=1S/C5H10N2O3/c6-3(5(9)10)1-2-4(7)8/h3H,1-2,6H2,(H2,7,8)(H,9,10)/t3-/m0/s1</p>**

**</body>**

**</notes>**

**<annotation>**

**<rdf:RDF xmlns:rdf="http://www.w3.org/1999/02/22-rdf-syntax-ns#" xmlns:bqmodel="http://biomodels.net/model-qualifiers/" xmlns:bqbiol="http://biomodels.net/biology-qualifiers/">**

**<rdf:Description rdf:about="_3afe26de-b7be-4497-bc27-45bcdef98f58">**

**<bqbiol:is>**

**<rdf:Bag>**

**<rdf:li rdf:resource="http://identifiers.org/PUBCHEM.COMPOUND/738"/></rdf:Bag></bqbiol:is><bqbiol:is>**

**<rdf:Bag>**

**<rdf:li rdf:resource="http://identifiers.org/PUBCHEM.COMPOUND/5961"/></rdf:Bag></bqbiol:is><bqbiol:is>**

**<rdf:Bag>**

**<rdf:li rdf:resource="http://identifiers.org/PUBCHEM.COMPOUND/25244553"/></rdf:Bag></bqbiol:is><bqbiol:is>**

**<rdf:Bag>**

**<rdf:li rdf:resource="http://identifiers.org/PUBCHEM.COMPOUND/6992086"/></rdf:Bag></bqbiol:is><bqbiol:is>**

**<rdf:Bag>**

**<rdf:li rdf:resource="http://identifiers.org/INCHIKEY/ZDXPYRJPNDTMRX-VKHMYHEASA-N"/></rdf:Bag></bqbiol:is><bqbiol:is>**

**<rdf:Bag>**

**<rdf:li rdf:resource="http://identifiers.org/KEGG.COMPOUND/C00303"/></rdf:Bag></bqbiol:is><bqbiol:is>**

**<rdf:Bag>**

**<rdf:li rdf:resource="http://identifiers.org/KEGG.COMPOUND/C00064"/></rdf:Bag></bqbiol:is><bqbiol:is>**

**<rdf:Bag>**

**<rdf:li rdf:resource="http://identifiers.org/HMDB/HMDB00641"/></rdf:Bag></bqbiol:is><bqbiol:is>**

**<rdf:Bag>**

**<rdf:li rdf:resource="http://identifiers.org/CHEBI/CHEBI:18050"/></rdf:Bag></bqbiol:is><bqbiol:is>**

**<rdf:Bag>**

**<rdf:li rdf:resource="http://identifiers.org/CHEBI/CHEBI:58359"/></rdf:Bag></bqbiol:is><bqbiol:is>**

**<rdf:Bag>**

**<rdf:li rdf:resource="http://identifiers.org/CHEBI/CHEBI:28300"/></rdf:Bag></bqbiol:is><bqbiol:is>**

**<rdf:Bag>**

**<rdf:li rdf:resource="http://identifiers.org/INCHI/InChI=1S/C5H10N2O3/c6-3(5(9)10)1-2-4(7)8/h3H,1-2,6H2,(H2,7,8)(H,9,10)/t3-/m0/s1"/></rdf:Bag></bqbiol:is></rdf:Description></rdf:RDF>**

**</annotation>**

**</species>**

**<species id="M_CE5747" constant="false" hasOnlySubstanceUnits="false" name="N-retinylidene-N-retinylethanolamine" metaid="772d61ba-76ec-48bd-86fe-21d6dc006e3d" boundaryCondition="false" compartment="metaComp">**

**<notes>**

**<body xmlns="http://www.w3.org/1999/xhtml">**

**<p>FORMULA: C42H58NO</p>**

**<p>CHARGE: 0</p>**

**<p>INCHI: InChI=1/C42H58NO/c1-32(20-22-39-35(4)17-12-25-41(39,6)7)14-10-16-34(3)30-38-31-37(24-27-43(38)28-29-44)19-11-15-33(2)21-23-40-36(5)18-13-26-42(40,8)9/h10-11,14-16,19-24,27,30-31,44H,12-13,17-18,25-26,28-29H2,1-9H3/q+1/b16-10+,19-11+,22-20+,23-21+,32-14+,33-15+,34-30+</p>**

**</body>**

**</notes>**

**<annotation>**

**<rdf:RDF xmlns:rdf="http://www.w3.org/1999/02/22-rdf-syntax-ns#" xmlns:bqmodel="http://biomodels.net/model-qualifiers/" xmlns:bqbiol="http://biomodels.net/biology-qualifiers/">**

**<rdf:Description rdf:about="_772d61ba-76ec-48bd-86fe-21d6dc006e3d">**

**<bqbiol:is>**

**<rdf:Bag>**

**<rdf:li rdf:resource="http://identifiers.org/INCHI/InChI=1/C42H58NO/c1-32(20-22-39-35(4)17-12-25-41(39,6)7)14-10-16-34(3)30-38-31-37(24-27-43(38)28-29-44)19-11-15-33(2)21-23-40-36(5)18-13-26-42(40,8)9/h10-11,14-16,19-24,27,30-31,44H,12-13,17-18,25-26,28-29H2,1-9H3/q+1/b16-10+,19-11+,22-20+,23-21+,32-14+,33-15+,34-30+"/></rdf:Bag></bqbiol:is></rdf:Description></rdf:RDF>**

**</annotation>**

**</species>**

**<species id="M_dhlpro" constant="false" hasOnlySubstanceUnits="false" name="Dihydrolipolprotein" metaid="d20f395f-aea9-43e0-9a97-566897991957" boundaryCondition="false" compartment="metaComp">**

**<notes>**

**<body xmlns="http://www.w3.org/1999/xhtml">**

**<p>FORMULA: H2S2X</p>**

**<p>CHARGE: 0</p>**

**<p>KEGG.COMPOUND: C02972</p>**

**<p>CHEBI: CHEBI:16194</p>**

**</body>**

**</notes>**

**<annotation>**

**<rdf:RDF xmlns:rdf="http://www.w3.org/1999/02/22-rdf-syntax-ns#" xmlns:bqmodel="http://biomodels.net/model-qualifiers/" xmlns:bqbiol="http://biomodels.net/biology-qualifiers/">**

**<rdf:Description rdf:about="d20f395f-aea9-43e0-9a97-566897991957">**

**<bqbiol:is>**

**<rdf:Bag>**

**<rdf:li rdf:resource="http://identifiers.org/KEGG.COMPOUND/C02972"/></rdf:Bag></bqbiol:is><bqbiol:is>**

**<rdf:Bag>**

**<rdf:li rdf:resource="http://identifiers.org/CHEBI/CHEBI:16194"/></rdf:Bag></bqbiol:is></rdf:Description></rdf:RDF>**

**</annotation>**

**</species>**

**<species id="M_CE5337" constant="false" hasOnlySubstanceUnits="false" name="3-oxo-10(S)-hydroxy-octadeca-6E,8E,12Z-trienoyl-CoA" metaid="5dc5e92a-d7b0-4e3f-914f-4b47c2ca1954" boundaryCondition="false" compartment="metaComp">**

**<notes>**

**<body xmlns="http://www.w3.org/1999/xhtml">**

**<p>FORMULA: C39H58N7O19P3S</p>**

**<p>CHARGE: 0</p>**

**</body>**

**</notes>**

**<annotation>**

**<rdf:RDF xmlns:rdf="http://www.w3.org/1999/02/22-rdf-syntax-ns#" xmlns:bqmodel="http://biomodels.net/model-qualifiers/" xmlns:bqbiol="http://biomodels.net/biology-qualifiers/">**

**<rdf:Description rdf:about="_5dc5e92a-d7b0-4e3f-914f-4b47c2ca1954"/></rdf:RDF>**

**</annotation>**

**</species>**

**<species id="M_uri" constant="false" hasOnlySubstanceUnits="false" name="Uridine" metaid="34eb102a-ea43-4260-971a-e2a4be77d230" boundaryCondition="false" compartment="metaComp">**

**<notes>**

**<body xmlns="http://www.w3.org/1999/xhtml">**

**<p>FORMULA: C9H12N2O6</p>**

**<p>CHARGE: 0</p>**

**<p>PUBCHEM.COMPOUND: 45358305 || 45356795 || 1177 || 6029</p>**

**<p>INCHIKEY: DRTQHJPVMGBUCF-XVFCMESISA-N</p>**

**<p>KEGG.COMPOUND: C00299</p>**

**<p>HMDB: HMDB00296</p>**

**<p>CHEBI: CHEBI:16704</p>**

**<p>INCHI: InChI=1S/C9H12N2O6/c12-3-4-6(14)7(15)8(17-4)11-2-1-5(13)10-9(11)16/h1-2,4,6-8,12,14-15H,3H2,(H,10,13,16)/t4-,6-,7-,8-/m1/s1</p>**

**</body>**

**</notes>**

**<annotation>**

**<rdf:RDF xmlns:rdf="http://www.w3.org/1999/02/22-rdf-syntax-ns#" xmlns:bqmodel="http://biomodels.net/model-qualifiers/" xmlns:bqbiol="http://biomodels.net/biology-qualifiers/">**

**<rdf:Description rdf:about="_34eb102a-ea43-4260-971a-e2a4be77d230">**

**<bqbiol:is>**

**<rdf:Bag>**

**<rdf:li rdf:resource="http://identifiers.org/PUBCHEM.COMPOUND/45358305"/></rdf:Bag></bqbiol:is><bqbiol:is>**

**<rdf:Bag>**

**<rdf:li rdf:resource="http://identifiers.org/PUBCHEM.COMPOUND/45356795"/></rdf:Bag></bqbiol:is><bqbiol:is>**

**<rdf:Bag>**

**<rdf:li rdf:resource="http://identifiers.org/PUBCHEM.COMPOUND/1177"/></rdf:Bag></bqbiol:is><bqbiol:is>**

**<rdf:Bag>**

**<rdf:li rdf:resource="http://identifiers.org/PUBCHEM.COMPOUND/6029"/></rdf:Bag></bqbiol:is><bqbiol:is>**

**<rdf:Bag>**

**<rdf:li rdf:resource="http://identifiers.org/INCHIKEY/DRTQHJPVMGBUCF-XVFCMESISA-N"/></rdf:Bag></bqbiol:is><bqbiol:is>**

**<rdf:Bag>**

**<rdf:li rdf:resource="http://identifiers.org/KEGG.COMPOUND/C00299"/></rdf:Bag></bqbiol:is><bqbiol:is>**

**<rdf:Bag>**

**<rdf:li rdf:resource="http://identifiers.org/HMDB/HMDB00296"/></rdf:Bag></bqbiol:is><bqbiol:is>**

**<rdf:Bag>**

**<rdf:li rdf:resource="http://identifiers.org/CHEBI/CHEBI:16704"/></rdf:Bag></bqbiol:is><bqbiol:is>**

**<rdf:Bag>**

**<rdf:li rdf:resource="http://identifiers.org/INCHI/InChI=1S/C9H12N2O6/c12-3-4-6(14)7(15)8(17-4)11-2-1-5(13)10-9(11)16/h1-2,4,6-8,12,14-15H,3H2,(H,10,13,16)/t4-,6-,7-,8-/m1/s1"/></rdf:Bag></bqbiol:is></rdf:Description></rdf:RDF>**

**</annotation>**

**</species>**

**<species id="M_HC00229" constant="false" hasOnlySubstanceUnits="false" name="Isomaltose" metaid="f2daee15-8694-44b3-9fa7-f2ed384de509" boundaryCondition="false" compartment="metaComp">**

**<notes>**

**<body xmlns="http://www.w3.org/1999/xhtml">**

**<p>FORMULA: C12H22O11</p>**

**<p>CHARGE: 0</p>**

**<p>PUBCHEM.COMPOUND: 872 || 439193 || 46781992 || 10357 || 5460022</p>**

**<p>INCHIKEY: DLRVVLDZNNYCBX-RTPHMHGBSA-N</p>**

**<p>KEGG.COMPOUND: C00252</p>**

**<p>HMDB: HMDB02923</p>**

**<p>CHEBI: CHEBI:28189</p>**

**<p>INCHI: InChI=1S/C12H22O11/c13-1-3-5(14)8(17)10(19)12(23-3)21-2-4-6(15)7(16)9(18)11(20)22-4/h3-20H,1-2H2/t3-,4-,5-,6-,7+,8+,9-,10-,11?,12+/m1/s1 || InChI=1S/C12H22O11/c13-1-4(15)7(17)8(18)5(16)3-22-12-11(21)10(20)9(19)6(2-14)23-12/h1,4-12,14-21H,2-3H2/t4-,5+,6+,7+,8+,9+,10-,11+,12-/m0/s1</p>**

**</body>**

**</notes>**

**<annotation>**

**<rdf:RDF xmlns:rdf="http://www.w3.org/1999/02/22-rdf-syntax-ns#" xmlns:bqmodel="http://biomodels.net/model-qualifiers/" xmlns:bqbiol="http://biomodels.net/biology-qualifiers/">**

**<rdf:Description rdf:about="f2daee15-8694-44b3-9fa7-f2ed384de509">**

**<bqbiol:is>**

**<rdf:Bag>**

**<rdf:li rdf:resource="http://identifiers.org/PUBCHEM.COMPOUND/872"/></rdf:Bag></bqbiol:is><bqbiol:is>**

**<rdf:Bag>**

**<rdf:li rdf:resource="http://identifiers.org/PUBCHEM.COMPOUND/439193"/></rdf:Bag></bqbiol:is><bqbiol:is>**

**<rdf:Bag>**

**<rdf:li rdf:resource="http://identifiers.org/PUBCHEM.COMPOUND/46781992"/></rdf:Bag></bqbiol:is><bqbiol:is>**

**<rdf:Bag>**

**<rdf:li rdf:resource="http://identifiers.org/PUBCHEM.COMPOUND/10357"/></rdf:Bag></bqbiol:is><bqbiol:is>**

**<rdf:Bag>**

**<rdf:li rdf:resource="http://identifiers.org/PUBCHEM.COMPOUND/5460022"/></rdf:Bag></bqbiol:is><bqbiol:is>**

**<rdf:Bag>**

**<rdf:li rdf:resource="http://identifiers.org/INCHIKEY/DLRVVLDZNNYCBX-RTPHMHGBSA-N"/></rdf:Bag></bqbiol:is><bqbiol:is>**

**<rdf:Bag>**

**<rdf:li rdf:resource="http://identifiers.org/KEGG.COMPOUND/C00252"/></rdf:Bag></bqbiol:is><bqbiol:is>**

**<rdf:Bag>**

**<rdf:li rdf:resource="http://identifiers.org/HMDB/HMDB02923"/></rdf:Bag></bqbiol:is><bqbiol:is>**

**<rdf:Bag>**

**<rdf:li rdf:resource="http://identifiers.org/CHEBI/CHEBI:28189"/></rdf:Bag></bqbiol:is><bqbiol:is>**

**<rdf:Bag>**

**<rdf:li rdf:resource="http://identifiers.org/INCHI/InChI=1S/C12H22O11/c13-1-3-5(14)8(17)10(19)12(23-3)21-2-4-6(15)7(16)9(18)11(20)22-4/h3-20H,1-2H2/t3-,4-,5-,6-,7+,8+,9-,10-,11?,12+/m1/s1"/></rdf:Bag></bqbiol:is><bqbiol:is>**

**<rdf:Bag>**

**<rdf:li rdf:resource="http://identifiers.org/INCHI/InChI=1S/C12H22O11/c13-1-4(15)7(17)8(18)5(16)3-22-12-11(21)10(20)9(19)6(2-14)23-12/h1,4-12,14-21H,2-3H2/t4-,5+,6+,7+,8+,9+,10-,11+,12-/m0/s1"/></rdf:Bag></bqbiol:is></rdf:Description></rdf:RDF>**

**</annotation>**

**</species>**

**<species id="M_CE5331" constant="false" hasOnlySubstanceUnits="false" name="3,5-dioxo-12(S)-hydroxy-eicosa-8E,10E,14Z-trienoyl-CoA" metaid="54289f8b-4fb1-4884-97c2-a6c216b9722b" boundaryCondition="false" compartment="metaComp">**

**<notes>**

**<body xmlns="http://www.w3.org/1999/xhtml">**

**<p>FORMULA: C41H60N7O20P3S</p>**

**<p>CHARGE: 0</p>**

**</body>**

**</notes>**

**<annotation>**

**<rdf:RDF xmlns:rdf="http://www.w3.org/1999/02/22-rdf-syntax-ns#" xmlns:bqmodel="http://biomodels.net/model-qualifiers/" xmlns:bqbiol="http://biomodels.net/biology-qualifiers/">**

**<rdf:Description rdf:about="_54289f8b-4fb1-4884-97c2-a6c216b9722b"/></rdf:RDF>**

**</annotation>**

**</species>**

**<species id="M_HC00619" constant="false" hasOnlySubstanceUnits="false" name="Ferrocytochrome b5" metaid="13f76d03-46f1-4a4e-a0cb-cf687353de10" boundaryCondition="false" compartment="metaComp">**

**<notes>**

**<body xmlns="http://www.w3.org/1999/xhtml">**

**<p>CHARGE: 0</p>**

**<p>PUBCHEM.COMPOUND: 4245</p>**

**<p>KEGG.COMPOUND: C00999</p>**

**<p>CHEBI: CHEBI:16518</p>**

**</body>**

**</notes>**

**<annotation>**

**<rdf:RDF xmlns:rdf="http://www.w3.org/1999/02/22-rdf-syntax-ns#" xmlns:bqmodel="http://biomodels.net/model-qualifiers/" xmlns:bqbiol="http://biomodels.net/biology-qualifiers/">**

**<rdf:Description rdf:about="_13f76d03-46f1-4a4e-a0cb-cf687353de10">**

**<bqbiol:is>**

**<rdf:Bag>**

**<rdf:li rdf:resource="http://identifiers.org/PUBCHEM.COMPOUND/4245"/></rdf:Bag></bqbiol:is><bqbiol:is>**

**<rdf:Bag>**

**<rdf:li rdf:resource="http://identifiers.org/KEGG.COMPOUND/C00999"/></rdf:Bag></bqbiol:is><bqbiol:is>**

**<rdf:Bag>**

**<rdf:li rdf:resource="http://identifiers.org/CHEBI/CHEBI:16518"/></rdf:Bag></bqbiol:is></rdf:Description></rdf:RDF>**

**</annotation>**

**</species>**

**<species id="M_ura" constant="false" hasOnlySubstanceUnits="false" name="Uracil" metaid="1d93a96b-0fce-4ef5-beca-bbe6b8a25ea6" boundaryCondition="false" compartment="metaComp">**

**<notes>**

**<body xmlns="http://www.w3.org/1999/xhtml">**

**<p>FORMULA: C4H4N2O2</p>**

**<p>CHARGE: 0</p>**

**<p>PUBCHEM.COMPOUND: 11083870 || 10171240 || 10975456 || 11251987 || 1174 || 6451479 || 16217569 || 12212752 || 25178219 || 58931967</p>**

**<p>INCHIKEY: ISAKRJDGNUQOIC-UHFFFAOYSA-N</p>**

**<p>HMDB: HMDB00300</p>**

**<p>KEGG.COMPOUND: C00106</p>**

**<p>CHEBI: CHEBI:17568</p>**

**<p>INCHI: InChI=1S/C4H4N2O2/c7-3-1-2-5-4(8)6-3/h1-2H,(H2,5,6,7,8)</p>**

**</body>**

**</notes>**

**<annotation>**

**<rdf:RDF xmlns:rdf="http://www.w3.org/1999/02/22-rdf-syntax-ns#" xmlns:bqmodel="http://biomodels.net/model-qualifiers/" xmlns:bqbiol="http://biomodels.net/biology-qualifiers/">**

**<rdf:Description rdf:about="_1d93a96b-0fce-4ef5-beca-bbe6b8a25ea6">**

**<bqbiol:is>**

**<rdf:Bag>**

**<rdf:li rdf:resource="http://identifiers.org/PUBCHEM.COMPOUND/11083870"/></rdf:Bag></bqbiol:is><bqbiol:is>**

**<rdf:Bag>**

**<rdf:li rdf:resource="http://identifiers.org/PUBCHEM.COMPOUND/10171240"/></rdf:Bag></bqbiol:is><bqbiol:is>**

**<rdf:Bag>**

**<rdf:li rdf:resource="http://identifiers.org/PUBCHEM.COMPOUND/10975456"/></rdf:Bag></bqbiol:is><bqbiol:is>**

**<rdf:Bag>**

**<rdf:li rdf:resource="http://identifiers.org/PUBCHEM.COMPOUND/11251987"/></rdf:Bag></bqbiol:is><bqbiol:is>**

**<rdf:Bag>**

**<rdf:li rdf:resource="http://identifiers.org/PUBCHEM.COMPOUND/1174"/></rdf:Bag></bqbiol:is><bqbiol:is>**

**<rdf:Bag>**

**<rdf:li rdf:resource="http://identifiers.org/PUBCHEM.COMPOUND/6451479"/></rdf:Bag></bqbiol:is><bqbiol:is>**

**<rdf:Bag>**

**<rdf:li rdf:resource="http://identifiers.org/PUBCHEM.COMPOUND/16217569"/></rdf:Bag></bqbiol:is><bqbiol:is>**

**<rdf:Bag>**

**<rdf:li rdf:resource="http://identifiers.org/PUBCHEM.COMPOUND/12212752"/></rdf:Bag></bqbiol:is><bqbiol:is>**

**<rdf:Bag>**

**<rdf:li rdf:resource="http://identifiers.org/PUBCHEM.COMPOUND/25178219"/></rdf:Bag></bqbiol:is><bqbiol:is>**

**<rdf:Bag>**

**<rdf:li rdf:resource="http://identifiers.org/PUBCHEM.COMPOUND/58931967"/></rdf:Bag></bqbiol:is><bqbiol:is>**

**<rdf:Bag>**

**<rdf:li rdf:resource="http://identifiers.org/INCHIKEY/ISAKRJDGNUQOIC-UHFFFAOYSA-N"/></rdf:Bag></bqbiol:is><bqbiol:is>**

**<rdf:Bag>**

**<rdf:li rdf:resource="http://identifiers.org/HMDB/HMDB00300"/></rdf:Bag></bqbiol:is><bqbiol:is>**

**<rdf:Bag>**

**<rdf:li rdf:resource="http://identifiers.org/KEGG.COMPOUND/C00106"/></rdf:Bag></bqbiol:is><bqbiol:is>**

**<rdf:Bag>**

**<rdf:li rdf:resource="http://identifiers.org/CHEBI/CHEBI:17568"/></rdf:Bag></bqbiol:is><bqbiol:is>**

**<rdf:Bag>**

**<rdf:li rdf:resource="http://identifiers.org/INCHI/InChI=1S/C4H4N2O2/c7-3-1-2-5-4(8)6-3/h1-2H,(H2,5,6,7,8)"/></rdf:Bag></bqbiol:is></rdf:Description></rdf:RDF>**

**</annotation>**

**</species>**

**<species id="M_etha" constant="false" hasOnlySubstanceUnits="false" name="etha[c]" metaid="3938a879-c51c-4051-b033-b077f0cb7e46" boundaryCondition="false" compartment="metaComp">**

**<notes>**

**<body xmlns="http://www.w3.org/1999/xhtml">**

**<p>FORMULA: C2H8NO</p>**

**<p>CHARGE: 0</p>**

**<p>PUBCHEM.COMPOUND: 700</p>**

**<p>KEGG.COMPOUND: C00189</p>**

**<p>HMDB: HMDB00149</p>**

**<p>CHEBI: CHEBI:57603 || CHEBI:16000</p>**

**<p>INCHI: InChI=1S/C2H7NO/c3-1-2-4/h4H,1-3H2 || InChI=1S/C2H7NO/c3-1-2-4/h4H,1-3H2/p+1</p>**

**</body>**

**</notes>**

**<annotation>**

**<rdf:RDF xmlns:rdf="http://www.w3.org/1999/02/22-rdf-syntax-ns#" xmlns:bqmodel="http://biomodels.net/model-qualifiers/" xmlns:bqbiol="http://biomodels.net/biology-qualifiers/">**

**<rdf:Description rdf:about="_3938a879-c51c-4051-b033-b077f0cb7e46">**

**<bqbiol:is>**

**<rdf:Bag>**

**<rdf:li rdf:resource="http://identifiers.org/PUBCHEM.COMPOUND/700"/></rdf:Bag></bqbiol:is><bqbiol:is>**

**<rdf:Bag>**

**<rdf:li rdf:resource="http://identifiers.org/KEGG.COMPOUND/C00189"/></rdf:Bag></bqbiol:is><bqbiol:is>**

**<rdf:Bag>**

**<rdf:li rdf:resource="http://identifiers.org/HMDB/HMDB00149"/></rdf:Bag></bqbiol:is><bqbiol:is>**

**<rdf:Bag>**

**<rdf:li rdf:resource="http://identifiers.org/CHEBI/CHEBI:57603"/></rdf:Bag></bqbiol:is><bqbiol:is>**

**<rdf:Bag>**

**<rdf:li rdf:resource="http://identifiers.org/CHEBI/CHEBI:16000"/></rdf:Bag></bqbiol:is><bqbiol:is>**

**<rdf:Bag>**

**<rdf:li rdf:resource="http://identifiers.org/INCHI/InChI=1S/C2H7NO/c3-1-2-4/h4H,1-3H2"/></rdf:Bag></bqbiol:is><bqbiol:is>**

**<rdf:Bag>**

**<rdf:li rdf:resource="http://identifiers.org/INCHI/InChI=1S/C2H7NO/c3-1-2-4/h4H,1-3H2/p+1"/></rdf:Bag></bqbiol:is></rdf:Description></rdf:RDF>**

**</annotation>**

**</species>**

**<species id="M_prist" constant="false" hasOnlySubstanceUnits="false" name="pristanic acid" metaid="0f8fa6d9-439a-4aef-b127-78f8a5a87626" boundaryCondition="false" compartment="metaComp">**

**<notes>**

**<body xmlns="http://www.w3.org/1999/xhtml">**

**<p>FORMULA: C19H37O2</p>**

**<p>CHARGE: 0</p>**

**<p>PUBCHEM.COMPOUND: 123929</p>**

**<p>INCHIKEY: PAHGJZDQXIOYTH-UHFFFAOYSA-N</p>**

**<p>HMDB: HMDB00795</p>**

**<p>CHEBI: CHEBI:51340</p>**

**<p>INCHI: InChI=1S/C19H38O2/c1-15(2)9-6-10-16(3)11-7-12-17(4)13-8-14-18(5)19(20)21/h15-18H,6-14H2,1-5H3,(H,20,21)</p>**

**</body>**

**</notes>**

**<annotation>**

**<rdf:RDF xmlns:rdf="http://www.w3.org/1999/02/22-rdf-syntax-ns#" xmlns:bqmodel="http://biomodels.net/model-qualifiers/" xmlns:bqbiol="http://biomodels.net/biology-qualifiers/">**

**<rdf:Description rdf:about="_0f8fa6d9-439a-4aef-b127-78f8a5a87626">**

**<bqbiol:is>**

**<rdf:Bag>**

**<rdf:li rdf:resource="http://identifiers.org/PUBCHEM.COMPOUND/123929"/></rdf:Bag></bqbiol:is><bqbiol:is>**

**<rdf:Bag>**

**<rdf:li rdf:resource="http://identifiers.org/INCHIKEY/PAHGJZDQXIOYTH-UHFFFAOYSA-N"/></rdf:Bag></bqbiol:is><bqbiol:is>**

**<rdf:Bag>**

**<rdf:li rdf:resource="http://identifiers.org/HMDB/HMDB00795"/></rdf:Bag></bqbiol:is><bqbiol:is>**

**<rdf:Bag>**

**<rdf:li rdf:resource="http://identifiers.org/CHEBI/CHEBI:51340"/></rdf:Bag></bqbiol:is><bqbiol:is>**

**<rdf:Bag>**

**<rdf:li rdf:resource="http://identifiers.org/INCHI/InChI=1S/C19H38O2/c1-15(2)9-6-10-16(3)11-7-12-17(4)13-8-14-18(5)19(20)21/h15-18H,6-14H2,1-5H3,(H,20,21)"/></rdf:Bag></bqbiol:is></rdf:Description></rdf:RDF>**

**</annotation>**

**</species>**

**<species id="M_doldp_L" constant="false" hasOnlySubstanceUnits="false" name="Dolichol diphosphate, human liver homolog" metaid="5b9c3d8a-86ab-4c29-8da5-0e17259375af" boundaryCondition="false" compartment="metaComp">**

**<notes>**

**<body xmlns="http://www.w3.org/1999/xhtml">**

**<p>FORMULA: C1080H1758O70P20</p>**

**<p>CHARGE: 0</p>**

**<p>KEGG.COMPOUND: C00621</p>**

**<p>CHEBI: CHEBI:15750</p>**

**</body>**

**</notes>**

**<annotation>**

**<rdf:RDF xmlns:rdf="http://www.w3.org/1999/02/22-rdf-syntax-ns#" xmlns:bqmodel="http://biomodels.net/model-qualifiers/" xmlns:bqbiol="http://biomodels.net/biology-qualifiers/">**

**<rdf:Description rdf:about="_5b9c3d8a-86ab-4c29-8da5-0e17259375af">**

**<bqbiol:is>**

**<rdf:Bag>**

**<rdf:li rdf:resource="http://identifiers.org/KEGG.COMPOUND/C00621"/></rdf:Bag></bqbiol:is><bqbiol:is>**

**<rdf:Bag>**

**<rdf:li rdf:resource="http://identifiers.org/CHEBI/CHEBI:15750"/></rdf:Bag></bqbiol:is></rdf:Description></rdf:RDF>**

**</annotation>**

**</species>**

**<species id="M_CE5730" constant="false" hasOnlySubstanceUnits="false" name="Prostaglandin PGB2 glyceryl ester" metaid="3dd50e8d-56a3-497f-ae14-a5a6145a4daf" boundaryCondition="false" compartment="metaComp">**

**<notes>**

**<body xmlns="http://www.w3.org/1999/xhtml">**

**<p>FORMULA: C23H36O6</p>**

**<p>CHARGE: 0</p>**

**</body>**

**</notes>**

**<annotation>**

**<rdf:RDF xmlns:rdf="http://www.w3.org/1999/02/22-rdf-syntax-ns#" xmlns:bqmodel="http://biomodels.net/model-qualifiers/" xmlns:bqbiol="http://biomodels.net/biology-qualifiers/">**

**<rdf:Description rdf:about="_3dd50e8d-56a3-497f-ae14-a5a6145a4daf"/></rdf:RDF>**

**</annotation>**

**</species>**

**<species id="M_CE5341" constant="false" hasOnlySubstanceUnits="false" name="5-oxo-12(S)-hydroxy-eicosa-2E,8E,10E,14Z-tetraenoyl-CoA" metaid="7fd6667b-e369-4f73-8ebb-1c5b90a50d3c" boundaryCondition="false" compartment="metaComp">**

**<notes>**

**<body xmlns="http://www.w3.org/1999/xhtml">**

**<p>FORMULA: C41H60N7O19P3S</p>**

**<p>CHARGE: 0</p>**

**</body>**

**</notes>**

**<annotation>**

**<rdf:RDF xmlns:rdf="http://www.w3.org/1999/02/22-rdf-syntax-ns#" xmlns:bqmodel="http://biomodels.net/model-qualifiers/" xmlns:bqbiol="http://biomodels.net/biology-qualifiers/">**

**<rdf:Description rdf:about="_7fd6667b-e369-4f73-8ebb-1c5b90a50d3c"/></rdf:RDF>**

**</annotation>**

**</species>**

**<species id="M_eicostetcoa" constant="false" hasOnlySubstanceUnits="false" name="eicosatetranoyl coenzyme A" metaid="e4e5e023-1331-4f95-915e-75ec75f3e4ad" boundaryCondition="false" compartment="metaComp">**

**<notes>**

**<body xmlns="http://www.w3.org/1999/xhtml">**

**<p>FORMULA: C41H62N7O17P3S</p>**

**<p>CHARGE: 0</p>**

**<p>PUBCHEM.COMPOUND: 23724641</p>**

**<p>KEGG.COMPOUND: C16164</p>**

**<p>INCHI: InChI=1/C41H66N7O17P3S/c1-4-5-6-7-8-9-10-11-12-13-14-15-16-17-18-19-20-21-32(50)69-25-24-43-31(49)22-23-44-39(53)36(52)41(2,3)27-62-68(59,60)65-67(57,58)61-26-30-35(64-66(54,55)56)34(51)40(63-30)48-29-47-33-37(42)45-28-46-38(33)48/h5-6,8-9,11-12,14-15,28-30,34-36,40,51-52H,4,7,10,13,16-27H2,1-3H3,(H,43,49)(H,44,53)(H,57,58)(H,59,60)(H2,42,45,46)(H2,54,55,56)/b6-5-,9-8-,12-11-,15-14-/t30-,34-,35-,36?,40-/m1/s1</p>**

**</body>**

**</notes>**

**<annotation>**

**<rdf:RDF xmlns:rdf="http://www.w3.org/1999/02/22-rdf-syntax-ns#" xmlns:bqmodel="http://biomodels.net/model-qualifiers/" xmlns:bqbiol="http://biomodels.net/biology-qualifiers/">**

**<rdf:Description rdf:about="e4e5e023-1331-4f95-915e-75ec75f3e4ad">**

**<bqbiol:is>**

**<rdf:Bag>**

**<rdf:li rdf:resource="http://identifiers.org/PUBCHEM.COMPOUND/23724641"/></rdf:Bag></bqbiol:is><bqbiol:is>**

**<rdf:Bag>**

**<rdf:li rdf:resource="http://identifiers.org/KEGG.COMPOUND/C16164"/></rdf:Bag></bqbiol:is><bqbiol:is>**

**<rdf:Bag>**

**<rdf:li rdf:resource="http://identifiers.org/INCHI/InChI=1/C41H66N7O17P3S/c1-4-5-6-7-8-9-10-11-12-13-14-15-16-17-18-19-20-21-32(50)69-25-24-43-31(49)22-23-44-39(53)36(52)41(2,3)27-62-68(59,60)65-67(57,58)61-26-30-35(64-66(54,55)56)34(51)40(63-30)48-29-47-33-37(42)45-28-46-38(33)48/h5-6,8-9,11-12,14-15,28-30,34-36,40,51-52H,4,7,10,13,16-27H2,1-3H3,(H,43,49)(H,44,53)(H,57,58)(H,59,60)(H2,42,45,46)(H2,54,55,56)/b6-5-,9-8-,12-11-,15-14-/t30-,34-,35-,36?,40-/m1/s1"/></rdf:Bag></bqbiol:is></rdf:Description></rdf:RDF>**

**</annotation>**

**</species>**

**<species id="M_5hxkyn" constant="false" hasOnlySubstanceUnits="false" name="5-hydroxykynurenine" metaid="9d7aff2e-2408-4fb0-bd84-ef7bce3ce2cd" boundaryCondition="false" compartment="metaComp">**

**<notes>**

**<body xmlns="http://www.w3.org/1999/xhtml">**

**<p>FORMULA: C10H12N2O4</p>**

**<p>CHARGE: 0</p>**

**<p>PUBCHEM.COMPOUND: 440745 || 9548587 || 53262328</p>**

**<p>INCHIKEY: OTDQYOVYQQZAJL-QMMMGPOBSA-N</p>**

**<p>KEGG.COMPOUND: C05651</p>**

**<p>HMDB: HMDB12819</p>**

**<p>CHEBI: CHEBI:2076 || CHEBI:62624 || CHEBI:36408</p>**

**<p>INCHI: InChI=1S/C10H12N2O4/c11-7-2-1-5(13)3-6(7)9(14)4-8(12)10(15)16/h1-3,8,13H,4,11-12H2,(H,15,16) || InChI=1S/C10H12N2O4/c11-7-2-1-5(13)3-6(7)9(14)4-8(12)10(15)16/h1-3,8,13H,4,11-12H2,(H,15,16)/t8-/m0/s1</p>**

**</body>**

**</notes>**

**<annotation>**

**<rdf:RDF xmlns:rdf="http://www.w3.org/1999/02/22-rdf-syntax-ns#" xmlns:bqmodel="http://biomodels.net/model-qualifiers/" xmlns:bqbiol="http://biomodels.net/biology-qualifiers/">**

**<rdf:Description rdf:about="_9d7aff2e-2408-4fb0-bd84-ef7bce3ce2cd">**

**<bqbiol:is>**

**<rdf:Bag>**

**<rdf:li rdf:resource="http://identifiers.org/PUBCHEM.COMPOUND/440745"/></rdf:Bag></bqbiol:is><bqbiol:is>**

**<rdf:Bag>**

**<rdf:li rdf:resource="http://identifiers.org/PUBCHEM.COMPOUND/9548587"/></rdf:Bag></bqbiol:is><bqbiol:is>**

**<rdf:Bag>**

**<rdf:li rdf:resource="http://identifiers.org/PUBCHEM.COMPOUND/53262328"/></rdf:Bag></bqbiol:is><bqbiol:is>**

**<rdf:Bag>**

**<rdf:li rdf:resource="http://identifiers.org/INCHIKEY/OTDQYOVYQQZAJL-QMMMGPOBSA-N"/></rdf:Bag></bqbiol:is><bqbiol:is>**

**<rdf:Bag>**

**<rdf:li rdf:resource="http://identifiers.org/KEGG.COMPOUND/C05651"/></rdf:Bag></bqbiol:is><bqbiol:is>**

**<rdf:Bag>**

**<rdf:li rdf:resource="http://identifiers.org/HMDB/HMDB12819"/></rdf:Bag></bqbiol:is><bqbiol:is>**

**<rdf:Bag>**

**<rdf:li rdf:resource="http://identifiers.org/CHEBI/CHEBI:2076"/></rdf:Bag></bqbiol:is><bqbiol:is>**

**<rdf:Bag>**

**<rdf:li rdf:resource="http://identifiers.org/CHEBI/CHEBI:62624"/></rdf:Bag></bqbiol:is><bqbiol:is>**

**<rdf:Bag>**

**<rdf:li rdf:resource="http://identifiers.org/CHEBI/CHEBI:36408"/></rdf:Bag></bqbiol:is><bqbiol:is>**

**<rdf:Bag>**

**<rdf:li rdf:resource="http://identifiers.org/INCHI/InChI=1S/C10H12N2O4/c11-7-2-1-5(13)3-6(7)9(14)4-8(12)10(15)16/h1-3,8,13H,4,11-12H2,(H,15,16)"/></rdf:Bag></bqbiol:is><bqbiol:is>**

**<rdf:Bag>**

**<rdf:li rdf:resource="http://identifiers.org/INCHI/InChI=1S/C10H12N2O4/c11-7-2-1-5(13)3-6(7)9(14)4-8(12)10(15)16/h1-3,8,13H,4,11-12H2,(H,15,16)/t8-/m0/s1"/></rdf:Bag></bqbiol:is></rdf:Description></rdf:RDF>**

**</annotation>**

**</species>**

**<species id="M_CE5347" constant="false" hasOnlySubstanceUnits="false" name="5-oxo-12(R)-hydroxy-eicosa-2E,8E,10E,14Z-tetraenoyl-CoA" metaid="1fe2925c-0cd5-4cfe-b240-3f90d49e9990" boundaryCondition="false" compartment="metaComp">**

**<notes>**

**<body xmlns="http://www.w3.org/1999/xhtml">**

**<p>FORMULA: C41H60N7O19P3S</p>**

**<p>CHARGE: 0</p>**

**</body>**

**</notes>**

**<annotation>**

**<rdf:RDF xmlns:rdf="http://www.w3.org/1999/02/22-rdf-syntax-ns#" xmlns:bqmodel="http://biomodels.net/model-qualifiers/" xmlns:bqbiol="http://biomodels.net/biology-qualifiers/">**

**<rdf:Description rdf:about="_1fe2925c-0cd5-4cfe-b240-3f90d49e9990"/></rdf:RDF>**

**</annotation>**

**</species>**

**<species id="M_CE5346" constant="false" hasOnlySubstanceUnits="false" name="3-oxo-10(R)-hydroxy-octadeca-6E,8E,12Z-trienoyl-CoA" metaid="9e41eea9-d22b-4dca-8c9d-4c6afe7c5bc5" boundaryCondition="false" compartment="metaComp">**

**<notes>**

**<body xmlns="http://www.w3.org/1999/xhtml">**

**<p>FORMULA: C39H58N7O19P3S</p>**

**<p>CHARGE: 0</p>**

**</body>**

**</notes>**

**<annotation>**

**<rdf:RDF xmlns:rdf="http://www.w3.org/1999/02/22-rdf-syntax-ns#" xmlns:bqmodel="http://biomodels.net/model-qualifiers/" xmlns:bqbiol="http://biomodels.net/biology-qualifiers/">**

**<rdf:Description rdf:about="_9e41eea9-d22b-4dca-8c9d-4c6afe7c5bc5"/></rdf:RDF>**

**</annotation>**

**</species>**

**<species id="M_CE5349" constant="false" hasOnlySubstanceUnits="false" name="5-oxo-6E-12-epi-leukotriene B4" metaid="d57be19c-d07b-42b9-8283-a8815fa59b55" boundaryCondition="false" compartment="metaComp">**

**<notes>**

**<body xmlns="http://www.w3.org/1999/xhtml">**

**<p>FORMULA: C20H29O4</p>**

**<p>CHARGE: 0</p>**

**</body>**

**</notes>**

**<annotation>**

**<rdf:RDF xmlns:rdf="http://www.w3.org/1999/02/22-rdf-syntax-ns#" xmlns:bqmodel="http://biomodels.net/model-qualifiers/" xmlns:bqbiol="http://biomodels.net/biology-qualifiers/">**

**<rdf:Description rdf:about="d57be19c-d07b-42b9-8283-a8815fa59b55"/></rdf:RDF>**

**</annotation>**

**</species>**

**<species id="M_C10164" constant="false" hasOnlySubstanceUnits="false" name="Picolinic acid" metaid="9ea7e353-fa55-45df-b3b0-49869c579d3c" boundaryCondition="false" compartment="metaComp">**

**<notes>**

**<body xmlns="http://www.w3.org/1999/xhtml">**

**<p>FORMULA: C6H4NO2</p>**

**<p>CHARGE: 0</p>**

**<p>PUBCHEM.COMPOUND: 60206411 || 1018 || 57433474</p>**

**<p>INCHIKEY: SIOXPEMLGUPBBT-UHFFFAOYSA-N</p>**

**<p>KEGG.COMPOUND: C10164</p>**

**<p>HMDB: HMDB02243</p>**

**<p>CHEBI: CHEBI:38184 || CHEBI:28747</p>**

**<p>INCHI: InChI=1S/C6H5NO2/c8-6(9)5-3-1-2-4-7-5/h1-4H,(H,8,9)</p>**

**</body>**

**</notes>**

**<annotation>**

**<rdf:RDF xmlns:rdf="http://www.w3.org/1999/02/22-rdf-syntax-ns#" xmlns:bqmodel="http://biomodels.net/model-qualifiers/" xmlns:bqbiol="http://biomodels.net/biology-qualifiers/">**

**<rdf:Description rdf:about="_9ea7e353-fa55-45df-b3b0-49869c579d3c">**

**<bqbiol:is>**

**<rdf:Bag>**

**<rdf:li rdf:resource="http://identifiers.org/PUBCHEM.COMPOUND/60206411"/></rdf:Bag></bqbiol:is><bqbiol:is>**

**<rdf:Bag>**

**<rdf:li rdf:resource="http://identifiers.org/PUBCHEM.COMPOUND/1018"/></rdf:Bag></bqbiol:is><bqbiol:is>**

**<rdf:Bag>**

**<rdf:li rdf:resource="http://identifiers.org/PUBCHEM.COMPOUND/57433474"/></rdf:Bag></bqbiol:is><bqbiol:is>**

**<rdf:Bag>**

**<rdf:li rdf:resource="http://identifiers.org/INCHIKEY/SIOXPEMLGUPBBT-UHFFFAOYSA-N"/></rdf:Bag></bqbiol:is><bqbiol:is>**

**<rdf:Bag>**

**<rdf:li rdf:resource="http://identifiers.org/KEGG.COMPOUND/C10164"/></rdf:Bag></bqbiol:is><bqbiol:is>**

**<rdf:Bag>**

**<rdf:li rdf:resource="http://identifiers.org/HMDB/HMDB02243"/></rdf:Bag></bqbiol:is><bqbiol:is>**

**<rdf:Bag>**

**<rdf:li rdf:resource="http://identifiers.org/CHEBI/CHEBI:38184"/></rdf:Bag></bqbiol:is><bqbiol:is>**

**<rdf:Bag>**

**<rdf:li rdf:resource="http://identifiers.org/CHEBI/CHEBI:28747"/></rdf:Bag></bqbiol:is><bqbiol:is>**

**<rdf:Bag>**

**<rdf:li rdf:resource="http://identifiers.org/INCHI/InChI=1S/C6H5NO2/c8-6(9)5-3-1-2-4-7-5/h1-4H,(H,8,9)"/></rdf:Bag></bqbiol:is></rdf:Description></rdf:RDF>**

**</annotation>**

**</species>**

**<species id="M_strdnc" constant="false" hasOnlySubstanceUnits="false" name="stearidonic acid C18:4, n-3" metaid="43e9fbf5-2887-4b47-903f-c05dd6d2d03c" boundaryCondition="false" compartment="metaComp">**

**<notes>**

**<body xmlns="http://www.w3.org/1999/xhtml">**

**<p>FORMULA: C18H27O2</p>**

**<p>CHARGE: 0</p>**

**<p>PUBCHEM.COMPOUND: 47205608 || 163841 || 5312508</p>**

**<p>KEGG.COMPOUND: C16300</p>**

**<p>HMDB: HMDB06547</p>**

**<p>CHEBI: CHEBI:32389</p>**

**<p>INCHI: InChI=1/C18H28O2/c1-2-3-4-5-6-7-8-9-10-11-12-13-14-15-16-17-18(19)20/h3-4,6-7,9-10,12-13H,2,5,8,11,14-17H2,1H3,(H,19,20)/b4-3-,7-6-,10-9-,13-12- || InChI=1S/C18H28O2/c1-2-3-4-5-6-7-8-9-10-11-12-13-14-15-16-17-18(19)20/h3-4,6-7,9-10,12-13H,2,5,8,11,14-17H2,1H3,(H,19,20)/b4-3-,7-6-,10-9-,13-12-</p>**

**</body>**

**</notes>**

**<annotation>**

**<rdf:RDF xmlns:rdf="http://www.w3.org/1999/02/22-rdf-syntax-ns#" xmlns:bqmodel="http://biomodels.net/model-qualifiers/" xmlns:bqbiol="http://biomodels.net/biology-qualifiers/">**

**<rdf:Description rdf:about="_43e9fbf5-2887-4b47-903f-c05dd6d2d03c">**

**<bqbiol:is>**

**<rdf:Bag>**

**<rdf:li rdf:resource="http://identifiers.org/PUBCHEM.COMPOUND/47205608"/></rdf:Bag></bqbiol:is><bqbiol:is>**

**<rdf:Bag>**

**<rdf:li rdf:resource="http://identifiers.org/PUBCHEM.COMPOUND/163841"/></rdf:Bag></bqbiol:is><bqbiol:is>**

**<rdf:Bag>**

**<rdf:li rdf:resource="http://identifiers.org/PUBCHEM.COMPOUND/5312508"/></rdf:Bag></bqbiol:is><bqbiol:is>**

**<rdf:Bag>**

**<rdf:li rdf:resource="http://identifiers.org/KEGG.COMPOUND/C16300"/></rdf:Bag></bqbiol:is><bqbiol:is>**

**<rdf:Bag>**

**<rdf:li rdf:resource="http://identifiers.org/HMDB/HMDB06547"/></rdf:Bag></bqbiol:is><bqbiol:is>**

**<rdf:Bag>**

**<rdf:li rdf:resource="http://identifiers.org/CHEBI/CHEBI:32389"/></rdf:Bag></bqbiol:is><bqbiol:is>**

**<rdf:Bag>**

**<rdf:li rdf:resource="http://identifiers.org/INCHI/InChI=1/C18H28O2/c1-2-3-4-5-6-7-8-9-10-11-12-13-14-15-16-17-18(19)20/h3-4,6-7,9-10,12-13H,2,5,8,11,14-17H2,1H3,(H,19,20)/b4-3-,7-6-,10-9-,13-12-"/></rdf:Bag></bqbiol:is><bqbiol:is>**

**<rdf:Bag>**

**<rdf:li rdf:resource="http://identifiers.org/INCHI/InChI=1S/C18H28O2/c1-2-3-4-5-6-7-8-9-10-11-12-13-14-15-16-17-18(19)20/h3-4,6-7,9-10,12-13H,2,5,8,11,14-17H2,1H3,(H,19,20)/b4-3-,7-6-,10-9-,13-12-"/></rdf:Bag></bqbiol:is></rdf:Description></rdf:RDF>**

**</annotation>**

**</species>**

**<species id="M_CE5348" constant="false" hasOnlySubstanceUnits="false" name="5-oxo-12(R)-hydroxy-eicosa-8E,10E,14Z-trienoyl-CoA" metaid="5f4cff57-5c21-4516-86d8-7f18f5e834f0" boundaryCondition="false" compartment="metaComp">**

**<notes>**

**<body xmlns="http://www.w3.org/1999/xhtml">**

**<p>FORMULA: C41H62N7O19P3S</p>**

**<p>CHARGE: 0</p>**

**</body>**

**</notes>**

**<annotation>**

**<rdf:RDF xmlns:rdf="http://www.w3.org/1999/02/22-rdf-syntax-ns#" xmlns:bqmodel="http://biomodels.net/model-qualifiers/" xmlns:bqbiol="http://biomodels.net/biology-qualifiers/">**

**<rdf:Description rdf:about="_5f4cff57-5c21-4516-86d8-7f18f5e834f0"/></rdf:RDF>**

**</annotation>**

**</species>**

**<species id="M_CE5343" constant="false" hasOnlySubstanceUnits="false" name="6,7-dihydro-5-oxo-12-epi-leukotriene B" metaid="79205fa5-4cd6-41fa-b26a-ed74ae172d50" boundaryCondition="false" compartment="metaComp">**

**<notes>**

**<body xmlns="http://www.w3.org/1999/xhtml">**

**<p>FORMULA: C20H31O4</p>**

**<p>CHARGE: 0</p>**

**</body>**

**</notes>**

**<annotation>**

**<rdf:RDF xmlns:rdf="http://www.w3.org/1999/02/22-rdf-syntax-ns#" xmlns:bqmodel="http://biomodels.net/model-qualifiers/" xmlns:bqbiol="http://biomodels.net/biology-qualifiers/">**

**<rdf:Description rdf:about="_79205fa5-4cd6-41fa-b26a-ed74ae172d50"/></rdf:RDF>**

**</annotation>**

**</species>**

**<species id="M_CE5342" constant="false" hasOnlySubstanceUnits="false" name="5-oxo-12(S)-hydroxy-eicosa-8E,10E,14Z-trienoyl-CoA" metaid="4e317f96-ed02-4485-886c-6a51d17fb22a" boundaryCondition="false" compartment="metaComp">**

**<notes>**

**<body xmlns="http://www.w3.org/1999/xhtml">**

**<p>FORMULA: C41H62N7O19P3S</p>**

**<p>CHARGE: 0</p>**

**</body>**

**</notes>**

**<annotation>**

**<rdf:RDF xmlns:rdf="http://www.w3.org/1999/02/22-rdf-syntax-ns#" xmlns:bqmodel="http://biomodels.net/model-qualifiers/" xmlns:bqbiol="http://biomodels.net/biology-qualifiers/">**

**<rdf:Description rdf:about="_4e317f96-ed02-4485-886c-6a51d17fb22a"/></rdf:RDF>**

**</annotation>**

**</species>**

**<species id="M_CE5345" constant="false" hasOnlySubstanceUnits="false" name="3,5-dioxo-12(R)-hydroxy-eicosa-8E,10E,14Z-trienoyl-CoA" metaid="69b90865-df41-4c71-b479-cf5cdf90629b" boundaryCondition="false" compartment="metaComp">**

**<notes>**

**<body xmlns="http://www.w3.org/1999/xhtml">**

**<p>FORMULA: C41H60N7O20P3S</p>**

**<p>CHARGE: 0</p>**

**</body>**

**</notes>**

**<annotation>**

**<rdf:RDF xmlns:rdf="http://www.w3.org/1999/02/22-rdf-syntax-ns#" xmlns:bqmodel="http://biomodels.net/model-qualifiers/" xmlns:bqbiol="http://biomodels.net/biology-qualifiers/">**

**<rdf:Description rdf:about="_69b90865-df41-4c71-b479-cf5cdf90629b"/></rdf:RDF>**

**</annotation>**

**</species>**

**<species id="M_amp" constant="false" hasOnlySubstanceUnits="false" name="AMP" metaid="4bff9f1b-0abe-45f8-a696-028facb92c34" boundaryCondition="false" compartment="metaComp">**

**<notes>**

**<body xmlns="http://www.w3.org/1999/xhtml">**

**<p>FORMULA: C10H12N5O7P</p>**

**<p>CHARGE: 0</p>**

**<p>PUBCHEM.COMPOUND: 11987739 || 6083</p>**

**<p>INCHIKEY: UDMBCSSLTHHNCD-KQYNXXCUSA-N</p>**

**<p>KEGG.COMPOUND: C00020</p>**

**<p>HMDB: HMDB00045</p>**

**<p>CHEBI: CHEBI:16027 || CHEBI:456215</p>**

**<p>INCHI: InChI=1S/C10H14N5O7P/c11-8-5-9(13-2-12-8)15(3-14-5)10-7(17)6(16)4(22-10)1-21-23(18,19)20/h2-4,6-7,10,16-17H,1H2,(H2,11,12,13)(H2,18,19,20)/t4-,6-,7-,10-/m1/s1 || InChI=1S/C10H14N5O7P/c11-8-5-9(13-2-12-8)15(3-14-5)10-7(17)6(16)4(22-10)1-21-23(18,19)20/h2-4,6-7,10,16-17H,1H2,(H2,11,12,13)(H2,18,19,20)/p-2/t4-,6-,7-,10-/m1/s1</p>**

**</body>**

**</notes>**

**<annotation>**

**<rdf:RDF xmlns:rdf="http://www.w3.org/1999/02/22-rdf-syntax-ns#" xmlns:bqmodel="http://biomodels.net/model-qualifiers/" xmlns:bqbiol="http://biomodels.net/biology-qualifiers/">**

**<rdf:Description rdf:about="_4bff9f1b-0abe-45f8-a696-028facb92c34">**

**<bqbiol:is>**

**<rdf:Bag>**

**<rdf:li rdf:resource="http://identifiers.org/PUBCHEM.COMPOUND/11987739"/></rdf:Bag></bqbiol:is><bqbiol:is>**

**<rdf:Bag>**

**<rdf:li rdf:resource="http://identifiers.org/PUBCHEM.COMPOUND/6083"/></rdf:Bag></bqbiol:is><bqbiol:is>**

**<rdf:Bag>**

**<rdf:li rdf:resource="http://identifiers.org/INCHIKEY/UDMBCSSLTHHNCD-KQYNXXCUSA-N"/></rdf:Bag></bqbiol:is><bqbiol:is>**

**<rdf:Bag>**

**<rdf:li rdf:resource="http://identifiers.org/KEGG.COMPOUND/C00020"/></rdf:Bag></bqbiol:is><bqbiol:is>**

**<rdf:Bag>**

**<rdf:li rdf:resource="http://identifiers.org/HMDB/HMDB00045"/></rdf:Bag></bqbiol:is><bqbiol:is>**

**<rdf:Bag>**

**<rdf:li rdf:resource="http://identifiers.org/CHEBI/CHEBI:16027"/></rdf:Bag></bqbiol:is><bqbiol:is>**

**<rdf:Bag>**

**<rdf:li rdf:resource="http://identifiers.org/CHEBI/CHEBI:456215"/></rdf:Bag></bqbiol:is><bqbiol:is>**

**<rdf:Bag>**

**<rdf:li rdf:resource="http://identifiers.org/INCHI/InChI=1S/C10H14N5O7P/c11-8-5-9(13-2-12-8)15(3-14-5)10-7(17)6(16)4(22-10)1-21-23(18,19)20/h2-4,6-7,10,16-17H,1H2,(H2,11,12,13)(H2,18,19,20)/t4-,6-,7-,10-/m1/s1"/></rdf:Bag></bqbiol:is><bqbiol:is>**

**<rdf:Bag>**

**<rdf:li rdf:resource="http://identifiers.org/INCHI/InChI=1S/C10H14N5O7P/c11-8-5-9(13-2-12-8)15(3-14-5)10-7(17)6(16)4(22-10)1-21-23(18,19)20/h2-4,6-7,10,16-17H,1H2,(H2,11,12,13)(H2,18,19,20)/p-2/t4-,6-,7-,10-/m1/s1"/></rdf:Bag></bqbiol:is></rdf:Description></rdf:RDF>**

**</annotation>**

**</species>**

**<species id="M_CE5344" constant="false" hasOnlySubstanceUnits="false" name="3(S),12(R)-dihydroxy-5-oxo-eicosa-8E,10E,14Z-trienoyl-CoA" metaid="281275fb-c05c-42fe-8cd3-3b9accec3b88" boundaryCondition="false" compartment="metaComp">**

**<notes>**

**<body xmlns="http://www.w3.org/1999/xhtml">**

**<p>FORMULA: C41H62N7O20P3S</p>**

**<p>CHARGE: 0</p>**

**</body>**

**</notes>**

**<annotation>**

**<rdf:RDF xmlns:rdf="http://www.w3.org/1999/02/22-rdf-syntax-ns#" xmlns:bqmodel="http://biomodels.net/model-qualifiers/" xmlns:bqbiol="http://biomodels.net/biology-qualifiers/">**

**<rdf:Description rdf:about="_281275fb-c05c-42fe-8cd3-3b9accec3b88"/></rdf:RDF>**

**</annotation>**

**</species>**

**<species id="M_mepi" constant="false" hasOnlySubstanceUnits="false" name="Metanephrine" metaid="b193aeba-b07a-4c31-871e-27324f8ba443" boundaryCondition="false" compartment="metaComp">**

**<notes>**

**<body xmlns="http://www.w3.org/1999/xhtml">**

**<p>FORMULA: C10H16NO3</p>**

**<p>CHARGE: 0</p>**

**<p>PUBCHEM.COMPOUND: 21100</p>**

**<p>INCHIKEY: JWJCTZKFYGDABJ-UHFFFAOYSA-N</p>**

**<p>HMDB: HMDB04063</p>**

**<p>KEGG.COMPOUND: C05588</p>**

**<p>CHEBI: CHEBI:144365 || CHEBI:6270</p>**

**<p>INCHI: InChI=1S/C10H15NO3/c1-11-6-9(13)7-3-4-8(12)10(5-7)14-2/h3-5,9,11-13H,6H2,1-2H3</p>**

**</body>**

**</notes>**

**<annotation>**

**<rdf:RDF xmlns:rdf="http://www.w3.org/1999/02/22-rdf-syntax-ns#" xmlns:bqmodel="http://biomodels.net/model-qualifiers/" xmlns:bqbiol="http://biomodels.net/biology-qualifiers/">**

**<rdf:Description rdf:about="b193aeba-b07a-4c31-871e-27324f8ba443">**

**<bqbiol:is>**

**<rdf:Bag>**

**<rdf:li rdf:resource="http://identifiers.org/PUBCHEM.COMPOUND/21100"/></rdf:Bag></bqbiol:is><bqbiol:is>**

**<rdf:Bag>**

**<rdf:li rdf:resource="http://identifiers.org/INCHIKEY/JWJCTZKFYGDABJ-UHFFFAOYSA-N"/></rdf:Bag></bqbiol:is><bqbiol:is>**

**<rdf:Bag>**

**<rdf:li rdf:resource="http://identifiers.org/HMDB/HMDB04063"/></rdf:Bag></bqbiol:is><bqbiol:is>**

**<rdf:Bag>**

**<rdf:li rdf:resource="http://identifiers.org/KEGG.COMPOUND/C05588"/></rdf:Bag></bqbiol:is><bqbiol:is>**

**<rdf:Bag>**

**<rdf:li rdf:resource="http://identifiers.org/CHEBI/CHEBI:144365"/></rdf:Bag></bqbiol:is><bqbiol:is>**

**<rdf:Bag>**

**<rdf:li rdf:resource="http://identifiers.org/CHEBI/CHEBI:6270"/></rdf:Bag></bqbiol:is><bqbiol:is>**

**<rdf:Bag>**

**<rdf:li rdf:resource="http://identifiers.org/INCHI/InChI=1S/C10H15NO3/c1-11-6-9(13)7-3-4-8(12)10(5-7)14-2/h3-5,9,11-13H,6H2,1-2H3"/></rdf:Bag></bqbiol:is></rdf:Description></rdf:RDF>**

**</annotation>**

**</species>**

**<species id="M_aicar" constant="false" hasOnlySubstanceUnits="false" name="5-Amino-1-(5-Phospho-D-ribosyl)imidazole-4-carboxamide" metaid="b25087e6-3b33-4eec-9d4b-0dc4806c3f56" boundaryCondition="false" compartment="metaComp">**

**<notes>**

**<body xmlns="http://www.w3.org/1999/xhtml">**

**<p>FORMULA: C9H13N4O8P</p>**

**<p>CHARGE: 0</p>**

**<p>PUBCHEM.COMPOUND: 65110</p>**

**<p>INCHIKEY: NOTGFIUVDGNKRI-UUOKFMHZSA-N</p>**

**<p>KEGG.COMPOUND: C04677</p>**

**<p>HMDB: HMDB01517</p>**

**<p>CHEBI: CHEBI:18406 || CHEBI:58475</p>**

**<p>INCHI: InChI=1S/C9H15N4O8P/c10-7-4(8(11)16)12-2-13(7)9-6(15)5(14)3(21-9)1-20-22(17,18)19/h2-3,5-6,9,14-15H,1,10H2,(H2,11,16)(H2,17,18,19)/t3-,5-,6-,9-/m1/s1 || InChI=1S/C9H15N4O8P/c10-7-4(8(11)16)12-2-13(7)9-6(15)5(14)3(21-9)1-20-22(17,18)19/h2-3,5-6,9,14-15H,1,10H2,(H2,11,16)(H2,17,18,19)/p-2/t3-,5-,6-,9-/m1/s1</p>**

**</body>**

**</notes>**

**<annotation>**

**<rdf:RDF xmlns:rdf="http://www.w3.org/1999/02/22-rdf-syntax-ns#" xmlns:bqmodel="http://biomodels.net/model-qualifiers/" xmlns:bqbiol="http://biomodels.net/biology-qualifiers/">**

**<rdf:Description rdf:about="b25087e6-3b33-4eec-9d4b-0dc4806c3f56">**

**<bqbiol:is>**

**<rdf:Bag>**

**<rdf:li rdf:resource="http://identifiers.org/PUBCHEM.COMPOUND/65110"/></rdf:Bag></bqbiol:is><bqbiol:is>**

**<rdf:Bag>**

**<rdf:li rdf:resource="http://identifiers.org/INCHIKEY/NOTGFIUVDGNKRI-UUOKFMHZSA-N"/></rdf:Bag></bqbiol:is><bqbiol:is>**

**<rdf:Bag>**

**<rdf:li rdf:resource="http://identifiers.org/KEGG.COMPOUND/C04677"/></rdf:Bag></bqbiol:is><bqbiol:is>**

**<rdf:Bag>**

**<rdf:li rdf:resource="http://identifiers.org/HMDB/HMDB01517"/></rdf:Bag></bqbiol:is><bqbiol:is>**

**<rdf:Bag>**

**<rdf:li rdf:resource="http://identifiers.org/CHEBI/CHEBI:18406"/></rdf:Bag></bqbiol:is><bqbiol:is>**

**<rdf:Bag>**

**<rdf:li rdf:resource="http://identifiers.org/CHEBI/CHEBI:58475"/></rdf:Bag></bqbiol:is><bqbiol:is>**

**<rdf:Bag>**

**<rdf:li rdf:resource="http://identifiers.org/INCHI/InChI=1S/C9H15N4O8P/c10-7-4(8(11)16)12-2-13(7)9-6(15)5(14)3(21-9)1-20-22(17,18)19/h2-3,5-6,9,14-15H,1,10H2,(H2,11,16)(H2,17,18,19)/t3-,5-,6-,9-/m1/s1"/></rdf:Bag></bqbiol:is><bqbiol:is>**

**<rdf:Bag>**

**<rdf:li rdf:resource="http://identifiers.org/INCHI/InChI=1S/C9H15N4O8P/c10-7-4(8(11)16)12-2-13(7)9-6(15)5(14)3(21-9)1-20-22(17,18)19/h2-3,5-6,9,14-15H,1,10H2,(H2,11,16)(H2,17,18,19)/p-2/t3-,5-,6-,9-/m1/s1"/></rdf:Bag></bqbiol:is></rdf:Description></rdf:RDF>**

**</annotation>**

**</species>**

**<species id="M_doldp_U" constant="false" hasOnlySubstanceUnits="false" name="Dolichol diphosphate, human uterine homolog" metaid="e592e3a2-0854-4d81-aacb-fcea57cb8d29" boundaryCondition="false" compartment="metaComp">**

**<notes>**

**<body xmlns="http://www.w3.org/1999/xhtml">**

**<p>FORMULA: C1025H1670O70P20</p>**

**<p>CHARGE: 0</p>**

**<p>KEGG.COMPOUND: C00621</p>**

**<p>CHEBI: CHEBI:15750</p>**

**</body>**

**</notes>**

**<annotation>**

**<rdf:RDF xmlns:rdf="http://www.w3.org/1999/02/22-rdf-syntax-ns#" xmlns:bqmodel="http://biomodels.net/model-qualifiers/" xmlns:bqbiol="http://biomodels.net/biology-qualifiers/">**

**<rdf:Description rdf:about="e592e3a2-0854-4d81-aacb-fcea57cb8d29">**

**<bqbiol:is>**

**<rdf:Bag>**

**<rdf:li rdf:resource="http://identifiers.org/KEGG.COMPOUND/C00621"/></rdf:Bag></bqbiol:is><bqbiol:is>**

**<rdf:Bag>**

**<rdf:li rdf:resource="http://identifiers.org/CHEBI/CHEBI:15750"/></rdf:Bag></bqbiol:is></rdf:Description></rdf:RDF>**

**</annotation>**

**</species>**

**<species id="M_retfa" constant="false" hasOnlySubstanceUnits="false" name="fatty acid retinol" metaid="d577401a-01f4-4e3d-a9ed-db3b4caf4bb7" boundaryCondition="false" compartment="metaComp">**

**<notes>**

**<body xmlns="http://www.w3.org/1999/xhtml">**

**<p>FORMULA: C20H29OFULLR2CO</p>**

**<p>CHARGE: 0</p>**

**</body>**

**</notes>**

**<annotation>**

**<rdf:RDF xmlns:rdf="http://www.w3.org/1999/02/22-rdf-syntax-ns#" xmlns:bqmodel="http://biomodels.net/model-qualifiers/" xmlns:bqbiol="http://biomodels.net/biology-qualifiers/">**

**<rdf:Description rdf:about="d577401a-01f4-4e3d-a9ed-db3b4caf4bb7"/></rdf:RDF>**

**</annotation>**

**</species>**

**<species id="M_dag_hs" constant="false" hasOnlySubstanceUnits="false" name="diglyceride" metaid="84d07265-97bd-49a2-b711-97acb90cebd2" boundaryCondition="false" compartment="metaComp">**

**<notes>**

**<body xmlns="http://www.w3.org/1999/xhtml">**

**<p>FORMULA: C3H6OFULLRCO2FULLR2CO2</p>**

**<p>CHARGE: 0</p>**

**<p>KEGG.COMPOUND: C00165</p>**

**<p>CHEBI: CHEBI:18035</p>**

**</body>**

**</notes>**

**<annotation>**

**<rdf:RDF xmlns:rdf="http://www.w3.org/1999/02/22-rdf-syntax-ns#" xmlns:bqmodel="http://biomodels.net/model-qualifiers/" xmlns:bqbiol="http://biomodels.net/biology-qualifiers/">**

**<rdf:Description rdf:about="_84d07265-97bd-49a2-b711-97acb90cebd2">**

**<bqbiol:is>**

**<rdf:Bag>**

**<rdf:li rdf:resource="http://identifiers.org/KEGG.COMPOUND/C00165"/></rdf:Bag></bqbiol:is><bqbiol:is>**

**<rdf:Bag>**

**<rdf:li rdf:resource="http://identifiers.org/CHEBI/CHEBI:18035"/></rdf:Bag></bqbiol:is></rdf:Description></rdf:RDF>**

**</annotation>**

**</species>**

**<species id="M_n2m2masn" constant="false" hasOnlySubstanceUnits="false" name="((N-acetyl-D-glucosaminyl)2-(alpha-D-mannosyl)2-beta-D-mannosyl-diacetylchitobiosyl)-L-asparagine (protein)" metaid="be63015d-185c-4021-bfa5-13231bc850c5" boundaryCondition="false" compartment="metaComp">**

**<notes>**

**<body xmlns="http://www.w3.org/1999/xhtml">**

**<p>FORMULA: C50H83N4O35X</p>**

**<p>CHARGE: 0</p>**

**<p>KEGG.COMPOUND: C11530</p>**

**</body>**

**</notes>**

**<annotation>**

**<rdf:RDF xmlns:rdf="http://www.w3.org/1999/02/22-rdf-syntax-ns#" xmlns:bqmodel="http://biomodels.net/model-qualifiers/" xmlns:bqbiol="http://biomodels.net/biology-qualifiers/">**

**<rdf:Description rdf:about="be63015d-185c-4021-bfa5-13231bc850c5">**

**<bqbiol:is>**

**<rdf:Bag>**

**<rdf:li rdf:resource="http://identifiers.org/KEGG.COMPOUND/C11530"/></rdf:Bag></bqbiol:is></rdf:Description></rdf:RDF>**

**</annotation>**

**</species>**

**<species id="M_fucfucgalacglcgal14acglcgalgluside_hs" constant="false" hasOnlySubstanceUnits="false" name="(Gal)3 (Glc)1 (GlcNAc)2 (LFuc)2 (Cer)1" metaid="7576e3b6-8fe0-4084-8b9f-2e14ab6356f1" boundaryCondition="false" compartment="metaComp">**

**<notes>**

**<body xmlns="http://www.w3.org/1999/xhtml">**

**<p>FORMULA: C70H122N3O40FULLRCO</p>**

**<p>CHARGE: 0</p>**

**</body>**

**</notes>**

**<annotation>**

**<rdf:RDF xmlns:rdf="http://www.w3.org/1999/02/22-rdf-syntax-ns#" xmlns:bqmodel="http://biomodels.net/model-qualifiers/" xmlns:bqbiol="http://biomodels.net/biology-qualifiers/">**

**<rdf:Description rdf:about="_7576e3b6-8fe0-4084-8b9f-2e14ab6356f1"/></rdf:RDF>**

**</annotation>**

**</species>**

**<species id="M_so4" constant="false" hasOnlySubstanceUnits="false" name="sulfate" metaid="6245f2fc-334d-403b-bfa6-db29faaff933" boundaryCondition="false" compartment="metaComp">**

**<notes>**

**<body xmlns="http://www.w3.org/1999/xhtml">**

**<p>FORMULA: O4S</p>**

**<p>CHARGE: 0</p>**

**<p>PUBCHEM.COMPOUND: 1117 || 1118</p>**

**<p>INCHIKEY: QAOWNCQODCNURD-UHFFFAOYSA-N</p>**

**<p>KEGG.COMPOUND: C00059</p>**

**<p>HMDB: HMDB01448</p>**

**<p>CHEBI: CHEBI:26836 || CHEBI:16189</p>**

**<p>INCHI: InChI=1S/H2O4S/c1-5(2,3)4/h(H2,1,2,3,4)/p-2 || InChI=1S/H2O4S/c1-5(2,3)4/h(H2,1,2,3,4)</p>**

**</body>**

**</notes>**

**<annotation>**

**<rdf:RDF xmlns:rdf="http://www.w3.org/1999/02/22-rdf-syntax-ns#" xmlns:bqmodel="http://biomodels.net/model-qualifiers/" xmlns:bqbiol="http://biomodels.net/biology-qualifiers/">**

**<rdf:Description rdf:about="_6245f2fc-334d-403b-bfa6-db29faaff933">**

**<bqbiol:is>**

**<rdf:Bag>**

**<rdf:li rdf:resource="http://identifiers.org/PUBCHEM.COMPOUND/1117"/></rdf:Bag></bqbiol:is><bqbiol:is>**

**<rdf:Bag>**

**<rdf:li rdf:resource="http://identifiers.org/PUBCHEM.COMPOUND/1118"/></rdf:Bag></bqbiol:is><bqbiol:is>**

**<rdf:Bag>**

**<rdf:li rdf:resource="http://identifiers.org/INCHIKEY/QAOWNCQODCNURD-UHFFFAOYSA-N"/></rdf:Bag></bqbiol:is><bqbiol:is>**

**<rdf:Bag>**

**<rdf:li rdf:resource="http://identifiers.org/KEGG.COMPOUND/C00059"/></rdf:Bag></bqbiol:is><bqbiol:is>**

**<rdf:Bag>**

**<rdf:li rdf:resource="http://identifiers.org/HMDB/HMDB01448"/></rdf:Bag></bqbiol:is><bqbiol:is>**

**<rdf:Bag>**

**<rdf:li rdf:resource="http://identifiers.org/CHEBI/CHEBI:26836"/></rdf:Bag></bqbiol:is><bqbiol:is>**

**<rdf:Bag>**

**<rdf:li rdf:resource="http://identifiers.org/CHEBI/CHEBI:16189"/></rdf:Bag></bqbiol:is><bqbiol:is>**

**<rdf:Bag>**

**<rdf:li rdf:resource="http://identifiers.org/INCHI/InChI=1S/H2O4S/c1-5(2,3)4/h(H2,1,2,3,4)/p-2"/></rdf:Bag></bqbiol:is><bqbiol:is>**

**<rdf:Bag>**

**<rdf:li rdf:resource="http://identifiers.org/INCHI/InChI=1S/H2O4S/c1-5(2,3)4/h(H2,1,2,3,4)"/></rdf:Bag></bqbiol:is></rdf:Description></rdf:RDF>**

**</annotation>**

**</species>**

**<species id="M_so3" constant="false" hasOnlySubstanceUnits="false" name="hydrogensulfite" metaid="44e2d941-1795-4ebd-8ebc-6f89b32da220" boundaryCondition="false" compartment="metaComp">**

**<notes>**

**<body xmlns="http://www.w3.org/1999/xhtml">**

**<p>FORMULA: O3S</p>**

**<p>CHARGE: 0</p>**

**<p>PUBCHEM.COMPOUND: 1099 || 1100</p>**

**<p>INCHIKEY: LSNNMFCWUKXFEE-UHFFFAOYSA-M</p>**

**<p>KEGG.COMPOUND: C00094</p>**

**<p>HMDB: HMDB00240</p>**

**<p>CHEBI: CHEBI:17359 || CHEBI:17137 || CHEBI:48854</p>**

**<p>INCHI: InChI=1S/H2O3S/c1-4(2)3/h(H2,1,2,3)/p-2 || InChI=1S/H2O3S/c1-4(2)3/h(H2,1,2,3) || InChI=1S/H2O3S/c1-4(2)3/h(H2,1,2,3)/p-1</p>**

**</body>**

**</notes>**

**<annotation>**

**<rdf:RDF xmlns:rdf="http://www.w3.org/1999/02/22-rdf-syntax-ns#" xmlns:bqmodel="http://biomodels.net/model-qualifiers/" xmlns:bqbiol="http://biomodels.net/biology-qualifiers/">**

**<rdf:Description rdf:about="_44e2d941-1795-4ebd-8ebc-6f89b32da220">**

**<bqbiol:is>**

**<rdf:Bag>**

**<rdf:li rdf:resource="http://identifiers.org/PUBCHEM.COMPOUND/1099"/></rdf:Bag></bqbiol:is><bqbiol:is>**

**<rdf:Bag>**

**<rdf:li rdf:resource="http://identifiers.org/PUBCHEM.COMPOUND/1100"/></rdf:Bag></bqbiol:is><bqbiol:is>**

**<rdf:Bag>**

**<rdf:li rdf:resource="http://identifiers.org/INCHIKEY/LSNNMFCWUKXFEE-UHFFFAOYSA-M"/></rdf:Bag></bqbiol:is><bqbiol:is>**

**<rdf:Bag>**

**<rdf:li rdf:resource="http://identifiers.org/KEGG.COMPOUND/C00094"/></rdf:Bag></bqbiol:is><bqbiol:is>**

**<rdf:Bag>**

**<rdf:li rdf:resource="http://identifiers.org/HMDB/HMDB00240"/></rdf:Bag></bqbiol:is><bqbiol:is>**

**<rdf:Bag>**

**<rdf:li rdf:resource="http://identifiers.org/CHEBI/CHEBI:17359"/></rdf:Bag></bqbiol:is><bqbiol:is>**

**<rdf:Bag>**

**<rdf:li rdf:resource="http://identifiers.org/CHEBI/CHEBI:17137"/></rdf:Bag></bqbiol:is><bqbiol:is>**

**<rdf:Bag>**

**<rdf:li rdf:resource="http://identifiers.org/CHEBI/CHEBI:48854"/></rdf:Bag></bqbiol:is><bqbiol:is>**

**<rdf:Bag>**

**<rdf:li rdf:resource="http://identifiers.org/INCHI/InChI=1S/H2O3S/c1-4(2)3/h(H2,1,2,3)/p-2"/></rdf:Bag></bqbiol:is><bqbiol:is>**

**<rdf:Bag>**

**<rdf:li rdf:resource="http://identifiers.org/INCHI/InChI=1S/H2O3S/c1-4(2)3/h(H2,1,2,3)"/></rdf:Bag></bqbiol:is><bqbiol:is>**

**<rdf:Bag>**

**<rdf:li rdf:resource="http://identifiers.org/INCHI/InChI=1S/H2O3S/c1-4(2)3/h(H2,1,2,3)/p-1"/></rdf:Bag></bqbiol:is></rdf:Description></rdf:RDF>**

**</annotation>**

**</species>**

**<species id="M_dlnlcg" constant="false" hasOnlySubstanceUnits="false" name="dihomo-gamma-linolenic acid (n-6)" metaid="7a44a3d9-c274-4358-9370-029135dd86a7" boundaryCondition="false" compartment="metaComp">**

**<notes>**

**<body xmlns="http://www.w3.org/1999/xhtml">**

**<p>FORMULA: C20H33O2</p>**

**<p>CHARGE: 0</p>**

**<p>PUBCHEM.COMPOUND: 5280581 || 3011</p>**

**<p>KEGG.COMPOUND: C03242</p>**

**<p>HMDB: HMDB02925</p>**

**<p>CHEBI: CHEBI:53486</p>**

**<p>INCHI: InChI=1S/C20H34O2/c1-2-3-4-5-6-7-8-9-10-11-12-13-14-15-16-17-18-19-20(21)22/h6-7,9-10,12-13H,2-5,8,11,14-19H2,1H3,(H,21,22)/b7-6-,10-9-,13-12- || InChI=1S/C20H34O2/c1-2-3-4-5-6-7-8-9-10-11-12-13-14-15-16-17-18-19-20(21)22/h6-7,9-10,12-13H,2-5,8,11,14-19H2,1H3,(H,21,22)/p-1/b7-6-,10-9-,13-12-</p>**

**</body>**

**</notes>**

**<annotation>**

**<rdf:RDF xmlns:rdf="http://www.w3.org/1999/02/22-rdf-syntax-ns#" xmlns:bqmodel="http://biomodels.net/model-qualifiers/" xmlns:bqbiol="http://biomodels.net/biology-qualifiers/">**

**<rdf:Description rdf:about="_7a44a3d9-c274-4358-9370-029135dd86a7">**

**<bqbiol:is>**

**<rdf:Bag>**

**<rdf:li rdf:resource="http://identifiers.org/PUBCHEM.COMPOUND/5280581"/></rdf:Bag></bqbiol:is><bqbiol:is>**

**<rdf:Bag>**

**<rdf:li rdf:resource="http://identifiers.org/PUBCHEM.COMPOUND/3011"/></rdf:Bag></bqbiol:is><bqbiol:is>**

**<rdf:Bag>**

**<rdf:li rdf:resource="http://identifiers.org/KEGG.COMPOUND/C03242"/></rdf:Bag></bqbiol:is><bqbiol:is>**

**<rdf:Bag>**

**<rdf:li rdf:resource="http://identifiers.org/HMDB/HMDB02925"/></rdf:Bag></bqbiol:is><bqbiol:is>**

**<rdf:Bag>**

**<rdf:li rdf:resource="http://identifiers.org/CHEBI/CHEBI:53486"/></rdf:Bag></bqbiol:is><bqbiol:is>**

**<rdf:Bag>**

**<rdf:li rdf:resource="http://identifiers.org/INCHI/InChI=1S/C20H34O2/c1-2-3-4-5-6-7-8-9-10-11-12-13-14-15-16-17-18-19-20(21)22/h6-7,9-10,12-13H,2-5,8,11,14-19H2,1H3,(H,21,22)/b7-6-,10-9-,13-12-"/></rdf:Bag></bqbiol:is><bqbiol:is>**

**<rdf:Bag>**

**<rdf:li rdf:resource="http://identifiers.org/INCHI/InChI=1S/C20H34O2/c1-2-3-4-5-6-7-8-9-10-11-12-13-14-15-16-17-18-19-20(21)22/h6-7,9-10,12-13H,2-5,8,11,14-19H2,1H3,(H,21,22)/p-1/b7-6-,10-9-,13-12-"/></rdf:Bag></bqbiol:is></rdf:Description></rdf:RDF>**

**</annotation>**

**</species>**

**<species id="M_ru5p_D" constant="false" hasOnlySubstanceUnits="false" name="D-ribulose 5-phosphate(2-)" metaid="08b8bb66-f58d-4d88-885e-7a0a1a7e5088" boundaryCondition="false" compartment="metaComp">**

**<notes>**

**<body xmlns="http://www.w3.org/1999/xhtml">**

**<p>FORMULA: C5H9O8P</p>**

**<p>CHARGE: 0</p>**

**<p>PUBCHEM.COMPOUND: 53477706 || 439184</p>**

**<p>KEGG.COMPOUND: C00199 || C00117</p>**

**<p>HMDB: HMDB00618</p>**

**<p>CHEBI: CHEBI:17363 || CHEBI:58121 || CHEBI:17797</p>**

**<p>INCHI: InChI=1S/C5H11O8P/c6-1-3(7)5(9)4(8)2-13-14(10,11)12/h4-6,8-9H,1-2H2,(H2,10,11,12)/t4-,5+/m1/s1 || InChI=1S/C5H11O8P/c6-3-2(1-12-14(9,10)11)13-5(8)4(3)7/h2-8H,1H2,(H2,9,10,11)/t2-,3-,4-,5?/m0/s1 || InChI=1S/C5H11O8P/c6-1-3(7)5(9)4(8)2-13-14(10,11)12/h4-6,8-9H,1-2H2,(H2,10,11,12)/p-2/t4-,5+/m1/s1</p>**

**</body>**

**</notes>**

**<annotation>**

**<rdf:RDF xmlns:rdf="http://www.w3.org/1999/02/22-rdf-syntax-ns#" xmlns:bqmodel="http://biomodels.net/model-qualifiers/" xmlns:bqbiol="http://biomodels.net/biology-qualifiers/">**

**<rdf:Description rdf:about="_08b8bb66-f58d-4d88-885e-7a0a1a7e5088">**

**<bqbiol:is>**

**<rdf:Bag>**

**<rdf:li rdf:resource="http://identifiers.org/PUBCHEM.COMPOUND/53477706"/></rdf:Bag></bqbiol:is><bqbiol:is>**

**<rdf:Bag>**

**<rdf:li rdf:resource="http://identifiers.org/PUBCHEM.COMPOUND/439184"/></rdf:Bag></bqbiol:is><bqbiol:is>**

**<rdf:Bag>**

**<rdf:li rdf:resource="http://identifiers.org/KEGG.COMPOUND/C00199"/></rdf:Bag></bqbiol:is><bqbiol:is>**

**<rdf:Bag>**

**<rdf:li rdf:resource="http://identifiers.org/KEGG.COMPOUND/C00117"/></rdf:Bag></bqbiol:is><bqbiol:is>**

**<rdf:Bag>**

**<rdf:li rdf:resource="http://identifiers.org/HMDB/HMDB00618"/></rdf:Bag></bqbiol:is><bqbiol:is>**

**<rdf:Bag>**

**<rdf:li rdf:resource="http://identifiers.org/CHEBI/CHEBI:17363"/></rdf:Bag></bqbiol:is><bqbiol:is>**

**<rdf:Bag>**

**<rdf:li rdf:resource="http://identifiers.org/CHEBI/CHEBI:58121"/></rdf:Bag></bqbiol:is><bqbiol:is>**

**<rdf:Bag>**

**<rdf:li rdf:resource="http://identifiers.org/CHEBI/CHEBI:17797"/></rdf:Bag></bqbiol:is><bqbiol:is>**

**<rdf:Bag>**

**<rdf:li rdf:resource="http://identifiers.org/INCHI/InChI=1S/C5H11O8P/c6-1-3(7)5(9)4(8)2-13-14(10,11)12/h4-6,8-9H,1-2H2,(H2,10,11,12)/t4-,5+/m1/s1"/></rdf:Bag></bqbiol:is><bqbiol:is>**

**<rdf:Bag>**

**<rdf:li rdf:resource="http://identifiers.org/INCHI/InChI=1S/C5H11O8P/c6-3-2(1-12-14(9,10)11)13-5(8)4(3)7/h2-8H,1H2,(H2,9,10,11)/t2-,3-,4-,5?/m0/s1"/></rdf:Bag></bqbiol:is><bqbiol:is>**

**<rdf:Bag>**

**<rdf:li rdf:resource="http://identifiers.org/INCHI/InChI=1S/C5H11O8P/c6-1-3(7)5(9)4(8)2-13-14(10,11)12/h4-6,8-9H,1-2H2,(H2,10,11,12)/p-2/t4-,5+/m1/s1"/></rdf:Bag></bqbiol:is></rdf:Description></rdf:RDF>**

**</annotation>**

**</species>**

**<species id="M_q10" constant="false" hasOnlySubstanceUnits="false" name="ubiquinone-10" metaid="482077e3-3c95-493f-bb7a-90be43d3cace" boundaryCondition="false" compartment="metaComp">**

**<notes>**

**<body xmlns="http://www.w3.org/1999/xhtml">**

**<p>FORMULA: C59H90O4</p>**

**<p>CHARGE: 0</p>**

**<p>PUBCHEM.COMPOUND: 12303768 || 5281915</p>**

**<p>INCHIKEY: ACTIUHUUMQJHFO-UPTCCGCDSA-N</p>**

**<p>KEGG.COMPOUND: C11378</p>**

**<p>HMDB: HMDB01072</p>**

**<p>CHEBI: CHEBI:46245</p>**

**<p>INCHI: InChI=1S/C59H90O4/c1-44(2)24-15-25-45(3)26-16-27-46(4)28-17-29-47(5)30-18-31-48(6)32-19-33-49(7)34-20-35-50(8)36-21-37-51(9)38-22-39-52(10)40-23-41-53(11)42-43-55-54(12)56(60)58(62-13)59(63-14)57(55)61/h24,26,28,30,32,34,36,38,40,42H,15-23,25,27,29,31,33,35,37,39,41,43H2,1-14H3/b45-26+,46-28+,47-30+,48-32+,49-34+,50-36+,51-38+,52-40+,53-42+</p>**

**</body>**

**</notes>**

**<annotation>**

**<rdf:RDF xmlns:rdf="http://www.w3.org/1999/02/22-rdf-syntax-ns#" xmlns:bqmodel="http://biomodels.net/model-qualifiers/" xmlns:bqbiol="http://biomodels.net/biology-qualifiers/">**

**<rdf:Description rdf:about="_482077e3-3c95-493f-bb7a-90be43d3cace">**

**<bqbiol:is>**

**<rdf:Bag>**

**<rdf:li rdf:resource="http://identifiers.org/PUBCHEM.COMPOUND/12303768"/></rdf:Bag></bqbiol:is><bqbiol:is>**

**<rdf:Bag>**

**<rdf:li rdf:resource="http://identifiers.org/PUBCHEM.COMPOUND/5281915"/></rdf:Bag></bqbiol:is><bqbiol:is>**

**<rdf:Bag>**

**<rdf:li rdf:resource="http://identifiers.org/INCHIKEY/ACTIUHUUMQJHFO-UPTCCGCDSA-N"/></rdf:Bag></bqbiol:is><bqbiol:is>**

**<rdf:Bag>**

**<rdf:li rdf:resource="http://identifiers.org/KEGG.COMPOUND/C11378"/></rdf:Bag></bqbiol:is><bqbiol:is>**

**<rdf:Bag>**

**<rdf:li rdf:resource="http://identifiers.org/HMDB/HMDB01072"/></rdf:Bag></bqbiol:is><bqbiol:is>**

**<rdf:Bag>**

**<rdf:li rdf:resource="http://identifiers.org/CHEBI/CHEBI:46245"/></rdf:Bag></bqbiol:is><bqbiol:is>**

**<rdf:Bag>**

**<rdf:li rdf:resource="http://identifiers.org/INCHI/InChI=1S/C59H90O4/c1-44(2)24-15-25-45(3)26-16-27-46(4)28-17-29-47(5)30-18-31-48(6)32-19-33-49(7)34-20-35-50(8)36-21-37-51(9)38-22-39-52(10)40-23-41-53(11)42-43-55-54(12)56(60)58(62-13)59(63-14)57(55)61/h24,26,28,30,32,34,36,38,40,42H,15-23,25,27,29,31,33,35,37,39,41,43H2,1-14H3/b45-26+,46-28+,47-30+,48-32+,49-34+,50-36+,51-38+,52-40+,53-42+"/></rdf:Bag></bqbiol:is></rdf:Description></rdf:RDF>**

**</annotation>**

**</species>**

**<species id="M_CE5352" constant="false" hasOnlySubstanceUnits="false" name="6,7-dihydro-leukotriene B4" metaid="509fff27-2f53-4c97-9cb7-10f65d6b4ab0" boundaryCondition="false" compartment="metaComp">**

**<notes>**

**<body xmlns="http://www.w3.org/1999/xhtml">**

**<p>FORMULA: C20H33O4</p>**

**<p>CHARGE: 0</p>**

**</body>**

**</notes>**

**<annotation>**

**<rdf:RDF xmlns:rdf="http://www.w3.org/1999/02/22-rdf-syntax-ns#" xmlns:bqmodel="http://biomodels.net/model-qualifiers/" xmlns:bqbiol="http://biomodels.net/biology-qualifiers/">**

**<rdf:Description rdf:about="_509fff27-2f53-4c97-9cb7-10f65d6b4ab0"/></rdf:RDF>**

**</annotation>**

**</species>**

**<species id="M_prgstrn" constant="false" hasOnlySubstanceUnits="false" name="progesterone" metaid="45e69402-a3ef-486e-a173-05daf67eb022" boundaryCondition="false" compartment="metaComp">**

**<notes>**

**<body xmlns="http://www.w3.org/1999/xhtml">**

**<p>FORMULA: C21H30O2</p>**

**<p>CHARGE: 0</p>**

**<p>PUBCHEM.COMPOUND: 16401562 || 5994 || 4920 || 5320716</p>**

**<p>INCHIKEY: RJKFOVLPORLFTN-LEKSSAKUSA-N</p>**

**<p>HMDB: HMDB01830</p>**

**<p>KEGG.COMPOUND: C00410</p>**

**<p>CHEBI: CHEBI:17026</p>**

**<p>INCHI: InChI=1S/C21H30O2/c1-13(22)17-6-7-18-16-5-4-14-12-15(23)8-10-20(14,2)19(16)9-11-21(17,18)3/h12,16-19H,4-11H2,1-3H3/t16-,17+,18-,19-,20-,21+/m0/s1</p>**

**</body>**

**</notes>**

**<annotation>**

**<rdf:RDF xmlns:rdf="http://www.w3.org/1999/02/22-rdf-syntax-ns#" xmlns:bqmodel="http://biomodels.net/model-qualifiers/" xmlns:bqbiol="http://biomodels.net/biology-qualifiers/">**

**<rdf:Description rdf:about="_45e69402-a3ef-486e-a173-05daf67eb022">**

**<bqbiol:is>**

**<rdf:Bag>**

**<rdf:li rdf:resource="http://identifiers.org/PUBCHEM.COMPOUND/16401562"/></rdf:Bag></bqbiol:is><bqbiol:is>**

**<rdf:Bag>**

**<rdf:li rdf:resource="http://identifiers.org/PUBCHEM.COMPOUND/5994"/></rdf:Bag></bqbiol:is><bqbiol:is>**

**<rdf:Bag>**

**<rdf:li rdf:resource="http://identifiers.org/PUBCHEM.COMPOUND/4920"/></rdf:Bag></bqbiol:is><bqbiol:is>**

**<rdf:Bag>**

**<rdf:li rdf:resource="http://identifiers.org/PUBCHEM.COMPOUND/5320716"/></rdf:Bag></bqbiol:is><bqbiol:is>**

**<rdf:Bag>**

**<rdf:li rdf:resource="http://identifiers.org/INCHIKEY/RJKFOVLPORLFTN-LEKSSAKUSA-N"/></rdf:Bag></bqbiol:is><bqbiol:is>**

**<rdf:Bag>**

**<rdf:li rdf:resource="http://identifiers.org/HMDB/HMDB01830"/></rdf:Bag></bqbiol:is><bqbiol:is>**

**<rdf:Bag>**

**<rdf:li rdf:resource="http://identifiers.org/KEGG.COMPOUND/C00410"/></rdf:Bag></bqbiol:is><bqbiol:is>**

**<rdf:Bag>**

**<rdf:li rdf:resource="http://identifiers.org/CHEBI/CHEBI:17026"/></rdf:Bag></bqbiol:is><bqbiol:is>**

**<rdf:Bag>**

**<rdf:li rdf:resource="http://identifiers.org/INCHI/InChI=1S/C21H30O2/c1-13(22)17-6-7-18-16-5-4-14-12-15(23)8-10-20(14,2)19(16)9-11-21(17,18)3/h12,16-19H,4-11H2,1-3H3/t16-,17+,18-,19-,20-,21+/m0/s1"/></rdf:Bag></bqbiol:is></rdf:Description></rdf:RDF>**

**</annotation>**

**</species>**

**<species id="M_onpthl" constant="false" hasOnlySubstanceUnits="false" name="naphthalene epoxide" metaid="0d37b815-c61e-4ad5-ad3d-abf7b3e6580e" boundaryCondition="false" compartment="metaComp">**

**<notes>**

**<body xmlns="http://www.w3.org/1999/xhtml">**

**<p>FORMULA: C10H8O</p>**

**<p>CHARGE: 0</p>**

**<p>PUBCHEM.COMPOUND: 108063</p>**

**<p>INCHIKEY: XQIJIALOJPIKGX-UHFFFAOYSA-N</p>**

**<p>HMDB: HMDB06215</p>**

**<p>CHEBI: CHEBI:52431</p>**

**<p>INCHI: InChI=1/C10H8O/c1-2-4-8-7(3-1)5-6-9-10(8)11-9/h1-6,9-10H || InChI=1S/C10H8O/c1-2-4-8-7(3-1)5-6-9-10(8)11-9/h1-6,9-10H</p>**

**</body>**

**</notes>**

**<annotation>**

**<rdf:RDF xmlns:rdf="http://www.w3.org/1999/02/22-rdf-syntax-ns#" xmlns:bqmodel="http://biomodels.net/model-qualifiers/" xmlns:bqbiol="http://biomodels.net/biology-qualifiers/">**

**<rdf:Description rdf:about="_0d37b815-c61e-4ad5-ad3d-abf7b3e6580e">**

**<bqbiol:is>**

**<rdf:Bag>**

**<rdf:li rdf:resource="http://identifiers.org/PUBCHEM.COMPOUND/108063"/></rdf:Bag></bqbiol:is><bqbiol:is>**

**<rdf:Bag>**

**<rdf:li rdf:resource="http://identifiers.org/INCHIKEY/XQIJIALOJPIKGX-UHFFFAOYSA-N"/></rdf:Bag></bqbiol:is><bqbiol:is>**

**<rdf:Bag>**

**<rdf:li rdf:resource="http://identifiers.org/HMDB/HMDB06215"/></rdf:Bag></bqbiol:is><bqbiol:is>**

**<rdf:Bag>**

**<rdf:li rdf:resource="http://identifiers.org/CHEBI/CHEBI:52431"/></rdf:Bag></bqbiol:is><bqbiol:is>**

**<rdf:Bag>**

**<rdf:li rdf:resource="http://identifiers.org/INCHI/InChI=1/C10H8O/c1-2-4-8-7(3-1)5-6-9-10(8)11-9/h1-6,9-10H"/></rdf:Bag></bqbiol:is><bqbiol:is>**

**<rdf:Bag>**

**<rdf:li rdf:resource="http://identifiers.org/INCHI/InChI=1S/C10H8O/c1-2-4-8-7(3-1)5-6-9-10(8)11-9/h1-6,9-10H"/></rdf:Bag></bqbiol:is></rdf:Description></rdf:RDF>**

**</annotation>**

**</species>**

**<species id="M_meoh" constant="false" hasOnlySubstanceUnits="false" name="methanol" metaid="4ad2ebea-74ae-43ef-a9c5-cf11b5f5d70f" boundaryCondition="false" compartment="metaComp">**

**<notes>**

**<body xmlns="http://www.w3.org/1999/xhtml">**

**<p>FORMULA: CH4O1</p>**

**<p>CHARGE: 0</p>**

**<p>PUBCHEM.COMPOUND: 887</p>**

**<p>INCHIKEY: OKKJLVBELUTLKV-UHFFFAOYSA-N</p>**

**<p>KEGG.COMPOUND: C00132</p>**

**<p>HMDB: HMDB01875</p>**

**<p>CHEBI: CHEBI:17790</p>**

**<p>INCHI: InChI=1S/CH4O/c1-2/h2H,1H3</p>**

**</body>**

**</notes>**

**<annotation>**

**<rdf:RDF xmlns:rdf="http://www.w3.org/1999/02/22-rdf-syntax-ns#" xmlns:bqmodel="http://biomodels.net/model-qualifiers/" xmlns:bqbiol="http://biomodels.net/biology-qualifiers/">**

**<rdf:Description rdf:about="_4ad2ebea-74ae-43ef-a9c5-cf11b5f5d70f">**

**<bqbiol:is>**

**<rdf:Bag>**

**<rdf:li rdf:resource="http://identifiers.org/PUBCHEM.COMPOUND/887"/></rdf:Bag></bqbiol:is><bqbiol:is>**

**<rdf:Bag>**

**<rdf:li rdf:resource="http://identifiers.org/INCHIKEY/OKKJLVBELUTLKV-UHFFFAOYSA-N"/></rdf:Bag></bqbiol:is><bqbiol:is>**

**<rdf:Bag>**

**<rdf:li rdf:resource="http://identifiers.org/KEGG.COMPOUND/C00132"/></rdf:Bag></bqbiol:is><bqbiol:is>**

**<rdf:Bag>**

**<rdf:li rdf:resource="http://identifiers.org/HMDB/HMDB01875"/></rdf:Bag></bqbiol:is><bqbiol:is>**

**<rdf:Bag>**

**<rdf:li rdf:resource="http://identifiers.org/CHEBI/CHEBI:17790"/></rdf:Bag></bqbiol:is><bqbiol:is>**

**<rdf:Bag>**

**<rdf:li rdf:resource="http://identifiers.org/INCHI/InChI=1S/CH4O/c1-2/h2H,1H3"/></rdf:Bag></bqbiol:is></rdf:Description></rdf:RDF>**

**</annotation>**

**</species>**

**<species id="M_hs_pre13" constant="false" hasOnlySubstanceUnits="false" name="GlcN2S-IdoA-GlcN2,6diS-GlcA2S-GlcN2,6diS-IdoA2S-GlcN2,6diS-GlcA-GlcNAc-GlcA-Gal-Gal-Xyl-L-Ser (protein)" metaid="6cb882da-ed4b-44fa-85be-e85b9afa0fab" boundaryCondition="false" compartment="metaComp">**

**<notes>**

**<body xmlns="http://www.w3.org/1999/xhtml">**

**<p>FORMULA: C79H116N5O92S9X</p>**

**<p>CHARGE: 0</p>**

**</body>**

**</notes>**

**<annotation>**

**<rdf:RDF xmlns:rdf="http://www.w3.org/1999/02/22-rdf-syntax-ns#" xmlns:bqmodel="http://biomodels.net/model-qualifiers/" xmlns:bqbiol="http://biomodels.net/biology-qualifiers/">**

**<rdf:Description rdf:about="_6cb882da-ed4b-44fa-85be-e85b9afa0fab"/></rdf:RDF>**

**</annotation>**

**</species>**

**<species id="M_ksi_pre31" constant="false" hasOnlySubstanceUnits="false" name="keratan sulfate I biosynthesis, precursor 31" metaid="c2e8502d-ce46-467f-a783-4765a6a2639d" boundaryCondition="false" compartment="metaComp">**

**<notes>**

**<body xmlns="http://www.w3.org/1999/xhtml">**

**<p>FORMULA: C227H363N16O189S9X</p>**

**<p>CHARGE: 0</p>**

**</body>**

**</notes>**

**<annotation>**

**<rdf:RDF xmlns:rdf="http://www.w3.org/1999/02/22-rdf-syntax-ns#" xmlns:bqmodel="http://biomodels.net/model-qualifiers/" xmlns:bqbiol="http://biomodels.net/biology-qualifiers/">**

**<rdf:Description rdf:about="c2e8502d-ce46-467f-a783-4765a6a2639d"/></rdf:RDF>**

**</annotation>**

**</species>**

**<species id="M_dectricoa" constant="false" hasOnlySubstanceUnits="false" name="2,4,7-decatrienoylcoa" metaid="52eca926-5d3c-44b6-ba7f-651d41bc097c" boundaryCondition="false" compartment="metaComp">**

**<notes>**

**<body xmlns="http://www.w3.org/1999/xhtml">**

**<p>FORMULA: C31H44N7O17P3S</p>**

**<p>CHARGE: 0</p>**

**</body>**

**</notes>**

**<annotation>**

**<rdf:RDF xmlns:rdf="http://www.w3.org/1999/02/22-rdf-syntax-ns#" xmlns:bqmodel="http://biomodels.net/model-qualifiers/" xmlns:bqbiol="http://biomodels.net/biology-qualifiers/">**

**<rdf:Description rdf:about="_52eca926-5d3c-44b6-ba7f-651d41bc097c"/></rdf:RDF>**

**</annotation>**

**</species>**

**<species id="M_ksi_pre30" constant="false" hasOnlySubstanceUnits="false" name="keratan sulfate I biosynthesis, precursor 30" metaid="e9f63f40-8cd6-4985-93d0-97c2af4a0eff" boundaryCondition="false" compartment="metaComp">**

**<notes>**

**<body xmlns="http://www.w3.org/1999/xhtml">**

**<p>FORMULA: C219H350N15O184S9X</p>**

**<p>CHARGE: 0</p>**

**</body>**

**</notes>**

**<annotation>**

**<rdf:RDF xmlns:rdf="http://www.w3.org/1999/02/22-rdf-syntax-ns#" xmlns:bqmodel="http://biomodels.net/model-qualifiers/" xmlns:bqbiol="http://biomodels.net/biology-qualifiers/">**

**<rdf:Description rdf:about="e9f63f40-8cd6-4985-93d0-97c2af4a0eff"/></rdf:RDF>**

**</annotation>**

**</species>**

**<species id="M_hs_pre12" constant="false" hasOnlySubstanceUnits="false" name="GlcN2S-IdoA-GlcN2S-GlcA2S-GlcN2S-IdoA2S-GlcN2S-GlcA-GlcNAc-GlcA-Gal-Gal-Xyl-L-Ser (protein)" metaid="5d2ec3bc-cf07-47e9-aef0-2f02976708c2" boundaryCondition="false" compartment="metaComp">**

**<notes>**

**<body xmlns="http://www.w3.org/1999/xhtml">**

**<p>FORMULA: C79H119N5O83S6X</p>**

**<p>CHARGE: 0</p>**

**</body>**

**</notes>**

**<annotation>**

**<rdf:RDF xmlns:rdf="http://www.w3.org/1999/02/22-rdf-syntax-ns#" xmlns:bqmodel="http://biomodels.net/model-qualifiers/" xmlns:bqbiol="http://biomodels.net/biology-qualifiers/">**

**<rdf:Description rdf:about="_5d2ec3bc-cf07-47e9-aef0-2f02976708c2"/></rdf:RDF>**

**</annotation>**

**</species>**

**<species id="M_hs_pre11" constant="false" hasOnlySubstanceUnits="false" name="(GlcN2S-IdoA-GlcN2S-GlcA)2-GlcNAc-GlcA-Gal-Gal-Xyl-L-Ser (protein)" metaid="0b335ee9-634d-4bec-ac5b-9db50965a442" boundaryCondition="false" compartment="metaComp">**

**<notes>**

**<body xmlns="http://www.w3.org/1999/xhtml">**

**<p>FORMULA: C79H121N5O77S4X</p>**

**<p>CHARGE: 0</p>**

**</body>**

**</notes>**

**<annotation>**

**<rdf:RDF xmlns:rdf="http://www.w3.org/1999/02/22-rdf-syntax-ns#" xmlns:bqmodel="http://biomodels.net/model-qualifiers/" xmlns:bqbiol="http://biomodels.net/biology-qualifiers/">**

**<rdf:Description rdf:about="_0b335ee9-634d-4bec-ac5b-9db50965a442"/></rdf:RDF>**

**</annotation>**

**</species>**

**<species id="M_ksi_pre33" constant="false" hasOnlySubstanceUnits="false" name="keratan sulfate I biosynthesis, precursor 33" metaid="4341e72e-566b-45cf-9ec8-773ffd355e80" boundaryCondition="false" compartment="metaComp">**

**<notes>**

**<body xmlns="http://www.w3.org/1999/xhtml">**

**<p>FORMULA: C233H372N16O197S10X</p>**

**<p>CHARGE: 0</p>**

**</body>**

**</notes>**

**<annotation>**

**<rdf:RDF xmlns:rdf="http://www.w3.org/1999/02/22-rdf-syntax-ns#" xmlns:bqmodel="http://biomodels.net/model-qualifiers/" xmlns:bqbiol="http://biomodels.net/biology-qualifiers/">**

**<rdf:Description rdf:about="_4341e72e-566b-45cf-9ec8-773ffd355e80"/></rdf:RDF>**

**</annotation>**

**</species>**

**<species id="M_hs_pre10" constant="false" hasOnlySubstanceUnits="false" name="(GlcN2S-GlcA)4-GlcNAc-GlcA-Gal-Gal-Xyl-L-Ser (protein)" metaid="e1129a8d-82af-48c7-ab68-97cbb1baf04e" boundaryCondition="false" compartment="metaComp">**

**<notes>**

**<body xmlns="http://www.w3.org/1999/xhtml">**

**<p>FORMULA: C79H121N5O77S4X</p>**

**<p>CHARGE: 0</p>**

**</body>**

**</notes>**

**<annotation>**

**<rdf:RDF xmlns:rdf="http://www.w3.org/1999/02/22-rdf-syntax-ns#" xmlns:bqmodel="http://biomodels.net/model-qualifiers/" xmlns:bqbiol="http://biomodels.net/biology-qualifiers/">**

**<rdf:Description rdf:about="e1129a8d-82af-48c7-ab68-97cbb1baf04e"/></rdf:RDF>**

**</annotation>**

**</species>**

**<species id="M_ksi_pre32" constant="false" hasOnlySubstanceUnits="false" name="keratan sulfate I biosynthesis, precursor 32" metaid="63bf5b3f-efcf-460d-bfbf-a3f4bdc99f89" boundaryCondition="false" compartment="metaComp">**

**<notes>**

**<body xmlns="http://www.w3.org/1999/xhtml">**

**<p>FORMULA: C227H362N16O192S10X</p>**

**<p>CHARGE: 0</p>**

**</body>**

**</notes>**

**<annotation>**

**<rdf:RDF xmlns:rdf="http://www.w3.org/1999/02/22-rdf-syntax-ns#" xmlns:bqmodel="http://biomodels.net/model-qualifiers/" xmlns:bqbiol="http://biomodels.net/biology-qualifiers/">**

**<rdf:Description rdf:about="_63bf5b3f-efcf-460d-bfbf-a3f4bdc99f89"/></rdf:RDF>**

**</annotation>**

**</species>**

**<species id="M_CE5752" constant="false" hasOnlySubstanceUnits="false" name="iso-A2E(13-cis)" metaid="1a9f1e87-1131-443f-a82a-84d20395f2e5" boundaryCondition="false" compartment="metaComp">**

**<notes>**

**<body xmlns="http://www.w3.org/1999/xhtml">**

**<p>FORMULA: C42H58NO</p>**

**<p>CHARGE: 0</p>**

**<p>INCHI: InChI=1/C42H58NO/c1-32(20-22-39-35(4)17-12-25-41(39,6)7)14-10-16-34(3)30-38-31-37(24-27-43(38)28-29-44)19-11-15-33(2)21-23-40-36(5)18-13-26-42(40,8)9/h10-11,14-16,19-24,27,30-31,44H,12-13,17-18,25-26,28-29H2,1-9H3/q+1/b16-10+,19-11+,22-20+,23-21+,32-14+,33-15+,34-30-</p>**

**</body>**

**</notes>**

**<annotation>**

**<rdf:RDF xmlns:rdf="http://www.w3.org/1999/02/22-rdf-syntax-ns#" xmlns:bqmodel="http://biomodels.net/model-qualifiers/" xmlns:bqbiol="http://biomodels.net/biology-qualifiers/">**

**<rdf:Description rdf:about="_1a9f1e87-1131-443f-a82a-84d20395f2e5">**

**<bqbiol:is>**

**<rdf:Bag>**

**<rdf:li rdf:resource="http://identifiers.org/INCHI/InChI=1/C42H58NO/c1-32(20-22-39-35(4)17-12-25-41(39,6)7)14-10-16-34(3)30-38-31-37(24-27-43(38)28-29-44)19-11-15-33(2)21-23-40-36(5)18-13-26-42(40,8)9/h10-11,14-16,19-24,27,30-31,44H,12-13,17-18,25-26,28-29H2,1-9H3/q+1/b16-10+,19-11+,22-20+,23-21+,32-14+,33-15+,34-30-"/></rdf:Bag></bqbiol:is></rdf:Description></rdf:RDF>**

**</annotation>**

**</species>**

**<species id="M_ksi_pre35" constant="false" hasOnlySubstanceUnits="false" name="keratan sulfate I biosynthesis, precursor 35" metaid="ca7c5830-e435-40cf-8492-d302da79b305" boundaryCondition="false" compartment="metaComp">**

**<notes>**

**<body xmlns="http://www.w3.org/1999/xhtml">**

**<p>FORMULA: C241H384N17O205S11X</p>**

**<p>CHARGE: 0</p>**

**</body>**

**</notes>**

**<annotation>**

**<rdf:RDF xmlns:rdf="http://www.w3.org/1999/02/22-rdf-syntax-ns#" xmlns:bqmodel="http://biomodels.net/model-qualifiers/" xmlns:bqbiol="http://biomodels.net/biology-qualifiers/">**

**<rdf:Description rdf:about="ca7c5830-e435-40cf-8492-d302da79b305"/></rdf:RDF>**

**</annotation>**

**</species>**

**<species id="M_cs_a_b_e_pre1" constant="false" hasOnlySubstanceUnits="false" name="chondroitin sulfate A (GalNAc4S-GlcA), B (IdoA2S-GalNAc4S), and E (GalNAc4,6diS-GlcA), precursor 1" metaid="5abb603d-dc3b-465c-b236-bd59e1fbb9f2" boundaryCondition="false" compartment="metaComp">**

**<notes>**

**<body xmlns="http://www.w3.org/1999/xhtml">**

**<p>FORMULA: C31H47NO31S2X</p>**

**<p>CHARGE: 0</p>**

**</body>**

**</notes>**

**<annotation>**

**<rdf:RDF xmlns:rdf="http://www.w3.org/1999/02/22-rdf-syntax-ns#" xmlns:bqmodel="http://biomodels.net/model-qualifiers/" xmlns:bqbiol="http://biomodels.net/biology-qualifiers/">**

**<rdf:Description rdf:about="_5abb603d-dc3b-465c-b236-bd59e1fbb9f2"/></rdf:RDF>**

**</annotation>**

**</species>**

**<species id="M_ditp" constant="false" hasOnlySubstanceUnits="false" name="dITP(4-)" metaid="0498678f-98d4-4e0a-ae99-37f6790b35fc" boundaryCondition="false" compartment="metaComp">**

**<notes>**

**<body xmlns="http://www.w3.org/1999/xhtml">**

**<p>FORMULA: C10H11N4O13P3</p>**

**<p>CHARGE: 0</p>**

**<p>PUBCHEM.COMPOUND: 146302</p>**

**<p>KEGG.COMPOUND: C01345</p>**

**<p>HMDB: HMDB03537</p>**

**<p>CHEBI: CHEBI:28807 || CHEBI:61382</p>**

**<p>INCHI: InChI=1S/C10H15N4O13P3/c15-5-1-7(14-4-13-8-9(14)11-3-12-10(8)16)25-6(5)2-24-29(20,21)27-30(22,23)26-28(17,18)19/h3-7,15H,1-2H2,(H,20,21)(H,22,23)(H,11,12,16)(H2,17,18,19)/t5-,6+,7+/m0/s1 || InChI=1S/C10H15N4O13P3/c15-5-1-7(14-4-13-8-9(14)11-3-12-10(8)16)25-6(5)2-24-29(20,21)27-30(22,23)26-28(17,18)19/h3-7,15H,1-2H2,(H,20,21)(H,22,23)(H,11,12,16)(H2,17,18,19)/p-4/t5-,6+,7+/m0/s1</p>**

**</body>**

**</notes>**

**<annotation>**

**<rdf:RDF xmlns:rdf="http://www.w3.org/1999/02/22-rdf-syntax-ns#" xmlns:bqmodel="http://biomodels.net/model-qualifiers/" xmlns:bqbiol="http://biomodels.net/biology-qualifiers/">**

**<rdf:Description rdf:about="_0498678f-98d4-4e0a-ae99-37f6790b35fc">**

**<bqbiol:is>**

**<rdf:Bag>**

**<rdf:li rdf:resource="http://identifiers.org/PUBCHEM.COMPOUND/146302"/></rdf:Bag></bqbiol:is><bqbiol:is>**

**<rdf:Bag>**

**<rdf:li rdf:resource="http://identifiers.org/KEGG.COMPOUND/C01345"/></rdf:Bag></bqbiol:is><bqbiol:is>**

**<rdf:Bag>**

**<rdf:li rdf:resource="http://identifiers.org/HMDB/HMDB03537"/></rdf:Bag></bqbiol:is><bqbiol:is>**

**<rdf:Bag>**

**<rdf:li rdf:resource="http://identifiers.org/CHEBI/CHEBI:28807"/></rdf:Bag></bqbiol:is><bqbiol:is>**

**<rdf:Bag>**

**<rdf:li rdf:resource="http://identifiers.org/CHEBI/CHEBI:61382"/></rdf:Bag></bqbiol:is><bqbiol:is>**

**<rdf:Bag>**

**<rdf:li rdf:resource="http://identifiers.org/INCHI/InChI=1S/C10H15N4O13P3/c15-5-1-7(14-4-13-8-9(14)11-3-12-10(8)16)25-6(5)2-24-29(20,21)27-30(22,23)26-28(17,18)19/h3-7,15H,1-2H2,(H,20,21)(H,22,23)(H,11,12,16)(H2,17,18,19)/t5-,6+,7+/m0/s1"/></rdf:Bag></bqbiol:is><bqbiol:is>**

**<rdf:Bag>**

**<rdf:li rdf:resource="http://identifiers.org/INCHI/InChI=1S/C10H15N4O13P3/c15-5-1-7(14-4-13-8-9(14)11-3-12-10(8)16)25-6(5)2-24-29(20,21)27-30(22,23)26-28(17,18)19/h3-7,15H,1-2H2,(H,20,21)(H,22,23)(H,11,12,16)(H2,17,18,19)/p-4/t5-,6+,7+/m0/s1"/></rdf:Bag></bqbiol:is></rdf:Description></rdf:RDF>**

**</annotation>**

**</species>**

**<species id="M_CE5753" constant="false" hasOnlySubstanceUnits="false" name="iso-A2E(11-cis)" metaid="9320b6bf-306c-4547-bdb0-3227770ea24f" boundaryCondition="false" compartment="metaComp">**

**<notes>**

**<body xmlns="http://www.w3.org/1999/xhtml">**

**<p>FORMULA: C42H58NO</p>**

**<p>CHARGE: 0</p>**

**<p>INCHI: InChI=1/C42H58NO/c1-32(20-22-39-35(4)17-12-25-41(39,6)7)14-10-16-34(3)30-38-31-37(24-27-43(38)28-29-44)19-11-15-33(2)21-23-40-36(5)18-13-26-42(40,8)9/h10-11,14-16,19-24,27,30-31,44H,12-13,17-18,25-26,28-29H2,1-9H3/q+1/b16-10+,19-11-,22-20+,23-21+,32-14+,33-15+,34-30+</p>**

**</body>**

**</notes>**

**<annotation>**

**<rdf:RDF xmlns:rdf="http://www.w3.org/1999/02/22-rdf-syntax-ns#" xmlns:bqmodel="http://biomodels.net/model-qualifiers/" xmlns:bqbiol="http://biomodels.net/biology-qualifiers/">**

**<rdf:Description rdf:about="_9320b6bf-306c-4547-bdb0-3227770ea24f">**

**<bqbiol:is>**

**<rdf:Bag>**

**<rdf:li rdf:resource="http://identifiers.org/INCHI/InChI=1/C42H58NO/c1-32(20-22-39-35(4)17-12-25-41(39,6)7)14-10-16-34(3)30-38-31-37(24-27-43(38)28-29-44)19-11-15-33(2)21-23-40-36(5)18-13-26-42(40,8)9/h10-11,14-16,19-24,27,30-31,44H,12-13,17-18,25-26,28-29H2,1-9H3/q+1/b16-10+,19-11-,22-20+,23-21+,32-14+,33-15+,34-30+"/></rdf:Bag></bqbiol:is></rdf:Description></rdf:RDF>**

**</annotation>**

**</species>**

**<species id="M_ksi_pre34" constant="false" hasOnlySubstanceUnits="false" name="keratan sulfate I biosynthesis, precursor 34" metaid="23ac92ef-b931-4ce1-b9c1-fe63fc91258b" boundaryCondition="false" compartment="metaComp">**

**<notes>**

**<body xmlns="http://www.w3.org/1999/xhtml">**

**<p>FORMULA: C241H385N17O202S10X</p>**

**<p>CHARGE: 0</p>**

**</body>**

**</notes>**

**<annotation>**

**<rdf:RDF xmlns:rdf="http://www.w3.org/1999/02/22-rdf-syntax-ns#" xmlns:bqmodel="http://biomodels.net/model-qualifiers/" xmlns:bqbiol="http://biomodels.net/biology-qualifiers/">**

**<rdf:Description rdf:about="_23ac92ef-b931-4ce1-b9c1-fe63fc91258b"/></rdf:RDF>**

**</annotation>**

**</species>**

**<species id="M_CE5754" constant="false" hasOnlySubstanceUnits="false" name="iso-A2E(9-cis)" metaid="5c61d125-6e69-432b-b1c9-bbb87aa77cc3" boundaryCondition="false" compartment="metaComp">**

**<notes>**

**<body xmlns="http://www.w3.org/1999/xhtml">**

**<p>FORMULA: C42H58NO</p>**

**<p>CHARGE: 0</p>**

**<p>INCHI: InChI=1/C42H58NO/c1-32(20-22-39-35(4)17-12-25-41(39,6)7)14-10-16-34(3)30-38-31-37(24-27-43(38)28-29-44)19-11-15-33(2)21-23-40-36(5)18-13-26-42(40,8)9/h10-11,14-16,19-24,27,30-31,44H,12-13,17-18,25-26,28-29H2,1-9H3/q+1/b16-10+,19-11+,22-20+,23-21+,32-14+,33-15-,34-30+</p>**

**</body>**

**</notes>**

**<annotation>**

**<rdf:RDF xmlns:rdf="http://www.w3.org/1999/02/22-rdf-syntax-ns#" xmlns:bqmodel="http://biomodels.net/model-qualifiers/" xmlns:bqbiol="http://biomodels.net/biology-qualifiers/">**

**<rdf:Description rdf:about="_5c61d125-6e69-432b-b1c9-bbb87aa77cc3">**

**<bqbiol:is>**

**<rdf:Bag>**

**<rdf:li rdf:resource="http://identifiers.org/INCHI/InChI=1/C42H58NO/c1-32(20-22-39-35(4)17-12-25-41(39,6)7)14-10-16-34(3)30-38-31-37(24-27-43(38)28-29-44)19-11-15-33(2)21-23-40-36(5)18-13-26-42(40,8)9/h10-11,14-16,19-24,27,30-31,44H,12-13,17-18,25-26,28-29H2,1-9H3/q+1/b16-10+,19-11+,22-20+,23-21+,32-14+,33-15-,34-30+"/></rdf:Bag></bqbiol:is></rdf:Description></rdf:RDF>**

**</annotation>**

**</species>**

**<species id="M_CE5755" constant="false" hasOnlySubstanceUnits="false" name="iso-A2E(9,13-di-cis)" metaid="a9ee9e86-36cf-42b2-b69e-944fc9d63b8a" boundaryCondition="false" compartment="metaComp">**

**<notes>**

**<body xmlns="http://www.w3.org/1999/xhtml">**

**<p>FORMULA: C42H58NO</p>**

**<p>CHARGE: 0</p>**

**<p>INCHI: InChI=1/C42H58NO/c1-32(20-22-39-35(4)17-12-25-41(39,6)7)14-10-16-34(3)30-38-31-37(24-27-43(38)28-29-44)19-11-15-33(2)21-23-40-36(5)18-13-26-42(40,8)9/h10-11,14-16,19-24,27,30-31,44H,12-13,17-18,25-26,28-29H2,1-9H3/q+1/b16-10+,19-11+,22-20+,23-21+,32-14+,33-15-,34-30-</p>**

**</body>**

**</notes>**

**<annotation>**

**<rdf:RDF xmlns:rdf="http://www.w3.org/1999/02/22-rdf-syntax-ns#" xmlns:bqmodel="http://biomodels.net/model-qualifiers/" xmlns:bqbiol="http://biomodels.net/biology-qualifiers/">**

**<rdf:Description rdf:about="a9ee9e86-36cf-42b2-b69e-944fc9d63b8a">**

**<bqbiol:is>**

**<rdf:Bag>**

**<rdf:li rdf:resource="http://identifiers.org/INCHI/InChI=1/C42H58NO/c1-32(20-22-39-35(4)17-12-25-41(39,6)7)14-10-16-34(3)30-38-31-37(24-27-43(38)28-29-44)19-11-15-33(2)21-23-40-36(5)18-13-26-42(40,8)9/h10-11,14-16,19-24,27,30-31,44H,12-13,17-18,25-26,28-29H2,1-9H3/q+1/b16-10+,19-11+,22-20+,23-21+,32-14+,33-15-,34-30-"/></rdf:Bag></bqbiol:is></rdf:Description></rdf:RDF>**

**</annotation>**

**</species>**

**<species id="M_hs_pre15" constant="false" hasOnlySubstanceUnits="false" name="heparan sulfate, precursor 15" metaid="7c3cad24-2146-4ae1-bc4c-bb3d03e19338" boundaryCondition="false" compartment="metaComp">**

**<notes>**

**<body xmlns="http://www.w3.org/1999/xhtml">**

**<p>FORMULA: C79H114N5O98S11X</p>**

**<p>CHARGE: 0</p>**

**</body>**

**</notes>**

**<annotation>**

**<rdf:RDF xmlns:rdf="http://www.w3.org/1999/02/22-rdf-syntax-ns#" xmlns:bqmodel="http://biomodels.net/model-qualifiers/" xmlns:bqbiol="http://biomodels.net/biology-qualifiers/">**

**<rdf:Description rdf:about="_7c3cad24-2146-4ae1-bc4c-bb3d03e19338"/></rdf:RDF>**

**</annotation>**

**</species>**

**<species id="M_CE5756" constant="false" hasOnlySubstanceUnits="false" name="9-cis-retinoyl-beta-D-glucuronide" metaid="f4766929-9a02-4c0d-9207-7175939b1f2a" boundaryCondition="false" compartment="metaComp">**

**<notes>**

**<body xmlns="http://www.w3.org/1999/xhtml">**

**<p>FORMULA: C26H35O8</p>**

**<p>CHARGE: 0</p>**

**<p>PUBCHEM.COMPOUND: 5281877</p>**

**<p>KEGG.COMPOUND: C11061</p>**

**<p>HMDB: HMDB03141</p>**

**<p>CHEBI: CHEBI:28870</p>**

**<p>INCHI: InChI=1S/C26H36O8/c1-15(11-12-18-17(3)10-7-13-26(18,4)5)8-6-9-16(2)14-19(27)33-25-22(30)20(28)21(29)23(34-25)24(31)32/h6,8-9,11-12,14,20-23,25,28-30H,7,10,13H2,1-5H3,(H,31,32)/b9-6+,12-11+,15-8+,16-14+/t20-,21-,22+,23-,25+/m0/s1 || InChI=1/C26H36O8/c1-15(11-12-18-17(3)10-7-13-26(18,4)5)8-6-9-16(2)14-19(27)33-25-22(30)20(28)21(29)23(34-25)24(31)32/h6,8-9,11-12,14,20-23,25,28-30H,7,10,13H2,1-5H3,(H,31,32)/p-1/b9-6+,12-11+,15-8-,16-14+/t20-,21+,22-,23-,25?/m1/s1</p>**

**</body>**

**</notes>**

**<annotation>**

**<rdf:RDF xmlns:rdf="http://www.w3.org/1999/02/22-rdf-syntax-ns#" xmlns:bqmodel="http://biomodels.net/model-qualifiers/" xmlns:bqbiol="http://biomodels.net/biology-qualifiers/">**

**<rdf:Description rdf:about="f4766929-9a02-4c0d-9207-7175939b1f2a">**

**<bqbiol:is>**

**<rdf:Bag>**

**<rdf:li rdf:resource="http://identifiers.org/PUBCHEM.COMPOUND/5281877"/></rdf:Bag></bqbiol:is><bqbiol:is>**

**<rdf:Bag>**

**<rdf:li rdf:resource="http://identifiers.org/KEGG.COMPOUND/C11061"/></rdf:Bag></bqbiol:is><bqbiol:is>**

**<rdf:Bag>**

**<rdf:li rdf:resource="http://identifiers.org/HMDB/HMDB03141"/></rdf:Bag></bqbiol:is><bqbiol:is>**

**<rdf:Bag>**

**<rdf:li rdf:resource="http://identifiers.org/CHEBI/CHEBI:28870"/></rdf:Bag></bqbiol:is><bqbiol:is>**

**<rdf:Bag>**

**<rdf:li rdf:resource="http://identifiers.org/INCHI/InChI=1S/C26H36O8/c1-15(11-12-18-17(3)10-7-13-26(18,4)5)8-6-9-16(2)14-19(27)33-25-22(30)20(28)21(29)23(34-25)24(31)32/h6,8-9,11-12,14,20-23,25,28-30H,7,10,13H2,1-5H3,(H,31,32)/b9-6+,12-11+,15-8+,16-14+/t20-,21-,22+,23-,25+/m0/s1"/></rdf:Bag></bqbiol:is><bqbiol:is>**

**<rdf:Bag>**

**<rdf:li rdf:resource="http://identifiers.org/INCHI/InChI=1/C26H36O8/c1-15(11-12-18-17(3)10-7-13-26(18,4)5)8-6-9-16(2)14-19(27)33-25-22(30)20(28)21(29)23(34-25)24(31)32/h6,8-9,11-12,14,20-23,25,28-30H,7,10,13H2,1-5H3,(H,31,32)/p-1/b9-6+,12-11+,15-8-,16-14+/t20-,21+,22-,23-,25?/m1/s1"/></rdf:Bag></bqbiol:is></rdf:Description></rdf:RDF>**

**</annotation>**

**</species>**

**<species id="M_ksi_pre36" constant="false" hasOnlySubstanceUnits="false" name="keratan sulfate I biosynthesis, precursor 36" metaid="b5c6467b-3704-4852-9787-a2372ec1068a" boundaryCondition="false" compartment="metaComp">**

**<notes>**

**<body xmlns="http://www.w3.org/1999/xhtml">**

**<p>FORMULA: C247H394N17O210S11X</p>**

**<p>CHARGE: 0</p>**

**</body>**

**</notes>**

**<annotation>**

**<rdf:RDF xmlns:rdf="http://www.w3.org/1999/02/22-rdf-syntax-ns#" xmlns:bqmodel="http://biomodels.net/model-qualifiers/" xmlns:bqbiol="http://biomodels.net/biology-qualifiers/">**

**<rdf:Description rdf:about="b5c6467b-3704-4852-9787-a2372ec1068a"/></rdf:RDF>**

**</annotation>**

**</species>**

**<species id="M_hs_pre14" constant="false" hasOnlySubstanceUnits="false" name="heparan sulfate, precursor 14" metaid="a5f1d141-f465-41bf-a2d4-d3aac50fefc8" boundaryCondition="false" compartment="metaComp">**

**<notes>**

**<body xmlns="http://www.w3.org/1999/xhtml">**

**<p>FORMULA: C79H115N5O95S10X</p>**

**<p>CHARGE: 0</p>**

**</body>**

**</notes>**

**<annotation>**

**<rdf:RDF xmlns:rdf="http://www.w3.org/1999/02/22-rdf-syntax-ns#" xmlns:bqmodel="http://biomodels.net/model-qualifiers/" xmlns:bqbiol="http://biomodels.net/biology-qualifiers/">**

**<rdf:Description rdf:about="a5f1d141-f465-41bf-a2d4-d3aac50fefc8"/></rdf:RDF>**

**</annotation>**

**</species>**

**<species id="M_CE5757" constant="false" hasOnlySubstanceUnits="false" name="4-oxo-9-cis-retinoyl-beta-glucuronide" metaid="4dcc2e31-5464-4c8f-a880-cdbd864cf429" boundaryCondition="false" compartment="metaComp">**

**<notes>**

**<body xmlns="http://www.w3.org/1999/xhtml">**

**<p>FORMULA: C26H33O9</p>**

**<p>CHARGE: 0</p>**

**<p>INCHI: InChI=1/C26H34O9/c1-14(9-11-17-16(3)10-12-18(27)26(17,4)5)7-6-8-15(2)13-19(28)34-25-22(31)20(29)21(30)23(35-25)24(32)33/h6-9,11,13,20-23,25,29-31H,10,12H2,1-5H3,(H,32,33)/p-1/b8-6+,11-9+,14-7-,15-13+/t20-,21+,22-,23-,25?/m1/s1</p>**

**</body>**

**</notes>**

**<annotation>**

**<rdf:RDF xmlns:rdf="http://www.w3.org/1999/02/22-rdf-syntax-ns#" xmlns:bqmodel="http://biomodels.net/model-qualifiers/" xmlns:bqbiol="http://biomodels.net/biology-qualifiers/">**

**<rdf:Description rdf:about="_4dcc2e31-5464-4c8f-a880-cdbd864cf429">**

**<bqbiol:is>**

**<rdf:Bag>**

**<rdf:li rdf:resource="http://identifiers.org/INCHI/InChI=1/C26H34O9/c1-14(9-11-17-16(3)10-12-18(27)26(17,4)5)7-6-8-15(2)13-19(28)34-25-22(31)20(29)21(30)23(35-25)24(32)33/h6-9,11,13,20-23,25,29-31H,10,12H2,1-5H3,(H,32,33)/p-1/b8-6+,11-9+,14-7-,15-13+/t20-,21+,22-,23-,25?/m1/s1"/></rdf:Bag></bqbiol:is></rdf:Description></rdf:RDF>**

**</annotation>**

**</species>**

**<species id="M_akg" constant="false" hasOnlySubstanceUnits="false" name="2-oxoglutarate(2-)" metaid="4dbf89bb-b74d-4bf1-ae3b-34f9e64773ce" boundaryCondition="false" compartment="metaComp">**

**<notes>**

**<body xmlns="http://www.w3.org/1999/xhtml">**

**<p>FORMULA: C5H4O5</p>**

**<p>CHARGE: 0</p>**

**<p>PUBCHEM.COMPOUND: 51</p>**

**<p>KEGG.COMPOUND: C00026</p>**

**<p>HMDB: HMDB00208</p>**

**<p>CHEBI: CHEBI:30915 || CHEBI:16810</p>**

**<p>INCHI: InChI=1S/C5H6O5/c6-3(5(9)10)1-2-4(7)8/h1-2H2,(H,7,8)(H,9,10) || InChI=1S/C5H6O5/c6-3(5(9)10)1-2-4(7)8/h1-2H2,(H,7,8)(H,9,10)/p-2</p>**

**</body>**

**</notes>**

**<annotation>**

**<rdf:RDF xmlns:rdf="http://www.w3.org/1999/02/22-rdf-syntax-ns#" xmlns:bqmodel="http://biomodels.net/model-qualifiers/" xmlns:bqbiol="http://biomodels.net/biology-qualifiers/">**

**<rdf:Description rdf:about="_4dbf89bb-b74d-4bf1-ae3b-34f9e64773ce">**

**<bqbiol:is>**

**<rdf:Bag>**

**<rdf:li rdf:resource="http://identifiers.org/PUBCHEM.COMPOUND/51"/></rdf:Bag></bqbiol:is><bqbiol:is>**

**<rdf:Bag>**

**<rdf:li rdf:resource="http://identifiers.org/KEGG.COMPOUND/C00026"/></rdf:Bag></bqbiol:is><bqbiol:is>**

**<rdf:Bag>**

**<rdf:li rdf:resource="http://identifiers.org/HMDB/HMDB00208"/></rdf:Bag></bqbiol:is><bqbiol:is>**

**<rdf:Bag>**

**<rdf:li rdf:resource="http://identifiers.org/CHEBI/CHEBI:30915"/></rdf:Bag></bqbiol:is><bqbiol:is>**

**<rdf:Bag>**

**<rdf:li rdf:resource="http://identifiers.org/CHEBI/CHEBI:16810"/></rdf:Bag></bqbiol:is><bqbiol:is>**

**<rdf:Bag>**

**<rdf:li rdf:resource="http://identifiers.org/INCHI/InChI=1S/C5H6O5/c6-3(5(9)10)1-2-4(7)8/h1-2H2,(H,7,8)(H,9,10)"/></rdf:Bag></bqbiol:is><bqbiol:is>**

**<rdf:Bag>**

**<rdf:li rdf:resource="http://identifiers.org/INCHI/InChI=1S/C5H6O5/c6-3(5(9)10)1-2-4(7)8/h1-2H2,(H,7,8)(H,9,10)/p-2"/></rdf:Bag></bqbiol:is></rdf:Description></rdf:RDF>**

**</annotation>**

**</species>**

**<species id="M_ocdcea" constant="false" hasOnlySubstanceUnits="false" name="oleate" metaid="82d1c83e-49ce-473f-a53d-4b2e051f9f4a" boundaryCondition="false" compartment="metaComp">**

**<notes>**

**<body xmlns="http://www.w3.org/1999/xhtml">**

**<p>FORMULA: C18H33O2</p>**

**<p>CHARGE: 0</p>**

**<p>PUBCHEM.COMPOUND: 445639 || 5460221</p>**

**<p>INCHIKEY: ZQPPMHVWECSIRJ-KTKRTIGZSA-M</p>**

**<p>KEGG.COMPOUND: C00712</p>**

**<p>HMDB: HMDB00207</p>**

**<p>CHEBI: CHEBI:30823 || CHEBI:16196</p>**

**<p>INCHI: InChI=1S/C18H34O2/c1-2-3-4-5-6-7-8-9-10-11-12-13-14-15-16-17-18(19)20/h9-10H,2-8,11-17H2,1H3,(H,19,20)/p-1/b10-9- || InChI=1S/C18H34O2/c1-2-3-4-5-6-7-8-9-10-11-12-13-14-15-16-17-18(19)20/h9-10H,2-8,11-17H2,1H3,(H,19,20)/b10-9-</p>**

**</body>**

**</notes>**

**<annotation>**

**<rdf:RDF xmlns:rdf="http://www.w3.org/1999/02/22-rdf-syntax-ns#" xmlns:bqmodel="http://biomodels.net/model-qualifiers/" xmlns:bqbiol="http://biomodels.net/biology-qualifiers/">**

**<rdf:Description rdf:about="_82d1c83e-49ce-473f-a53d-4b2e051f9f4a">**

**<bqbiol:is>**

**<rdf:Bag>**

**<rdf:li rdf:resource="http://identifiers.org/PUBCHEM.COMPOUND/445639"/></rdf:Bag></bqbiol:is><bqbiol:is>**

**<rdf:Bag>**

**<rdf:li rdf:resource="http://identifiers.org/PUBCHEM.COMPOUND/5460221"/></rdf:Bag></bqbiol:is><bqbiol:is>**

**<rdf:Bag>**

**<rdf:li rdf:resource="http://identifiers.org/INCHIKEY/ZQPPMHVWECSIRJ-KTKRTIGZSA-M"/></rdf:Bag></bqbiol:is><bqbiol:is>**

**<rdf:Bag>**

**<rdf:li rdf:resource="http://identifiers.org/KEGG.COMPOUND/C00712"/></rdf:Bag></bqbiol:is><bqbiol:is>**

**<rdf:Bag>**

**<rdf:li rdf:resource="http://identifiers.org/HMDB/HMDB00207"/></rdf:Bag></bqbiol:is><bqbiol:is>**

**<rdf:Bag>**

**<rdf:li rdf:resource="http://identifiers.org/CHEBI/CHEBI:30823"/></rdf:Bag></bqbiol:is><bqbiol:is>**

**<rdf:Bag>**

**<rdf:li rdf:resource="http://identifiers.org/CHEBI/CHEBI:16196"/></rdf:Bag></bqbiol:is><bqbiol:is>**

**<rdf:Bag>**

**<rdf:li rdf:resource="http://identifiers.org/INCHI/InChI=1S/C18H34O2/c1-2-3-4-5-6-7-8-9-10-11-12-13-14-15-16-17-18(19)20/h9-10H,2-8,11-17H2,1H3,(H,19,20)/p-1/b10-9-"/></rdf:Bag></bqbiol:is><bqbiol:is>**

**<rdf:Bag>**

**<rdf:li rdf:resource="http://identifiers.org/INCHI/InChI=1S/C18H34O2/c1-2-3-4-5-6-7-8-9-10-11-12-13-14-15-16-17-18(19)20/h9-10H,2-8,11-17H2,1H3,(H,19,20)/b10-9-"/></rdf:Bag></bqbiol:is></rdf:Description></rdf:RDF>**

**</annotation>**

**</species>**

**<species id="M_C02592" constant="false" hasOnlySubstanceUnits="false" name="Taurolithocholate" metaid="77b5553e-7851-4e5b-91f2-56d4fd06e200" boundaryCondition="false" compartment="metaComp">**

**<notes>**

**<body xmlns="http://www.w3.org/1999/xhtml">**

**<p>FORMULA: C26H44NO5S</p>**

**<p>CHARGE: 0</p>**

**<p>PUBCHEM.COMPOUND: 53477716 || 439763</p>**

**<p>INCHIKEY: QBYUNVOYXHFVKC-GBURMNQMSA-N</p>**

**<p>KEGG.COMPOUND: C02592</p>**

**<p>HMDB: HMDB00722</p>**

**<p>CHEBI: CHEBI:36259 || CHEBI:17179</p>**

**<p>INCHI: InChI=1S/C26H45NO5S/c1-17(4-9-24(29)27-14-15-33(30,31)32)21-7-8-22-20-6-5-18-16-19(28)10-12-25(18,2)23(20)11-13-26(21,22)3/h17-23,28H,4-16H2,1-3H3,(H,27,29)(H,30,31,32)/p-1/t17-,18-,19-,20+,21-,22+,23+,25+,26-/m1/s1 || InChI=1S/C26H45NO5S/c1-17(4-9-24(29)27-14-15-33(30,31)32)21-7-8-22-20-6-5-18-16-19(28)10-12-25(18,2)23(20)11-13-26(21,22)3/h17-23,28H,4-16H2,1-3H3,(H,27,29)(H,30,31,32)/t17-,18-,19-,20+,21-,22+,23+,25+,26-/m1/s1 || InChI=1S/C26H45NO5S/c1-17(4-9-24(29)27-14-15-33(30,31)32)21-7-8-22-20-6-5-18-16-19(28)10-12-25(18,2)23(20)11-13-26(21,22)3/h17-23,28H,4-16H2,1-3H3,(H,27,29)(H,30,31,32)/t17-,18?,19-,20?,21?,22?,23?,25+,26-/m1/s1</p>**

**</body>**

**</notes>**

**<annotation>**

**<rdf:RDF xmlns:rdf="http://www.w3.org/1999/02/22-rdf-syntax-ns#" xmlns:bqmodel="http://biomodels.net/model-qualifiers/" xmlns:bqbiol="http://biomodels.net/biology-qualifiers/">**

**<rdf:Description rdf:about="_77b5553e-7851-4e5b-91f2-56d4fd06e200">**

**<bqbiol:is>**

**<rdf:Bag>**

**<rdf:li rdf:resource="http://identifiers.org/PUBCHEM.COMPOUND/53477716"/></rdf:Bag></bqbiol:is><bqbiol:is>**

**<rdf:Bag>**

**<rdf:li rdf:resource="http://identifiers.org/PUBCHEM.COMPOUND/439763"/></rdf:Bag></bqbiol:is><bqbiol:is>**

**<rdf:Bag>**

**<rdf:li rdf:resource="http://identifiers.org/INCHIKEY/QBYUNVOYXHFVKC-GBURMNQMSA-N"/></rdf:Bag></bqbiol:is><bqbiol:is>**

**<rdf:Bag>**

**<rdf:li rdf:resource="http://identifiers.org/KEGG.COMPOUND/C02592"/></rdf:Bag></bqbiol:is><bqbiol:is>**

**<rdf:Bag>**

**<rdf:li rdf:resource="http://identifiers.org/HMDB/HMDB00722"/></rdf:Bag></bqbiol:is><bqbiol:is>**

**<rdf:Bag>**

**<rdf:li rdf:resource="http://identifiers.org/CHEBI/CHEBI:36259"/></rdf:Bag></bqbiol:is><bqbiol:is>**

**<rdf:Bag>**

**<rdf:li rdf:resource="http://identifiers.org/CHEBI/CHEBI:17179"/></rdf:Bag></bqbiol:is><bqbiol:is>**

**<rdf:Bag>**

**<rdf:li rdf:resource="http://identifiers.org/INCHI/InChI=1S/C26H45NO5S/c1-17(4-9-24(29)27-14-15-33(30,31)32)21-7-8-22-20-6-5-18-16-19(28)10-12-25(18,2)23(20)11-13-26(21,22)3/h17-23,28H,4-16H2,1-3H3,(H,27,29)(H,30,31,32)/p-1/t17-,18-,19-,20+,21-,22+,23+,25+,26-/m1/s1"/></rdf:Bag></bqbiol:is><bqbiol:is>**

**<rdf:Bag>**

**<rdf:li rdf:resource="http://identifiers.org/INCHI/InChI=1S/C26H45NO5S/c1-17(4-9-24(29)27-14-15-33(30,31)32)21-7-8-22-20-6-5-18-16-19(28)10-12-25(18,2)23(20)11-13-26(21,22)3/h17-23,28H,4-16H2,1-3H3,(H,27,29)(H,30,31,32)/t17-,18-,19-,20+,21-,22+,23+,25+,26-/m1/s1"/></rdf:Bag></bqbiol:is><bqbiol:is>**

**<rdf:Bag>**

**<rdf:li rdf:resource="http://identifiers.org/INCHI/InChI=1S/C26H45NO5S/c1-17(4-9-24(29)27-14-15-33(30,31)32)21-7-8-22-20-6-5-18-16-19(28)10-12-25(18,2)23(20)11-13-26(21,22)3/h17-23,28H,4-16H2,1-3H3,(H,27,29)(H,30,31,32)/t17-,18?,19-,20?,21?,22?,23?,25+,26-/m1/s1"/></rdf:Bag></bqbiol:is></rdf:Description></rdf:RDF>**

**</annotation>**

**</species>**

**<species id="M_tmtrdcoa" constant="false" hasOnlySubstanceUnits="false" name="4,8,12-trimethyl tridecanoylcoa" metaid="3be60c00-74eb-493c-b55f-ff5fdf0c7627" boundaryCondition="false" compartment="metaComp">**

**<notes>**

**<body xmlns="http://www.w3.org/1999/xhtml">**

**<p>FORMULA: C37H62N7O17P3S</p>**

**<p>CHARGE: 0</p>**

**</body>**

**</notes>**

**<annotation>**

**<rdf:RDF xmlns:rdf="http://www.w3.org/1999/02/22-rdf-syntax-ns#" xmlns:bqmodel="http://biomodels.net/model-qualifiers/" xmlns:bqbiol="http://biomodels.net/biology-qualifiers/">**

**<rdf:Description rdf:about="_3be60c00-74eb-493c-b55f-ff5fdf0c7627"/></rdf:RDF>**

**</annotation>**

**</species>**

**<species id="M_omhdecacid" constant="false" hasOnlySubstanceUnits="false" name="w-hydroxydecanoicacid" metaid="7cdca8f3-e3be-45bb-88a1-5ba8b2ca8a89" boundaryCondition="false" compartment="metaComp">**

**<notes>**

**<body xmlns="http://www.w3.org/1999/xhtml">**

**<p>FORMULA: C10H19O3</p>**

**<p>CHARGE: 0</p>**

**</body>**

**</notes>**

**<annotation>**

**<rdf:RDF xmlns:rdf="http://www.w3.org/1999/02/22-rdf-syntax-ns#" xmlns:bqmodel="http://biomodels.net/model-qualifiers/" xmlns:bqbiol="http://biomodels.net/biology-qualifiers/">**

**<rdf:Description rdf:about="_7cdca8f3-e3be-45bb-88a1-5ba8b2ca8a89"/></rdf:RDF>**

**</annotation>**

**</species>**

**<species id="M_3deccrn" constant="false" hasOnlySubstanceUnits="false" name="3-hydroxydecanoylcarnitine" metaid="b8431cbc-6897-4935-9360-05fef90560ce" boundaryCondition="false" compartment="metaComp">**

**<notes>**

**<body xmlns="http://www.w3.org/1999/xhtml">**

**<p>FORMULA: C17H33NO5</p>**

**<p>CHARGE: 0</p>**

**</body>**

**</notes>**

**<annotation>**

**<rdf:RDF xmlns:rdf="http://www.w3.org/1999/02/22-rdf-syntax-ns#" xmlns:bqmodel="http://biomodels.net/model-qualifiers/" xmlns:bqbiol="http://biomodels.net/biology-qualifiers/">**

**<rdf:Description rdf:about="b8431cbc-6897-4935-9360-05fef90560ce"/></rdf:RDF>**

**</annotation>**

**</species>**

**<species id="M_mag_hs" constant="false" hasOnlySubstanceUnits="false" name="monoacylglycerol 2" metaid="934c86c6-7929-4322-96ca-0adcbbbbc32d" boundaryCondition="false" compartment="metaComp">**

**<notes>**

**<body xmlns="http://www.w3.org/1999/xhtml">**

**<p>FORMULA: C3H7O2FULLR2CO2</p>**

**<p>CHARGE: 0</p>**

**</body>**

**</notes>**

**<annotation>**

**<rdf:RDF xmlns:rdf="http://www.w3.org/1999/02/22-rdf-syntax-ns#" xmlns:bqmodel="http://biomodels.net/model-qualifiers/" xmlns:bqbiol="http://biomodels.net/biology-qualifiers/">**

**<rdf:Description rdf:about="_934c86c6-7929-4322-96ca-0adcbbbbc32d"/></rdf:RDF>**

**</annotation>**

**</species>**

**<species id="M_3thexddcoacrn" constant="false" hasOnlySubstanceUnits="false" name="3-hydroxy trans7,10-hexadecadienoyl carnitine" metaid="cdce4f7c-ac56-4f84-b573-6c47abd2cc47" boundaryCondition="false" compartment="metaComp">**

**<notes>**

**<body xmlns="http://www.w3.org/1999/xhtml">**

**<p>FORMULA: C23H41NO5</p>**

**<p>CHARGE: 0</p>**

**</body>**

**</notes>**

**<annotation>**

**<rdf:RDF xmlns:rdf="http://www.w3.org/1999/02/22-rdf-syntax-ns#" xmlns:bqmodel="http://biomodels.net/model-qualifiers/" xmlns:bqbiol="http://biomodels.net/biology-qualifiers/">**

**<rdf:Description rdf:about="cdce4f7c-ac56-4f84-b573-6c47abd2cc47"/></rdf:RDF>**

**</annotation>**

**</species>**

**<species id="M_5hoxnfkyn" constant="false" hasOnlySubstanceUnits="false" name="5-hydroxy-N-formylkynurenine" metaid="c49d9c2e-dace-4c70-ab4d-6035f619bd91" boundaryCondition="false" compartment="metaComp">**

**<notes>**

**<body xmlns="http://www.w3.org/1999/xhtml">**

**<p>FORMULA: C11H12N2O5</p>**

**<p>CHARGE: 0</p>**

**<p>PUBCHEM.COMPOUND: 440744 || 9548588</p>**

**<p>INCHIKEY: LSTOUSIIVKMJBU-QMMMGPOBSA-N</p>**

**<p>KEGG.COMPOUND: C05648</p>**

**<p>HMDB: HMDB04086</p>**

**<p>CHEBI: CHEBI:2065 || CHEBI:36407</p>**

**<p>INCHI: InChI=1S/C11H12N2O5/c12-8(11(17)18)4-10(16)7-3-6(15)1-2-9(7)13-5-14/h1-3,5,8,15H,4,12H2,(H,13,14)(H,17,18) || InChI=1S/C11H12N2O5/c12-8(11(17)18)4-10(16)7-3-6(15)1-2-9(7)13-5-14/h1-3,5,8,15H,4,12H2,(H,13,14)(H,17,18)/t8-/m0/s1</p>**

**</body>**

**</notes>**

**<annotation>**

**<rdf:RDF xmlns:rdf="http://www.w3.org/1999/02/22-rdf-syntax-ns#" xmlns:bqmodel="http://biomodels.net/model-qualifiers/" xmlns:bqbiol="http://biomodels.net/biology-qualifiers/">**

**<rdf:Description rdf:about="c49d9c2e-dace-4c70-ab4d-6035f619bd91">**

**<bqbiol:is>**

**<rdf:Bag>**

**<rdf:li rdf:resource="http://identifiers.org/PUBCHEM.COMPOUND/440744"/></rdf:Bag></bqbiol:is><bqbiol:is>**

**<rdf:Bag>**

**<rdf:li rdf:resource="http://identifiers.org/PUBCHEM.COMPOUND/9548588"/></rdf:Bag></bqbiol:is><bqbiol:is>**

**<rdf:Bag>**

**<rdf:li rdf:resource="http://identifiers.org/INCHIKEY/LSTOUSIIVKMJBU-QMMMGPOBSA-N"/></rdf:Bag></bqbiol:is><bqbiol:is>**

**<rdf:Bag>**

**<rdf:li rdf:resource="http://identifiers.org/KEGG.COMPOUND/C05648"/></rdf:Bag></bqbiol:is><bqbiol:is>**

**<rdf:Bag>**

**<rdf:li rdf:resource="http://identifiers.org/HMDB/HMDB04086"/></rdf:Bag></bqbiol:is><bqbiol:is>**

**<rdf:Bag>**

**<rdf:li rdf:resource="http://identifiers.org/CHEBI/CHEBI:2065"/></rdf:Bag></bqbiol:is><bqbiol:is>**

**<rdf:Bag>**

**<rdf:li rdf:resource="http://identifiers.org/CHEBI/CHEBI:36407"/></rdf:Bag></bqbiol:is><bqbiol:is>**

**<rdf:Bag>**

**<rdf:li rdf:resource="http://identifiers.org/INCHI/InChI=1S/C11H12N2O5/c12-8(11(17)18)4-10(16)7-3-6(15)1-2-9(7)13-5-14/h1-3,5,8,15H,4,12H2,(H,13,14)(H,17,18)"/></rdf:Bag></bqbiol:is><bqbiol:is>**

**<rdf:Bag>**

**<rdf:li rdf:resource="http://identifiers.org/INCHI/InChI=1S/C11H12N2O5/c12-8(11(17)18)4-10(16)7-3-6(15)1-2-9(7)13-5-14/h1-3,5,8,15H,4,12H2,(H,13,14)(H,17,18)/t8-/m0/s1"/></rdf:Bag></bqbiol:is></rdf:Description></rdf:RDF>**

**</annotation>**

**</species>**

**<species id="M_gd1b2_hs" constant="false" hasOnlySubstanceUnits="false" name="GD1beta" metaid="bb83f892-486c-4b7d-99be-40ab4bef5006" boundaryCondition="false" compartment="metaComp">**

**<notes>**

**<body xmlns="http://www.w3.org/1999/xhtml">**

**<p>FORMULA: C66H111N4O38FULLRCO</p>**

**<p>CHARGE: 0</p>**

**</body>**

**</notes>**

**<annotation>**

**<rdf:RDF xmlns:rdf="http://www.w3.org/1999/02/22-rdf-syntax-ns#" xmlns:bqmodel="http://biomodels.net/model-qualifiers/" xmlns:bqbiol="http://biomodels.net/biology-qualifiers/">**

**<rdf:Description rdf:about="bb83f892-486c-4b7d-99be-40ab4bef5006"/></rdf:RDF>**

**</annotation>**

**</species>**

**<species id="M_CE0713" constant="false" hasOnlySubstanceUnits="false" name="3-oxolinoleoyl-CoA" metaid="e97ed176-2fe8-43ca-b410-5274e691c392" boundaryCondition="false" compartment="metaComp">**

**<notes>**

**<body xmlns="http://www.w3.org/1999/xhtml">**

**<p>FORMULA: C39H60N7O18P3S</p>**

**<p>CHARGE: 0</p>**

**</body>**

**</notes>**

**<annotation>**

**<rdf:RDF xmlns:rdf="http://www.w3.org/1999/02/22-rdf-syntax-ns#" xmlns:bqmodel="http://biomodels.net/model-qualifiers/" xmlns:bqbiol="http://biomodels.net/biology-qualifiers/">**

**<rdf:Description rdf:about="e97ed176-2fe8-43ca-b410-5274e691c392"/></rdf:RDF>**

**</annotation>**

**</species>**

**<species id="M_CE5789" constant="false" hasOnlySubstanceUnits="false" name="kinetensin 1-8" metaid="4028ad98-2c6c-4a46-9b38-d5bf07e27cb8" boundaryCondition="false" compartment="metaComp">**

**<notes>**

**<body xmlns="http://www.w3.org/1999/xhtml">**

**<p>FORMULA: C50H76N16O10</p>**

**<p>CHARGE: 0</p>**

**<p>PUBCHEM.COMPOUND: 53481566</p>**

**<p>INCHIKEY: KLNGALQMFAURPH-DCLZXSHISA-N</p>**

**<p>HMDB: HMDB12985</p>**

**<p>INCHI: InChI=1S/C50H74N16O10/c1-4-28(2)40(51)46(73)60-29(3)41(68)61-34(13-8-20-57-49(52)53)42(69)62-35(14-9-21-58-50(54)55)43(70)64-37(25-32-26-56-27-59-32)47(74)66-22-10-15-39(66)45(72)63-36(23-31-16-18-33(67)19-17-31)44(71)65-38(48(75)76)24-30-11-6-5-7-12-30/h5-7,11-12,16-19,26-29,34-40,67H,4,8-10,13-15,20-25,51H2,1-3H3,(H,56,59)(H,60,73)(H,61,68)(H,62,69)(H,63,72)(H,64,70)(H,65,71)(H,75,76)(H4,52,53,57)(H4,54,55,58)/t28-,29+,34-,35+,36+,37-,38-,39+,40-/m1/s1</p>**

**</body>**

**</notes>**

**<annotation>**

**<rdf:RDF xmlns:rdf="http://www.w3.org/1999/02/22-rdf-syntax-ns#" xmlns:bqmodel="http://biomodels.net/model-qualifiers/" xmlns:bqbiol="http://biomodels.net/biology-qualifiers/">**

**<rdf:Description rdf:about="_4028ad98-2c6c-4a46-9b38-d5bf07e27cb8">**

**<bqbiol:is>**

**<rdf:Bag>**

**<rdf:li rdf:resource="http://identifiers.org/PUBCHEM.COMPOUND/53481566"/></rdf:Bag></bqbiol:is><bqbiol:is>**

**<rdf:Bag>**

**<rdf:li rdf:resource="http://identifiers.org/INCHIKEY/KLNGALQMFAURPH-DCLZXSHISA-N"/></rdf:Bag></bqbiol:is><bqbiol:is>**

**<rdf:Bag>**

**<rdf:li rdf:resource="http://identifiers.org/HMDB/HMDB12985"/></rdf:Bag></bqbiol:is><bqbiol:is>**

**<rdf:Bag>**

**<rdf:li rdf:resource="http://identifiers.org/INCHI/InChI=1S/C50H74N16O10/c1-4-28(2)40(51)46(73)60-29(3)41(68)61-34(13-8-20-57-49(52)53)42(69)62-35(14-9-21-58-50(54)55)43(70)64-37(25-32-26-56-27-59-32)47(74)66-22-10-15-39(66)45(72)63-36(23-31-16-18-33(67)19-17-31)44(71)65-38(48(75)76)24-30-11-6-5-7-12-30/h5-7,11-12,16-19,26-29,34-40,67H,4,8-10,13-15,20-25,51H2,1-3H3,(H,56,59)(H,60,73)(H,61,68)(H,62,69)(H,63,72)(H,64,70)(H,65,71)(H,75,76)(H4,52,53,57)(H4,54,55,58)/t28-,29+,34-,35+,36+,37-,38-,39+,40-/m1/s1"/></rdf:Bag></bqbiol:is></rdf:Description></rdf:RDF>**

**</annotation>**

**</species>**

**<species id="M_c10crn" constant="false" hasOnlySubstanceUnits="false" name="decanoyl carnitine" metaid="8c6ef33d-b8cd-458a-88e8-4086b5eb81ee" boundaryCondition="false" compartment="metaComp">**

**<notes>**

**<body xmlns="http://www.w3.org/1999/xhtml">**

**<p>FORMULA: C17H33NO4</p>**

**<p>CHARGE: 0</p>**

**<p>PUBCHEM.COMPOUND: 10245190</p>**

**<p>HMDB: HMDB00651</p>**

**<p>CHEBI: CHEBI:68830</p>**

**<p>INCHI: InChI=1S/C17H33NO4/c1-5-6-7-8-9-10-11-12-17(21)22-15(13-16(19)20)14-18(2,3)4/h15H,5-14H2,1-4H3</p>**

**</body>**

**</notes>**

**<annotation>**

**<rdf:RDF xmlns:rdf="http://www.w3.org/1999/02/22-rdf-syntax-ns#" xmlns:bqmodel="http://biomodels.net/model-qualifiers/" xmlns:bqbiol="http://biomodels.net/biology-qualifiers/">**

**<rdf:Description rdf:about="_8c6ef33d-b8cd-458a-88e8-4086b5eb81ee">**

**<bqbiol:is>**

**<rdf:Bag>**

**<rdf:li rdf:resource="http://identifiers.org/PUBCHEM.COMPOUND/10245190"/></rdf:Bag></bqbiol:is><bqbiol:is>**

**<rdf:Bag>**

**<rdf:li rdf:resource="http://identifiers.org/HMDB/HMDB00651"/></rdf:Bag></bqbiol:is><bqbiol:is>**

**<rdf:Bag>**

**<rdf:li rdf:resource="http://identifiers.org/CHEBI/CHEBI:68830"/></rdf:Bag></bqbiol:is><bqbiol:is>**

**<rdf:Bag>**

**<rdf:li rdf:resource="http://identifiers.org/INCHI/InChI=1S/C17H33NO4/c1-5-6-7-8-9-10-11-12-17(21)22-15(13-16(19)20)14-18(2,3)4/h15H,5-14H2,1-4H3"/></rdf:Bag></bqbiol:is></rdf:Description></rdf:RDF>**

**</annotation>**

**</species>**

**<species id="M_CE5788" constant="false" hasOnlySubstanceUnits="false" name="kinetensin 1-7" metaid="d5ffdc7d-b2bd-406b-b7a1-b93e4673a3f1" boundaryCondition="false" compartment="metaComp">**

**<notes>**

**<body xmlns="http://www.w3.org/1999/xhtml">**

**<p>FORMULA: C41H67N15O9</p>**

**<p>CHARGE: 0</p>**

**<p>PUBCHEM.COMPOUND: 53481565</p>**

**<p>INCHIKEY: QMQIVQMAYCBWNH-BKRGGBFWSA-N</p>**

**<p>HMDB: HMDB12984</p>**

**<p>INCHI: InChI=1S/C41H65N15O9/c1-4-22(2)32(42)37(62)51-23(3)33(58)52-27(8-5-15-48-40(43)44)34(59)53-28(9-6-16-49-41(45)46)35(60)54-29(19-25-20-47-21-50-25)38(63)56-17-7-10-31(56)36(61)55-30(39(64)65)18-24-11-13-26(57)14-12-24/h11-14,20-23,27-32,57H,4-10,15-19,42H2,1-3H3,(H,47,50)(H,51,62)(H,52,58)(H,53,59)(H,54,60)(H,55,61)(H,64,65)(H4,43,44,48)(H4,45,46,49)/t22-,23+,27-,28+,29+,30-,31-,32-/m1/s1</p>**

**</body>**

**</notes>**

**<annotation>**

**<rdf:RDF xmlns:rdf="http://www.w3.org/1999/02/22-rdf-syntax-ns#" xmlns:bqmodel="http://biomodels.net/model-qualifiers/" xmlns:bqbiol="http://biomodels.net/biology-qualifiers/">**

**<rdf:Description rdf:about="d5ffdc7d-b2bd-406b-b7a1-b93e4673a3f1">**

**<bqbiol:is>**

**<rdf:Bag>**

**<rdf:li rdf:resource="http://identifiers.org/PUBCHEM.COMPOUND/53481565"/></rdf:Bag></bqbiol:is><bqbiol:is>**

**<rdf:Bag>**

**<rdf:li rdf:resource="http://identifiers.org/INCHIKEY/QMQIVQMAYCBWNH-BKRGGBFWSA-N"/></rdf:Bag></bqbiol:is><bqbiol:is>**

**<rdf:Bag>**

**<rdf:li rdf:resource="http://identifiers.org/HMDB/HMDB12984"/></rdf:Bag></bqbiol:is><bqbiol:is>**

**<rdf:Bag>**

**<rdf:li rdf:resource="http://identifiers.org/INCHI/InChI=1S/C41H65N15O9/c1-4-22(2)32(42)37(62)51-23(3)33(58)52-27(8-5-15-48-40(43)44)34(59)53-28(9-6-16-49-41(45)46)35(60)54-29(19-25-20-47-21-50-25)38(63)56-17-7-10-31(56)36(61)55-30(39(64)65)18-24-11-13-26(57)14-12-24/h11-14,20-23,27-32,57H,4-10,15-19,42H2,1-3H3,(H,47,50)(H,51,62)(H,52,58)(H,53,59)(H,54,60)(H,55,61)(H,64,65)(H4,43,44,48)(H4,45,46,49)/t22-,23+,27-,28+,29+,30-,31-,32-/m1/s1"/></rdf:Bag></bqbiol:is></rdf:Description></rdf:RDF>**

**</annotation>**

**</species>**

**<species id="M_CE5787" constant="false" hasOnlySubstanceUnits="false" name="kinetensin 1-3" metaid="727c7470-b159-4840-8da3-105e3fbd3b00" boundaryCondition="false" compartment="metaComp">**

**<notes>**

**<body xmlns="http://www.w3.org/1999/xhtml">**

**<p>FORMULA: C15H31N6O4</p>**

**<p>CHARGE: 0</p>**

**<p>PUBCHEM.COMPOUND: 53481564</p>**

**<p>INCHIKEY: JXUGDUWBMKIJDC-MMWGEVLESA-N</p>**

**<p>HMDB: HMDB12983</p>**

**<p>INCHI: InChI=1S/C15H30N6O4/c1-4-8(2)11(16)13(23)20-9(3)12(22)21-10(14(24)25)6-5-7-19-15(17)18/h8-11H,4-7,16H2,1-3H3,(H,20,23)(H,21,22)(H,24,25)(H4,17,18,19)/t8-,9-,10+,11-/m0/s1</p>**

**</body>**

**</notes>**

**<annotation>**

**<rdf:RDF xmlns:rdf="http://www.w3.org/1999/02/22-rdf-syntax-ns#" xmlns:bqmodel="http://biomodels.net/model-qualifiers/" xmlns:bqbiol="http://biomodels.net/biology-qualifiers/">**

**<rdf:Description rdf:about="_727c7470-b159-4840-8da3-105e3fbd3b00">**

**<bqbiol:is>**

**<rdf:Bag>**

**<rdf:li rdf:resource="http://identifiers.org/PUBCHEM.COMPOUND/53481564"/></rdf:Bag></bqbiol:is><bqbiol:is>**

**<rdf:Bag>**

**<rdf:li rdf:resource="http://identifiers.org/INCHIKEY/JXUGDUWBMKIJDC-MMWGEVLESA-N"/></rdf:Bag></bqbiol:is><bqbiol:is>**

**<rdf:Bag>**

**<rdf:li rdf:resource="http://identifiers.org/HMDB/HMDB12983"/></rdf:Bag></bqbiol:is><bqbiol:is>**

**<rdf:Bag>**

**<rdf:li rdf:resource="http://identifiers.org/INCHI/InChI=1S/C15H30N6O4/c1-4-8(2)11(16)13(23)20-9(3)12(22)21-10(14(24)25)6-5-7-19-15(17)18/h8-11H,4-7,16H2,1-3H3,(H,20,23)(H,21,22)(H,24,25)(H4,17,18,19)/t8-,9-,10+,11-/m0/s1"/></rdf:Bag></bqbiol:is></rdf:Description></rdf:RDF>**

**</annotation>**

**</species>**

**<species id="M_CE5786" constant="false" hasOnlySubstanceUnits="false" name="kinetensin" metaid="3f942d97-b969-4d44-943a-ec7173bd0776" boundaryCondition="false" compartment="metaComp">**

**<notes>**

**<body xmlns="http://www.w3.org/1999/xhtml">**

**<p>FORMULA: C56H87N17O11</p>**

**<p>CHARGE: 0</p>**

**<p>PUBCHEM.COMPOUND: 147043 || 53481569</p>**

**<p>INCHIKEY: PANUJGMSOSQAAY-HAGIGRARSA-N</p>**

**<p>HMDB: HMDB12988</p>**

**<p>INCHI: InChI=1S/C56H85N17O11/c1-6-32(4)45(57)52(81)66-33(5)46(75)67-38(15-10-22-63-55(58)59)47(76)68-39(16-11-23-64-56(60)61)48(77)71-42(28-36-29-62-30-65-36)53(82)73-24-12-17-44(73)51(80)70-41(27-35-18-20-37(74)21-19-35)49(78)69-40(26-34-13-8-7-9-14-34)50(79)72-43(54(83)84)25-31(2)3/h7-9,13-14,18-21,29-33,38-45,74H,6,10-12,15-17,22-28,57H2,1-5H3,(H,62,65)(H,66,81)(H,67,75)(H,68,76)(H,69,78)(H,70,80)(H,71,77)(H,72,79)(H,83,84)(H4,58,59,63)(H4,60,61,64)/t32-,33+,38-,39+,40+,41-,42-,43+,44-,45-/m0/s1</p>**

**</body>**

**</notes>**

**<annotation>**

**<rdf:RDF xmlns:rdf="http://www.w3.org/1999/02/22-rdf-syntax-ns#" xmlns:bqmodel="http://biomodels.net/model-qualifiers/" xmlns:bqbiol="http://biomodels.net/biology-qualifiers/">**

**<rdf:Description rdf:about="_3f942d97-b969-4d44-943a-ec7173bd0776">**

**<bqbiol:is>**

**<rdf:Bag>**

**<rdf:li rdf:resource="http://identifiers.org/PUBCHEM.COMPOUND/147043"/></rdf:Bag></bqbiol:is><bqbiol:is>**

**<rdf:Bag>**

**<rdf:li rdf:resource="http://identifiers.org/PUBCHEM.COMPOUND/53481569"/></rdf:Bag></bqbiol:is><bqbiol:is>**

**<rdf:Bag>**

**<rdf:li rdf:resource="http://identifiers.org/INCHIKEY/PANUJGMSOSQAAY-HAGIGRARSA-N"/></rdf:Bag></bqbiol:is><bqbiol:is>**

**<rdf:Bag>**

**<rdf:li rdf:resource="http://identifiers.org/HMDB/HMDB12988"/></rdf:Bag></bqbiol:is><bqbiol:is>**

**<rdf:Bag>**

**<rdf:li rdf:resource="http://identifiers.org/INCHI/InChI=1S/C56H85N17O11/c1-6-32(4)45(57)52(81)66-33(5)46(75)67-38(15-10-22-63-55(58)59)47(76)68-39(16-11-23-64-56(60)61)48(77)71-42(28-36-29-62-30-65-36)53(82)73-24-12-17-44(73)51(80)70-41(27-35-18-20-37(74)21-19-35)49(78)69-40(26-34-13-8-7-9-14-34)50(79)72-43(54(83)84)25-31(2)3/h7-9,13-14,18-21,29-33,38-45,74H,6,10-12,15-17,22-28,57H2,1-5H3,(H,62,65)(H,66,81)(H,67,75)(H,68,76)(H,69,78)(H,70,80)(H,71,77)(H,72,79)(H,83,84)(H4,58,59,63)(H4,60,61,64)/t32-,33+,38-,39+,40+,41-,42-,43+,44-,45-/m0/s1"/></rdf:Bag></bqbiol:is></rdf:Description></rdf:RDF>**

**</annotation>**

**</species>**

**<species id="M_tdchola" constant="false" hasOnlySubstanceUnits="false" name="taurochenodeoxycholate" metaid="f7adbcce-2277-4462-8747-ee7ce6b5b613" boundaryCondition="false" compartment="metaComp">**

**<notes>**

**<body xmlns="http://www.w3.org/1999/xhtml">**

**<p>FORMULA: C26H45NO6S</p>**

**<p>CHARGE: 0</p>**

**<p>PUBCHEM.COMPOUND: 10591 || 387316</p>**

**<p>INCHIKEY: BHTRKEVKTKCXOH-BJLOMENOSA-N</p>**

**<p>KEGG.COMPOUND: C05465</p>**

**<p>HMDB: HMDB00951</p>**

**<p>CHEBI: CHEBI:9407 || CHEBI:16525</p>**

**<p>INCHI: InChI=1S/C26H45NO6S/c1-16(4-7-23(30)27-12-13-34(31,32)33)19-5-6-20-24-21(9-11-26(19,20)3)25(2)10-8-18(28)14-17(25)15-22(24)29/h16-22,24,28-29H,4-15H2,1-3H3,(H,27,30)(H,31,32,33)/t16-,17+,18-,19-,20+,21+,22-,24+,25+,26-/m1/s1</p>**

**</body>**

**</notes>**

**<annotation>**

**<rdf:RDF xmlns:rdf="http://www.w3.org/1999/02/22-rdf-syntax-ns#" xmlns:bqmodel="http://biomodels.net/model-qualifiers/" xmlns:bqbiol="http://biomodels.net/biology-qualifiers/">**

**<rdf:Description rdf:about="f7adbcce-2277-4462-8747-ee7ce6b5b613">**

**<bqbiol:is>**

**<rdf:Bag>**

**<rdf:li rdf:resource="http://identifiers.org/PUBCHEM.COMPOUND/10591"/></rdf:Bag></bqbiol:is><bqbiol:is>**

**<rdf:Bag>**

**<rdf:li rdf:resource="http://identifiers.org/PUBCHEM.COMPOUND/387316"/></rdf:Bag></bqbiol:is><bqbiol:is>**

**<rdf:Bag>**

**<rdf:li rdf:resource="http://identifiers.org/INCHIKEY/BHTRKEVKTKCXOH-BJLOMENOSA-N"/></rdf:Bag></bqbiol:is><bqbiol:is>**

**<rdf:Bag>**

**<rdf:li rdf:resource="http://identifiers.org/KEGG.COMPOUND/C05465"/></rdf:Bag></bqbiol:is><bqbiol:is>**

**<rdf:Bag>**

**<rdf:li rdf:resource="http://identifiers.org/HMDB/HMDB00951"/></rdf:Bag></bqbiol:is><bqbiol:is>**

**<rdf:Bag>**

**<rdf:li rdf:resource="http://identifiers.org/CHEBI/CHEBI:9407"/></rdf:Bag></bqbiol:is><bqbiol:is>**

**<rdf:Bag>**

**<rdf:li rdf:resource="http://identifiers.org/CHEBI/CHEBI:16525"/></rdf:Bag></bqbiol:is><bqbiol:is>**

**<rdf:Bag>**

**<rdf:li rdf:resource="http://identifiers.org/INCHI/InChI=1S/C26H45NO6S/c1-16(4-7-23(30)27-12-13-34(31,32)33)19-5-6-20-24-21(9-11-26(19,20)3)25(2)10-8-18(28)14-17(25)15-22(24)29/h16-22,24,28-29H,4-15H2,1-3H3,(H,27,30)(H,31,32,33)/t16-,17+,18-,19-,20+,21+,22-,24+,25+,26-/m1/s1"/></rdf:Bag></bqbiol:is></rdf:Description></rdf:RDF>**

**</annotation>**

**</species>**

**<species id="M_C09640" constant="false" hasOnlySubstanceUnits="false" name="(-)-Salsoline" metaid="025637ff-bca5-4391-ad1d-2da623bd2da0" boundaryCondition="false" compartment="metaComp">**

**<notes>**

**<body xmlns="http://www.w3.org/1999/xhtml">**

**<p>FORMULA: C11H16NO2</p>**

**<p>CHARGE: 0</p>**

**<p>PUBCHEM.COMPOUND: 11830 || 442356</p>**

**<p>INCHIKEY: YTPRLBGPGZHUPD-ZETCQYMHSA-N</p>**

**<p>KEGG.COMPOUND: C09640</p>**

**<p>HMDB: HMDB12469</p>**

**<p>CHEBI: CHEBI:112 || CHEBI:761542</p>**

**<p>INCHI: InChI=1S/C11H15NO2/c1-7-9-6-11(14-2)10(13)5-8(9)3-4-12-7/h5-7,12-13H,3-4H2,1-2H3/t7-/m0/s1 || InChI=1/C11H15NO2/c1-7-9-6-11(14-2)10(13)5-8(9)3-4-12-7/h5-7,12-13H,3-4H2,1-2H3/t7-/m0/s1</p>**

**</body>**

**</notes>**

**<annotation>**

**<rdf:RDF xmlns:rdf="http://www.w3.org/1999/02/22-rdf-syntax-ns#" xmlns:bqmodel="http://biomodels.net/model-qualifiers/" xmlns:bqbiol="http://biomodels.net/biology-qualifiers/">**

**<rdf:Description rdf:about="_025637ff-bca5-4391-ad1d-2da623bd2da0">**

**<bqbiol:is>**

**<rdf:Bag>**

**<rdf:li rdf:resource="http://identifiers.org/PUBCHEM.COMPOUND/11830"/></rdf:Bag></bqbiol:is><bqbiol:is>**

**<rdf:Bag>**

**<rdf:li rdf:resource="http://identifiers.org/PUBCHEM.COMPOUND/442356"/></rdf:Bag></bqbiol:is><bqbiol:is>**

**<rdf:Bag>**

**<rdf:li rdf:resource="http://identifiers.org/INCHIKEY/YTPRLBGPGZHUPD-ZETCQYMHSA-N"/></rdf:Bag></bqbiol:is><bqbiol:is>**

**<rdf:Bag>**

**<rdf:li rdf:resource="http://identifiers.org/KEGG.COMPOUND/C09640"/></rdf:Bag></bqbiol:is><bqbiol:is>**

**<rdf:Bag>**

**<rdf:li rdf:resource="http://identifiers.org/HMDB/HMDB12469"/></rdf:Bag></bqbiol:is><bqbiol:is>**

**<rdf:Bag>**

**<rdf:li rdf:resource="http://identifiers.org/CHEBI/CHEBI:112"/></rdf:Bag></bqbiol:is><bqbiol:is>**

**<rdf:Bag>**

**<rdf:li rdf:resource="http://identifiers.org/CHEBI/CHEBI:761542"/></rdf:Bag></bqbiol:is><bqbiol:is>**

**<rdf:Bag>**

**<rdf:li rdf:resource="http://identifiers.org/INCHI/InChI=1S/C11H15NO2/c1-7-9-6-11(14-2)10(13)5-8(9)3-4-12-7/h5-7,12-13H,3-4H2,1-2H3/t7-/m0/s1"/></rdf:Bag></bqbiol:is><bqbiol:is>**

**<rdf:Bag>**

**<rdf:li rdf:resource="http://identifiers.org/INCHI/InChI=1/C11H15NO2/c1-7-9-6-11(14-2)10(13)5-8(9)3-4-12-7/h5-7,12-13H,3-4H2,1-2H3/t7-/m0/s1"/></rdf:Bag></bqbiol:is></rdf:Description></rdf:RDF>**

**</annotation>**

**</species>**

**<species id="M_h2o" constant="false" hasOnlySubstanceUnits="false" name="H2O" metaid="9eb55933-0765-46a8-a0c1-f2d588c1650c" boundaryCondition="false" compartment="metaComp">**

**<notes>**

**<body xmlns="http://www.w3.org/1999/xhtml">**

**<p>FORMULA: H2O</p>**

**<p>CHARGE: 0</p>**

**<p>PUBCHEM.COMPOUND: 962 || 22247451</p>**

**<p>INCHIKEY: XLYOFNOQVPJJNP-UHFFFAOYSA-N</p>**

**<p>HMDB: HMDB02111</p>**

**<p>KEGG.COMPOUND: C00001</p>**

**<p>CHEBI: CHEBI:15377 || CHEBI:29375</p>**

**<p>INCHI: InChI=1S/H2O/h1H2</p>**

**</body>**

**</notes>**

**<annotation>**

**<rdf:RDF xmlns:rdf="http://www.w3.org/1999/02/22-rdf-syntax-ns#" xmlns:bqmodel="http://biomodels.net/model-qualifiers/" xmlns:bqbiol="http://biomodels.net/biology-qualifiers/">**

**<rdf:Description rdf:about="_9eb55933-0765-46a8-a0c1-f2d588c1650c">**

**<bqbiol:is>**

**<rdf:Bag>**

**<rdf:li rdf:resource="http://identifiers.org/PUBCHEM.COMPOUND/962"/></rdf:Bag></bqbiol:is><bqbiol:is>**

**<rdf:Bag>**

**<rdf:li rdf:resource="http://identifiers.org/PUBCHEM.COMPOUND/22247451"/></rdf:Bag></bqbiol:is><bqbiol:is>**

**<rdf:Bag>**

**<rdf:li rdf:resource="http://identifiers.org/INCHIKEY/XLYOFNOQVPJJNP-UHFFFAOYSA-N"/></rdf:Bag></bqbiol:is><bqbiol:is>**

**<rdf:Bag>**

**<rdf:li rdf:resource="http://identifiers.org/HMDB/HMDB02111"/></rdf:Bag></bqbiol:is><bqbiol:is>**

**<rdf:Bag>**

**<rdf:li rdf:resource="http://identifiers.org/KEGG.COMPOUND/C00001"/></rdf:Bag></bqbiol:is><bqbiol:is>**

**<rdf:Bag>**

**<rdf:li rdf:resource="http://identifiers.org/CHEBI/CHEBI:15377"/></rdf:Bag></bqbiol:is><bqbiol:is>**

**<rdf:Bag>**

**<rdf:li rdf:resource="http://identifiers.org/CHEBI/CHEBI:29375"/></rdf:Bag></bqbiol:is><bqbiol:is>**

**<rdf:Bag>**

**<rdf:li rdf:resource="http://identifiers.org/INCHI/InChI=1S/H2O/h1H2"/></rdf:Bag></bqbiol:is></rdf:Description></rdf:RDF>**

**</annotation>**

**</species>**

**<species id="M_3odcoa" constant="false" hasOnlySubstanceUnits="false" name="3-Oxodecanoyl-CoA" metaid="d5475174-73ef-4a6d-a228-2b68134509a1" boundaryCondition="false" compartment="metaComp">**

**<notes>**

**<body xmlns="http://www.w3.org/1999/xhtml">**

**<p>FORMULA: C31H48N7O18P3S</p>**

**<p>CHARGE: 0</p>**

**<p>PUBCHEM.COMPOUND: 440606</p>**

**<p>INCHIKEY: AZCVXMAPLHSIKY-HSJNEKGZSA-N</p>**

**<p>KEGG.COMPOUND: C05265</p>**

**<p>HMDB: HMDB03939</p>**

**<p>CHEBI: CHEBI:62548 || CHEBI:28528</p>**

**<p>INCHI: InChI=1S/C31H52N7O18P3S/c1-4-5-6-7-8-9-19(39)14-22(41)60-13-12-33-21(40)10-11-34-29(44)26(43)31(2,3)16-53-59(50,51)56-58(48,49)52-15-20-25(55-57(45,46)47)24(42)30(54-20)38-18-37-23-27(32)35-17-36-28(23)38/h17-18,20,24-26,30,42-43H,4-16H2,1-3H3,(H,33,40)(H,34,44)(H,48,49)(H,50,51)(H2,32,35,36)(H2,45,46,47)/t20-,24-,25-,26+,30-/m1/s1 || InChI=1S/C31H52N7O18P3S/c1-4-5-6-7-8-9-19(39)14-22(41)60-13-12-33-21(40)10-11-34-29(44)26(43)31(2,3)16-53-59(50,51)56-58(48,49)52-15-20-25(55-57(45,46)47)24(42)30(54-20)38-18-37-23-27(32)35-17-36-28(23)38/h17-18,20,24-26,30,42-43H,4-16H2,1-3H3,(H,33,40)(H,34,44)(H,48,49)(H,50,51)(H2,32,35,36)(H2,45,46,47)/t20-,24-,25-,26?,30-/m1/s1 || InChI=1S/C31H52N7O18P3S/c1-4-5-6-7-8-9-19(39)14-22(41)60-13-12-33-21(40)10-11-34-29(44)26(43)31(2,3)16-53-59(50,51)56-58(48,49)52-15-20-25(55-57(45,46)47)24(42)30(54-20)38-18-37-23-27(32)35-17-36-28(23)38/h17-18,20,24-26,30,42-43H,4-16H2,1-3H3,(H,33,40)(H,34,44)(H,48,49)(H,50,51)(H2,32,35,36)(H2,45,46,47)/p-4/t20-,24-,25-,26+,30-/m1/s1</p>**

**</body>**

**</notes>**

**<annotation>**

**<rdf:RDF xmlns:rdf="http://www.w3.org/1999/02/22-rdf-syntax-ns#" xmlns:bqmodel="http://biomodels.net/model-qualifiers/" xmlns:bqbiol="http://biomodels.net/biology-qualifiers/">**

**<rdf:Description rdf:about="d5475174-73ef-4a6d-a228-2b68134509a1">**

**<bqbiol:is>**

**<rdf:Bag>**

**<rdf:li rdf:resource="http://identifiers.org/PUBCHEM.COMPOUND/440606"/></rdf:Bag></bqbiol:is><bqbiol:is>**

**<rdf:Bag>**

**<rdf:li rdf:resource="http://identifiers.org/INCHIKEY/AZCVXMAPLHSIKY-HSJNEKGZSA-N"/></rdf:Bag></bqbiol:is><bqbiol:is>**

**<rdf:Bag>**

**<rdf:li rdf:resource="http://identifiers.org/KEGG.COMPOUND/C05265"/></rdf:Bag></bqbiol:is><bqbiol:is>**

**<rdf:Bag>**

**<rdf:li rdf:resource="http://identifiers.org/HMDB/HMDB03939"/></rdf:Bag></bqbiol:is><bqbiol:is>**

**<rdf:Bag>**

**<rdf:li rdf:resource="http://identifiers.org/CHEBI/CHEBI:62548"/></rdf:Bag></bqbiol:is><bqbiol:is>**

**<rdf:Bag>**

**<rdf:li rdf:resource="http://identifiers.org/CHEBI/CHEBI:28528"/></rdf:Bag></bqbiol:is><bqbiol:is>**

**<rdf:Bag>**

**<rdf:li rdf:resource="http://identifiers.org/INCHI/InChI=1S/C31H52N7O18P3S/c1-4-5-6-7-8-9-19(39)14-22(41)60-13-12-33-21(40)10-11-34-29(44)26(43)31(2,3)16-53-59(50,51)56-58(48,49)52-15-20-25(55-57(45,46)47)24(42)30(54-20)38-18-37-23-27(32)35-17-36-28(23)38/h17-18,20,24-26,30,42-43H,4-16H2,1-3H3,(H,33,40)(H,34,44)(H,48,49)(H,50,51)(H2,32,35,36)(H2,45,46,47)/t20-,24-,25-,26+,30-/m1/s1"/></rdf:Bag></bqbiol:is><bqbiol:is>**

**<rdf:Bag>**

**<rdf:li rdf:resource="http://identifiers.org/INCHI/InChI=1S/C31H52N7O18P3S/c1-4-5-6-7-8-9-19(39)14-22(41)60-13-12-33-21(40)10-11-34-29(44)26(43)31(2,3)16-53-59(50,51)56-58(48,49)52-15-20-25(55-57(45,46)47)24(42)30(54-20)38-18-37-23-27(32)35-17-36-28(23)38/h17-18,20,24-26,30,42-43H,4-16H2,1-3H3,(H,33,40)(H,34,44)(H,48,49)(H,50,51)(H2,32,35,36)(H2,45,46,47)/t20-,24-,25-,26?,30-/m1/s1"/></rdf:Bag></bqbiol:is><bqbiol:is>**

**<rdf:Bag>**

**<rdf:li rdf:resource="http://identifiers.org/INCHI/InChI=1S/C31H52N7O18P3S/c1-4-5-6-7-8-9-19(39)14-22(41)60-13-12-33-21(40)10-11-34-29(44)26(43)31(2,3)16-53-59(50,51)56-58(48,49)52-15-20-25(55-57(45,46)47)24(42)30(54-20)38-18-37-23-27(32)35-17-36-28(23)38/h17-18,20,24-26,30,42-43H,4-16H2,1-3H3,(H,33,40)(H,34,44)(H,48,49)(H,50,51)(H2,32,35,36)(H2,45,46,47)/p-4/t20-,24-,25-,26+,30-/m1/s1"/></rdf:Bag></bqbiol:is></rdf:Description></rdf:RDF>**

**</annotation>**

**</species>**

**<species id="M_CE5783" constant="false" hasOnlySubstanceUnits="false" name="Somatostatin fragment 3-14" metaid="1ecf10c1-faff-4a59-9837-7aff93283102" boundaryCondition="false" compartment="metaComp">**

**<notes>**

**<body xmlns="http://www.w3.org/1999/xhtml">**

**<p>FORMULA: C72H100N16O17S2</p>**

**<p>CHARGE: 0</p>**

**<p>PUBCHEM.COMPOUND: 53481604</p>**

**<p>INCHIKEY: TZTLCJNYBHBVBN-DZCACUQDSA-N</p>**

**<p>HMDB: HMDB13071</p>**

**<p>INCHI: InChI=1S/C72H98N16O17S2/c1-40(90)59-70(102)85-54(34-44-22-10-5-11-23-44)68(100)88-60(41(2)91)71(103)86-57(38-89)69(101)80-51(72(104)105)28-31-106-107-39-47(75)61(93)78-49(26-14-16-29-73)62(94)84-56(36-58(76)92)67(99)82-52(32-42-18-6-3-7-19-42)64(96)81-53(33-43-20-8-4-9-21-43)65(97)83-55(35-45-37-77-48-25-13-12-24-46(45)48)66(98)79-50(63(95)87-59)27-15-17-30-74/h3-13,18-25,37,40-41,47,49-57,59-60,77,89-91H,14-17,26-36,38-39,73-75H2,1-2H3,(H2,76,92)(H,78,93)(H,79,98)(H,80,101)(H,81,96)(H,82,99)(H,83,97)(H,84,94)(H,85,102)(H,86,103)(H,87,95)(H,88,100)(H,104,105)/t40?,41-,47-,49+,50+,51-,52+,53+,54+,55+,56+,57+,59?,60+/m0/s1</p>**

**</body>**

**</notes>**

**<annotation>**

**<rdf:RDF xmlns:rdf="http://www.w3.org/1999/02/22-rdf-syntax-ns#" xmlns:bqmodel="http://biomodels.net/model-qualifiers/" xmlns:bqbiol="http://biomodels.net/biology-qualifiers/">**

**<rdf:Description rdf:about="_1ecf10c1-faff-4a59-9837-7aff93283102">**

**<bqbiol:is>**

**<rdf:Bag>**

**<rdf:li rdf:resource="http://identifiers.org/PUBCHEM.COMPOUND/53481604"/></rdf:Bag></bqbiol:is><bqbiol:is>**

**<rdf:Bag>**

**<rdf:li rdf:resource="http://identifiers.org/INCHIKEY/TZTLCJNYBHBVBN-DZCACUQDSA-N"/></rdf:Bag></bqbiol:is><bqbiol:is>**

**<rdf:Bag>**

**<rdf:li rdf:resource="http://identifiers.org/HMDB/HMDB13071"/></rdf:Bag></bqbiol:is><bqbiol:is>**

**<rdf:Bag>**

**<rdf:li rdf:resource="http://identifiers.org/INCHI/InChI=1S/C72H98N16O17S2/c1-40(90)59-70(102)85-54(34-44-22-10-5-11-23-44)68(100)88-60(41(2)91)71(103)86-57(38-89)69(101)80-51(72(104)105)28-31-106-107-39-47(75)61(93)78-49(26-14-16-29-73)62(94)84-56(36-58(76)92)67(99)82-52(32-42-18-6-3-7-19-42)64(96)81-53(33-43-20-8-4-9-21-43)65(97)83-55(35-45-37-77-48-25-13-12-24-46(45)48)66(98)79-50(63(95)87-59)27-15-17-30-74/h3-13,18-25,37,40-41,47,49-57,59-60,77,89-91H,14-17,26-36,38-39,73-75H2,1-2H3,(H2,76,92)(H,78,93)(H,79,98)(H,80,101)(H,81,96)(H,82,99)(H,83,97)(H,84,94)(H,85,102)(H,86,103)(H,87,95)(H,88,100)(H,104,105)/t40?,41-,47-,49+,50+,51-,52+,53+,54+,55+,56+,57+,59?,60+/m0/s1"/></rdf:Bag></bqbiol:is></rdf:Description></rdf:RDF>**

**</annotation>**

**</species>**

**<species id="M_tdcoa" constant="false" hasOnlySubstanceUnits="false" name="Tetradecanoyl-CoA (n-C14:0CoA)" metaid="e938a880-6592-4f1f-bc35-e8bf835be898" boundaryCondition="false" compartment="metaComp">**

**<notes>**

**<body xmlns="http://www.w3.org/1999/xhtml">**

**<p>FORMULA: C35H58N7O17P3S</p>**

**<p>CHARGE: 0</p>**

**<p>PUBCHEM.COMPOUND: 25244582 || 65113</p>**

**<p>INCHIKEY: DUAFKXOFBZQTQE-QSGBVPJFSA-J</p>**

**<p>KEGG.COMPOUND: C02593</p>**

**<p>HMDB: HMDB01521</p>**

**<p>CHEBI: CHEBI:15532 || CHEBI:57385</p>**

**<p>INCHI: InChI=1S/C35H62N7O17P3S/c1-4-5-6-7-8-9-10-11-12-13-14-15-26(44)63-19-18-37-25(43)16-17-38-33(47)30(46)35(2,3)21-56-62(53,54)59-61(51,52)55-20-24-29(58-60(48,49)50)28(45)34(57-24)42-23-41-27-31(36)39-22-40-32(27)42/h22-24,28-30,34,45-46H,4-21H2,1-3H3,(H,37,43)(H,38,47)(H,51,52)(H,53,54)(H2,36,39,40)(H2,48,49,50)/t24-,28-,29-,30?,34-/m1/s1 || InChI=1S/C35H62N7O17P3S/c1-4-5-6-7-8-9-10-11-12-13-14-15-26(44)63-19-18-37-25(43)16-17-38-33(47)30(46)35(2,3)21-56-62(53,54)59-61(51,52)55-20-24-29(58-60(48,49)50)28(45)34(57-24)42-23-41-27-31(36)39-22-40-32(27)42/h22-24,28-30,34,45-46H,4-21H2,1-3H3,(H,37,43)(H,38,47)(H,51,52)(H,53,54)(H2,36,39,40)(H2,48,49,50)/p-4/t24-,28-,29-,30+,34-/m1/s1</p>**

**</body>**

**</notes>**

**<annotation>**

**<rdf:RDF xmlns:rdf="http://www.w3.org/1999/02/22-rdf-syntax-ns#" xmlns:bqmodel="http://biomodels.net/model-qualifiers/" xmlns:bqbiol="http://biomodels.net/biology-qualifiers/">**

**<rdf:Description rdf:about="e938a880-6592-4f1f-bc35-e8bf835be898">**

**<bqbiol:is>**

**<rdf:Bag>**

**<rdf:li rdf:resource="http://identifiers.org/PUBCHEM.COMPOUND/25244582"/></rdf:Bag></bqbiol:is><bqbiol:is>**

**<rdf:Bag>**

**<rdf:li rdf:resource="http://identifiers.org/PUBCHEM.COMPOUND/65113"/></rdf:Bag></bqbiol:is><bqbiol:is>**

**<rdf:Bag>**

**<rdf:li rdf:resource="http://identifiers.org/INCHIKEY/DUAFKXOFBZQTQE-QSGBVPJFSA-J"/></rdf:Bag></bqbiol:is><bqbiol:is>**

**<rdf:Bag>**

**<rdf:li rdf:resource="http://identifiers.org/KEGG.COMPOUND/C02593"/></rdf:Bag></bqbiol:is><bqbiol:is>**

**<rdf:Bag>**

**<rdf:li rdf:resource="http://identifiers.org/HMDB/HMDB01521"/></rdf:Bag></bqbiol:is><bqbiol:is>**

**<rdf:Bag>**

**<rdf:li rdf:resource="http://identifiers.org/CHEBI/CHEBI:15532"/></rdf:Bag></bqbiol:is><bqbiol:is>**

**<rdf:Bag>**

**<rdf:li rdf:resource="http://identifiers.org/CHEBI/CHEBI:57385"/></rdf:Bag></bqbiol:is><bqbiol:is>**

**<rdf:Bag>**

**<rdf:li rdf:resource="http://identifiers.org/INCHI/InChI=1S/C35H62N7O17P3S/c1-4-5-6-7-8-9-10-11-12-13-14-15-26(44)63-19-18-37-25(43)16-17-38-33(47)30(46)35(2,3)21-56-62(53,54)59-61(51,52)55-20-24-29(58-60(48,49)50)28(45)34(57-24)42-23-41-27-31(36)39-22-40-32(27)42/h22-24,28-30,34,45-46H,4-21H2,1-3H3,(H,37,43)(H,38,47)(H,51,52)(H,53,54)(H2,36,39,40)(H2,48,49,50)/t24-,28-,29-,30?,34-/m1/s1"/></rdf:Bag></bqbiol:is><bqbiol:is>**

**<rdf:Bag>**

**<rdf:li rdf:resource="http://identifiers.org/INCHI/InChI=1S/C35H62N7O17P3S/c1-4-5-6-7-8-9-10-11-12-13-14-15-26(44)63-19-18-37-25(43)16-17-38-33(47)30(46)35(2,3)21-56-62(53,54)59-61(51,52)55-20-24-29(58-60(48,49)50)28(45)34(57-24)42-23-41-27-31(36)39-22-40-32(27)42/h22-24,28-30,34,45-46H,4-21H2,1-3H3,(H,37,43)(H,38,47)(H,51,52)(H,53,54)(H2,36,39,40)(H2,48,49,50)/p-4/t24-,28-,29-,30+,34-/m1/s1"/></rdf:Bag></bqbiol:is></rdf:Description></rdf:RDF>**

**</annotation>**

**</species>**

**<species id="M_CE5782" constant="false" hasOnlySubstanceUnits="false" name="somatostatin" metaid="6a5f0038-bd7b-4b30-91e5-83ab9203b91d" boundaryCondition="false" compartment="metaComp">**

**<notes>**

**<body xmlns="http://www.w3.org/1999/xhtml">**

**<p>FORMULA: C76H106N18O19S2</p>**

**<p>CHARGE: 0</p>**

**<p>PUBCHEM.COMPOUND: 53481605 || 44291156</p>**

**<p>INCHIKEY: KTEPWBJBSMMZIC-LJTSQLRWSA-N</p>**

**<p>HMDB: HMDB13072</p>**

**<p>KEGG.COMPOUND: C16022</p>**

**<p>CHEBI: CHEBI:64628</p>**

**<p>INCHI: InChI=1S/C76H104N18O19S2/c1-41(79)64(100)82-37-61(99)83-58-39-114-115-40-59(76(112)113)92-72(108)57(38-95)91-75(111)63(43(3)97)94-71(107)54(33-46-23-11-6-12-24-46)90-74(110)62(42(2)96)93-66(102)51(28-16-18-30-78)84-69(105)55(34-47-36-81-49-26-14-13-25-48(47)49)88-68(104)53(32-45-21-9-5-10-22-45)86-67(103)52(31-44-19-7-4-8-20-44)87-70(106)56(35-60(80)98)89-65(101)50(85-73(58)109)27-15-17-29-77/h4-14,19-26,36,41-43,50-59,62-63,81,95-97H,15-18,27-35,37-40,77-79H2,1-3H3,(H2,80,98)(H,82,100)(H,83,99)(H,84,105)(H,85,109)(H,86,103)(H,87,106)(H,88,104)(H,89,101)(H,90,110)(H,91,111)(H,92,108)(H,93,102)(H,94,107)(H,112,113)/t41-,42+,43+,50+,51-,52+,53-,54-,55+,56-,57-,58+,59-,62+,63+/m0/s1 || InChI=1S/C77H106N18O19S2/c1-42(80)65(101)83-39-62(100)84-60-41-116-115-32-29-53(77(113)114)87-73(109)59(40-96)93-76(112)64(44(3)98)95-72(108)56(35-47-23-11-6-12-24-47)92-75(111)63(43(2)97)94-67(103)52(28-16-18-31-79)85-70(106)57(36-48-38-82-50-26-14-13-25-49(48)50)90-69(105)55(34-46-21-9-5-10-22-46)88-68(104)54(33-45-19-7-4-8-20-45)89-71(107)58(37-61(81)99)91-66(102)51(86-74(60)110)27-15-17-30-78/h4-14,19-26,38,42-44,51-60,63-64,82,96-98H,15-18,27-37,39-41,78-80H2,1-3H3,(H2,81,99)(H,83,101)(H,84,100)(H,85,106)(H,86,110)(H,87,109)(H,88,104)(H,89,107)(H,90,105)(H,91,102)(H,92,111)(H,93,112)(H,94,103)(H,95,108)(H,113,114)/t42-,43+,44?,51-,52-,53+,54-,55-,56-,57-,58-,59-,60?,63-,64?/m1/s1 || InChI=1S/C76H104N18O19S2/c1-41(79)64(100)82-37-61(99)83-58-39-114-115-40-59(76(112)113)92-72(108)57(38-95)91-75(111)63(43(3)97)94-71(107)54(33-46-23-11-6-12-24-46)90-74(110)62(42(2)96)93-66(102)51(28-16-18-30-78)84-69(105)55(34-47-36-81-49-26-14-13-25-48(47)49)88-68(104)53(32-45-21-9-5-10-22-45)86-67(103)52(31-44-19-7-4-8-20-44)87-70(106)56(35-60(80)98)89-65(101)50(85-73(58)109)27-15-17-29-77/h4-14,19-26,36,41-43,50-59,62-63,81,95-97H,15-18,27-35,37-40,77-79H2,1-3H3,(H2,80,98)(H,82,100)(H,83,99)(H,84,105)(H,85,109)(H,86,103)(H,87,106)(H,88,104)(H,89,101)(H,90,110)(H,91,111)(H,92,108)(H,93,102)(H,94,107)(H,112,113)/t41-,42+,43+,50-,51-,52-,53-,54-,55-,56-,57-,58-,59-,62-,63-/m0/s1</p>**

**</body>**

**</notes>**

**<annotation>**

**<rdf:RDF xmlns:rdf="http://www.w3.org/1999/02/22-rdf-syntax-ns#" xmlns:bqmodel="http://biomodels.net/model-qualifiers/" xmlns:bqbiol="http://biomodels.net/biology-qualifiers/">**

**<rdf:Description rdf:about="_6a5f0038-bd7b-4b30-91e5-83ab9203b91d">**

**<bqbiol:is>**

**<rdf:Bag>**

**<rdf:li rdf:resource="http://identifiers.org/PUBCHEM.COMPOUND/53481605"/></rdf:Bag></bqbiol:is><bqbiol:is>**

**<rdf:Bag>**

**<rdf:li rdf:resource="http://identifiers.org/PUBCHEM.COMPOUND/44291156"/></rdf:Bag></bqbiol:is><bqbiol:is>**

**<rdf:Bag>**

**<rdf:li rdf:resource="http://identifiers.org/INCHIKEY/KTEPWBJBSMMZIC-LJTSQLRWSA-N"/></rdf:Bag></bqbiol:is><bqbiol:is>**

**<rdf:Bag>**

**<rdf:li rdf:resource="http://identifiers.org/HMDB/HMDB13072"/></rdf:Bag></bqbiol:is><bqbiol:is>**

**<rdf:Bag>**

**<rdf:li rdf:resource="http://identifiers.org/KEGG.COMPOUND/C16022"/></rdf:Bag></bqbiol:is><bqbiol:is>**

**<rdf:Bag>**

**<rdf:li rdf:resource="http://identifiers.org/CHEBI/CHEBI:64628"/></rdf:Bag></bqbiol:is><bqbiol:is>**

**<rdf:Bag>**

**<rdf:li rdf:resource="http://identifiers.org/INCHI/InChI=1S/C76H104N18O19S2/c1-41(79)64(100)82-37-61(99)83-58-39-114-115-40-59(76(112)113)92-72(108)57(38-95)91-75(111)63(43(3)97)94-71(107)54(33-46-23-11-6-12-24-46)90-74(110)62(42(2)96)93-66(102)51(28-16-18-30-78)84-69(105)55(34-47-36-81-49-26-14-13-25-48(47)49)88-68(104)53(32-45-21-9-5-10-22-45)86-67(103)52(31-44-19-7-4-8-20-44)87-70(106)56(35-60(80)98)89-65(101)50(85-73(58)109)27-15-17-29-77/h4-14,19-26,36,41-43,50-59,62-63,81,95-97H,15-18,27-35,37-40,77-79H2,1-3H3,(H2,80,98)(H,82,100)(H,83,99)(H,84,105)(H,85,109)(H,86,103)(H,87,106)(H,88,104)(H,89,101)(H,90,110)(H,91,111)(H,92,108)(H,93,102)(H,94,107)(H,112,113)/t41-,42+,43+,50+,51-,52+,53-,54-,55+,56-,57-,58+,59-,62+,63+/m0/s1"/></rdf:Bag></bqbiol:is><bqbiol:is>**

**<rdf:Bag>**

**<rdf:li rdf:resource="http://identifiers.org/INCHI/InChI=1S/C77H106N18O19S2/c1-42(80)65(101)83-39-62(100)84-60-41-116-115-32-29-53(77(113)114)87-73(109)59(40-96)93-76(112)64(44(3)98)95-72(108)56(35-47-23-11-6-12-24-47)92-75(111)63(43(2)97)94-67(103)52(28-16-18-31-79)85-70(106)57(36-48-38-82-50-26-14-13-25-49(48)50)90-69(105)55(34-46-21-9-5-10-22-46)88-68(104)54(33-45-19-7-4-8-20-45)89-71(107)58(37-61(81)99)91-66(102)51(86-74(60)110)27-15-17-30-78/h4-14,19-26,38,42-44,51-60,63-64,82,96-98H,15-18,27-37,39-41,78-80H2,1-3H3,(H2,81,99)(H,83,101)(H,84,100)(H,85,106)(H,86,110)(H,87,109)(H,88,104)(H,89,107)(H,90,105)(H,91,102)(H,92,111)(H,93,112)(H,94,103)(H,95,108)(H,113,114)/t42-,43+,44?,51-,52-,53+,54-,55-,56-,57-,58-,59-,60?,63-,64?/m1/s1"/></rdf:Bag></bqbiol:is><bqbiol:is>**

**<rdf:Bag>**

**<rdf:li rdf:resource="http://identifiers.org/INCHI/InChI=1S/C76H104N18O19S2/c1-41(79)64(100)82-37-61(99)83-58-39-114-115-40-59(76(112)113)92-72(108)57(38-95)91-75(111)63(43(3)97)94-71(107)54(33-46-23-11-6-12-24-46)90-74(110)62(42(2)96)93-66(102)51(28-16-18-30-78)84-69(105)55(34-47-36-81-49-26-14-13-25-48(47)49)88-68(104)53(32-45-21-9-5-10-22-45)86-67(103)52(31-44-19-7-4-8-20-44)87-70(106)56(35-60(80)98)89-65(101)50(85-73(58)109)27-15-17-29-77/h4-14,19-26,36,41-43,50-59,62-63,81,95-97H,15-18,27-35,37-40,77-79H2,1-3H3,(H2,80,98)(H,82,100)(H,83,99)(H,84,105)(H,85,109)(H,86,103)(H,87,106)(H,88,104)(H,89,101)(H,90,110)(H,91,111)(H,92,108)(H,93,102)(H,94,107)(H,112,113)/t41-,42+,43+,50-,51-,52-,53-,54-,55-,56-,57-,58-,59-,62-,63-/m0/s1"/></rdf:Bag></bqbiol:is></rdf:Description></rdf:RDF>**

**</annotation>**

**</species>**

**<species id="M_C09642" constant="false" hasOnlySubstanceUnits="false" name="(-)-Salsolinol" metaid="475d5018-2b41-455b-b7a3-2c2aa1acf384" boundaryCondition="false" compartment="metaComp">**

**<notes>**

**<body xmlns="http://www.w3.org/1999/xhtml">**

**<p>FORMULA: C10H14NO2</p>**

**<p>CHARGE: 0</p>**

**<p>PUBCHEM.COMPOUND: 91588 || 11832 || 54456</p>**

**<p>INCHIKEY: IBRKLUSXDYATLG-LURJTMIESA-N</p>**

**<p>KEGG.COMPOUND: C09642</p>**

**<p>HMDB: HMDB05199</p>**

**<p>CHEBI: CHEBI:113 || CHEBI:123715</p>**

**<p>INCHI: InChI=1S/C10H13NO2/c1-6-8-5-10(13)9(12)4-7(8)2-3-11-6/h4-6,11-13H,2-3H2,1H3/t6-/m0/s1 || InChI=1S/C10H13NO2/c1-6-8-5-10(13)9(12)4-7(8)2-3-11-6/h4-6,11-13H,2-3H2,1H3</p>**

**</body>**

**</notes>**

**<annotation>**

**<rdf:RDF xmlns:rdf="http://www.w3.org/1999/02/22-rdf-syntax-ns#" xmlns:bqmodel="http://biomodels.net/model-qualifiers/" xmlns:bqbiol="http://biomodels.net/biology-qualifiers/">**

**<rdf:Description rdf:about="_475d5018-2b41-455b-b7a3-2c2aa1acf384">**

**<bqbiol:is>**

**<rdf:Bag>**

**<rdf:li rdf:resource="http://identifiers.org/PUBCHEM.COMPOUND/91588"/></rdf:Bag></bqbiol:is><bqbiol:is>**

**<rdf:Bag>**

**<rdf:li rdf:resource="http://identifiers.org/PUBCHEM.COMPOUND/11832"/></rdf:Bag></bqbiol:is><bqbiol:is>**

**<rdf:Bag>**

**<rdf:li rdf:resource="http://identifiers.org/PUBCHEM.COMPOUND/54456"/></rdf:Bag></bqbiol:is><bqbiol:is>**

**<rdf:Bag>**

**<rdf:li rdf:resource="http://identifiers.org/INCHIKEY/IBRKLUSXDYATLG-LURJTMIESA-N"/></rdf:Bag></bqbiol:is><bqbiol:is>**

**<rdf:Bag>**

**<rdf:li rdf:resource="http://identifiers.org/KEGG.COMPOUND/C09642"/></rdf:Bag></bqbiol:is><bqbiol:is>**

**<rdf:Bag>**

**<rdf:li rdf:resource="http://identifiers.org/HMDB/HMDB05199"/></rdf:Bag></bqbiol:is><bqbiol:is>**

**<rdf:Bag>**

**<rdf:li rdf:resource="http://identifiers.org/CHEBI/CHEBI:113"/></rdf:Bag></bqbiol:is><bqbiol:is>**

**<rdf:Bag>**

**<rdf:li rdf:resource="http://identifiers.org/CHEBI/CHEBI:123715"/></rdf:Bag></bqbiol:is><bqbiol:is>**

**<rdf:Bag>**

**<rdf:li rdf:resource="http://identifiers.org/INCHI/InChI=1S/C10H13NO2/c1-6-8-5-10(13)9(12)4-7(8)2-3-11-6/h4-6,11-13H,2-3H2,1H3/t6-/m0/s1"/></rdf:Bag></bqbiol:is><bqbiol:is>**

**<rdf:Bag>**

**<rdf:li rdf:resource="http://identifiers.org/INCHI/InChI=1S/C10H13NO2/c1-6-8-5-10(13)9(12)4-7(8)2-3-11-6/h4-6,11-13H,2-3H2,1H3"/></rdf:Bag></bqbiol:is></rdf:Description></rdf:RDF>**

**</annotation>**

**</species>**

**<species id="M_dhcrm_hs" constant="false" hasOnlySubstanceUnits="false" name="dihydroceramide" metaid="9b4f37ee-449b-48cc-807f-2b20e2b327cf" boundaryCondition="false" compartment="metaComp">**

**<notes>**

**<body xmlns="http://www.w3.org/1999/xhtml">**

**<p>FORMULA: C18H38NO2FULLRCO</p>**

**<p>CHARGE: 0</p>**

**<p>PUBCHEM.COMPOUND: 16755624</p>**

**<p>INCHIKEY: XSDVOEIEBUGRQX-RBUKOAKNSA-N</p>**

**<p>HMDB: HMDB06752</p>**

**<p>KEGG.COMPOUND: C12126</p>**

**<p>CHEBI: CHEBI:31488</p>**

**<p>INCHI: InChI=1S/C19H39NO3/c1-2-3-4-5-6-7-8-9-10-11-12-13-14-15-19(23)18(16-21)20-17-22/h17-19,21,23H,2-16H2,1H3,(H,20,22)/t18-,19+/m0/s1</p>**

**</body>**

**</notes>**

**<annotation>**

**<rdf:RDF xmlns:rdf="http://www.w3.org/1999/02/22-rdf-syntax-ns#" xmlns:bqmodel="http://biomodels.net/model-qualifiers/" xmlns:bqbiol="http://biomodels.net/biology-qualifiers/">**

**<rdf:Description rdf:about="_9b4f37ee-449b-48cc-807f-2b20e2b327cf">**

**<bqbiol:is>**

**<rdf:Bag>**

**<rdf:li rdf:resource="http://identifiers.org/PUBCHEM.COMPOUND/16755624"/></rdf:Bag></bqbiol:is><bqbiol:is>**

**<rdf:Bag>**

**<rdf:li rdf:resource="http://identifiers.org/INCHIKEY/XSDVOEIEBUGRQX-RBUKOAKNSA-N"/></rdf:Bag></bqbiol:is><bqbiol:is>**

**<rdf:Bag>**

**<rdf:li rdf:resource="http://identifiers.org/HMDB/HMDB06752"/></rdf:Bag></bqbiol:is><bqbiol:is>**

**<rdf:Bag>**

**<rdf:li rdf:resource="http://identifiers.org/KEGG.COMPOUND/C12126"/></rdf:Bag></bqbiol:is><bqbiol:is>**

**<rdf:Bag>**

**<rdf:li rdf:resource="http://identifiers.org/CHEBI/CHEBI:31488"/></rdf:Bag></bqbiol:is><bqbiol:is>**

**<rdf:Bag>**

**<rdf:li rdf:resource="http://identifiers.org/INCHI/InChI=1S/C19H39NO3/c1-2-3-4-5-6-7-8-9-10-11-12-13-14-15-19(23)18(16-21)20-17-22/h17-19,21,23H,2-16H2,1H3,(H,20,22)/t18-,19+/m0/s1"/></rdf:Bag></bqbiol:is></rdf:Description></rdf:RDF>**

**</annotation>**

**</species>**

**<species id="M_val_L" constant="false" hasOnlySubstanceUnits="false" name="L-valine" metaid="aa94680c-1126-40bb-af9a-f78e2a9479de" boundaryCondition="false" compartment="metaComp">**

**<notes>**

**<body xmlns="http://www.w3.org/1999/xhtml">**

**<p>FORMULA: C5H11NO2</p>**

**<p>CHARGE: 0</p>**

**<p>PUBCHEM.COMPOUND: 1182 || 6287 || 5237032 || 6971018</p>**

**<p>INCHIKEY: KZSNJWFQEVHDMF-BYPYZUCNSA-N</p>**

**<p>KEGG.COMPOUND: C16436 || C00183</p>**

**<p>HMDB: HMDB00883</p>**

**<p>CHEBI: CHEBI:27266 || CHEBI:57762 || CHEBI:16414</p>**

**<p>INCHI: InChI=1S/C5H11NO2/c1-3(2)4(6)5(7)8/h3-4H,6H2,1-2H3,(H,7,8)/t4-/m0/s1</p>**

**</body>**

**</notes>**

**<annotation>**

**<rdf:RDF xmlns:rdf="http://www.w3.org/1999/02/22-rdf-syntax-ns#" xmlns:bqmodel="http://biomodels.net/model-qualifiers/" xmlns:bqbiol="http://biomodels.net/biology-qualifiers/">**

**<rdf:Description rdf:about="aa94680c-1126-40bb-af9a-f78e2a9479de">**

**<bqbiol:is>**

**<rdf:Bag>**

**<rdf:li rdf:resource="http://identifiers.org/PUBCHEM.COMPOUND/1182"/></rdf:Bag></bqbiol:is><bqbiol:is>**

**<rdf:Bag>**

**<rdf:li rdf:resource="http://identifiers.org/PUBCHEM.COMPOUND/6287"/></rdf:Bag></bqbiol:is><bqbiol:is>**

**<rdf:Bag>**

**<rdf:li rdf:resource="http://identifiers.org/PUBCHEM.COMPOUND/5237032"/></rdf:Bag></bqbiol:is><bqbiol:is>**

**<rdf:Bag>**

**<rdf:li rdf:resource="http://identifiers.org/PUBCHEM.COMPOUND/6971018"/></rdf:Bag></bqbiol:is><bqbiol:is>**

**<rdf:Bag>**

**<rdf:li rdf:resource="http://identifiers.org/INCHIKEY/KZSNJWFQEVHDMF-BYPYZUCNSA-N"/></rdf:Bag></bqbiol:is><bqbiol:is>**

**<rdf:Bag>**

**<rdf:li rdf:resource="http://identifiers.org/KEGG.COMPOUND/C16436"/></rdf:Bag></bqbiol:is><bqbiol:is>**

**<rdf:Bag>**

**<rdf:li rdf:resource="http://identifiers.org/KEGG.COMPOUND/C00183"/></rdf:Bag></bqbiol:is><bqbiol:is>**

**<rdf:Bag>**

**<rdf:li rdf:resource="http://identifiers.org/HMDB/HMDB00883"/></rdf:Bag></bqbiol:is><bqbiol:is>**

**<rdf:Bag>**

**<rdf:li rdf:resource="http://identifiers.org/CHEBI/CHEBI:27266"/></rdf:Bag></bqbiol:is><bqbiol:is>**

**<rdf:Bag>**

**<rdf:li rdf:resource="http://identifiers.org/CHEBI/CHEBI:57762"/></rdf:Bag></bqbiol:is><bqbiol:is>**

**<rdf:Bag>**

**<rdf:li rdf:resource="http://identifiers.org/CHEBI/CHEBI:16414"/></rdf:Bag></bqbiol:is><bqbiol:is>**

**<rdf:Bag>**

**<rdf:li rdf:resource="http://identifiers.org/INCHI/InChI=1S/C5H11NO2/c1-3(2)4(6)5(7)8/h3-4H,6H2,1-2H3,(H,7,8)/t4-/m0/s1"/></rdf:Bag></bqbiol:is></rdf:Description></rdf:RDF>**

**</annotation>**

**</species>**

**<species id="M_c81crn" constant="false" hasOnlySubstanceUnits="false" name="octenoyl carnitine" metaid="4a3f82f6-d95e-4c79-b9f8-703c029b3719" boundaryCondition="false" compartment="metaComp">**

**<notes>**

**<body xmlns="http://www.w3.org/1999/xhtml">**

**<p>FORMULA: C15H27NO4</p>**

**<p>CHARGE: 0</p>**

**<p>PUBCHEM.COMPOUND: 53481667</p>**

**<p>HMDB: HMDB13324</p>**

**<p>INCHI: InChI=1S/C15H27NO4/c1-5-6-7-8-9-10-15(19)20-13(16(2,3)4)11-12-14(17)18/h9-10,13H,5-8,11-12H2,1-4H3/b10-9+/t13-/m0/s1</p>**

**</body>**

**</notes>**

**<annotation>**

**<rdf:RDF xmlns:rdf="http://www.w3.org/1999/02/22-rdf-syntax-ns#" xmlns:bqmodel="http://biomodels.net/model-qualifiers/" xmlns:bqbiol="http://biomodels.net/biology-qualifiers/">**

**<rdf:Description rdf:about="_4a3f82f6-d95e-4c79-b9f8-703c029b3719">**

**<bqbiol:is>**

**<rdf:Bag>**

**<rdf:li rdf:resource="http://identifiers.org/PUBCHEM.COMPOUND/53481667"/></rdf:Bag></bqbiol:is><bqbiol:is>**

**<rdf:Bag>**

**<rdf:li rdf:resource="http://identifiers.org/HMDB/HMDB13324"/></rdf:Bag></bqbiol:is><bqbiol:is>**

**<rdf:Bag>**

**<rdf:li rdf:resource="http://identifiers.org/INCHI/InChI=1S/C15H27NO4/c1-5-6-7-8-9-10-15(19)20-13(16(2,3)4)11-12-14(17)18/h9-10,13H,5-8,11-12H2,1-4H3/b10-9+/t13-/m0/s1"/></rdf:Bag></bqbiol:is></rdf:Description></rdf:RDF>**

**</annotation>**

**</species>**

**<species id="M_sprm" constant="false" hasOnlySubstanceUnits="false" name="spermine(4+)" metaid="cd71b2b9-6cfc-4554-893c-cdbe71aa10d4" boundaryCondition="false" compartment="metaComp">**

**<notes>**

**<body xmlns="http://www.w3.org/1999/xhtml">**

**<p>FORMULA: C10H30N4</p>**

**<p>CHARGE: 0</p>**

**<p>PUBCHEM.COMPOUND: 1103</p>**

**<p>KEGG.COMPOUND: C00750</p>**

**<p>HMDB: HMDB01256</p>**

**<p>CHEBI: CHEBI:45725 || CHEBI:15746</p>**

**<p>INCHI: InChI=1S/C10H26N4/c11-5-3-9-13-7-1-2-8-14-10-4-6-12/h13-14H,1-12H2</p>**

**</body>**

**</notes>**

**<annotation>**

**<rdf:RDF xmlns:rdf="http://www.w3.org/1999/02/22-rdf-syntax-ns#" xmlns:bqmodel="http://biomodels.net/model-qualifiers/" xmlns:bqbiol="http://biomodels.net/biology-qualifiers/">**

**<rdf:Description rdf:about="cd71b2b9-6cfc-4554-893c-cdbe71aa10d4">**

**<bqbiol:is>**

**<rdf:Bag>**

**<rdf:li rdf:resource="http://identifiers.org/PUBCHEM.COMPOUND/1103"/></rdf:Bag></bqbiol:is><bqbiol:is>**

**<rdf:Bag>**

**<rdf:li rdf:resource="http://identifiers.org/KEGG.COMPOUND/C00750"/></rdf:Bag></bqbiol:is><bqbiol:is>**

**<rdf:Bag>**

**<rdf:li rdf:resource="http://identifiers.org/HMDB/HMDB01256"/></rdf:Bag></bqbiol:is><bqbiol:is>**

**<rdf:Bag>**

**<rdf:li rdf:resource="http://identifiers.org/CHEBI/CHEBI:45725"/></rdf:Bag></bqbiol:is><bqbiol:is>**

**<rdf:Bag>**

**<rdf:li rdf:resource="http://identifiers.org/CHEBI/CHEBI:15746"/></rdf:Bag></bqbiol:is><bqbiol:is>**

**<rdf:Bag>**

**<rdf:li rdf:resource="http://identifiers.org/INCHI/InChI=1S/C10H26N4/c11-5-3-9-13-7-1-2-8-14-10-4-6-12/h13-14H,1-12H2"/></rdf:Bag></bqbiol:is></rdf:Description></rdf:RDF>**

**</annotation>**

**</species>**

**<species id="M_ttdcrn" constant="false" hasOnlySubstanceUnits="false" name="tetradecanoyl carnitine" metaid="ec2a4423-de83-4398-9654-19af250a3b58" boundaryCondition="false" compartment="metaComp">**

**<notes>**

**<body xmlns="http://www.w3.org/1999/xhtml">**

**<p>FORMULA: C21H41NO4</p>**

**<p>CHARGE: 0</p>**

**<p>PUBCHEM.COMPOUND: 3413344 || 53477791</p>**

**<p>HMDB: HMDB05066</p>**

**<p>INCHI: InChI=1/C21H41NO4/c1-5-6-7-8-9-10-11-12-13-14-15-16-21(25)26-19(17-20(23)24)18-22(2,3)4/h19H,5-18H2,1-4H3/t19-/m1/s1 || InChI=1S/C21H41NO4/c1-5-6-7-8-9-10-11-12-13-14-15-16-21(25)26-19(17-20(23)24)18-22(2,3)4/h19H,5-18H2,1-4H3/t19-/m1/s1</p>**

**</body>**

**</notes>**

**<annotation>**

**<rdf:RDF xmlns:rdf="http://www.w3.org/1999/02/22-rdf-syntax-ns#" xmlns:bqmodel="http://biomodels.net/model-qualifiers/" xmlns:bqbiol="http://biomodels.net/biology-qualifiers/">**

**<rdf:Description rdf:about="ec2a4423-de83-4398-9654-19af250a3b58">**

**<bqbiol:is>**

**<rdf:Bag>**

**<rdf:li rdf:resource="http://identifiers.org/PUBCHEM.COMPOUND/3413344"/></rdf:Bag></bqbiol:is><bqbiol:is>**

**<rdf:Bag>**

**<rdf:li rdf:resource="http://identifiers.org/PUBCHEM.COMPOUND/53477791"/></rdf:Bag></bqbiol:is><bqbiol:is>**

**<rdf:Bag>**

**<rdf:li rdf:resource="http://identifiers.org/HMDB/HMDB05066"/></rdf:Bag></bqbiol:is><bqbiol:is>**

**<rdf:Bag>**

**<rdf:li rdf:resource="http://identifiers.org/INCHI/InChI=1/C21H41NO4/c1-5-6-7-8-9-10-11-12-13-14-15-16-21(25)26-19(17-20(23)24)18-22(2,3)4/h19H,5-18H2,1-4H3/t19-/m1/s1"/></rdf:Bag></bqbiol:is><bqbiol:is>**

**<rdf:Bag>**

**<rdf:li rdf:resource="http://identifiers.org/INCHI/InChI=1S/C21H41NO4/c1-5-6-7-8-9-10-11-12-13-14-15-16-21(25)26-19(17-20(23)24)18-22(2,3)4/h19H,5-18H2,1-4H3/t19-/m1/s1"/></rdf:Bag></bqbiol:is></rdf:Description></rdf:RDF>**

**</annotation>**

**</species>**

**<species id="M_HC01710" constant="false" hasOnlySubstanceUnits="false" name="2,5-Diamino-6-(5-triphosphoryl-3,4-trihydroxy-2-oxopentyl)- amino-4-oxopyrimidine" metaid="86001fb2-134e-43b3-b275-aa938993cf5f" boundaryCondition="false" compartment="metaComp">**

**<notes>**

**<body xmlns="http://www.w3.org/1999/xhtml">**

**<p>CHARGE: 0</p>**

**<p>PUBCHEM.COMPOUND: 8404</p>**

**<p>KEGG.COMPOUND: C06148</p>**

**<p>CHEBI: CHEBI:28003</p>**

**</body>**

**</notes>**

**<annotation>**

**<rdf:RDF xmlns:rdf="http://www.w3.org/1999/02/22-rdf-syntax-ns#" xmlns:bqmodel="http://biomodels.net/model-qualifiers/" xmlns:bqbiol="http://biomodels.net/biology-qualifiers/">**

**<rdf:Description rdf:about="_86001fb2-134e-43b3-b275-aa938993cf5f">**

**<bqbiol:is>**

**<rdf:Bag>**

**<rdf:li rdf:resource="http://identifiers.org/PUBCHEM.COMPOUND/8404"/></rdf:Bag></bqbiol:is><bqbiol:is>**

**<rdf:Bag>**

**<rdf:li rdf:resource="http://identifiers.org/KEGG.COMPOUND/C06148"/></rdf:Bag></bqbiol:is><bqbiol:is>**

**<rdf:Bag>**

**<rdf:li rdf:resource="http://identifiers.org/CHEBI/CHEBI:28003"/></rdf:Bag></bqbiol:is></rdf:Description></rdf:RDF>**

**</annotation>**

**</species>**

**<species id="M_HC01712" constant="false" hasOnlySubstanceUnits="false" name="S-Glutaryldihydrolipoamide" metaid="eb620b29-6f28-4f51-a43d-35b3065273e6" boundaryCondition="false" compartment="metaComp">**

**<notes>**

**<body xmlns="http://www.w3.org/1999/xhtml">**

**<p>FORMULA: C13H22NO4S2</p>**

**<p>CHARGE: 0</p>**

**<p>PUBCHEM.COMPOUND: 11953879</p>**

**<p>INCHIKEY: PWTIHZUSTBSVGF-UHFFFAOYSA-N</p>**

**<p>HMDB: HMDB06832</p>**

**<p>KEGG.COMPOUND: C06157</p>**

**<p>CHEBI: CHEBI:28391</p>**

**<p>INCHI: InChI=1S/C13H23NO4S2/c14-11(15)5-2-1-4-10(19)8-9-20-13(18)7-3-6-12(16)17/h10,19H,1-9H2,(H2,14,15)(H,16,17)</p>**

**</body>**

**</notes>**

**<annotation>**

**<rdf:RDF xmlns:rdf="http://www.w3.org/1999/02/22-rdf-syntax-ns#" xmlns:bqmodel="http://biomodels.net/model-qualifiers/" xmlns:bqbiol="http://biomodels.net/biology-qualifiers/">**

**<rdf:Description rdf:about="eb620b29-6f28-4f51-a43d-35b3065273e6">**

**<bqbiol:is>**

**<rdf:Bag>**

**<rdf:li rdf:resource="http://identifiers.org/PUBCHEM.COMPOUND/11953879"/></rdf:Bag></bqbiol:is><bqbiol:is>**

**<rdf:Bag>**

**<rdf:li rdf:resource="http://identifiers.org/INCHIKEY/PWTIHZUSTBSVGF-UHFFFAOYSA-N"/></rdf:Bag></bqbiol:is><bqbiol:is>**

**<rdf:Bag>**

**<rdf:li rdf:resource="http://identifiers.org/HMDB/HMDB06832"/></rdf:Bag></bqbiol:is><bqbiol:is>**

**<rdf:Bag>**

**<rdf:li rdf:resource="http://identifiers.org/KEGG.COMPOUND/C06157"/></rdf:Bag></bqbiol:is><bqbiol:is>**

**<rdf:Bag>**

**<rdf:li rdf:resource="http://identifiers.org/CHEBI/CHEBI:28391"/></rdf:Bag></bqbiol:is><bqbiol:is>**

**<rdf:Bag>**

**<rdf:li rdf:resource="http://identifiers.org/INCHI/InChI=1S/C13H23NO4S2/c14-11(15)5-2-1-4-10(19)8-9-20-13(18)7-3-6-12(16)17/h10,19H,1-9H2,(H2,14,15)(H,16,17)"/></rdf:Bag></bqbiol:is></rdf:Description></rdf:RDF>**

**</annotation>**

**</species>**

**<species id="M_dd2coa" constant="false" hasOnlySubstanceUnits="false" name="trans-dodec-2-enoyl-CoA(4-)" metaid="6b0e5200-f8e3-4e71-acd6-d0250c6def4b" boundaryCondition="false" compartment="metaComp">**

**<notes>**

**<body xmlns="http://www.w3.org/1999/xhtml">**

**<p>FORMULA: C33H52N7O17P3S</p>**

**<p>CHARGE: 0</p>**

**<p>PUBCHEM.COMPOUND: 45266564</p>**

**<p>KEGG.COMPOUND: C03221</p>**

**<p>HMDB: HMDB03712</p>**

**<p>CHEBI: CHEBI:57330 || CHEBI:15471</p>**

**<p>INCHI: InChI=1S/C33H56N7O17P3S/c1-4-5-6-7-8-9-10-11-12-13-24(42)61-17-16-35-23(41)14-15-36-31(45)28(44)33(2,3)19-54-60(51,52)57-59(49,50)53-18-22-27(56-58(46,47)48)26(43)32(55-22)40-21-39-25-29(34)37-20-38-30(25)40/h12-13,20-22,26-28,32,43-44H,4-11,14-19H2,1-3H3,(H,35,41)(H,36,45)(H,49,50)(H,51,52)(H2,34,37,38)(H2,46,47,48)/p-4/b13-12+/t22-,26-,27-,28+,32-/m1/s1</p>**

**</body>**

**</notes>**

**<annotation>**

**<rdf:RDF xmlns:rdf="http://www.w3.org/1999/02/22-rdf-syntax-ns#" xmlns:bqmodel="http://biomodels.net/model-qualifiers/" xmlns:bqbiol="http://biomodels.net/biology-qualifiers/">**

**<rdf:Description rdf:about="_6b0e5200-f8e3-4e71-acd6-d0250c6def4b">**

**<bqbiol:is>**

**<rdf:Bag>**

**<rdf:li rdf:resource="http://identifiers.org/PUBCHEM.COMPOUND/45266564"/></rdf:Bag></bqbiol:is><bqbiol:is>**

**<rdf:Bag>**

**<rdf:li rdf:resource="http://identifiers.org/KEGG.COMPOUND/C03221"/></rdf:Bag></bqbiol:is><bqbiol:is>**

**<rdf:Bag>**

**<rdf:li rdf:resource="http://identifiers.org/HMDB/HMDB03712"/></rdf:Bag></bqbiol:is><bqbiol:is>**

**<rdf:Bag>**

**<rdf:li rdf:resource="http://identifiers.org/CHEBI/CHEBI:57330"/></rdf:Bag></bqbiol:is><bqbiol:is>**

**<rdf:Bag>**

**<rdf:li rdf:resource="http://identifiers.org/CHEBI/CHEBI:15471"/></rdf:Bag></bqbiol:is><bqbiol:is>**

**<rdf:Bag>**

**<rdf:li rdf:resource="http://identifiers.org/INCHI/InChI=1S/C33H56N7O17P3S/c1-4-5-6-7-8-9-10-11-12-13-24(42)61-17-16-35-23(41)14-15-36-31(45)28(44)33(2,3)19-54-60(51,52)57-59(49,50)53-18-22-27(56-58(46,47)48)26(43)32(55-22)40-21-39-25-29(34)37-20-38-30(25)40/h12-13,20-22,26-28,32,43-44H,4-11,14-19H2,1-3H3,(H,35,41)(H,36,45)(H,49,50)(H,51,52)(H2,34,37,38)(H2,46,47,48)/p-4/b13-12+/t22-,26-,27-,28+,32-/m1/s1"/></rdf:Bag></bqbiol:is></rdf:Description></rdf:RDF>**

**</annotation>**

**</species>**

**<species id="M_34dhoxpeg" constant="false" hasOnlySubstanceUnits="false" name="3,4-Dihydroxyphenylethyleneglycol" metaid="d4576145-e523-4f5d-af2f-1cdab87b657d" boundaryCondition="false" compartment="metaComp">**

**<notes>**

**<body xmlns="http://www.w3.org/1999/xhtml">**

**<p>FORMULA: C8H10O4</p>**

**<p>CHARGE: 0</p>**

**<p>PUBCHEM.COMPOUND: 91528</p>**

**<p>INCHIKEY: MTVWFVDWRVYDOR-UHFFFAOYSA-N</p>**

**<p>KEGG.COMPOUND: C05576</p>**

**<p>HMDB: HMDB00318</p>**

**<p>CHEBI: CHEBI:1387</p>**

**<p>INCHI: InChI=1/C8H10O4/c9-4-8(12)5-1-2-6(10)7(11)3-5/h1-3,8-12H,4H2 || InChI=1S/C8H10O4/c9-4-8(12)5-1-2-6(10)7(11)3-5/h1-3,8-12H,4H2</p>**

**</body>**

**</notes>**

**<annotation>**

**<rdf:RDF xmlns:rdf="http://www.w3.org/1999/02/22-rdf-syntax-ns#" xmlns:bqmodel="http://biomodels.net/model-qualifiers/" xmlns:bqbiol="http://biomodels.net/biology-qualifiers/">**

**<rdf:Description rdf:about="d4576145-e523-4f5d-af2f-1cdab87b657d">**

**<bqbiol:is>**

**<rdf:Bag>**

**<rdf:li rdf:resource="http://identifiers.org/PUBCHEM.COMPOUND/91528"/></rdf:Bag></bqbiol:is><bqbiol:is>**

**<rdf:Bag>**

**<rdf:li rdf:resource="http://identifiers.org/INCHIKEY/MTVWFVDWRVYDOR-UHFFFAOYSA-N"/></rdf:Bag></bqbiol:is><bqbiol:is>**

**<rdf:Bag>**

**<rdf:li rdf:resource="http://identifiers.org/KEGG.COMPOUND/C05576"/></rdf:Bag></bqbiol:is><bqbiol:is>**

**<rdf:Bag>**

**<rdf:li rdf:resource="http://identifiers.org/HMDB/HMDB00318"/></rdf:Bag></bqbiol:is><bqbiol:is>**

**<rdf:Bag>**

**<rdf:li rdf:resource="http://identifiers.org/CHEBI/CHEBI:1387"/></rdf:Bag></bqbiol:is><bqbiol:is>**

**<rdf:Bag>**

**<rdf:li rdf:resource="http://identifiers.org/INCHI/InChI=1/C8H10O4/c9-4-8(12)5-1-2-6(10)7(11)3-5/h1-3,8-12H,4H2"/></rdf:Bag></bqbiol:is><bqbiol:is>**

**<rdf:Bag>**

**<rdf:li rdf:resource="http://identifiers.org/INCHI/InChI=1S/C8H10O4/c9-4-8(12)5-1-2-6(10)7(11)3-5/h1-3,8-12H,4H2"/></rdf:Bag></bqbiol:is></rdf:Description></rdf:RDF>**

**</annotation>**

**</species>**

**<species id="M_im4act" constant="false" hasOnlySubstanceUnits="false" name="imidazole-4-acetaldehyde" metaid="a2ea6319-d779-4afb-a350-005d95047750" boundaryCondition="false" compartment="metaComp">**

**<notes>**

**<body xmlns="http://www.w3.org/1999/xhtml">**

**<p>FORMULA: C5H6N2O</p>**

**<p>CHARGE: 0</p>**

**<p>PUBCHEM.COMPOUND: 150841</p>**

**<p>INCHIKEY: MQSRGWNVEZRLDK-UHFFFAOYSA-N</p>**

**<p>KEGG.COMPOUND: C05130</p>**

**<p>HMDB: HMDB03905</p>**

**<p>CHEBI: CHEBI:27398</p>**

**<p>INCHI: InChI=1S/C5H6N2O/c8-2-1-5-3-6-4-7-5/h2-4H,1H2,(H,6,7)</p>**

**</body>**

**</notes>**

**<annotation>**

**<rdf:RDF xmlns:rdf="http://www.w3.org/1999/02/22-rdf-syntax-ns#" xmlns:bqmodel="http://biomodels.net/model-qualifiers/" xmlns:bqbiol="http://biomodels.net/biology-qualifiers/">**

**<rdf:Description rdf:about="a2ea6319-d779-4afb-a350-005d95047750">**

**<bqbiol:is>**

**<rdf:Bag>**

**<rdf:li rdf:resource="http://identifiers.org/PUBCHEM.COMPOUND/150841"/></rdf:Bag></bqbiol:is><bqbiol:is>**

**<rdf:Bag>**

**<rdf:li rdf:resource="http://identifiers.org/INCHIKEY/MQSRGWNVEZRLDK-UHFFFAOYSA-N"/></rdf:Bag></bqbiol:is><bqbiol:is>**

**<rdf:Bag>**

**<rdf:li rdf:resource="http://identifiers.org/KEGG.COMPOUND/C05130"/></rdf:Bag></bqbiol:is><bqbiol:is>**

**<rdf:Bag>**

**<rdf:li rdf:resource="http://identifiers.org/HMDB/HMDB03905"/></rdf:Bag></bqbiol:is><bqbiol:is>**

**<rdf:Bag>**

**<rdf:li rdf:resource="http://identifiers.org/CHEBI/CHEBI:27398"/></rdf:Bag></bqbiol:is><bqbiol:is>**

**<rdf:Bag>**

**<rdf:li rdf:resource="http://identifiers.org/INCHI/InChI=1S/C5H6N2O/c8-2-1-5-3-6-4-7-5/h2-4H,1H2,(H,6,7)"/></rdf:Bag></bqbiol:is></rdf:Description></rdf:RDF>**

**</annotation>**

**</species>**

**<species id="M_CE5776" constant="false" hasOnlySubstanceUnits="false" name="iso-A2E(11-cis)" metaid="3330d60a-22a7-4b54-b598-f79caa9d22f3" boundaryCondition="false" compartment="metaComp">**

**<notes>**

**<body xmlns="http://www.w3.org/1999/xhtml">**

**<p>FORMULA: C42H58NO</p>**

**<p>CHARGE: 0</p>**

**</body>**

**</notes>**

**<annotation>**

**<rdf:RDF xmlns:rdf="http://www.w3.org/1999/02/22-rdf-syntax-ns#" xmlns:bqmodel="http://biomodels.net/model-qualifiers/" xmlns:bqbiol="http://biomodels.net/biology-qualifiers/">**

**<rdf:Description rdf:about="_3330d60a-22a7-4b54-b598-f79caa9d22f3"/></rdf:RDF>**

**</annotation>**

**</species>**

**<species id="M_lnlc" constant="false" hasOnlySubstanceUnits="false" name="linoleate" metaid="eebfdb2b-368e-49c5-8074-2bb019cc66b3" boundaryCondition="false" compartment="metaComp">**

**<notes>**

**<body xmlns="http://www.w3.org/1999/xhtml">**

**<p>FORMULA: C18H31O2</p>**

**<p>CHARGE: 0</p>**

**<p>PUBCHEM.COMPOUND: 5280450</p>**

**<p>INCHIKEY: OYHQOLUKZRVURQ-HZJYTTRNSA-N</p>**

**<p>KEGG.COMPOUND: C01595</p>**

**<p>HMDB: HMDB00673</p>**

**<p>CHEBI: CHEBI:30245 || CHEBI:17351</p>**

**<p>INCHI: InChI=1S/C18H32O2/c1-2-3-4-5-6-7-8-9-10-11-12-13-14-15-16-17-18(19)20/h6-7,9-10H,2-5,8,11-17H2,1H3,(H,19,20)/p-1/b7-6-,10-9- || InChI=1S/C18H32O2/c1-2-3-4-5-6-7-8-9-10-11-12-13-14-15-16-17-18(19)20/h6-7,9-10H,2-5,8,11-17H2,1H3,(H,19,20)/b7-6-,10-9-</p>**

**</body>**

**</notes>**

**<annotation>**

**<rdf:RDF xmlns:rdf="http://www.w3.org/1999/02/22-rdf-syntax-ns#" xmlns:bqmodel="http://biomodels.net/model-qualifiers/" xmlns:bqbiol="http://biomodels.net/biology-qualifiers/">**

**<rdf:Description rdf:about="eebfdb2b-368e-49c5-8074-2bb019cc66b3">**

**<bqbiol:is>**

**<rdf:Bag>**

**<rdf:li rdf:resource="http://identifiers.org/PUBCHEM.COMPOUND/5280450"/></rdf:Bag></bqbiol:is><bqbiol:is>**

**<rdf:Bag>**

**<rdf:li rdf:resource="http://identifiers.org/INCHIKEY/OYHQOLUKZRVURQ-HZJYTTRNSA-N"/></rdf:Bag></bqbiol:is><bqbiol:is>**

**<rdf:Bag>**

**<rdf:li rdf:resource="http://identifiers.org/KEGG.COMPOUND/C01595"/></rdf:Bag></bqbiol:is><bqbiol:is>**

**<rdf:Bag>**

**<rdf:li rdf:resource="http://identifiers.org/HMDB/HMDB00673"/></rdf:Bag></bqbiol:is><bqbiol:is>**

**<rdf:Bag>**

**<rdf:li rdf:resource="http://identifiers.org/CHEBI/CHEBI:30245"/></rdf:Bag></bqbiol:is><bqbiol:is>**

**<rdf:Bag>**

**<rdf:li rdf:resource="http://identifiers.org/CHEBI/CHEBI:17351"/></rdf:Bag></bqbiol:is><bqbiol:is>**

**<rdf:Bag>**

**<rdf:li rdf:resource="http://identifiers.org/INCHI/InChI=1S/C18H32O2/c1-2-3-4-5-6-7-8-9-10-11-12-13-14-15-16-17-18(19)20/h6-7,9-10H,2-5,8,11-17H2,1H3,(H,19,20)/p-1/b7-6-,10-9-"/></rdf:Bag></bqbiol:is><bqbiol:is>**

**<rdf:Bag>**

**<rdf:li rdf:resource="http://identifiers.org/INCHI/InChI=1S/C18H32O2/c1-2-3-4-5-6-7-8-9-10-11-12-13-14-15-16-17-18(19)20/h6-7,9-10H,2-5,8,11-17H2,1H3,(H,19,20)/b7-6-,10-9-"/></rdf:Bag></bqbiol:is></rdf:Description></rdf:RDF>**

**</annotation>**

**</species>**

**<species id="M_CE5775" constant="false" hasOnlySubstanceUnits="false" name="iso-A2E(9-cis)" metaid="bd6a1210-df17-4769-aaa2-0f0beeda22cc" boundaryCondition="false" compartment="metaComp">**

**<notes>**

**<body xmlns="http://www.w3.org/1999/xhtml">**

**<p>FORMULA: C42H58NO</p>**

**<p>CHARGE: 0</p>**

**</body>**

**</notes>**

**<annotation>**

**<rdf:RDF xmlns:rdf="http://www.w3.org/1999/02/22-rdf-syntax-ns#" xmlns:bqmodel="http://biomodels.net/model-qualifiers/" xmlns:bqbiol="http://biomodels.net/biology-qualifiers/">**

**<rdf:Description rdf:about="bd6a1210-df17-4769-aaa2-0f0beeda22cc"/></rdf:RDF>**

**</annotation>**

**</species>**

**<species id="M_xol7aone" constant="false" hasOnlySubstanceUnits="false" name="7alpha-hydroxycholest-4-en-3-one" metaid="6c669dd2-aa44-4c31-a59d-c5f5fddabde7" boundaryCondition="false" compartment="metaComp">**

**<notes>**

**<body xmlns="http://www.w3.org/1999/xhtml">**

**<p>FORMULA: C27H44O2</p>**

**<p>CHARGE: 0</p>**

**<p>PUBCHEM.COMPOUND: 123743</p>**

**<p>INCHIKEY: IOIZWEJGGCZDOL-RQDYSCIWSA-N</p>**

**<p>KEGG.COMPOUND: C05455</p>**

**<p>HMDB: HMDB01993</p>**

**<p>CHEBI: CHEBI:17899</p>**

**<p>INCHI: InChI=1S/C27H44O2/c1-17(2)7-6-8-18(3)21-9-10-22-25-23(12-14-27(21,22)5)26(4)13-11-20(28)15-19(26)16-24(25)29/h15,17-18,21-25,29H,6-14,16H2,1-5H3/t18-,21-,22+,23+,24-,25+,26+,27-/m1/s1</p>**

**</body>**

**</notes>**

**<annotation>**

**<rdf:RDF xmlns:rdf="http://www.w3.org/1999/02/22-rdf-syntax-ns#" xmlns:bqmodel="http://biomodels.net/model-qualifiers/" xmlns:bqbiol="http://biomodels.net/biology-qualifiers/">**

**<rdf:Description rdf:about="_6c669dd2-aa44-4c31-a59d-c5f5fddabde7">**

**<bqbiol:is>**

**<rdf:Bag>**

**<rdf:li rdf:resource="http://identifiers.org/PUBCHEM.COMPOUND/123743"/></rdf:Bag></bqbiol:is><bqbiol:is>**

**<rdf:Bag>**

**<rdf:li rdf:resource="http://identifiers.org/INCHIKEY/IOIZWEJGGCZDOL-RQDYSCIWSA-N"/></rdf:Bag></bqbiol:is><bqbiol:is>**

**<rdf:Bag>**

**<rdf:li rdf:resource="http://identifiers.org/KEGG.COMPOUND/C05455"/></rdf:Bag></bqbiol:is><bqbiol:is>**

**<rdf:Bag>**

**<rdf:li rdf:resource="http://identifiers.org/HMDB/HMDB01993"/></rdf:Bag></bqbiol:is><bqbiol:is>**

**<rdf:Bag>**

**<rdf:li rdf:resource="http://identifiers.org/CHEBI/CHEBI:17899"/></rdf:Bag></bqbiol:is><bqbiol:is>**

**<rdf:Bag>**

**<rdf:li rdf:resource="http://identifiers.org/INCHI/InChI=1S/C27H44O2/c1-17(2)7-6-8-18(3)21-9-10-22-25-23(12-14-27(21,22)5)26(4)13-11-20(28)15-19(26)16-24(25)29/h15,17-18,21-25,29H,6-14,16H2,1-5H3/t18-,21-,22+,23+,24-,25+,26+,27-/m1/s1"/></rdf:Bag></bqbiol:is></rdf:Description></rdf:RDF>**

**</annotation>**

**</species>**

**<species id="M_3ddcrn" constant="false" hasOnlySubstanceUnits="false" name="3-hydroxydodecanoylcarnitine" metaid="05ac27de-9e65-47b1-816d-9d19597d4861" boundaryCondition="false" compartment="metaComp">**

**<notes>**

**<body xmlns="http://www.w3.org/1999/xhtml">**

**<p>FORMULA: C19H37NO5</p>**

**<p>CHARGE: 0</p>**

**</body>**

**</notes>**

**<annotation>**

**<rdf:RDF xmlns:rdf="http://www.w3.org/1999/02/22-rdf-syntax-ns#" xmlns:bqmodel="http://biomodels.net/model-qualifiers/" xmlns:bqbiol="http://biomodels.net/biology-qualifiers/">**

**<rdf:Description rdf:about="_05ac27de-9e65-47b1-816d-9d19597d4861"/></rdf:RDF>**

**</annotation>**

**</species>**

**<species id="M_for" constant="false" hasOnlySubstanceUnits="false" name="Formate" metaid="f873205f-8d40-49c2-aac3-ec29049d6586" boundaryCondition="false" compartment="metaComp">**

**<notes>**

**<body xmlns="http://www.w3.org/1999/xhtml">**

**<p>FORMULA: CH1O2</p>**

**<p>CHARGE: 0</p>**

**<p>PUBCHEM.COMPOUND: 284</p>**

**<p>INCHIKEY: BDAGIHXWWSANSR-UHFFFAOYSA-N</p>**

**<p>KEGG.COMPOUND: C00058</p>**

**<p>HMDB: HMDB00142</p>**

**<p>CHEBI: CHEBI:15740 || CHEBI:30751</p>**

**<p>INCHI: InChI=1S/CH2O2/c2-1-3/h1H,(H,2,3) || InChI=1S/CH2O2/c2-1-3/h1H,(H,2,3)/p-1</p>**

**</body>**

**</notes>**

**<annotation>**

**<rdf:RDF xmlns:rdf="http://www.w3.org/1999/02/22-rdf-syntax-ns#" xmlns:bqmodel="http://biomodels.net/model-qualifiers/" xmlns:bqbiol="http://biomodels.net/biology-qualifiers/">**

**<rdf:Description rdf:about="f873205f-8d40-49c2-aac3-ec29049d6586">**

**<bqbiol:is>**

**<rdf:Bag>**

**<rdf:li rdf:resource="http://identifiers.org/PUBCHEM.COMPOUND/284"/></rdf:Bag></bqbiol:is><bqbiol:is>**

**<rdf:Bag>**

**<rdf:li rdf:resource="http://identifiers.org/INCHIKEY/BDAGIHXWWSANSR-UHFFFAOYSA-N"/></rdf:Bag></bqbiol:is><bqbiol:is>**

**<rdf:Bag>**

**<rdf:li rdf:resource="http://identifiers.org/KEGG.COMPOUND/C00058"/></rdf:Bag></bqbiol:is><bqbiol:is>**

**<rdf:Bag>**

**<rdf:li rdf:resource="http://identifiers.org/HMDB/HMDB00142"/></rdf:Bag></bqbiol:is><bqbiol:is>**

**<rdf:Bag>**

**<rdf:li rdf:resource="http://identifiers.org/CHEBI/CHEBI:15740"/></rdf:Bag></bqbiol:is><bqbiol:is>**

**<rdf:Bag>**

**<rdf:li rdf:resource="http://identifiers.org/CHEBI/CHEBI:30751"/></rdf:Bag></bqbiol:is><bqbiol:is>**

**<rdf:Bag>**

**<rdf:li rdf:resource="http://identifiers.org/INCHI/InChI=1S/CH2O2/c2-1-3/h1H,(H,2,3)"/></rdf:Bag></bqbiol:is><bqbiol:is>**

**<rdf:Bag>**

**<rdf:li rdf:resource="http://identifiers.org/INCHI/InChI=1S/CH2O2/c2-1-3/h1H,(H,2,3)/p-1"/></rdf:Bag></bqbiol:is></rdf:Description></rdf:RDF>**

**</annotation>**

**</species>**

**<species id="M_CE7109" constant="false" hasOnlySubstanceUnits="false" name="5(S),6(S)-epoxy-15(R)-HEPE" metaid="61eb6e95-c68b-4fee-a106-6f15fbb4a764" boundaryCondition="false" compartment="metaComp">**

**<notes>**

**<body xmlns="http://www.w3.org/1999/xhtml">**

**<p>FORMULA: C20H27O4</p>**

**<p>CHARGE: 0</p>**

**</body>**

**</notes>**

**<annotation>**

**<rdf:RDF xmlns:rdf="http://www.w3.org/1999/02/22-rdf-syntax-ns#" xmlns:bqmodel="http://biomodels.net/model-qualifiers/" xmlns:bqbiol="http://biomodels.net/biology-qualifiers/">**

**<rdf:Description rdf:about="_61eb6e95-c68b-4fee-a106-6f15fbb4a764"/></rdf:RDF>**

**</annotation>**

**</species>**

**<species id="M_fol" constant="false" hasOnlySubstanceUnits="false" name="Folate" metaid="f1b19e5a-3189-4d52-bd56-959d5e4cf873" boundaryCondition="false" compartment="metaComp">**

**<notes>**

**<body xmlns="http://www.w3.org/1999/xhtml">**

**<p>FORMULA: C19H18N7O6</p>**

**<p>CHARGE: 0</p>**

**<p>PUBCHEM.COMPOUND: 6037</p>**

**<p>INCHIKEY: OVBPIULPVIDEAO-LBPRGKRZSA-N</p>**

**<p>KEGG.COMPOUND: C00504</p>**

**<p>HMDB: HMDB00121</p>**

**<p>CHEBI: CHEBI:27470</p>**

**<p>INCHI: InChI=1S/C19H19N7O6/c20-19-25-15-14(17(30)26-19)23-11(8-22-15)7-21-10-3-1-9(2-4-10)16(29)24-12(18(31)32)5-6-13(27)28/h1-4,8,12,21H,5-7H2,(H,24,29)(H,27,28)(H,31,32)(H3,20,22,25,26,30)/t12-/m0/s1</p>**

**</body>**

**</notes>**

**<annotation>**

**<rdf:RDF xmlns:rdf="http://www.w3.org/1999/02/22-rdf-syntax-ns#" xmlns:bqmodel="http://biomodels.net/model-qualifiers/" xmlns:bqbiol="http://biomodels.net/biology-qualifiers/">**

**<rdf:Description rdf:about="f1b19e5a-3189-4d52-bd56-959d5e4cf873">**

**<bqbiol:is>**

**<rdf:Bag>**

**<rdf:li rdf:resource="http://identifiers.org/PUBCHEM.COMPOUND/6037"/></rdf:Bag></bqbiol:is><bqbiol:is>**

**<rdf:Bag>**

**<rdf:li rdf:resource="http://identifiers.org/INCHIKEY/OVBPIULPVIDEAO-LBPRGKRZSA-N"/></rdf:Bag></bqbiol:is><bqbiol:is>**

**<rdf:Bag>**

**<rdf:li rdf:resource="http://identifiers.org/KEGG.COMPOUND/C00504"/></rdf:Bag></bqbiol:is><bqbiol:is>**

**<rdf:Bag>**

**<rdf:li rdf:resource="http://identifiers.org/HMDB/HMDB00121"/></rdf:Bag></bqbiol:is><bqbiol:is>**

**<rdf:Bag>**

**<rdf:li rdf:resource="http://identifiers.org/CHEBI/CHEBI:27470"/></rdf:Bag></bqbiol:is><bqbiol:is>**

**<rdf:Bag>**

**<rdf:li rdf:resource="http://identifiers.org/INCHI/InChI=1S/C19H19N7O6/c20-19-25-15-14(17(30)26-19)23-11(8-22-15)7-21-10-3-1-9(2-4-10)16(29)24-12(18(31)32)5-6-13(27)28/h1-4,8,12,21H,5-7H2,(H,24,29)(H,27,28)(H,31,32)(H3,20,22,25,26,30)/t12-/m0/s1"/></rdf:Bag></bqbiol:is></rdf:Description></rdf:RDF>**

**</annotation>**

**</species>**

**<species id="M_air" constant="false" hasOnlySubstanceUnits="false" name="5-amino-1-(5-phospho-D-ribosyl)imidazole" metaid="99cf3dba-bba0-4e52-8789-8cac53fb2a82" boundaryCondition="false" compartment="metaComp">**

**<notes>**

**<body xmlns="http://www.w3.org/1999/xhtml">**

**<p>FORMULA: C8H12N3O7P</p>**

**<p>CHARGE: 0</p>**

**<p>PUBCHEM.COMPOUND: 161500</p>**

**<p>KEGG.COMPOUND: C03373</p>**

**<p>HMDB: HMDB01235</p>**

**<p>CHEBI: CHEBI:28843 || CHEBI:58592</p>**

**<p>INCHI: InChI=1S/C8H14N3O7P/c9-5-1-10-3-11(5)8-7(13)6(12)4(18-8)2-17-19(14,15)16/h1,3-4,6-8,12-13H,2,9H2,(H2,14,15,16)/t4-,6-,7-,8-/m1/s1 || InChI=1S/C8H14N3O7P/c9-5-1-10-3-11(5)8-7(13)6(12)4(18-8)2-17-19(14,15)16/h1,3-4,6-8,12-13H,2,9H2,(H2,14,15,16)/p-1/t4-,6-,7-,8?/m1/s1</p>**

**</body>**

**</notes>**

**<annotation>**

**<rdf:RDF xmlns:rdf="http://www.w3.org/1999/02/22-rdf-syntax-ns#" xmlns:bqmodel="http://biomodels.net/model-qualifiers/" xmlns:bqbiol="http://biomodels.net/biology-qualifiers/">**

**<rdf:Description rdf:about="_99cf3dba-bba0-4e52-8789-8cac53fb2a82">**

**<bqbiol:is>**

**<rdf:Bag>**

**<rdf:li rdf:resource="http://identifiers.org/PUBCHEM.COMPOUND/161500"/></rdf:Bag></bqbiol:is><bqbiol:is>**

**<rdf:Bag>**

**<rdf:li rdf:resource="http://identifiers.org/KEGG.COMPOUND/C03373"/></rdf:Bag></bqbiol:is><bqbiol:is>**

**<rdf:Bag>**

**<rdf:li rdf:resource="http://identifiers.org/HMDB/HMDB01235"/></rdf:Bag></bqbiol:is><bqbiol:is>**

**<rdf:Bag>**

**<rdf:li rdf:resource="http://identifiers.org/CHEBI/CHEBI:28843"/></rdf:Bag></bqbiol:is><bqbiol:is>**

**<rdf:Bag>**

**<rdf:li rdf:resource="http://identifiers.org/CHEBI/CHEBI:58592"/></rdf:Bag></bqbiol:is><bqbiol:is>**

**<rdf:Bag>**

**<rdf:li rdf:resource="http://identifiers.org/INCHI/InChI=1S/C8H14N3O7P/c9-5-1-10-3-11(5)8-7(13)6(12)4(18-8)2-17-19(14,15)16/h1,3-4,6-8,12-13H,2,9H2,(H2,14,15,16)/t4-,6-,7-,8-/m1/s1"/></rdf:Bag></bqbiol:is><bqbiol:is>**

**<rdf:Bag>**

**<rdf:li rdf:resource="http://identifiers.org/INCHI/InChI=1S/C8H14N3O7P/c9-5-1-10-3-11(5)8-7(13)6(12)4(18-8)2-17-19(14,15)16/h1,3-4,6-8,12-13H,2,9H2,(H2,14,15,16)/p-1/t4-,6-,7-,8?/m1/s1"/></rdf:Bag></bqbiol:is></rdf:Description></rdf:RDF>**

**</annotation>**

**</species>**

**<species id="M_CE7101" constant="false" hasOnlySubstanceUnits="false" name="8alpha-hydroxy-gama-tocopherone" metaid="bc9246bc-e323-40c4-8353-a8790bcc0e0e" boundaryCondition="false" compartment="metaComp">**

**<notes>**

**<body xmlns="http://www.w3.org/1999/xhtml">**

**<p>FORMULA: C28H48O3</p>**

**<p>CHARGE: 0</p>**

**<p>INCHI: InChI=1/C28H48O3/c1-20(2)11-8-12-21(3)13-9-14-22(4)15-10-17-27(7)18-16-25-19-26(29)23(5)24(6)28(25,30)31-27/h19-22,30H,8-18H2,1-7H3/t21-,22-,27?,28?/m0/s1</p>**

**</body>**

**</notes>**

**<annotation>**

**<rdf:RDF xmlns:rdf="http://www.w3.org/1999/02/22-rdf-syntax-ns#" xmlns:bqmodel="http://biomodels.net/model-qualifiers/" xmlns:bqbiol="http://biomodels.net/biology-qualifiers/">**

**<rdf:Description rdf:about="bc9246bc-e323-40c4-8353-a8790bcc0e0e">**

**<bqbiol:is>**

**<rdf:Bag>**

**<rdf:li rdf:resource="http://identifiers.org/INCHI/InChI=1/C28H48O3/c1-20(2)11-8-12-21(3)13-9-14-22(4)15-10-17-27(7)18-16-25-19-26(29)23(5)24(6)28(25,30)31-27/h19-22,30H,8-18H2,1-7H3/t21-,22-,27?,28?/m0/s1"/></rdf:Bag></bqbiol:is></rdf:Description></rdf:RDF>**

**</annotation>**

**</species>**

**<species id="M_gdpddman" constant="false" hasOnlySubstanceUnits="false" name="GDP-4-dehydro-6-deoxy-alpha-D-mannose(2-)" metaid="b769a599-eee2-444e-821d-0c3c3c5aa053" boundaryCondition="false" compartment="metaComp">**

**<notes>**

**<body xmlns="http://www.w3.org/1999/xhtml">**

**<p>FORMULA: C16H21N5O15P2</p>**

**<p>CHARGE: 0</p>**

**<p>PUBCHEM.COMPOUND: 439446</p>**

**<p>KEGG.COMPOUND: C01222</p>**

**<p>HMDB: HMDB01346</p>**

**<p>CHEBI: CHEBI:16955 || CHEBI:57964</p>**

**<p>INCHI: InChI=1S/C16H23N5O15P2/c1-4-7(22)9(24)11(26)15(33-4)35-38(30,31)36-37(28,29)32-2-5-8(23)10(25)14(34-5)21-3-18-6-12(21)19-16(17)20-13(6)27/h3-5,8-11,14-15,23-26H,2H2,1H3,(H,28,29)(H,30,31)(H3,17,19,20,27)/t4-,5-,8-,9+,10-,11+,14-,15-/m1/s1 || InChI=1S/C16H23N5O15P2/c1-4-7(22)9(24)11(26)15(33-4)35-38(30,31)36-37(28,29)32-2-5-8(23)10(25)14(34-5)21-3-18-6-12(21)19-16(17)20-13(6)27/h3-5,8-11,14-15,23-26H,2H2,1H3,(H,28,29)(H,30,31)(H3,17,19,20,27)/p-2/t4-,5-,8-,9+,10-,11+,14-,15-/m1/s1</p>**

**</body>**

**</notes>**

**<annotation>**

**<rdf:RDF xmlns:rdf="http://www.w3.org/1999/02/22-rdf-syntax-ns#" xmlns:bqmodel="http://biomodels.net/model-qualifiers/" xmlns:bqbiol="http://biomodels.net/biology-qualifiers/">**

**<rdf:Description rdf:about="b769a599-eee2-444e-821d-0c3c3c5aa053">**

**<bqbiol:is>**

**<rdf:Bag>**

**<rdf:li rdf:resource="http://identifiers.org/PUBCHEM.COMPOUND/439446"/></rdf:Bag></bqbiol:is><bqbiol:is>**

**<rdf:Bag>**

**<rdf:li rdf:resource="http://identifiers.org/KEGG.COMPOUND/C01222"/></rdf:Bag></bqbiol:is><bqbiol:is>**

**<rdf:Bag>**

**<rdf:li rdf:resource="http://identifiers.org/HMDB/HMDB01346"/></rdf:Bag></bqbiol:is><bqbiol:is>**

**<rdf:Bag>**

**<rdf:li rdf:resource="http://identifiers.org/CHEBI/CHEBI:16955"/></rdf:Bag></bqbiol:is><bqbiol:is>**

**<rdf:Bag>**

**<rdf:li rdf:resource="http://identifiers.org/CHEBI/CHEBI:57964"/></rdf:Bag></bqbiol:is><bqbiol:is>**

**<rdf:Bag>**

**<rdf:li rdf:resource="http://identifiers.org/INCHI/InChI=1S/C16H23N5O15P2/c1-4-7(22)9(24)11(26)15(33-4)35-38(30,31)36-37(28,29)32-2-5-8(23)10(25)14(34-5)21-3-18-6-12(21)19-16(17)20-13(6)27/h3-5,8-11,14-15,23-26H,2H2,1H3,(H,28,29)(H,30,31)(H3,17,19,20,27)/t4-,5-,8-,9+,10-,11+,14-,15-/m1/s1"/></rdf:Bag></bqbiol:is><bqbiol:is>**

**<rdf:Bag>**

**<rdf:li rdf:resource="http://identifiers.org/INCHI/InChI=1S/C16H23N5O15P2/c1-4-7(22)9(24)11(26)15(33-4)35-38(30,31)36-37(28,29)32-2-5-8(23)10(25)14(34-5)21-3-18-6-12(21)19-16(17)20-13(6)27/h3-5,8-11,14-15,23-26H,2H2,1H3,(H,28,29)(H,30,31)(H3,17,19,20,27)/p-2/t4-,5-,8-,9+,10-,11+,14-,15-/m1/s1"/></rdf:Bag></bqbiol:is></rdf:Description></rdf:RDF>**

**</annotation>**

**</species>**

**<species id="M_CE3554" constant="false" hasOnlySubstanceUnits="false" name="20-trihydroxy-leukotriene-B4" metaid="cbe4b68d-1189-4529-a171-4d28b7367c9e" boundaryCondition="false" compartment="metaComp">**

**<notes>**

**<body xmlns="http://www.w3.org/1999/xhtml">**

**<p>FORMULA: C20H31O7</p>**

**<p>CHARGE: 0</p>**

**<p>PUBCHEM.COMPOUND: 53481513</p>**

**<p>INCHIKEY: UZWWTCBNUQJEPB-WLMOLUCVSA-N</p>**

**<p>HMDB: HMDB12643</p>**

**<p>INCHI: InChI=1S/C20H32O7/c21-17(11-6-2-1-5-9-16-20(25,26)27)12-7-3-4-8-13-18(22)14-10-15-19(23)24/h2-4,6-8,12-13,17-18,21-22,25-27H,1,5,9-11,14-16H2,(H,23,24)/b4-3+,6-2-,12-7+,13-8-/t17-,18-/m0/s1</p>**

**</body>**

**</notes>**

**<annotation>**

**<rdf:RDF xmlns:rdf="http://www.w3.org/1999/02/22-rdf-syntax-ns#" xmlns:bqmodel="http://biomodels.net/model-qualifiers/" xmlns:bqbiol="http://biomodels.net/biology-qualifiers/">**

**<rdf:Description rdf:about="cbe4b68d-1189-4529-a171-4d28b7367c9e">**

**<bqbiol:is>**

**<rdf:Bag>**

**<rdf:li rdf:resource="http://identifiers.org/PUBCHEM.COMPOUND/53481513"/></rdf:Bag></bqbiol:is><bqbiol:is>**

**<rdf:Bag>**

**<rdf:li rdf:resource="http://identifiers.org/INCHIKEY/UZWWTCBNUQJEPB-WLMOLUCVSA-N"/></rdf:Bag></bqbiol:is><bqbiol:is>**

**<rdf:Bag>**

**<rdf:li rdf:resource="http://identifiers.org/HMDB/HMDB12643"/></rdf:Bag></bqbiol:is><bqbiol:is>**

**<rdf:Bag>**

**<rdf:li rdf:resource="http://identifiers.org/INCHI/InChI=1S/C20H32O7/c21-17(11-6-2-1-5-9-16-20(25,26)27)12-7-3-4-8-13-18(22)14-10-15-19(23)24/h2-4,6-8,12-13,17-18,21-22,25-27H,1,5,9-11,14-16H2,(H,23,24)/b4-3+,6-2-,12-7+,13-8-/t17-,18-/m0/s1"/></rdf:Bag></bqbiol:is></rdf:Description></rdf:RDF>**

**</annotation>**

**</species>**

**<species id="M_HC01700" constant="false" hasOnlySubstanceUnits="false" name="gamma-Glutamyl-3-aminopropiononitrile" metaid="3887fc43-9e5b-4640-b8de-5ce67ffc6d72" boundaryCondition="false" compartment="metaComp">**

**<notes>**

**<body xmlns="http://www.w3.org/1999/xhtml">**

**<p>CHARGE: 0</p>**

**<p>PUBCHEM.COMPOUND: 8376</p>**

**<p>KEGG.COMPOUND: C06114</p>**

**<p>CHEBI: CHEBI:28092</p>**

**</body>**

**</notes>**

**<annotation>**

**<rdf:RDF xmlns:rdf="http://www.w3.org/1999/02/22-rdf-syntax-ns#" xmlns:bqmodel="http://biomodels.net/model-qualifiers/" xmlns:bqbiol="http://biomodels.net/biology-qualifiers/">**

**<rdf:Description rdf:about="_3887fc43-9e5b-4640-b8de-5ce67ffc6d72">**

**<bqbiol:is>**

**<rdf:Bag>**

**<rdf:li rdf:resource="http://identifiers.org/PUBCHEM.COMPOUND/8376"/></rdf:Bag></bqbiol:is><bqbiol:is>**

**<rdf:Bag>**

**<rdf:li rdf:resource="http://identifiers.org/KEGG.COMPOUND/C06114"/></rdf:Bag></bqbiol:is><bqbiol:is>**

**<rdf:Bag>**

**<rdf:li rdf:resource="http://identifiers.org/CHEBI/CHEBI:28092"/></rdf:Bag></bqbiol:is></rdf:Description></rdf:RDF>**

**</annotation>**

**</species>**

**<species id="M_nm4masn" constant="false" hasOnlySubstanceUnits="false" name="(N-acetyl-D-glucosaminyl-(alpha-D-mannosyl)4-beta-D-mannosyl-diacetylchitobiosyl)-L-asparagine (protein)" metaid="e7b962d2-eb58-4a62-8165-787272c98174" boundaryCondition="false" compartment="metaComp">**

**<notes>**

**<body xmlns="http://www.w3.org/1999/xhtml">**

**<p>FORMULA: C54H90N3O40X</p>**

**<p>CHARGE: 0</p>**

**<p>KEGG.COMPOUND: C05882</p>**

**</body>**

**</notes>**

**<annotation>**

**<rdf:RDF xmlns:rdf="http://www.w3.org/1999/02/22-rdf-syntax-ns#" xmlns:bqmodel="http://biomodels.net/model-qualifiers/" xmlns:bqbiol="http://biomodels.net/biology-qualifiers/">**

**<rdf:Description rdf:about="e7b962d2-eb58-4a62-8165-787272c98174">**

**<bqbiol:is>**

**<rdf:Bag>**

**<rdf:li rdf:resource="http://identifiers.org/KEGG.COMPOUND/C05882"/></rdf:Bag></bqbiol:is></rdf:Description></rdf:RDF>**

**</annotation>**

**</species>**

**<species id="M_glcur1p" constant="false" hasOnlySubstanceUnits="false" name="D-Glucuronate 1-phosphate" metaid="4244fb18-5c47-4091-8f42-abbc88c103b5" boundaryCondition="false" compartment="metaComp">**

**<notes>**

**<body xmlns="http://www.w3.org/1999/xhtml">**

**<p>FORMULA: C6H8O10P</p>**

**<p>CHARGE: 0</p>**

**<p>PUBCHEM.COMPOUND: 440650</p>**

**<p>INCHIKEY: AIQDYKMWENWVQJ-QIUUJYRFSA-N</p>**

**<p>KEGG.COMPOUND: C05385</p>**

**<p>HMDB: HMDB03976</p>**

**<p>CHEBI: CHEBI:35145 || CHEBI:28547 || CHEBI:16787</p>**

**<p>INCHI: InChI=1S/C6H11O10P/c7-1-2(8)4(5(10)11)15-6(3(1)9)16-17(12,13)14/h1-4,6-9H,(H,10,11)(H2,12,13,14)/t1-,2-,3+,4-,6+/m0/s1 || InChI=1S/C6H11O10P/c7-1-2(8)4(5(10)11)15-6(3(1)9)16-17(12,13)14/h1-4,6-9H,(H,10,11)(H2,12,13,14)/t1-,2-,3+,4-,6?/m0/s1 || InChI=1S/C6H11O10P/c7-1-2(8)4(5(10)11)15-6(3(1)9)16-17(12,13)14/h1-4,6-9H,(H,10,11)(H2,12,13,14)/p-1/t1-,2-,3+,4-,6?/m0/s1</p>**

**</body>**

**</notes>**

**<annotation>**

**<rdf:RDF xmlns:rdf="http://www.w3.org/1999/02/22-rdf-syntax-ns#" xmlns:bqmodel="http://biomodels.net/model-qualifiers/" xmlns:bqbiol="http://biomodels.net/biology-qualifiers/">**

**<rdf:Description rdf:about="_4244fb18-5c47-4091-8f42-abbc88c103b5">**

**<bqbiol:is>**

**<rdf:Bag>**

**<rdf:li rdf:resource="http://identifiers.org/PUBCHEM.COMPOUND/440650"/></rdf:Bag></bqbiol:is><bqbiol:is>**

**<rdf:Bag>**

**<rdf:li rdf:resource="http://identifiers.org/INCHIKEY/AIQDYKMWENWVQJ-QIUUJYRFSA-N"/></rdf:Bag></bqbiol:is><bqbiol:is>**

**<rdf:Bag>**

**<rdf:li rdf:resource="http://identifiers.org/KEGG.COMPOUND/C05385"/></rdf:Bag></bqbiol:is><bqbiol:is>**

**<rdf:Bag>**

**<rdf:li rdf:resource="http://identifiers.org/HMDB/HMDB03976"/></rdf:Bag></bqbiol:is><bqbiol:is>**

**<rdf:Bag>**

**<rdf:li rdf:resource="http://identifiers.org/CHEBI/CHEBI:35145"/></rdf:Bag></bqbiol:is><bqbiol:is>**

**<rdf:Bag>**

**<rdf:li rdf:resource="http://identifiers.org/CHEBI/CHEBI:28547"/></rdf:Bag></bqbiol:is><bqbiol:is>**

**<rdf:Bag>**

**<rdf:li rdf:resource="http://identifiers.org/CHEBI/CHEBI:16787"/></rdf:Bag></bqbiol:is><bqbiol:is>**

**<rdf:Bag>**

**<rdf:li rdf:resource="http://identifiers.org/INCHI/InChI=1S/C6H11O10P/c7-1-2(8)4(5(10)11)15-6(3(1)9)16-17(12,13)14/h1-4,6-9H,(H,10,11)(H2,12,13,14)/t1-,2-,3+,4-,6+/m0/s1"/></rdf:Bag></bqbiol:is><bqbiol:is>**

**<rdf:Bag>**

**<rdf:li rdf:resource="http://identifiers.org/INCHI/InChI=1S/C6H11O10P/c7-1-2(8)4(5(10)11)15-6(3(1)9)16-17(12,13)14/h1-4,6-9H,(H,10,11)(H2,12,13,14)/t1-,2-,3+,4-,6?/m0/s1"/></rdf:Bag></bqbiol:is><bqbiol:is>**

**<rdf:Bag>**

**<rdf:li rdf:resource="http://identifiers.org/INCHI/InChI=1S/C6H11O10P/c7-1-2(8)4(5(10)11)15-6(3(1)9)16-17(12,13)14/h1-4,6-9H,(H,10,11)(H2,12,13,14)/p-1/t1-,2-,3+,4-,6?/m0/s1"/></rdf:Bag></bqbiol:is></rdf:Description></rdf:RDF>**

**</annotation>**

**</species>**

**<species id="M_sebacid" constant="false" hasOnlySubstanceUnits="false" name="sebacicacid" metaid="6a9a30ff-5d53-4ea9-a05e-81e7c5990769" boundaryCondition="false" compartment="metaComp">**

**<notes>**

**<body xmlns="http://www.w3.org/1999/xhtml">**

**<p>FORMULA: C10H16O4</p>**

**<p>CHARGE: 0</p>**

**<p>PUBCHEM.COMPOUND: 5192</p>**

**<p>KEGG.COMPOUND: C08277</p>**

**<p>HMDB: HMDB00792</p>**

**<p>CHEBI: CHEBI:41865</p>**

**<p>INCHI: InChI=1S/C10H18O4/c11-9(12)7-5-3-1-2-4-6-8-10(13)14/h1-8H2,(H,11,12)(H,13,14)</p>**

**</body>**

**</notes>**

**<annotation>**

**<rdf:RDF xmlns:rdf="http://www.w3.org/1999/02/22-rdf-syntax-ns#" xmlns:bqmodel="http://biomodels.net/model-qualifiers/" xmlns:bqbiol="http://biomodels.net/biology-qualifiers/">**

**<rdf:Description rdf:about="_6a9a30ff-5d53-4ea9-a05e-81e7c5990769">**

**<bqbiol:is>**

**<rdf:Bag>**

**<rdf:li rdf:resource="http://identifiers.org/PUBCHEM.COMPOUND/5192"/></rdf:Bag></bqbiol:is><bqbiol:is>**

**<rdf:Bag>**

**<rdf:li rdf:resource="http://identifiers.org/KEGG.COMPOUND/C08277"/></rdf:Bag></bqbiol:is><bqbiol:is>**

**<rdf:Bag>**

**<rdf:li rdf:resource="http://identifiers.org/HMDB/HMDB00792"/></rdf:Bag></bqbiol:is><bqbiol:is>**

**<rdf:Bag>**

**<rdf:li rdf:resource="http://identifiers.org/CHEBI/CHEBI:41865"/></rdf:Bag></bqbiol:is><bqbiol:is>**

**<rdf:Bag>**

**<rdf:li rdf:resource="http://identifiers.org/INCHI/InChI=1S/C10H18O4/c11-9(12)7-5-3-1-2-4-6-8-10(13)14/h1-8H2,(H,11,12)(H,13,14)"/></rdf:Bag></bqbiol:is></rdf:Description></rdf:RDF>**

**</annotation>**

**</species>**

**<species id="M_CE6031" constant="false" hasOnlySubstanceUnits="false" name="androsterone sulfate" metaid="c829d49f-38c3-4fe2-838b-837d54f83d6d" boundaryCondition="false" compartment="metaComp">**

**<notes>**

**<body xmlns="http://www.w3.org/1999/xhtml">**

**<p>FORMULA: C19H29O5S</p>**

**<p>CHARGE: 0</p>**

**<p>PUBCHEM.COMPOUND: 159663</p>**

**<p>INCHIKEY: ZMITXKRGXGRMKS-HLUDHZFRSA-N</p>**

**<p>HMDB: HMDB02759 || HMDB01032</p>**

**<p>KEGG.COMPOUND: C04555</p>**

**<p>CHEBI: CHEBI:16814</p>**

**<p>INCHI: InChI=1S/C19H30O5S/c1-18-9-7-13(24-25(21,22)23)11-12(18)3-4-14-15-5-6-17(20)19(15,2)10-8-16(14)18/h12-16H,3-11H2,1-2H3,(H,21,22,23)/t12-,13+,14-,15-,16-,18-,19-/m0/s1 || InChI=1/C19H30O5S/c1-18-9-7-13(24-25(21,22)23)11-12(18)3-4-14-15-5-6-17(20)19(15,2)10-8-16(14)18/h12-16H,3-11H2,1-2H3,(H,21,22,23)/p-1/t12-,13+,14-,15-,16-,18+,19+/m0/s1 || InChI=1S/C19H28O5S/c1-18-9-7-13(24-25(21,22)23)11-12(18)3-4-14-15-5-6-17(20)19(15,2)10-8-16(14)18/h3,13-16H,4-11H2,1-2H3,(H,21,22,23)/t13-,14-,15-,16-,18-,19-/m0/s1</p>**

**</body>**

**</notes>**

**<annotation>**

**<rdf:RDF xmlns:rdf="http://www.w3.org/1999/02/22-rdf-syntax-ns#" xmlns:bqmodel="http://biomodels.net/model-qualifiers/" xmlns:bqbiol="http://biomodels.net/biology-qualifiers/">**

**<rdf:Description rdf:about="c829d49f-38c3-4fe2-838b-837d54f83d6d">**

**<bqbiol:is>**

**<rdf:Bag>**

**<rdf:li rdf:resource="http://identifiers.org/PUBCHEM.COMPOUND/159663"/></rdf:Bag></bqbiol:is><bqbiol:is>**

**<rdf:Bag>**

**<rdf:li rdf:resource="http://identifiers.org/INCHIKEY/ZMITXKRGXGRMKS-HLUDHZFRSA-N"/></rdf:Bag></bqbiol:is><bqbiol:is>**

**<rdf:Bag>**

**<rdf:li rdf:resource="http://identifiers.org/HMDB/HMDB02759"/></rdf:Bag></bqbiol:is><bqbiol:is>**

**<rdf:Bag>**

**<rdf:li rdf:resource="http://identifiers.org/HMDB/HMDB01032"/></rdf:Bag></bqbiol:is><bqbiol:is>**

**<rdf:Bag>**

**<rdf:li rdf:resource="http://identifiers.org/KEGG.COMPOUND/C04555"/></rdf:Bag></bqbiol:is><bqbiol:is>**

**<rdf:Bag>**

**<rdf:li rdf:resource="http://identifiers.org/CHEBI/CHEBI:16814"/></rdf:Bag></bqbiol:is><bqbiol:is>**

**<rdf:Bag>**

**<rdf:li rdf:resource="http://identifiers.org/INCHI/InChI=1S/C19H30O5S/c1-18-9-7-13(24-25(21,22)23)11-12(18)3-4-14-15-5-6-17(20)19(15,2)10-8-16(14)18/h12-16H,3-11H2,1-2H3,(H,21,22,23)/t12-,13+,14-,15-,16-,18-,19-/m0/s1"/></rdf:Bag></bqbiol:is><bqbiol:is>**

**<rdf:Bag>**

**<rdf:li rdf:resource="http://identifiers.org/INCHI/InChI=1/C19H30O5S/c1-18-9-7-13(24-25(21,22)23)11-12(18)3-4-14-15-5-6-17(20)19(15,2)10-8-16(14)18/h12-16H,3-11H2,1-2H3,(H,21,22,23)/p-1/t12-,13+,14-,15-,16-,18+,19+/m0/s1"/></rdf:Bag></bqbiol:is><bqbiol:is>**

**<rdf:Bag>**

**<rdf:li rdf:resource="http://identifiers.org/INCHI/InChI=1S/C19H28O5S/c1-18-9-7-13(24-25(21,22)23)11-12(18)3-4-14-15-5-6-17(20)19(15,2)10-8-16(14)18/h3,13-16H,4-11H2,1-2H3,(H,21,22,23)/t13-,14-,15-,16-,18-,19-/m0/s1"/></rdf:Bag></bqbiol:is></rdf:Description></rdf:RDF>**

**</annotation>**

**</species>**

**<species id="M_mgacpail_hs" constant="false" hasOnlySubstanceUnits="false" name="mannosyl-glucosaminyl-acylphosphatidylinositiol (H2)" metaid="e78d2715-a9ba-426e-a506-5016a2cd314e" boundaryCondition="false" compartment="metaComp">**

**<notes>**

**<body xmlns="http://www.w3.org/1999/xhtml">**

**<p>FORMULA: C37H68NO19PFULLRCO2FULLR2CO2</p>**

**<p>CHARGE: 0</p>**

**</body>**

**</notes>**

**<annotation>**

**<rdf:RDF xmlns:rdf="http://www.w3.org/1999/02/22-rdf-syntax-ns#" xmlns:bqmodel="http://biomodels.net/model-qualifiers/" xmlns:bqbiol="http://biomodels.net/biology-qualifiers/">**

**<rdf:Description rdf:about="e78d2715-a9ba-426e-a506-5016a2cd314e"/></rdf:RDF>**

**</annotation>**

**</species>**

**<species id="M_3mox4hpac" constant="false" hasOnlySubstanceUnits="false" name="(4-hydroxy-3-methoxyphenyl)acetaldehyde" metaid="25f1aeee-b40e-4e99-b4d7-5e57ab31283f" boundaryCondition="false" compartment="metaComp">**

**<notes>**

**<body xmlns="http://www.w3.org/1999/xhtml">**

**<p>FORMULA: C9H10O3</p>**

**<p>CHARGE: 0</p>**

**<p>PUBCHEM.COMPOUND: 151276</p>**

**<p>INCHIKEY: GOQGGGANVKPMNH-UHFFFAOYSA-N</p>**

**<p>KEGG.COMPOUND: C05581</p>**

**<p>HMDB: HMDB05175</p>**

**<p>CHEBI: CHEBI:28111</p>**

**<p>INCHI: InChI=1S/C9H10O3/c1-12-9-6-7(4-5-10)2-3-8(9)11/h2-3,5-6,11H,4H2,1H3</p>**

**</body>**

**</notes>**

**<annotation>**

**<rdf:RDF xmlns:rdf="http://www.w3.org/1999/02/22-rdf-syntax-ns#" xmlns:bqmodel="http://biomodels.net/model-qualifiers/" xmlns:bqbiol="http://biomodels.net/biology-qualifiers/">**

**<rdf:Description rdf:about="_25f1aeee-b40e-4e99-b4d7-5e57ab31283f">**

**<bqbiol:is>**

**<rdf:Bag>**

**<rdf:li rdf:resource="http://identifiers.org/PUBCHEM.COMPOUND/151276"/></rdf:Bag></bqbiol:is><bqbiol:is>**

**<rdf:Bag>**

**<rdf:li rdf:resource="http://identifiers.org/INCHIKEY/GOQGGGANVKPMNH-UHFFFAOYSA-N"/></rdf:Bag></bqbiol:is><bqbiol:is>**

**<rdf:Bag>**

**<rdf:li rdf:resource="http://identifiers.org/KEGG.COMPOUND/C05581"/></rdf:Bag></bqbiol:is><bqbiol:is>**

**<rdf:Bag>**

**<rdf:li rdf:resource="http://identifiers.org/HMDB/HMDB05175"/></rdf:Bag></bqbiol:is><bqbiol:is>**

**<rdf:Bag>**

**<rdf:li rdf:resource="http://identifiers.org/CHEBI/CHEBI:28111"/></rdf:Bag></bqbiol:is><bqbiol:is>**

**<rdf:Bag>**

**<rdf:li rdf:resource="http://identifiers.org/INCHI/InChI=1S/C9H10O3/c1-12-9-6-7(4-5-10)2-3-8(9)11/h2-3,5-6,11H,4H2,1H3"/></rdf:Bag></bqbiol:is></rdf:Description></rdf:RDF>**

**</annotation>**

**</species>**

**<species id="M_phe_L" constant="false" hasOnlySubstanceUnits="false" name="L-phenylalanine" metaid="f3269c6c-9f53-48ca-84d2-7e0b542d0029" boundaryCondition="false" compartment="metaComp">**

**<notes>**

**<body xmlns="http://www.w3.org/1999/xhtml">**

**<p>FORMULA: C9H11NO2</p>**

**<p>CHARGE: 0</p>**

**<p>PUBCHEM.COMPOUND: 6925665 || 71567 || 6919011 || 994 || 6140 || 57397115</p>**

**<p>INCHIKEY: COLNVLDHVKWLRT-QMMMGPOBSA-N</p>**

**<p>KEGG.COMPOUND: C02265 || C02057 || C00079</p>**

**<p>HMDB: HMDB00159</p>**

**<p>CHEBI: CHEBI:16998 || CHEBI:57981 || CHEBI:58095 || CHEBI:17295 || CHEBI:28044</p>**

**<p>INCHI: InChI=1S/C9H11NO2/c10-8(9(11)12)6-7-4-2-1-3-5-7/h1-5,8H,6,10H2,(H,11,12)/t8-/m0/s1</p>**

**</body>**

**</notes>**

**<annotation>**

**<rdf:RDF xmlns:rdf="http://www.w3.org/1999/02/22-rdf-syntax-ns#" xmlns:bqmodel="http://biomodels.net/model-qualifiers/" xmlns:bqbiol="http://biomodels.net/biology-qualifiers/">**

**<rdf:Description rdf:about="f3269c6c-9f53-48ca-84d2-7e0b542d0029">**

**<bqbiol:is>**

**<rdf:Bag>**

**<rdf:li rdf:resource="http://identifiers.org/PUBCHEM.COMPOUND/6925665"/></rdf:Bag></bqbiol:is><bqbiol:is>**

**<rdf:Bag>**

**<rdf:li rdf:resource="http://identifiers.org/PUBCHEM.COMPOUND/71567"/></rdf:Bag></bqbiol:is><bqbiol:is>**

**<rdf:Bag>**

**<rdf:li rdf:resource="http://identifiers.org/PUBCHEM.COMPOUND/6919011"/></rdf:Bag></bqbiol:is><bqbiol:is>**

**<rdf:Bag>**

**<rdf:li rdf:resource="http://identifiers.org/PUBCHEM.COMPOUND/994"/></rdf:Bag></bqbiol:is><bqbiol:is>**

**<rdf:Bag>**

**<rdf:li rdf:resource="http://identifiers.org/PUBCHEM.COMPOUND/6140"/></rdf:Bag></bqbiol:is><bqbiol:is>**

**<rdf:Bag>**

**<rdf:li rdf:resource="http://identifiers.org/PUBCHEM.COMPOUND/57397115"/></rdf:Bag></bqbiol:is><bqbiol:is>**

**<rdf:Bag>**

**<rdf:li rdf:resource="http://identifiers.org/INCHIKEY/COLNVLDHVKWLRT-QMMMGPOBSA-N"/></rdf:Bag></bqbiol:is><bqbiol:is>**

**<rdf:Bag>**

**<rdf:li rdf:resource="http://identifiers.org/KEGG.COMPOUND/C02265"/></rdf:Bag></bqbiol:is><bqbiol:is>**

**<rdf:Bag>**

**<rdf:li rdf:resource="http://identifiers.org/KEGG.COMPOUND/C02057"/></rdf:Bag></bqbiol:is><bqbiol:is>**

**<rdf:Bag>**

**<rdf:li rdf:resource="http://identifiers.org/KEGG.COMPOUND/C00079"/></rdf:Bag></bqbiol:is><bqbiol:is>**

**<rdf:Bag>**

**<rdf:li rdf:resource="http://identifiers.org/HMDB/HMDB00159"/></rdf:Bag></bqbiol:is><bqbiol:is>**

**<rdf:Bag>**

**<rdf:li rdf:resource="http://identifiers.org/CHEBI/CHEBI:16998"/></rdf:Bag></bqbiol:is><bqbiol:is>**

**<rdf:Bag>**

**<rdf:li rdf:resource="http://identifiers.org/CHEBI/CHEBI:57981"/></rdf:Bag></bqbiol:is><bqbiol:is>**

**<rdf:Bag>**

**<rdf:li rdf:resource="http://identifiers.org/CHEBI/CHEBI:58095"/></rdf:Bag></bqbiol:is><bqbiol:is>**

**<rdf:Bag>**

**<rdf:li rdf:resource="http://identifiers.org/CHEBI/CHEBI:17295"/></rdf:Bag></bqbiol:is><bqbiol:is>**

**<rdf:Bag>**

**<rdf:li rdf:resource="http://identifiers.org/CHEBI/CHEBI:28044"/></rdf:Bag></bqbiol:is><bqbiol:is>**

**<rdf:Bag>**

**<rdf:li rdf:resource="http://identifiers.org/INCHI/InChI=1S/C9H11NO2/c10-8(9(11)12)6-7-4-2-1-3-5-7/h1-5,8H,6,10H2,(H,11,12)/t8-/m0/s1"/></rdf:Bag></bqbiol:is></rdf:Description></rdf:RDF>**

**</annotation>**

**</species>**

**<species id="M_CE4633" constant="false" hasOnlySubstanceUnits="false" name="hypochlorous acid" metaid="35e239fe-5aff-40ab-8a36-0125349b3458" boundaryCondition="false" compartment="metaComp">**

**<notes>**

**<body xmlns="http://www.w3.org/1999/xhtml">**

**<p>FORMULA: CLHO</p>**

**<p>CHARGE: 0</p>**

**<p>PUBCHEM.COMPOUND: 24341</p>**

**<p>INCHIKEY: QWPPOHNGKGFGJK-UHFFFAOYSA-N</p>**

**<p>KEGG.COMPOUND: C19697</p>**

**<p>CHEBI: CHEBI:24757</p>**

**<p>INCHI: InChI=1S/ClHO/c1-2/h2H</p>**

**</body>**

**</notes>**

**<annotation>**

**<rdf:RDF xmlns:rdf="http://www.w3.org/1999/02/22-rdf-syntax-ns#" xmlns:bqmodel="http://biomodels.net/model-qualifiers/" xmlns:bqbiol="http://biomodels.net/biology-qualifiers/">**

**<rdf:Description rdf:about="_35e239fe-5aff-40ab-8a36-0125349b3458">**

**<bqbiol:is>**

**<rdf:Bag>**

**<rdf:li rdf:resource="http://identifiers.org/PUBCHEM.COMPOUND/24341"/></rdf:Bag></bqbiol:is><bqbiol:is>**

**<rdf:Bag>**

**<rdf:li rdf:resource="http://identifiers.org/INCHIKEY/QWPPOHNGKGFGJK-UHFFFAOYSA-N"/></rdf:Bag></bqbiol:is><bqbiol:is>**

**<rdf:Bag>**

**<rdf:li rdf:resource="http://identifiers.org/KEGG.COMPOUND/C19697"/></rdf:Bag></bqbiol:is><bqbiol:is>**

**<rdf:Bag>**

**<rdf:li rdf:resource="http://identifiers.org/CHEBI/CHEBI:24757"/></rdf:Bag></bqbiol:is><bqbiol:is>**

**<rdf:Bag>**

**<rdf:li rdf:resource="http://identifiers.org/INCHI/InChI=1S/ClHO/c1-2/h2H"/></rdf:Bag></bqbiol:is></rdf:Description></rdf:RDF>**

**</annotation>**

**</species>**

**<species id="M_13_cis_retnglc" constant="false" hasOnlySubstanceUnits="false" name="13-cis-retinoyl glucuronide" metaid="53ad5e09-9a14-42e8-b78e-547ca4892816" boundaryCondition="false" compartment="metaComp">**

**<notes>**

**<body xmlns="http://www.w3.org/1999/xhtml">**

**<p>FORMULA: C26H35O8</p>**

**<p>CHARGE: 0</p>**

**<p>PUBCHEM.COMPOUND: 5281877</p>**

**<p>KEGG.COMPOUND: C11061</p>**

**<p>HMDB: HMDB03141</p>**

**<p>CHEBI: CHEBI:28870</p>**

**<p>INCHI: InChI=1S/C26H36O8/c1-15(11-12-18-17(3)10-7-13-26(18,4)5)8-6-9-16(2)14-19(27)33-25-22(30)20(28)21(29)23(34-25)24(31)32/h6,8-9,11-12,14,20-23,25,28-30H,7,10,13H2,1-5H3,(H,31,32)/b9-6+,12-11+,15-8+,16-14+/t20-,21-,22+,23-,25+/m0/s1</p>**

**</body>**

**</notes>**

**<annotation>**

**<rdf:RDF xmlns:rdf="http://www.w3.org/1999/02/22-rdf-syntax-ns#" xmlns:bqmodel="http://biomodels.net/model-qualifiers/" xmlns:bqbiol="http://biomodels.net/biology-qualifiers/">**

**<rdf:Description rdf:about="_53ad5e09-9a14-42e8-b78e-547ca4892816">**

**<bqbiol:is>**

**<rdf:Bag>**

**<rdf:li rdf:resource="http://identifiers.org/PUBCHEM.COMPOUND/5281877"/></rdf:Bag></bqbiol:is><bqbiol:is>**

**<rdf:Bag>**

**<rdf:li rdf:resource="http://identifiers.org/KEGG.COMPOUND/C11061"/></rdf:Bag></bqbiol:is><bqbiol:is>**

**<rdf:Bag>**

**<rdf:li rdf:resource="http://identifiers.org/HMDB/HMDB03141"/></rdf:Bag></bqbiol:is><bqbiol:is>**

**<rdf:Bag>**

**<rdf:li rdf:resource="http://identifiers.org/CHEBI/CHEBI:28870"/></rdf:Bag></bqbiol:is><bqbiol:is>**

**<rdf:Bag>**

**<rdf:li rdf:resource="http://identifiers.org/INCHI/InChI=1S/C26H36O8/c1-15(11-12-18-17(3)10-7-13-26(18,4)5)8-6-9-16(2)14-19(27)33-25-22(30)20(28)21(29)23(34-25)24(31)32/h6,8-9,11-12,14,20-23,25,28-30H,7,10,13H2,1-5H3,(H,31,32)/b9-6+,12-11+,15-8+,16-14+/t20-,21-,22+,23-,25+/m0/s1"/></rdf:Bag></bqbiol:is></rdf:Description></rdf:RDF>**

**</annotation>**

**</species>**

**<species id="M_C02147" constant="false" hasOnlySubstanceUnits="false" name="dihydrolipoate" metaid="f347198b-3e7d-40ec-9fd3-d6a558159783" boundaryCondition="false" compartment="metaComp">**

**<notes>**

**<body xmlns="http://www.w3.org/1999/xhtml">**

**<p>FORMULA: C8H15O2S2</p>**

**<p>CHARGE: 0</p>**

**<p>PUBCHEM.COMPOUND: 421</p>**

**<p>INCHIKEY: IZFHEQBZOYJLPK-UHFFFAOYSA-N</p>**

**<p>KEGG.COMPOUND: C02147</p>**

**<p>HMDB: HMDB12210</p>**

**<p>CHEBI: CHEBI:30316 || CHEBI:18047</p>**

**<p>INCHI: InChI=1S/C8H16O2S2/c9-8(10)4-2-1-3-7(12)5-6-11/h7,11-12H,1-6H2,(H,9,10) || InChI=1S/C8H16O2S2/c9-8(10)4-2-1-3-7(12)5-6-11/h7,11-12H,1-6H2,(H,9,10)/p-1</p>**

**</body>**

**</notes>**

**<annotation>**

**<rdf:RDF xmlns:rdf="http://www.w3.org/1999/02/22-rdf-syntax-ns#" xmlns:bqmodel="http://biomodels.net/model-qualifiers/" xmlns:bqbiol="http://biomodels.net/biology-qualifiers/">**

**<rdf:Description rdf:about="f347198b-3e7d-40ec-9fd3-d6a558159783">**

**<bqbiol:is>**

**<rdf:Bag>**

**<rdf:li rdf:resource="http://identifiers.org/PUBCHEM.COMPOUND/421"/></rdf:Bag></bqbiol:is><bqbiol:is>**

**<rdf:Bag>**

**<rdf:li rdf:resource="http://identifiers.org/INCHIKEY/IZFHEQBZOYJLPK-UHFFFAOYSA-N"/></rdf:Bag></bqbiol:is><bqbiol:is>**

**<rdf:Bag>**

**<rdf:li rdf:resource="http://identifiers.org/KEGG.COMPOUND/C02147"/></rdf:Bag></bqbiol:is><bqbiol:is>**

**<rdf:Bag>**

**<rdf:li rdf:resource="http://identifiers.org/HMDB/HMDB12210"/></rdf:Bag></bqbiol:is><bqbiol:is>**

**<rdf:Bag>**

**<rdf:li rdf:resource="http://identifiers.org/CHEBI/CHEBI:30316"/></rdf:Bag></bqbiol:is><bqbiol:is>**

**<rdf:Bag>**

**<rdf:li rdf:resource="http://identifiers.org/CHEBI/CHEBI:18047"/></rdf:Bag></bqbiol:is><bqbiol:is>**

**<rdf:Bag>**

**<rdf:li rdf:resource="http://identifiers.org/INCHI/InChI=1S/C8H16O2S2/c9-8(10)4-2-1-3-7(12)5-6-11/h7,11-12H,1-6H2,(H,9,10)"/></rdf:Bag></bqbiol:is><bqbiol:is>**

**<rdf:Bag>**

**<rdf:li rdf:resource="http://identifiers.org/INCHI/InChI=1S/C8H16O2S2/c9-8(10)4-2-1-3-7(12)5-6-11/h7,11-12H,1-6H2,(H,9,10)/p-1"/></rdf:Bag></bqbiol:is></rdf:Description></rdf:RDF>**

**</annotation>**

**</species>**

**<species id="M_CE1447" constant="false" hasOnlySubstanceUnits="false" name="11-dehydrothromboxane B2" metaid="832020b4-50e9-468d-a432-529c451bfdc0" boundaryCondition="false" compartment="metaComp">**

**<notes>**

**<body xmlns="http://www.w3.org/1999/xhtml">**

**<p>FORMULA: C20H31O6</p>**

**<p>CHARGE: 0</p>**

**<p>PUBCHEM.COMPOUND: 440862 || 53477781</p>**

**<p>KEGG.COMPOUND: C05964</p>**

**<p>HMDB: HMDB04242</p>**

**<p>CHEBI: CHEBI:28667</p>**

**<p>INCHI: InChI=1S/C20H32O6/c1-2-3-6-9-15(21)12-13-18-16(17(22)14-20(25)26-18)10-7-4-5-8-11-19(23)24/h4,7,12-13,15-18,21-22H,2-3,5-6,8-11,14H2,1H3,(H,23,24)/b7-4-,13-12+/t15-,16-,17-,18+/m0/s1 || InChI=1S/C20H32O6/c1-2-3-6-9-15(21)12-13-18-16(17(22)14-20(25)26-18)10-7-4-5-8-11-19(23)24/h4,7,12-13,15-18,21-22H,2-3,5-6,8-11,14H2,1H3,(H,23,24)/b7-4+,13-12+/t15-,16-,17-,18+/m0/s1</p>**

**</body>**

**</notes>**

**<annotation>**

**<rdf:RDF xmlns:rdf="http://www.w3.org/1999/02/22-rdf-syntax-ns#" xmlns:bqmodel="http://biomodels.net/model-qualifiers/" xmlns:bqbiol="http://biomodels.net/biology-qualifiers/">**

**<rdf:Description rdf:about="_832020b4-50e9-468d-a432-529c451bfdc0">**

**<bqbiol:is>**

**<rdf:Bag>**

**<rdf:li rdf:resource="http://identifiers.org/PUBCHEM.COMPOUND/440862"/></rdf:Bag></bqbiol:is><bqbiol:is>**

**<rdf:Bag>**

**<rdf:li rdf:resource="http://identifiers.org/PUBCHEM.COMPOUND/53477781"/></rdf:Bag></bqbiol:is><bqbiol:is>**

**<rdf:Bag>**

**<rdf:li rdf:resource="http://identifiers.org/KEGG.COMPOUND/C05964"/></rdf:Bag></bqbiol:is><bqbiol:is>**

**<rdf:Bag>**

**<rdf:li rdf:resource="http://identifiers.org/HMDB/HMDB04242"/></rdf:Bag></bqbiol:is><bqbiol:is>**

**<rdf:Bag>**

**<rdf:li rdf:resource="http://identifiers.org/CHEBI/CHEBI:28667"/></rdf:Bag></bqbiol:is><bqbiol:is>**

**<rdf:Bag>**

**<rdf:li rdf:resource="http://identifiers.org/INCHI/InChI=1S/C20H32O6/c1-2-3-6-9-15(21)12-13-18-16(17(22)14-20(25)26-18)10-7-4-5-8-11-19(23)24/h4,7,12-13,15-18,21-22H,2-3,5-6,8-11,14H2,1H3,(H,23,24)/b7-4-,13-12+/t15-,16-,17-,18+/m0/s1"/></rdf:Bag></bqbiol:is><bqbiol:is>**

**<rdf:Bag>**

**<rdf:li rdf:resource="http://identifiers.org/INCHI/InChI=1S/C20H32O6/c1-2-3-6-9-15(21)12-13-18-16(17(22)14-20(25)26-18)10-7-4-5-8-11-19(23)24/h4,7,12-13,15-18,21-22H,2-3,5-6,8-11,14H2,1H3,(H,23,24)/b7-4+,13-12+/t15-,16-,17-,18+/m0/s1"/></rdf:Bag></bqbiol:is></rdf:Description></rdf:RDF>**

**</annotation>**

**</species>**

**<species id="M_utp" constant="false" hasOnlySubstanceUnits="false" name="UTP(4-)" metaid="63f40078-62ff-4413-b6a6-346eed1e8cf8" boundaryCondition="false" compartment="metaComp">**

**<notes>**

**<body xmlns="http://www.w3.org/1999/xhtml">**

**<p>FORMULA: C9H11N2O15P3</p>**

**<p>CHARGE: 0</p>**

**<p>PUBCHEM.COMPOUND: 6133</p>**

**<p>KEGG.COMPOUND: C00075</p>**

**<p>HMDB: HMDB00285</p>**

**<p>CHEBI: CHEBI:15713 || CHEBI:46398</p>**

**<p>INCHI: InChI=1S/C9H15N2O15P3/c12-5-1-2-11(9(15)10-5)8-7(14)6(13)4(24-8)3-23-28(19,20)26-29(21,22)25-27(16,17)18/h1-2,4,6-8,13-14H,3H2,(H,19,20)(H,21,22)(H,10,12,15)(H2,16,17,18)/p-4/t4-,6-,7-,8-/m1/s1 || InChI=1S/C9H15N2O15P3/c12-5-1-2-11(9(15)10-5)8-7(14)6(13)4(24-8)3-23-28(19,20)26-29(21,22)25-27(16,17)18/h1-2,4,6-8,13-14H,3H2,(H,19,20)(H,21,22)(H,10,12,15)(H2,16,17,18)/t4-,6-,7-,8-/m1/s1</p>**

**</body>**

**</notes>**

**<annotation>**

**<rdf:RDF xmlns:rdf="http://www.w3.org/1999/02/22-rdf-syntax-ns#" xmlns:bqmodel="http://biomodels.net/model-qualifiers/" xmlns:bqbiol="http://biomodels.net/biology-qualifiers/">**

**<rdf:Description rdf:about="_63f40078-62ff-4413-b6a6-346eed1e8cf8">**

**<bqbiol:is>**

**<rdf:Bag>**

**<rdf:li rdf:resource="http://identifiers.org/PUBCHEM.COMPOUND/6133"/></rdf:Bag></bqbiol:is><bqbiol:is>**

**<rdf:Bag>**

**<rdf:li rdf:resource="http://identifiers.org/KEGG.COMPOUND/C00075"/></rdf:Bag></bqbiol:is><bqbiol:is>**

**<rdf:Bag>**

**<rdf:li rdf:resource="http://identifiers.org/HMDB/HMDB00285"/></rdf:Bag></bqbiol:is><bqbiol:is>**

**<rdf:Bag>**

**<rdf:li rdf:resource="http://identifiers.org/CHEBI/CHEBI:15713"/></rdf:Bag></bqbiol:is><bqbiol:is>**

**<rdf:Bag>**

**<rdf:li rdf:resource="http://identifiers.org/CHEBI/CHEBI:46398"/></rdf:Bag></bqbiol:is><bqbiol:is>**

**<rdf:Bag>**

**<rdf:li rdf:resource="http://identifiers.org/INCHI/InChI=1S/C9H15N2O15P3/c12-5-1-2-11(9(15)10-5)8-7(14)6(13)4(24-8)3-23-28(19,20)26-29(21,22)25-27(16,17)18/h1-2,4,6-8,13-14H,3H2,(H,19,20)(H,21,22)(H,10,12,15)(H2,16,17,18)/p-4/t4-,6-,7-,8-/m1/s1"/></rdf:Bag></bqbiol:is><bqbiol:is>**

**<rdf:Bag>**

**<rdf:li rdf:resource="http://identifiers.org/INCHI/InChI=1S/C9H15N2O15P3/c12-5-1-2-11(9(15)10-5)8-7(14)6(13)4(24-8)3-23-28(19,20)26-29(21,22)25-27(16,17)18/h1-2,4,6-8,13-14H,3H2,(H,19,20)(H,21,22)(H,10,12,15)(H2,16,17,18)/t4-,6-,7-,8-/m1/s1"/></rdf:Bag></bqbiol:is></rdf:Description></rdf:RDF>**

**</annotation>**

**</species>**

**<species id="M_selnp" constant="false" hasOnlySubstanceUnits="false" name="Selenophosphate" metaid="79d50f9e-992f-4e81-a555-ea7ac3e0f0d0" boundaryCondition="false" compartment="metaComp">**

**<notes>**

**<body xmlns="http://www.w3.org/1999/xhtml">**

**<p>FORMULA: H2O3PSE</p>**

**<p>CHARGE: 0</p>**

**<p>PUBCHEM.COMPOUND: 1092</p>**

**<p>INCHIKEY: JRPHGDYSKGJTKZ-UHFFFAOYSA-M</p>**

**<p>HMDB: HMDB06407 || HMDB03840</p>**

**<p>KEGG.COMPOUND: C05172</p>**

**<p>CHEBI: CHEBI:29269 || CHEBI:64331 || CHEBI:16144</p>**

**<p>INCHI: InChI=1S/H3O3PSe/c1-4(2,3)5/h(H3,1,2,3,5)/p-1 || InChI=1S/H3O3PSe/c1-4(2,3)5/h(H3,1,2,3,5)/p-3 || InChI=1S/H3O3PSe/c1-4(2,3)5/h(H3,1,2,3,5)</p>**

**</body>**

**</notes>**

**<annotation>**

**<rdf:RDF xmlns:rdf="http://www.w3.org/1999/02/22-rdf-syntax-ns#" xmlns:bqmodel="http://biomodels.net/model-qualifiers/" xmlns:bqbiol="http://biomodels.net/biology-qualifiers/">**

**<rdf:Description rdf:about="_79d50f9e-992f-4e81-a555-ea7ac3e0f0d0">**

**<bqbiol:is>**

**<rdf:Bag>**

**<rdf:li rdf:resource="http://identifiers.org/PUBCHEM.COMPOUND/1092"/></rdf:Bag></bqbiol:is><bqbiol:is>**

**<rdf:Bag>**

**<rdf:li rdf:resource="http://identifiers.org/INCHIKEY/JRPHGDYSKGJTKZ-UHFFFAOYSA-M"/></rdf:Bag></bqbiol:is><bqbiol:is>**

**<rdf:Bag>**

**<rdf:li rdf:resource="http://identifiers.org/HMDB/HMDB06407"/></rdf:Bag></bqbiol:is><bqbiol:is>**

**<rdf:Bag>**

**<rdf:li rdf:resource="http://identifiers.org/HMDB/HMDB03840"/></rdf:Bag></bqbiol:is><bqbiol:is>**

**<rdf:Bag>**

**<rdf:li rdf:resource="http://identifiers.org/KEGG.COMPOUND/C05172"/></rdf:Bag></bqbiol:is><bqbiol:is>**

**<rdf:Bag>**

**<rdf:li rdf:resource="http://identifiers.org/CHEBI/CHEBI:29269"/></rdf:Bag></bqbiol:is><bqbiol:is>**

**<rdf:Bag>**

**<rdf:li rdf:resource="http://identifiers.org/CHEBI/CHEBI:64331"/></rdf:Bag></bqbiol:is><bqbiol:is>**

**<rdf:Bag>**

**<rdf:li rdf:resource="http://identifiers.org/CHEBI/CHEBI:16144"/></rdf:Bag></bqbiol:is><bqbiol:is>**

**<rdf:Bag>**

**<rdf:li rdf:resource="http://identifiers.org/INCHI/InChI=1S/H3O3PSe/c1-4(2,3)5/h(H3,1,2,3,5)/p-1"/></rdf:Bag></bqbiol:is><bqbiol:is>**

**<rdf:Bag>**

**<rdf:li rdf:resource="http://identifiers.org/INCHI/InChI=1S/H3O3PSe/c1-4(2,3)5/h(H3,1,2,3,5)/p-3"/></rdf:Bag></bqbiol:is><bqbiol:is>**

**<rdf:Bag>**

**<rdf:li rdf:resource="http://identifiers.org/INCHI/InChI=1S/H3O3PSe/c1-4(2,3)5/h(H3,1,2,3,5)"/></rdf:Bag></bqbiol:is></rdf:Description></rdf:RDF>**

**</annotation>**

**</species>**

**<species id="M_cmpacna" constant="false" hasOnlySubstanceUnits="false" name="CMP-N-acetyl-beta-neuraminate(2-)" metaid="71831308-4cb6-4dca-8553-b79394d8c542" boundaryCondition="false" compartment="metaComp">**

**<notes>**

**<body xmlns="http://www.w3.org/1999/xhtml">**

**<p>FORMULA: C20H29N4O16P</p>**

**<p>CHARGE: 0</p>**

**<p>PUBCHEM.COMPOUND: 448209</p>**

**<p>KEGG.COMPOUND: C00128</p>**

**<p>HMDB: HMDB01176</p>**

**<p>CHEBI: CHEBI:16556 || CHEBI:57812</p>**

**<p>INCHI: InChI=1S/C20H31N4O16P/c1-7(26)22-12-8(27)4-20(18(32)33,39-16(12)13(29)9(28)5-25)40-41(35,36)37-6-10-14(30)15(31)17(38-10)24-3-2-11(21)23-19(24)34/h2-3,8-10,12-17,25,27-31H,4-6H2,1H3,(H,22,26)(H,32,33)(H,35,36)(H2,21,23,34)/t8-,9+,10+,12+,13+,14+,15+,16+,17+,20+/m0/s1 || InChI=1S/C20H31N4O16P/c1-7(26)22-12-8(27)4-20(18(32)33,39-16(12)13(29)9(28)5-25)40-41(35,36)37-6-10-14(30)15(31)17(38-10)24-3-2-11(21)23-19(24)34/h2-3,8-10,12-17,25,27-31H,4-6H2,1H3,(H,22,26)(H,32,33)(H,35,36)(H2,21,23,34)/p-2/t8-,9+,10+,12+,13+,14+,15+,16+,17+,20+/m0/s1</p>**

**</body>**

**</notes>**

**<annotation>**

**<rdf:RDF xmlns:rdf="http://www.w3.org/1999/02/22-rdf-syntax-ns#" xmlns:bqmodel="http://biomodels.net/model-qualifiers/" xmlns:bqbiol="http://biomodels.net/biology-qualifiers/">**

**<rdf:Description rdf:about="_71831308-4cb6-4dca-8553-b79394d8c542">**

**<bqbiol:is>**

**<rdf:Bag>**

**<rdf:li rdf:resource="http://identifiers.org/PUBCHEM.COMPOUND/448209"/></rdf:Bag></bqbiol:is><bqbiol:is>**

**<rdf:Bag>**

**<rdf:li rdf:resource="http://identifiers.org/KEGG.COMPOUND/C00128"/></rdf:Bag></bqbiol:is><bqbiol:is>**

**<rdf:Bag>**

**<rdf:li rdf:resource="http://identifiers.org/HMDB/HMDB01176"/></rdf:Bag></bqbiol:is><bqbiol:is>**

**<rdf:Bag>**

**<rdf:li rdf:resource="http://identifiers.org/CHEBI/CHEBI:16556"/></rdf:Bag></bqbiol:is><bqbiol:is>**

**<rdf:Bag>**

**<rdf:li rdf:resource="http://identifiers.org/CHEBI/CHEBI:57812"/></rdf:Bag></bqbiol:is><bqbiol:is>**

**<rdf:Bag>**

**<rdf:li rdf:resource="http://identifiers.org/INCHI/InChI=1S/C20H31N4O16P/c1-7(26)22-12-8(27)4-20(18(32)33,39-16(12)13(29)9(28)5-25)40-41(35,36)37-6-10-14(30)15(31)17(38-10)24-3-2-11(21)23-19(24)34/h2-3,8-10,12-17,25,27-31H,4-6H2,1H3,(H,22,26)(H,32,33)(H,35,36)(H2,21,23,34)/t8-,9+,10+,12+,13+,14+,15+,16+,17+,20+/m0/s1"/></rdf:Bag></bqbiol:is><bqbiol:is>**

**<rdf:Bag>**

**<rdf:li rdf:resource="http://identifiers.org/INCHI/InChI=1S/C20H31N4O16P/c1-7(26)22-12-8(27)4-20(18(32)33,39-16(12)13(29)9(28)5-25)40-41(35,36)37-6-10-14(30)15(31)17(38-10)24-3-2-11(21)23-19(24)34/h2-3,8-10,12-17,25,27-31H,4-6H2,1H3,(H,22,26)(H,32,33)(H,35,36)(H2,21,23,34)/p-2/t8-,9+,10+,12+,13+,14+,15+,16+,17+,20+/m0/s1"/></rdf:Bag></bqbiol:is></rdf:Description></rdf:RDF>**

**</annotation>**

**</species>**

**<species id="M_orn" constant="false" hasOnlySubstanceUnits="false" name="Ornithine" metaid="5d5b304f-60de-4c29-a597-1d6ee7f9ff11" boundaryCondition="false" compartment="metaComp">**

**<notes>**

**<body xmlns="http://www.w3.org/1999/xhtml">**

**<p>FORMULA: C5H13N2O2</p>**

**<p>CHARGE: 0</p>**

**<p>PUBCHEM.COMPOUND: 6262</p>**

**<p>INCHIKEY: AHLPHDHHMVZTML-UHFFFAOYSA-N</p>**

**<p>KEGG.COMPOUND: C00077 || C01602</p>**

**<p>HMDB: HMDB00214</p>**

**<p>CHEBI: CHEBI:46912 || CHEBI:46911 || CHEBI:18257 || CHEBI:15729</p>**

**<p>INCHI: InChI=1S/C5H12N2O2/c6-3-1-2-4(7)5(8)9/h4H,1-3,6-7H2,(H,8,9)/t4-/m0/s1 || InChI=1S/C5H12N2O2/c6-3-1-2-4(7)5(8)9/h4H,1-3,6-7H2,(H,8,9) || InChI=1S/C5H12N2O2/c6-3-1-2-4(7)5(8)9/h4H,1-3,6-7H2,(H,8,9)/p+1</p>**

**</body>**

**</notes>**

**<annotation>**

**<rdf:RDF xmlns:rdf="http://www.w3.org/1999/02/22-rdf-syntax-ns#" xmlns:bqmodel="http://biomodels.net/model-qualifiers/" xmlns:bqbiol="http://biomodels.net/biology-qualifiers/">**

**<rdf:Description rdf:about="_5d5b304f-60de-4c29-a597-1d6ee7f9ff11">**

**<bqbiol:is>**

**<rdf:Bag>**

**<rdf:li rdf:resource="http://identifiers.org/PUBCHEM.COMPOUND/6262"/></rdf:Bag></bqbiol:is><bqbiol:is>**

**<rdf:Bag>**

**<rdf:li rdf:resource="http://identifiers.org/INCHIKEY/AHLPHDHHMVZTML-UHFFFAOYSA-N"/></rdf:Bag></bqbiol:is><bqbiol:is>**

**<rdf:Bag>**

**<rdf:li rdf:resource="http://identifiers.org/KEGG.COMPOUND/C00077"/></rdf:Bag></bqbiol:is><bqbiol:is>**

**<rdf:Bag>**

**<rdf:li rdf:resource="http://identifiers.org/KEGG.COMPOUND/C01602"/></rdf:Bag></bqbiol:is><bqbiol:is>**

**<rdf:Bag>**

**<rdf:li rdf:resource="http://identifiers.org/HMDB/HMDB00214"/></rdf:Bag></bqbiol:is><bqbiol:is>**

**<rdf:Bag>**

**<rdf:li rdf:resource="http://identifiers.org/CHEBI/CHEBI:46912"/></rdf:Bag></bqbiol:is><bqbiol:is>**

**<rdf:Bag>**

**<rdf:li rdf:resource="http://identifiers.org/CHEBI/CHEBI:46911"/></rdf:Bag></bqbiol:is><bqbiol:is>**

**<rdf:Bag>**

**<rdf:li rdf:resource="http://identifiers.org/CHEBI/CHEBI:18257"/></rdf:Bag></bqbiol:is><bqbiol:is>**

**<rdf:Bag>**

**<rdf:li rdf:resource="http://identifiers.org/CHEBI/CHEBI:15729"/></rdf:Bag></bqbiol:is><bqbiol:is>**

**<rdf:Bag>**

**<rdf:li rdf:resource="http://identifiers.org/INCHI/InChI=1S/C5H12N2O2/c6-3-1-2-4(7)5(8)9/h4H,1-3,6-7H2,(H,8,9)/t4-/m0/s1"/></rdf:Bag></bqbiol:is><bqbiol:is>**

**<rdf:Bag>**

**<rdf:li rdf:resource="http://identifiers.org/INCHI/InChI=1S/C5H12N2O2/c6-3-1-2-4(7)5(8)9/h4H,1-3,6-7H2,(H,8,9)"/></rdf:Bag></bqbiol:is><bqbiol:is>**

**<rdf:Bag>**

**<rdf:li rdf:resource="http://identifiers.org/INCHI/InChI=1S/C5H12N2O2/c6-3-1-2-4(7)5(8)9/h4H,1-3,6-7H2,(H,8,9)/p+1"/></rdf:Bag></bqbiol:is></rdf:Description></rdf:RDF>**

**</annotation>**

**</species>**

**<species id="M_chlstol" constant="false" hasOnlySubstanceUnits="false" name="5alpha-cholesta-7,24-dien-3beta-ol" metaid="dde85456-c2c0-4a36-8205-278067ab5de6" boundaryCondition="false" compartment="metaComp">**

**<notes>**

**<body xmlns="http://www.w3.org/1999/xhtml">**

**<p>FORMULA: C27H44O</p>**

**<p>CHARGE: 0</p>**

**<p>PUBCHEM.COMPOUND: 50986070 || 440670 || 5459827</p>**

**<p>INCHIKEY: PKEPPDGGTSZLBL-FZAJRYLSSA-N</p>**

**<p>KEGG.COMPOUND: C05439</p>**

**<p>HMDB: HMDB06842</p>**

**<p>CHEBI: CHEBI:16290</p>**

**<p>INCHI: InChI=1S/C27H44O/c1-18(2)7-6-8-19(3)23-11-12-24-22-10-9-20-17-21(28)13-15-26(20,4)25(22)14-16-27(23,24)5/h7,10,19-21,23-25,28H,6,8-9,11-17H2,1-5H3/t19-,20+,21+,23-,24+,25+,26+,27-/m1/s1 || InChI=1S/C27H44O/c1-18(2)7-6-8-19(3)23-11-12-24-22-10-9-20-17-21(28)13-15-26(20,4)25(22)14-16-27(23,24)5/h7,10,19-21,23-25,28H,6,8-9,11-17H2,1-5H3/t19-,20+,21+,23-,24+,25?,26+,27-/m1/s1</p>**

**</body>**

**</notes>**

**<annotation>**

**<rdf:RDF xmlns:rdf="http://www.w3.org/1999/02/22-rdf-syntax-ns#" xmlns:bqmodel="http://biomodels.net/model-qualifiers/" xmlns:bqbiol="http://biomodels.net/biology-qualifiers/">**

**<rdf:Description rdf:about="dde85456-c2c0-4a36-8205-278067ab5de6">**

**<bqbiol:is>**

**<rdf:Bag>**

**<rdf:li rdf:resource="http://identifiers.org/PUBCHEM.COMPOUND/50986070"/></rdf:Bag></bqbiol:is><bqbiol:is>**

**<rdf:Bag>**

**<rdf:li rdf:resource="http://identifiers.org/PUBCHEM.COMPOUND/440670"/></rdf:Bag></bqbiol:is><bqbiol:is>**

**<rdf:Bag>**

**<rdf:li rdf:resource="http://identifiers.org/PUBCHEM.COMPOUND/5459827"/></rdf:Bag></bqbiol:is><bqbiol:is>**

**<rdf:Bag>**

**<rdf:li rdf:resource="http://identifiers.org/INCHIKEY/PKEPPDGGTSZLBL-FZAJRYLSSA-N"/></rdf:Bag></bqbiol:is><bqbiol:is>**

**<rdf:Bag>**

**<rdf:li rdf:resource="http://identifiers.org/KEGG.COMPOUND/C05439"/></rdf:Bag></bqbiol:is><bqbiol:is>**

**<rdf:Bag>**

**<rdf:li rdf:resource="http://identifiers.org/HMDB/HMDB06842"/></rdf:Bag></bqbiol:is><bqbiol:is>**

**<rdf:Bag>**

**<rdf:li rdf:resource="http://identifiers.org/CHEBI/CHEBI:16290"/></rdf:Bag></bqbiol:is><bqbiol:is>**

**<rdf:Bag>**

**<rdf:li rdf:resource="http://identifiers.org/INCHI/InChI=1S/C27H44O/c1-18(2)7-6-8-19(3)23-11-12-24-22-10-9-20-17-21(28)13-15-26(20,4)25(22)14-16-27(23,24)5/h7,10,19-21,23-25,28H,6,8-9,11-17H2,1-5H3/t19-,20+,21+,23-,24+,25+,26+,27-/m1/s1"/></rdf:Bag></bqbiol:is><bqbiol:is>**

**<rdf:Bag>**

**<rdf:li rdf:resource="http://identifiers.org/INCHI/InChI=1S/C27H44O/c1-18(2)7-6-8-19(3)23-11-12-24-22-10-9-20-17-21(28)13-15-26(20,4)25(22)14-16-27(23,24)5/h7,10,19-21,23-25,28H,6,8-9,11-17H2,1-5H3/t19-,20+,21+,23-,24+,25?,26+,27-/m1/s1"/></rdf:Bag></bqbiol:is></rdf:Description></rdf:RDF>**

**</annotation>**

**</species>**

**<species id="M_tdcrn" constant="false" hasOnlySubstanceUnits="false" name="myristoyl carnitine" metaid="a347eeea-f288-4de4-bee1-cce506d2acad" boundaryCondition="false" compartment="metaComp">**

**<notes>**

**<body xmlns="http://www.w3.org/1999/xhtml">**

**<p>FORMULA: C21H41NO4</p>**

**<p>CHARGE: 0</p>**

**<p>PUBCHEM.COMPOUND: 53477791</p>**

**<p>HMDB: HMDB05066</p>**

**<p>INCHI: InChI=1S/C21H41NO4/c1-5-6-7-8-9-10-11-12-13-14-15-16-21(25)26-19(17-20(23)24)18-22(2,3)4/h19H,5-18H2,1-4H3/t19-/m1/s1</p>**

**</body>**

**</notes>**

**<annotation>**

**<rdf:RDF xmlns:rdf="http://www.w3.org/1999/02/22-rdf-syntax-ns#" xmlns:bqmodel="http://biomodels.net/model-qualifiers/" xmlns:bqbiol="http://biomodels.net/biology-qualifiers/">**

**<rdf:Description rdf:about="a347eeea-f288-4de4-bee1-cce506d2acad">**

**<bqbiol:is>**

**<rdf:Bag>**

**<rdf:li rdf:resource="http://identifiers.org/PUBCHEM.COMPOUND/53477791"/></rdf:Bag></bqbiol:is><bqbiol:is>**

**<rdf:Bag>**

**<rdf:li rdf:resource="http://identifiers.org/HMDB/HMDB05066"/></rdf:Bag></bqbiol:is><bqbiol:is>**

**<rdf:Bag>**

**<rdf:li rdf:resource="http://identifiers.org/INCHI/InChI=1S/C21H41NO4/c1-5-6-7-8-9-10-11-12-13-14-15-16-21(25)26-19(17-20(23)24)18-22(2,3)4/h19H,5-18H2,1-4H3/t19-/m1/s1"/></rdf:Bag></bqbiol:is></rdf:Description></rdf:RDF>**

**</annotation>**

**</species>**

**<species id="M_1ddecg3p" constant="false" hasOnlySubstanceUnits="false" name="1-acyl-sn-glycerol 3-phosphate(2-)" metaid="68a2417c-ee5a-44e5-b650-1961cd2f5dc1" boundaryCondition="false" compartment="metaComp">**

**<notes>**

**<body xmlns="http://www.w3.org/1999/xhtml">**

**<p>FORMULA: C15H29O7P1</p>**

**<p>CHARGE: 0</p>**

**<p>CHEBI: CHEBI:57970</p>**

**</body>**

**</notes>**

**<annotation>**

**<rdf:RDF xmlns:rdf="http://www.w3.org/1999/02/22-rdf-syntax-ns#" xmlns:bqmodel="http://biomodels.net/model-qualifiers/" xmlns:bqbiol="http://biomodels.net/biology-qualifiers/">**

**<rdf:Description rdf:about="_68a2417c-ee5a-44e5-b650-1961cd2f5dc1">**

**<bqbiol:is>**

**<rdf:Bag>**

**<rdf:li rdf:resource="http://identifiers.org/CHEBI/CHEBI:57970"/></rdf:Bag></bqbiol:is></rdf:Description></rdf:RDF>**

**</annotation>**

**</species>**

**<species id="M_10fthf6glu" constant="false" hasOnlySubstanceUnits="false" name="10-formyltetrahydrofolate-[Glu](6)" metaid="60a1bb0e-d933-48d5-87de-5a70e43348f3" boundaryCondition="false" compartment="metaComp">**

**<notes>**

**<body xmlns="http://www.w3.org/1999/xhtml">**

**<p>FORMULA: C45H51N12O22</p>**

**<p>CHARGE: 0</p>**

**<p>INCHI: InChI=1/C45H58N12O22/c46-45-55-36-35(38(67)56-45)48-21(17-47-36)18-57(19-58)22-3-1-20(2-4-22)37(66)54-28(44(78)79)9-15-33(63)52-26(42(74)75)7-13-31(61)50-24(40(70)71)5-11-29(59)49-23(39(68)69)6-12-30(60)51-25(41(72)73)8-14-32(62)53-27(43(76)77)10-16-34(64)65/h1-4,19,21,23-28,48H,5-18H2,(H,49,59)(H,50,61)(H,51,60)(H,52,63)(H,53,62)(H,54,66)(H,64,65)(H,68,69)(H,70,71)(H,72,73)(H,74,75)(H,76,77)(H,78,79)(H4,46,47,55,56,67)/p-7/t21?,23?,24?,25?,26?,27?,28-/m1/s1</p>**

**</body>**

**</notes>**

**<annotation>**

**<rdf:RDF xmlns:rdf="http://www.w3.org/1999/02/22-rdf-syntax-ns#" xmlns:bqmodel="http://biomodels.net/model-qualifiers/" xmlns:bqbiol="http://biomodels.net/biology-qualifiers/">**

**<rdf:Description rdf:about="_60a1bb0e-d933-48d5-87de-5a70e43348f3">**

**<bqbiol:is>**

**<rdf:Bag>**

**<rdf:li rdf:resource="http://identifiers.org/INCHI/InChI=1/C45H58N12O22/c46-45-55-36-35(38(67)56-45)48-21(17-47-36)18-57(19-58)22-3-1-20(2-4-22)37(66)54-28(44(78)79)9-15-33(63)52-26(42(74)75)7-13-31(61)50-24(40(70)71)5-11-29(59)49-23(39(68)69)6-12-30(60)51-25(41(72)73)8-14-32(62)53-27(43(76)77)10-16-34(64)65/h1-4,19,21,23-28,48H,5-18H2,(H,49,59)(H,50,61)(H,51,60)(H,52,63)(H,53,62)(H,54,66)(H,64,65)(H,68,69)(H,70,71)(H,72,73)(H,74,75)(H,76,77)(H,78,79)(H4,46,47,55,56,67)/p-7/t21?,23?,24?,25?,26?,27?,28-/m1/s1"/></rdf:Bag></bqbiol:is></rdf:Description></rdf:RDF>**

**</annotation>**

**</species>**

**<species id="M_CE5794" constant="false" hasOnlySubstanceUnits="false" name="neuromedin B" metaid="be61b0b6-fcb9-4fef-94b5-6fe3889a12fd" boundaryCondition="false" compartment="metaComp">**

**<notes>**

**<body xmlns="http://www.w3.org/1999/xhtml">**

**<p>FORMULA: C52H74N15O12S</p>**

**<p>CHARGE: 0</p>**

**<p>PUBCHEM.COMPOUND: 53481579</p>**

**<p>INCHIKEY: YPFNACALNKVZNK-ICQOWDAWSA-N</p>**

**<p>HMDB: HMDB13018</p>**

**<p>INCHI: InChI=1S/C52H73N15O12S/c1-27(2)17-36(64-51(78)40(21-41(54)69)61-42(70)22-53)48(75)66-38(19-31-23-57-34-14-10-9-13-33(31)34)47(74)60-28(3)46(73)67-44(29(4)68)52(79)58-25-43(71)62-39(20-32-24-56-26-59-32)50(77)65-37(18-30-11-7-6-8-12-30)49(76)63-35(45(55)72)15-16-80-5/h6-14,23-24,26-29,35-40,44,57,68H,15-22,25,53H2,1-5H3,(H2,54,69)(H2,55,72)(H,56,59)(H,58,79)(H,60,74)(H,61,70)(H,62,71)(H,63,76)(H,64,78)(H,65,77)(H,66,75)(H,67,73)/t28-,29-,35+,36+,37-,38-,39+,40-,44+/m1/s1</p>**

**</body>**

**</notes>**

**<annotation>**

**<rdf:RDF xmlns:rdf="http://www.w3.org/1999/02/22-rdf-syntax-ns#" xmlns:bqmodel="http://biomodels.net/model-qualifiers/" xmlns:bqbiol="http://biomodels.net/biology-qualifiers/">**

**<rdf:Description rdf:about="be61b0b6-fcb9-4fef-94b5-6fe3889a12fd">**

**<bqbiol:is>**

**<rdf:Bag>**

**<rdf:li rdf:resource="http://identifiers.org/PUBCHEM.COMPOUND/53481579"/></rdf:Bag></bqbiol:is><bqbiol:is>**

**<rdf:Bag>**

**<rdf:li rdf:resource="http://identifiers.org/INCHIKEY/YPFNACALNKVZNK-ICQOWDAWSA-N"/></rdf:Bag></bqbiol:is><bqbiol:is>**

**<rdf:Bag>**

**<rdf:li rdf:resource="http://identifiers.org/HMDB/HMDB13018"/></rdf:Bag></bqbiol:is><bqbiol:is>**

**<rdf:Bag>**

**<rdf:li rdf:resource="http://identifiers.org/INCHI/InChI=1S/C52H73N15O12S/c1-27(2)17-36(64-51(78)40(21-41(54)69)61-42(70)22-53)48(75)66-38(19-31-23-57-34-14-10-9-13-33(31)34)47(74)60-28(3)46(73)67-44(29(4)68)52(79)58-25-43(71)62-39(20-32-24-56-26-59-32)50(77)65-37(18-30-11-7-6-8-12-30)49(76)63-35(45(55)72)15-16-80-5/h6-14,23-24,26-29,35-40,44,57,68H,15-22,25,53H2,1-5H3,(H2,54,69)(H2,55,72)(H,56,59)(H,58,79)(H,60,74)(H,61,70)(H,62,71)(H,63,76)(H,64,78)(H,65,77)(H,66,75)(H,67,73)/t28-,29-,35+,36+,37-,38-,39+,40-,44+/m1/s1"/></rdf:Bag></bqbiol:is></rdf:Description></rdf:RDF>**

**</annotation>**

**</species>**

**<species id="M_CE5796" constant="false" hasOnlySubstanceUnits="false" name="neuromedin B (4-10)" metaid="a287dee2-9193-4ec7-b1c3-feee3b5c7200" boundaryCondition="false" compartment="metaComp">**

**<notes>**

**<body xmlns="http://www.w3.org/1999/xhtml">**

**<p>FORMULA: C40H54N11O8S</p>**

**<p>CHARGE: 0</p>**

**<p>PUBCHEM.COMPOUND: 53481578</p>**

**<p>INCHIKEY: MVOFLIKDVHKCBK-FVNKCRITSA-N</p>**

**<p>HMDB: HMDB13017</p>**

**<p>INCHI: InChI=1S/C40H53N11O8S/c1-22(47-37(56)28(41)16-25-18-44-29-12-8-7-11-27(25)29)36(55)51-34(23(2)52)40(59)45-20-33(53)48-32(17-26-19-43-21-46-26)39(58)50-31(15-24-9-5-4-6-10-24)38(57)49-30(35(42)54)13-14-60-3/h4-12,18-19,21-23,28,30-32,34,44,52H,13-17,20,41H2,1-3H3,(H2,42,54)(H,43,46)(H,45,59)(H,47,56)(H,48,53)(H,49,57)(H,50,58)(H,51,55)/t22-,23-,28-,30-,31+,32-,34-/m1/s1</p>**

**</body>**

**</notes>**

**<annotation>**

**<rdf:RDF xmlns:rdf="http://www.w3.org/1999/02/22-rdf-syntax-ns#" xmlns:bqmodel="http://biomodels.net/model-qualifiers/" xmlns:bqbiol="http://biomodels.net/biology-qualifiers/">**

**<rdf:Description rdf:about="a287dee2-9193-4ec7-b1c3-feee3b5c7200">**

**<bqbiol:is>**

**<rdf:Bag>**

**<rdf:li rdf:resource="http://identifiers.org/PUBCHEM.COMPOUND/53481578"/></rdf:Bag></bqbiol:is><bqbiol:is>**

**<rdf:Bag>**

**<rdf:li rdf:resource="http://identifiers.org/INCHIKEY/MVOFLIKDVHKCBK-FVNKCRITSA-N"/></rdf:Bag></bqbiol:is><bqbiol:is>**

**<rdf:Bag>**

**<rdf:li rdf:resource="http://identifiers.org/HMDB/HMDB13017"/></rdf:Bag></bqbiol:is><bqbiol:is>**

**<rdf:Bag>**

**<rdf:li rdf:resource="http://identifiers.org/INCHI/InChI=1S/C40H53N11O8S/c1-22(47-37(56)28(41)16-25-18-44-29-12-8-7-11-27(25)29)36(55)51-34(23(2)52)40(59)45-20-33(53)48-32(17-26-19-43-21-46-26)39(58)50-31(15-24-9-5-4-6-10-24)38(57)49-30(35(42)54)13-14-60-3/h4-12,18-19,21-23,28,30-32,34,44,52H,13-17,20,41H2,1-3H3,(H2,42,54)(H,43,46)(H,45,59)(H,47,56)(H,48,53)(H,49,57)(H,50,58)(H,51,55)/t22-,23-,28-,30-,31+,32-,34-/m1/s1"/></rdf:Bag></bqbiol:is></rdf:Description></rdf:RDF>**

**</annotation>**

**</species>**

**<species id="M_CE5795" constant="false" hasOnlySubstanceUnits="false" name="neuromedin B (1-3)" metaid="2531fc63-9b1b-423b-bade-42632e61d7ab" boundaryCondition="false" compartment="metaComp">**

**<notes>**

**<body xmlns="http://www.w3.org/1999/xhtml">**

**<p>FORMULA: C12H22N4O5</p>**

**<p>CHARGE: 0</p>**

**<p>PUBCHEM.COMPOUND: 53481577</p>**

**<p>INCHIKEY: JVWPPCWUDRJGAE-JGVFFNPUSA-N</p>**

**<p>HMDB: HMDB13016</p>**

**<p>INCHI: InChI=1S/C12H22N4O5/c1-6(2)3-8(12(20)21)16-11(19)7(4-9(14)17)15-10(18)5-13/h6-8H,3-5,13H2,1-2H3,(H2,14,17)(H,15,18)(H,16,19)(H,20,21)/t7-,8+/m0/s1</p>**

**</body>**

**</notes>**

**<annotation>**

**<rdf:RDF xmlns:rdf="http://www.w3.org/1999/02/22-rdf-syntax-ns#" xmlns:bqmodel="http://biomodels.net/model-qualifiers/" xmlns:bqbiol="http://biomodels.net/biology-qualifiers/">**

**<rdf:Description rdf:about="_2531fc63-9b1b-423b-bade-42632e61d7ab">**

**<bqbiol:is>**

**<rdf:Bag>**

**<rdf:li rdf:resource="http://identifiers.org/PUBCHEM.COMPOUND/53481577"/></rdf:Bag></bqbiol:is><bqbiol:is>**

**<rdf:Bag>**

**<rdf:li rdf:resource="http://identifiers.org/INCHIKEY/JVWPPCWUDRJGAE-JGVFFNPUSA-N"/></rdf:Bag></bqbiol:is><bqbiol:is>**

**<rdf:Bag>**

**<rdf:li rdf:resource="http://identifiers.org/HMDB/HMDB13016"/></rdf:Bag></bqbiol:is><bqbiol:is>**

**<rdf:Bag>**

**<rdf:li rdf:resource="http://identifiers.org/INCHI/InChI=1S/C12H22N4O5/c1-6(2)3-8(12(20)21)16-11(19)7(4-9(14)17)15-10(18)5-13/h6-8H,3-5,13H2,1-2H3,(H2,14,17)(H,15,18)(H,16,19)(H,20,21)/t7-,8+/m0/s1"/></rdf:Bag></bqbiol:is></rdf:Description></rdf:RDF>**

**</annotation>**

**</species>**

**<species id="M_c81coa" constant="false" hasOnlySubstanceUnits="false" name="octenoylcoa" metaid="62ad9ed8-267b-40a6-80fb-7f012f853711" boundaryCondition="false" compartment="metaComp">**

**<notes>**

**<body xmlns="http://www.w3.org/1999/xhtml">**

**<p>FORMULA: C29H44N7O17P3S</p>**

**<p>CHARGE: 0</p>**

**<p>PUBCHEM.COMPOUND: 5280769</p>**

**<p>KEGG.COMPOUND: C05276</p>**

**<p>HMDB: HMDB03949</p>**

**<p>CHEBI: CHEBI:27537</p>**

**<p>INCHI: InChI=1S/C29H48N7O17P3S/c1-4-5-6-7-8-9-20(38)57-13-12-31-19(37)10-11-32-27(41)24(40)29(2,3)15-50-56(47,48)53-55(45,46)49-14-18-23(52-54(42,43)44)22(39)28(51-18)36-17-35-21-25(30)33-16-34-26(21)36/h8-9,16-18,22-24,28,39-40H,4-7,10-15H2,1-3H3,(H,31,37)(H,32,41)(H,45,46)(H,47,48)(H2,30,33,34)(H2,42,43,44)/b9-8+/t18-,22-,23-,24+,28-/m1/s1 || InChI=1S/C29H48N7O17P3S/c1-4-5-6-7-8-9-20(38)57-13-12-31-19(37)10-11-32-27(41)24(40)29(2,3)15-50-56(47,48)53-55(45,46)49-14-18-23(52-54(42,43)44)22(39)28(51-18)36-17-35-21-25(30)33-16-34-26(21)36/h8-9,16-18,22-24,28,39-40H,4-7,10-15H2,1-3H3,(H,31,37)(H,32,41)(H,45,46)(H,47,48)(H2,30,33,34)(H2,42,43,44)/b9-8+/t18-,22-,23-,24?,28-/m1/s1</p>**

**</body>**

**</notes>**

**<annotation>**

**<rdf:RDF xmlns:rdf="http://www.w3.org/1999/02/22-rdf-syntax-ns#" xmlns:bqmodel="http://biomodels.net/model-qualifiers/" xmlns:bqbiol="http://biomodels.net/biology-qualifiers/">**

**<rdf:Description rdf:about="_62ad9ed8-267b-40a6-80fb-7f012f853711">**

**<bqbiol:is>**

**<rdf:Bag>**

**<rdf:li rdf:resource="http://identifiers.org/PUBCHEM.COMPOUND/5280769"/></rdf:Bag></bqbiol:is><bqbiol:is>**

**<rdf:Bag>**

**<rdf:li rdf:resource="http://identifiers.org/KEGG.COMPOUND/C05276"/></rdf:Bag></bqbiol:is><bqbiol:is>**

**<rdf:Bag>**

**<rdf:li rdf:resource="http://identifiers.org/HMDB/HMDB03949"/></rdf:Bag></bqbiol:is><bqbiol:is>**

**<rdf:Bag>**

**<rdf:li rdf:resource="http://identifiers.org/CHEBI/CHEBI:27537"/></rdf:Bag></bqbiol:is><bqbiol:is>**

**<rdf:Bag>**

**<rdf:li rdf:resource="http://identifiers.org/INCHI/InChI=1S/C29H48N7O17P3S/c1-4-5-6-7-8-9-20(38)57-13-12-31-19(37)10-11-32-27(41)24(40)29(2,3)15-50-56(47,48)53-55(45,46)49-14-18-23(52-54(42,43)44)22(39)28(51-18)36-17-35-21-25(30)33-16-34-26(21)36/h8-9,16-18,22-24,28,39-40H,4-7,10-15H2,1-3H3,(H,31,37)(H,32,41)(H,45,46)(H,47,48)(H2,30,33,34)(H2,42,43,44)/b9-8+/t18-,22-,23-,24+,28-/m1/s1"/></rdf:Bag></bqbiol:is><bqbiol:is>**

**<rdf:Bag>**

**<rdf:li rdf:resource="http://identifiers.org/INCHI/InChI=1S/C29H48N7O17P3S/c1-4-5-6-7-8-9-20(38)57-13-12-31-19(37)10-11-32-27(41)24(40)29(2,3)15-50-56(47,48)53-55(45,46)49-14-18-23(52-54(42,43)44)22(39)28(51-18)36-17-35-21-25(30)33-16-34-26(21)36/h8-9,16-18,22-24,28,39-40H,4-7,10-15H2,1-3H3,(H,31,37)(H,32,41)(H,45,46)(H,47,48)(H2,30,33,34)(H2,42,43,44)/b9-8+/t18-,22-,23-,24?,28-/m1/s1"/></rdf:Bag></bqbiol:is></rdf:Description></rdf:RDF>**

**</annotation>**

**</species>**

**<species id="M_CE5798" constant="false" hasOnlySubstanceUnits="false" name="neuromedin N (1-4)" metaid="9835faf7-d9ca-46da-ac6e-81d83b5e4317" boundaryCondition="false" compartment="metaComp">**

**<notes>**

**<body xmlns="http://www.w3.org/1999/xhtml">**

**<p>FORMULA: C26H40N4O6</p>**

**<p>CHARGE: 0</p>**

**<p>PUBCHEM.COMPOUND: 53481582</p>**

**<p>INCHIKEY: SKGLAZSLOGYCCA-PEFXOJROSA-N</p>**

**<p>HMDB: HMDB13021</p>**

**<p>INCHI: InChI=1S/C26H40N4O6/c1-5-15(3)21(27)25(34)30-13-7-8-20(30)24(33)28-19(14-17-9-11-18(31)12-10-17)23(32)29-22(26(35)36)16(4)6-2/h9-12,15-16,19-22,31H,5-8,13-14,27H2,1-4H3,(H,28,33)(H,29,32)(H,35,36)/t15-,16-,19+,20+,21-,22-/m1/s1</p>**

**</body>**

**</notes>**

**<annotation>**

**<rdf:RDF xmlns:rdf="http://www.w3.org/1999/02/22-rdf-syntax-ns#" xmlns:bqmodel="http://biomodels.net/model-qualifiers/" xmlns:bqbiol="http://biomodels.net/biology-qualifiers/">**

**<rdf:Description rdf:about="_9835faf7-d9ca-46da-ac6e-81d83b5e4317">**

**<bqbiol:is>**

**<rdf:Bag>**

**<rdf:li rdf:resource="http://identifiers.org/PUBCHEM.COMPOUND/53481582"/></rdf:Bag></bqbiol:is><bqbiol:is>**

**<rdf:Bag>**

**<rdf:li rdf:resource="http://identifiers.org/INCHIKEY/SKGLAZSLOGYCCA-PEFXOJROSA-N"/></rdf:Bag></bqbiol:is><bqbiol:is>**

**<rdf:Bag>**

**<rdf:li rdf:resource="http://identifiers.org/HMDB/HMDB13021"/></rdf:Bag></bqbiol:is><bqbiol:is>**

**<rdf:Bag>**

**<rdf:li rdf:resource="http://identifiers.org/INCHI/InChI=1S/C26H40N4O6/c1-5-15(3)21(27)25(34)30-13-7-8-20(30)24(33)28-19(14-17-9-11-18(31)12-10-17)23(32)29-22(26(35)36)16(4)6-2/h9-12,15-16,19-22,31H,5-8,13-14,27H2,1-4H3,(H,28,33)(H,29,32)(H,35,36)/t15-,16-,19+,20+,21-,22-/m1/s1"/></rdf:Bag></bqbiol:is></rdf:Description></rdf:RDF>**

**</annotation>**

**</species>**

**<species id="M_fmn" constant="false" hasOnlySubstanceUnits="false" name="FMN" metaid="8df8b580-02d1-4e30-920b-b1d35637d704" boundaryCondition="false" compartment="metaComp">**

**<notes>**

**<body xmlns="http://www.w3.org/1999/xhtml">**

**<p>FORMULA: C17H19N4O9P</p>**

**<p>CHARGE: 0</p>**

**<p>PUBCHEM.COMPOUND: 643976</p>**

**<p>KEGG.COMPOUND: C00061</p>**

**<p>HMDB: HMDB01520</p>**

**<p>CHEBI: CHEBI:58210 || CHEBI:17621</p>**

**<p>INCHI: InChI=1S/C17H21N4O9P/c1-7-3-9-10(4-8(7)2)21(15-13(18-9)16(25)20-17(26)19-15)5-11(22)14(24)12(23)6-30-31(27,28)29/h3-4,11-12,14,22-24H,5-6H2,1-2H3,(H3,20,25,26,27,28,29)/p-3/t11-,12+,14-/m0/s1 || InChI=1S/C17H21N4O9P/c1-7-3-9-10(4-8(7)2)21(15-13(18-9)16(25)20-17(26)19-15)5-11(22)14(24)12(23)6-30-31(27,28)29/h3-4,11-12,14,22-24H,5-6H2,1-2H3,(H,20,25,26)(H2,27,28,29)/t11-,12+,14-/m0/s1</p>**

**</body>**

**</notes>**

**<annotation>**

**<rdf:RDF xmlns:rdf="http://www.w3.org/1999/02/22-rdf-syntax-ns#" xmlns:bqmodel="http://biomodels.net/model-qualifiers/" xmlns:bqbiol="http://biomodels.net/biology-qualifiers/">**

**<rdf:Description rdf:about="_8df8b580-02d1-4e30-920b-b1d35637d704">**

**<bqbiol:is>**

**<rdf:Bag>**

**<rdf:li rdf:resource="http://identifiers.org/PUBCHEM.COMPOUND/643976"/></rdf:Bag></bqbiol:is><bqbiol:is>**

**<rdf:Bag>**

**<rdf:li rdf:resource="http://identifiers.org/KEGG.COMPOUND/C00061"/></rdf:Bag></bqbiol:is><bqbiol:is>**

**<rdf:Bag>**

**<rdf:li rdf:resource="http://identifiers.org/HMDB/HMDB01520"/></rdf:Bag></bqbiol:is><bqbiol:is>**

**<rdf:Bag>**

**<rdf:li rdf:resource="http://identifiers.org/CHEBI/CHEBI:58210"/></rdf:Bag></bqbiol:is><bqbiol:is>**

**<rdf:Bag>**

**<rdf:li rdf:resource="http://identifiers.org/CHEBI/CHEBI:17621"/></rdf:Bag></bqbiol:is><bqbiol:is>**

**<rdf:Bag>**

**<rdf:li rdf:resource="http://identifiers.org/INCHI/InChI=1S/C17H21N4O9P/c1-7-3-9-10(4-8(7)2)21(15-13(18-9)16(25)20-17(26)19-15)5-11(22)14(24)12(23)6-30-31(27,28)29/h3-4,11-12,14,22-24H,5-6H2,1-2H3,(H3,20,25,26,27,28,29)/p-3/t11-,12+,14-/m0/s1"/></rdf:Bag></bqbiol:is><bqbiol:is>**

**<rdf:Bag>**

**<rdf:li rdf:resource="http://identifiers.org/INCHI/InChI=1S/C17H21N4O9P/c1-7-3-9-10(4-8(7)2)21(15-13(18-9)16(25)20-17(26)19-15)5-11(22)14(24)12(23)6-30-31(27,28)29/h3-4,11-12,14,22-24H,5-6H2,1-2H3,(H,20,25,26)(H2,27,28,29)/t11-,12+,14-/m0/s1"/></rdf:Bag></bqbiol:is></rdf:Description></rdf:RDF>**

**</annotation>**

**</species>**

**<species id="M_CE5797" constant="false" hasOnlySubstanceUnits="false" name="neuromedin N" metaid="33ed9349-4efc-4a9e-80a0-ed795998b60b" boundaryCondition="false" compartment="metaComp">**

**<notes>**

**<body xmlns="http://www.w3.org/1999/xhtml">**

**<p>FORMULA: C32H51N5O7</p>**

**<p>CHARGE: 0</p>**

**<p>PUBCHEM.COMPOUND: 53481583</p>**

**<p>INCHIKEY: NSLIVCMCAULZET-WFJLUAAKSA-N</p>**

**<p>HMDB: HMDB13022</p>**

**<p>INCHI: InChI=1S/C32H51N5O7/c1-7-19(5)26(33)31(42)37-15-9-10-25(37)29(40)34-23(17-21-11-13-22(38)14-12-21)28(39)36-27(20(6)8-2)30(41)35-24(32(43)44)16-18(3)4/h11-14,18-20,23-27,38H,7-10,15-17,33H2,1-6H3,(H,34,40)(H,35,41)(H,36,39)(H,43,44)/t19-,20-,23+,24+,25+,26-,27-/m1/s1</p>**

**</body>**

**</notes>**

**<annotation>**

**<rdf:RDF xmlns:rdf="http://www.w3.org/1999/02/22-rdf-syntax-ns#" xmlns:bqmodel="http://biomodels.net/model-qualifiers/" xmlns:bqbiol="http://biomodels.net/biology-qualifiers/">**

**<rdf:Description rdf:about="_33ed9349-4efc-4a9e-80a0-ed795998b60b">**

**<bqbiol:is>**

**<rdf:Bag>**

**<rdf:li rdf:resource="http://identifiers.org/PUBCHEM.COMPOUND/53481583"/></rdf:Bag></bqbiol:is><bqbiol:is>**

**<rdf:Bag>**

**<rdf:li rdf:resource="http://identifiers.org/INCHIKEY/NSLIVCMCAULZET-WFJLUAAKSA-N"/></rdf:Bag></bqbiol:is><bqbiol:is>**

**<rdf:Bag>**

**<rdf:li rdf:resource="http://identifiers.org/HMDB/HMDB13022"/></rdf:Bag></bqbiol:is><bqbiol:is>**

**<rdf:Bag>**

**<rdf:li rdf:resource="http://identifiers.org/INCHI/InChI=1S/C32H51N5O7/c1-7-19(5)26(33)31(42)37-15-9-10-25(37)29(40)34-23(17-21-11-13-22(38)14-12-21)28(39)36-27(20(6)8-2)30(41)35-24(32(43)44)16-18(3)4/h11-14,18-20,23-27,38H,7-10,15-17,33H2,1-6H3,(H,34,40)(H,35,41)(H,36,39)(H,43,44)/t19-,20-,23+,24+,25+,26-,27-/m1/s1"/></rdf:Bag></bqbiol:is></rdf:Description></rdf:RDF>**

**</annotation>**

**</species>**

**<species id="M_g1m7masnB" constant="false" hasOnlySubstanceUnits="false" name="glucosyl-(alpha-D-mannosyl)7-beta-D-mannosyl-diacetylchitobiosyl-L-asparagine, isoform B (protein)" metaid="c3efd317-a0ec-4fbd-b328-85fcefe5fef5" boundaryCondition="false" compartment="metaComp">**

**<notes>**

**<body xmlns="http://www.w3.org/1999/xhtml">**

**<p>FORMULA: C70H117N2O55X</p>**

**<p>CHARGE: 0</p>**

**</body>**

**</notes>**

**<annotation>**

**<rdf:RDF xmlns:rdf="http://www.w3.org/1999/02/22-rdf-syntax-ns#" xmlns:bqmodel="http://biomodels.net/model-qualifiers/" xmlns:bqbiol="http://biomodels.net/biology-qualifiers/">**

**<rdf:Description rdf:about="c3efd317-a0ec-4fbd-b328-85fcefe5fef5"/></rdf:RDF>**

**</annotation>**

**</species>**

**<species id="M_sql" constant="false" hasOnlySubstanceUnits="false" name="squalene" metaid="12736eff-eb03-4e9f-b9a3-657ad0df54db" boundaryCondition="false" compartment="metaComp">**

**<notes>**

**<body xmlns="http://www.w3.org/1999/xhtml">**

**<p>FORMULA: C30H50</p>**

**<p>CHARGE: 0</p>**

**<p>PUBCHEM.COMPOUND: 25244109 || 638072 || 1105 || 11975273</p>**

**<p>INCHIKEY: YYGNTYWPHWGJRM-AAJYLUCBSA-N</p>**

**<p>KEGG.COMPOUND: C00751</p>**

**<p>HMDB: HMDB00256</p>**

**<p>CHEBI: CHEBI:15440</p>**

**<p>INCHI: InChI=1S/C30H50/c1-25(2)15-11-19-29(7)23-13-21-27(5)17-9-10-18-28(6)22-14-24-30(8)20-12-16-26(3)4/h15-18,23-24H,9-14,19-22H2,1-8H3/b27-17+,28-18+,29-23+,30-24+ || InChI=1S/C30H50/c1-25(2)15-11-19-29(7)23-13-21-27(5)17-9-10-18-28(6)22-14-24-30(8)20-12-16-26(3)4/h15-18,23-24H,9-14,19-22H2,1-8H3/b27-17+,28-18+,29-23-,30-24+</p>**

**</body>**

**</notes>**

**<annotation>**

**<rdf:RDF xmlns:rdf="http://www.w3.org/1999/02/22-rdf-syntax-ns#" xmlns:bqmodel="http://biomodels.net/model-qualifiers/" xmlns:bqbiol="http://biomodels.net/biology-qualifiers/">**

**<rdf:Description rdf:about="_12736eff-eb03-4e9f-b9a3-657ad0df54db">**

**<bqbiol:is>**

**<rdf:Bag>**

**<rdf:li rdf:resource="http://identifiers.org/PUBCHEM.COMPOUND/25244109"/></rdf:Bag></bqbiol:is><bqbiol:is>**

**<rdf:Bag>**

**<rdf:li rdf:resource="http://identifiers.org/PUBCHEM.COMPOUND/638072"/></rdf:Bag></bqbiol:is><bqbiol:is>**

**<rdf:Bag>**

**<rdf:li rdf:resource="http://identifiers.org/PUBCHEM.COMPOUND/1105"/></rdf:Bag></bqbiol:is><bqbiol:is>**

**<rdf:Bag>**

**<rdf:li rdf:resource="http://identifiers.org/PUBCHEM.COMPOUND/11975273"/></rdf:Bag></bqbiol:is><bqbiol:is>**

**<rdf:Bag>**

**<rdf:li rdf:resource="http://identifiers.org/INCHIKEY/YYGNTYWPHWGJRM-AAJYLUCBSA-N"/></rdf:Bag></bqbiol:is><bqbiol:is>**

**<rdf:Bag>**

**<rdf:li rdf:resource="http://identifiers.org/KEGG.COMPOUND/C00751"/></rdf:Bag></bqbiol:is><bqbiol:is>**

**<rdf:Bag>**

**<rdf:li rdf:resource="http://identifiers.org/HMDB/HMDB00256"/></rdf:Bag></bqbiol:is><bqbiol:is>**

**<rdf:Bag>**

**<rdf:li rdf:resource="http://identifiers.org/CHEBI/CHEBI:15440"/></rdf:Bag></bqbiol:is><bqbiol:is>**

**<rdf:Bag>**

**<rdf:li rdf:resource="http://identifiers.org/INCHI/InChI=1S/C30H50/c1-25(2)15-11-19-29(7)23-13-21-27(5)17-9-10-18-28(6)22-14-24-30(8)20-12-16-26(3)4/h15-18,23-24H,9-14,19-22H2,1-8H3/b27-17+,28-18+,29-23+,30-24+"/></rdf:Bag></bqbiol:is><bqbiol:is>**

**<rdf:Bag>**

**<rdf:li rdf:resource="http://identifiers.org/INCHI/InChI=1S/C30H50/c1-25(2)15-11-19-29(7)23-13-21-27(5)17-9-10-18-28(6)22-14-24-30(8)20-12-16-26(3)4/h15-18,23-24H,9-14,19-22H2,1-8H3/b27-17+,28-18+,29-23-,30-24+"/></rdf:Bag></bqbiol:is></rdf:Description></rdf:RDF>**

**</annotation>**

**</species>**

**<species id="M_CE5791" constant="false" hasOnlySubstanceUnits="false" name="kinetensin 4-8" metaid="12f0a122-6507-4940-b078-319103196db8" boundaryCondition="false" compartment="metaComp">**

**<notes>**

**<body xmlns="http://www.w3.org/1999/xhtml">**

**<p>FORMULA: C35H47N10O7</p>**

**<p>CHARGE: 0</p>**

**<p>PUBCHEM.COMPOUND: 53481568</p>**

**<p>INCHIKEY: CCANVONJZOLPKG-MJXUZWQSSA-N</p>**

**<p>HMDB: HMDB12987</p>**

**<p>INCHI: InChI=1S/C35H46N10O7/c36-25(8-4-14-40-35(37)38)30(47)43-27(18-23-19-39-20-41-23)33(50)45-15-5-9-29(45)32(49)42-26(16-22-10-12-24(46)13-11-22)31(48)44-28(34(51)52)17-21-6-2-1-3-7-21/h1-3,6-7,10-13,19-20,25-29,46H,4-5,8-9,14-18,36H2,(H,39,41)(H,42,49)(H,43,47)(H,44,48)(H,51,52)(H4,37,38,40)/t25-,26-,27+,28+,29-/m1/s1</p>**

**</body>**

**</notes>**

**<annotation>**

**<rdf:RDF xmlns:rdf="http://www.w3.org/1999/02/22-rdf-syntax-ns#" xmlns:bqmodel="http://biomodels.net/model-qualifiers/" xmlns:bqbiol="http://biomodels.net/biology-qualifiers/">**

**<rdf:Description rdf:about="_12f0a122-6507-4940-b078-319103196db8">**

**<bqbiol:is>**

**<rdf:Bag>**

**<rdf:li rdf:resource="http://identifiers.org/PUBCHEM.COMPOUND/53481568"/></rdf:Bag></bqbiol:is><bqbiol:is>**

**<rdf:Bag>**

**<rdf:li rdf:resource="http://identifiers.org/INCHIKEY/CCANVONJZOLPKG-MJXUZWQSSA-N"/></rdf:Bag></bqbiol:is><bqbiol:is>**

**<rdf:Bag>**

**<rdf:li rdf:resource="http://identifiers.org/HMDB/HMDB12987"/></rdf:Bag></bqbiol:is><bqbiol:is>**

**<rdf:Bag>**

**<rdf:li rdf:resource="http://identifiers.org/INCHI/InChI=1S/C35H46N10O7/c36-25(8-4-14-40-35(37)38)30(47)43-27(18-23-19-39-20-41-23)33(50)45-15-5-9-29(45)32(49)42-26(16-22-10-12-24(46)13-11-22)31(48)44-28(34(51)52)17-21-6-2-1-3-7-21/h1-3,6-7,10-13,19-20,25-29,46H,4-5,8-9,14-18,36H2,(H,39,41)(H,42,49)(H,43,47)(H,44,48)(H,51,52)(H4,37,38,40)/t25-,26-,27+,28+,29-/m1/s1"/></rdf:Bag></bqbiol:is></rdf:Description></rdf:RDF>**

**</annotation>**

**</species>**

**<species id="M_lxser" constant="false" hasOnlySubstanceUnits="false" name="Gal-Xyl-L-Ser (protein)" metaid="988c9ace-26e4-4d39-a26a-5c75a9ea7483" boundaryCondition="false" compartment="metaComp">**

**<notes>**

**<body xmlns="http://www.w3.org/1999/xhtml">**

**<p>FORMULA: C11H19O9X</p>**

**<p>CHARGE: 0</p>**

**<p>KEGG.COMPOUND: C01287</p>**

**</body>**

**</notes>**

**<annotation>**

**<rdf:RDF xmlns:rdf="http://www.w3.org/1999/02/22-rdf-syntax-ns#" xmlns:bqmodel="http://biomodels.net/model-qualifiers/" xmlns:bqbiol="http://biomodels.net/biology-qualifiers/">**

**<rdf:Description rdf:about="_988c9ace-26e4-4d39-a26a-5c75a9ea7483">**

**<bqbiol:is>**

**<rdf:Bag>**

**<rdf:li rdf:resource="http://identifiers.org/KEGG.COMPOUND/C01287"/></rdf:Bag></bqbiol:is></rdf:Description></rdf:RDF>**

**</annotation>**

**</species>**

**<species id="M_g1m7masnC" constant="false" hasOnlySubstanceUnits="false" name="glucosyl-(alpha-D-mannosyl)7-beta-D-mannosyl-diacetylchitobiosyl-L-asparagine, isoform C (protein)" metaid="d3de340d-d69b-45cf-bccb-576a61abdb8f" boundaryCondition="false" compartment="metaComp">**

**<notes>**

**<body xmlns="http://www.w3.org/1999/xhtml">**

**<p>FORMULA: C70H117N2O55X</p>**

**<p>CHARGE: 0</p>**

**</body>**

**</notes>**

**<annotation>**

**<rdf:RDF xmlns:rdf="http://www.w3.org/1999/02/22-rdf-syntax-ns#" xmlns:bqmodel="http://biomodels.net/model-qualifiers/" xmlns:bqbiol="http://biomodels.net/biology-qualifiers/">**

**<rdf:Description rdf:about="d3de340d-d69b-45cf-bccb-576a61abdb8f"/></rdf:RDF>**

**</annotation>**

**</species>**

**<species id="M_CE6027" constant="false" hasOnlySubstanceUnits="false" name="24,25,26,27-tetranor-23-oxo-hydroxyvitamin D3" metaid="5a8ce7cb-882a-41fb-9ab1-140e6d877965" boundaryCondition="false" compartment="metaComp">**

**<notes>**

**<body xmlns="http://www.w3.org/1999/xhtml">**

**<p>FORMULA: C23H34O3</p>**

**<p>CHARGE: 0</p>**

**<p>INCHI: InChI=1/C23H34O3/c1-15(10-12-24)20-8-9-21-17(5-4-11-23(20,21)3)6-7-18-13-19(25)14-22(26)16(18)2/h6-7,12,15,19-22,25-26H,2,4-5,8-11,13-14H2,1,3H3/b17-6+,18-7-/t15-,19+,20-,21+,22+,23?/m0/s1</p>**

**</body>**

**</notes>**

**<annotation>**

**<rdf:RDF xmlns:rdf="http://www.w3.org/1999/02/22-rdf-syntax-ns#" xmlns:bqmodel="http://biomodels.net/model-qualifiers/" xmlns:bqbiol="http://biomodels.net/biology-qualifiers/">**

**<rdf:Description rdf:about="_5a8ce7cb-882a-41fb-9ab1-140e6d877965">**

**<bqbiol:is>**

**<rdf:Bag>**

**<rdf:li rdf:resource="http://identifiers.org/INCHI/InChI=1/C23H34O3/c1-15(10-12-24)20-8-9-21-17(5-4-11-23(20,21)3)6-7-18-13-19(25)14-22(26)16(18)2/h6-7,12,15,19-22,25-26H,2,4-5,8-11,13-14H2,1,3H3/b17-6+,18-7-/t15-,19+,20-,21+,22+,23?/m0/s1"/></rdf:Bag></bqbiol:is></rdf:Description></rdf:RDF>**

**</annotation>**

**</species>**

**<species id="M_pect" constant="false" hasOnlySubstanceUnits="false" name="pectins" metaid="3d4c74b6-26a1-4eb0-8e9f-82ca98ff49d7" boundaryCondition="false" compartment="metaComp">**

**<notes>**

**<body xmlns="http://www.w3.org/1999/xhtml">**

**<p>FORMULA: C2535H3509O2535</p>**

**<p>CHARGE: 0</p>**

**<p>HMDB: HMDB03402</p>**

**</body>**

**</notes>**

**<annotation>**

**<rdf:RDF xmlns:rdf="http://www.w3.org/1999/02/22-rdf-syntax-ns#" xmlns:bqmodel="http://biomodels.net/model-qualifiers/" xmlns:bqbiol="http://biomodels.net/biology-qualifiers/">**

**<rdf:Description rdf:about="_3d4c74b6-26a1-4eb0-8e9f-82ca98ff49d7">**

**<bqbiol:is>**

**<rdf:Bag>**

**<rdf:li rdf:resource="http://identifiers.org/HMDB/HMDB03402"/></rdf:Bag></bqbiol:is></rdf:Description></rdf:RDF>**

**</annotation>**

**</species>**

**<species id="M_chtn" constant="false" hasOnlySubstanceUnits="false" name="chitin" metaid="f0b9a585-145d-4025-bf2d-0afa104cbc38" boundaryCondition="false" compartment="metaComp">**

**<notes>**

**<body xmlns="http://www.w3.org/1999/xhtml">**

**<p>FORMULA: C24H41N3O16</p>**

**<p>CHARGE: 0</p>**

**<p>INCHIKEY: DJHJJVWPFGHIPH-OODMECLYSA-N</p>**

**<p>HMDB: HMDB06698 || HMDB03362</p>**

**<p>CHEBI: CHEBI:17029 || CHEBI:71404</p>**

**<p>INCHI: InChI=1S/C24H41N3O16/c1-7(31)25-13-18(36)20(11(5-29)39-22(13)38)42-24-15(27-9(3)33)19(37)21(12(6-30)41-24)43-23-14(26-8(2)32)17(35)16(34)10(4-28)40-23/h10-24,28-30,34-38H,4-6H2,1-3H3,(H,25,31)(H,26,32)(H,27,33)/t10-,11-,12-,13-,14-,15-,16-,17-,18-,19-,20-,21-,22-,23+,24+/m1/s1 || InChI=1S/C28H49N3O16/c1-11(35)29-21-19(9-44-8-15-17(5-33)47-28(42)23(25(15)39)31-13(3)37)45-16(4-32)14(24(21)38)7-43-10-20-22(30-12(2)36)27(41)26(40)18(6-34)46-20/h14-28,32-34,38-42H,4-10H2,1-3H3,(H,29,35)(H,30,36)(H,31,37)/t14-,15-,16?,17?,18?,19+,20+,21?,22?,23?,24+,25+,26-,27-,28-/m1/s1</p>**

**</body>**

**</notes>**

**<annotation>**

**<rdf:RDF xmlns:rdf="http://www.w3.org/1999/02/22-rdf-syntax-ns#" xmlns:bqmodel="http://biomodels.net/model-qualifiers/" xmlns:bqbiol="http://biomodels.net/biology-qualifiers/">**

**<rdf:Description rdf:about="f0b9a585-145d-4025-bf2d-0afa104cbc38">**

**<bqbiol:is>**

**<rdf:Bag>**

**<rdf:li rdf:resource="http://identifiers.org/INCHIKEY/DJHJJVWPFGHIPH-OODMECLYSA-N"/></rdf:Bag></bqbiol:is><bqbiol:is>**

**<rdf:Bag>**

**<rdf:li rdf:resource="http://identifiers.org/HMDB/HMDB06698"/></rdf:Bag></bqbiol:is><bqbiol:is>**

**<rdf:Bag>**

**<rdf:li rdf:resource="http://identifiers.org/HMDB/HMDB03362"/></rdf:Bag></bqbiol:is><bqbiol:is>**

**<rdf:Bag>**

**<rdf:li rdf:resource="http://identifiers.org/CHEBI/CHEBI:17029"/></rdf:Bag></bqbiol:is><bqbiol:is>**

**<rdf:Bag>**

**<rdf:li rdf:resource="http://identifiers.org/CHEBI/CHEBI:71404"/></rdf:Bag></bqbiol:is><bqbiol:is>**

**<rdf:Bag>**

**<rdf:li rdf:resource="http://identifiers.org/INCHI/InChI=1S/C24H41N3O16/c1-7(31)25-13-18(36)20(11(5-29)39-22(13)38)42-24-15(27-9(3)33)19(37)21(12(6-30)41-24)43-23-14(26-8(2)32)17(35)16(34)10(4-28)40-23/h10-24,28-30,34-38H,4-6H2,1-3H3,(H,25,31)(H,26,32)(H,27,33)/t10-,11-,12-,13-,14-,15-,16-,17-,18-,19-,20-,21-,22-,23+,24+/m1/s1"/></rdf:Bag></bqbiol:is><bqbiol:is>**

**<rdf:Bag>**

**<rdf:li rdf:resource="http://identifiers.org/INCHI/InChI=1S/C28H49N3O16/c1-11(35)29-21-19(9-44-8-15-17(5-33)47-28(42)23(25(15)39)31-13(3)37)45-16(4-32)14(24(21)38)7-43-10-20-22(30-12(2)36)27(41)26(40)18(6-34)46-20/h14-28,32-34,38-42H,4-10H2,1-3H3,(H,29,35)(H,30,36)(H,31,37)/t14-,15-,16?,17?,18?,19+,20+,21?,22?,23?,24+,25+,26-,27-,28-/m1/s1"/></rdf:Bag></bqbiol:is></rdf:Description></rdf:RDF>**

**</annotation>**

**</species>**

**<species id="M_agm" constant="false" hasOnlySubstanceUnits="false" name="agmatinium(2+)" metaid="ff96e93e-3055-4a84-9551-28802c9250d0" boundaryCondition="false" compartment="metaComp">**

**<notes>**

**<body xmlns="http://www.w3.org/1999/xhtml">**

**<p>FORMULA: C5H16N4</p>**

**<p>CHARGE: 0</p>**

**<p>PUBCHEM.COMPOUND: 199</p>**

**<p>KEGG.COMPOUND: C00179</p>**

**<p>HMDB: HMDB01432</p>**

**<p>CHEBI: CHEBI:58145 || CHEBI:17431</p>**

**<p>INCHI: InChI=1S/C5H14N4/c6-3-1-2-4-9-5(7)8/h1-4,6H2,(H4,7,8,9) || InChI=1S/C5H14N4/c6-3-1-2-4-9-5(7)8/h1-4,6H2,(H4,7,8,9)/p+2</p>**

**</body>**

**</notes>**

**<annotation>**

**<rdf:RDF xmlns:rdf="http://www.w3.org/1999/02/22-rdf-syntax-ns#" xmlns:bqmodel="http://biomodels.net/model-qualifiers/" xmlns:bqbiol="http://biomodels.net/biology-qualifiers/">**

**<rdf:Description rdf:about="ff96e93e-3055-4a84-9551-28802c9250d0">**

**<bqbiol:is>**

**<rdf:Bag>**

**<rdf:li rdf:resource="http://identifiers.org/PUBCHEM.COMPOUND/199"/></rdf:Bag></bqbiol:is><bqbiol:is>**

**<rdf:Bag>**

**<rdf:li rdf:resource="http://identifiers.org/KEGG.COMPOUND/C00179"/></rdf:Bag></bqbiol:is><bqbiol:is>**

**<rdf:Bag>**

**<rdf:li rdf:resource="http://identifiers.org/HMDB/HMDB01432"/></rdf:Bag></bqbiol:is><bqbiol:is>**

**<rdf:Bag>**

**<rdf:li rdf:resource="http://identifiers.org/CHEBI/CHEBI:58145"/></rdf:Bag></bqbiol:is><bqbiol:is>**

**<rdf:Bag>**

**<rdf:li rdf:resource="http://identifiers.org/CHEBI/CHEBI:17431"/></rdf:Bag></bqbiol:is><bqbiol:is>**

**<rdf:Bag>**

**<rdf:li rdf:resource="http://identifiers.org/INCHI/InChI=1S/C5H14N4/c6-3-1-2-4-9-5(7)8/h1-4,6H2,(H4,7,8,9)"/></rdf:Bag></bqbiol:is><bqbiol:is>**

**<rdf:Bag>**

**<rdf:li rdf:resource="http://identifiers.org/INCHI/InChI=1S/C5H14N4/c6-3-1-2-4-9-5(7)8/h1-4,6H2,(H4,7,8,9)/p+2"/></rdf:Bag></bqbiol:is></rdf:Description></rdf:RDF>**

**</annotation>**

**</species>**

**<species id="M_5hoxindact" constant="false" hasOnlySubstanceUnits="false" name="(5-hydroxyindol-3-yl)acetaldehyde" metaid="3902debb-9398-4577-a3d7-61c4952f39f5" boundaryCondition="false" compartment="metaComp">**

**<notes>**

**<body xmlns="http://www.w3.org/1999/xhtml">**

**<p>FORMULA: C10H9NO2</p>**

**<p>CHARGE: 0</p>**

**<p>PUBCHEM.COMPOUND: 74688</p>**

**<p>INCHIKEY: OBFAPCIUSYHFIE-UHFFFAOYSA-N</p>**

**<p>KEGG.COMPOUND: C05634</p>**

**<p>HMDB: HMDB04073</p>**

**<p>CHEBI: CHEBI:50157</p>**

**<p>INCHI: InChI=1S/C10H9NO2/c12-4-3-7-6-11-10-2-1-8(13)5-9(7)10/h1-2,4-6,11,13H,3H2</p>**

**</body>**

**</notes>**

**<annotation>**

**<rdf:RDF xmlns:rdf="http://www.w3.org/1999/02/22-rdf-syntax-ns#" xmlns:bqmodel="http://biomodels.net/model-qualifiers/" xmlns:bqbiol="http://biomodels.net/biology-qualifiers/">**

**<rdf:Description rdf:about="_3902debb-9398-4577-a3d7-61c4952f39f5">**

**<bqbiol:is>**

**<rdf:Bag>**

**<rdf:li rdf:resource="http://identifiers.org/PUBCHEM.COMPOUND/74688"/></rdf:Bag></bqbiol:is><bqbiol:is>**

**<rdf:Bag>**

**<rdf:li rdf:resource="http://identifiers.org/INCHIKEY/OBFAPCIUSYHFIE-UHFFFAOYSA-N"/></rdf:Bag></bqbiol:is><bqbiol:is>**

**<rdf:Bag>**

**<rdf:li rdf:resource="http://identifiers.org/KEGG.COMPOUND/C05634"/></rdf:Bag></bqbiol:is><bqbiol:is>**

**<rdf:Bag>**

**<rdf:li rdf:resource="http://identifiers.org/HMDB/HMDB04073"/></rdf:Bag></bqbiol:is><bqbiol:is>**

**<rdf:Bag>**

**<rdf:li rdf:resource="http://identifiers.org/CHEBI/CHEBI:50157"/></rdf:Bag></bqbiol:is><bqbiol:is>**

**<rdf:Bag>**

**<rdf:li rdf:resource="http://identifiers.org/INCHI/InChI=1S/C10H9NO2/c12-4-3-7-6-11-10-2-1-8(13)5-9(7)10/h1-2,4-6,11,13H,3H2"/></rdf:Bag></bqbiol:is></rdf:Description></rdf:RDF>**

**</annotation>**

**</species>**

**<species id="M_3ivcrn" constant="false" hasOnlySubstanceUnits="false" name="3-hydroxy-isovaleryl carnitine" metaid="7132f4a5-2865-4a44-82fb-bcc48cd0215f" boundaryCondition="false" compartment="metaComp">**

**<notes>**

**<body xmlns="http://www.w3.org/1999/xhtml">**

**<p>FORMULA: C12H23NO5</p>**

**<p>CHARGE: 0</p>**

**<p>CHEBI: CHEBI:73027 || CHEBI:82958</p>**

**<p>INCHI: InChI=1S/C12H23NO5/c1-12(2,17)7-11(16)18-9(6-10(14)15)8-13(3,4)5/h9,17H,6-8H2,1-5H3</p>**

**</body>**

**</notes>**

**<annotation>**

**<rdf:RDF xmlns:rdf="http://www.w3.org/1999/02/22-rdf-syntax-ns#" xmlns:bqmodel="http://biomodels.net/model-qualifiers/" xmlns:bqbiol="http://biomodels.net/biology-qualifiers/">**

**<rdf:Description rdf:about="_7132f4a5-2865-4a44-82fb-bcc48cd0215f"/></rdf:RDF>**

**</annotation>**

**</species>**

**<species id="M_fe2" constant="false" hasOnlySubstanceUnits="false" name="Fe2+" metaid="5e264609-9d78-45cc-bba0-3849bb6915ac" boundaryCondition="false" compartment="metaComp">**

**<notes>**

**<body xmlns="http://www.w3.org/1999/xhtml">**

**<p>FORMULA: FE</p>**

**<p>CHARGE: 0</p>**

**<p>PUBCHEM.COMPOUND: 27284</p>**

**<p>KEGG.COMPOUND: C14818 || C00023</p>**

**<p>HMDB: HMDB00692</p>**

**<p>CHEBI: CHEBI:29033</p>**

**<p>INCHI: InChI=1S/Fe/q+2</p>**

**</body>**

**</notes>**

**<annotation>**

**<rdf:RDF xmlns:rdf="http://www.w3.org/1999/02/22-rdf-syntax-ns#" xmlns:bqmodel="http://biomodels.net/model-qualifiers/" xmlns:bqbiol="http://biomodels.net/biology-qualifiers/">**

**<rdf:Description rdf:about="_5e264609-9d78-45cc-bba0-3849bb6915ac">**

**<bqbiol:is>**

**<rdf:Bag>**

**<rdf:li rdf:resource="http://identifiers.org/PUBCHEM.COMPOUND/27284"/></rdf:Bag></bqbiol:is><bqbiol:is>**

**<rdf:Bag>**

**<rdf:li rdf:resource="http://identifiers.org/KEGG.COMPOUND/C14818"/></rdf:Bag></bqbiol:is><bqbiol:is>**

**<rdf:Bag>**

**<rdf:li rdf:resource="http://identifiers.org/KEGG.COMPOUND/C00023"/></rdf:Bag></bqbiol:is><bqbiol:is>**

**<rdf:Bag>**

**<rdf:li rdf:resource="http://identifiers.org/HMDB/HMDB00692"/></rdf:Bag></bqbiol:is><bqbiol:is>**

**<rdf:Bag>**

**<rdf:li rdf:resource="http://identifiers.org/CHEBI/CHEBI:29033"/></rdf:Bag></bqbiol:is><bqbiol:is>**

**<rdf:Bag>**

**<rdf:li rdf:resource="http://identifiers.org/INCHI/InChI=1S/Fe/q+2"/></rdf:Bag></bqbiol:is></rdf:Description></rdf:RDF>**

**</annotation>**

**</species>**

**<species id="M_hcys_L" constant="false" hasOnlySubstanceUnits="false" name="L-homocysteine" metaid="ffcf0b88-bdb3-46fb-b196-a50d4b068fde" boundaryCondition="false" compartment="metaComp">**

**<notes>**

**<body xmlns="http://www.w3.org/1999/xhtml">**

**<p>FORMULA: C4H9NO2S</p>**

**<p>CHARGE: 0</p>**

**<p>PUBCHEM.COMPOUND: 778 || 49791978 || 91552 || 6971015</p>**

**<p>INCHIKEY: FFFHZYDWPBMWHY-VKHMYHEASA-N</p>**

**<p>KEGG.COMPOUND: C00155 || C05330</p>**

**<p>HMDB: HMDB00742</p>**

**<p>CHEBI: CHEBI:17588 || CHEBI:58065 || CHEBI:58199 || CHEBI:17230</p>**

**<p>INCHI: InChI=1S/C4H9NO2S/c5-3(1-2-8)4(6)7/h3,8H,1-2,5H2,(H,6,7) || InChI=1S/C4H9NO2S/c5-3(1-2-8)4(6)7/h3,8H,1-2,5H2,(H,6,7)/t3-/m0/s1</p>**

**</body>**

**</notes>**

**<annotation>**

**<rdf:RDF xmlns:rdf="http://www.w3.org/1999/02/22-rdf-syntax-ns#" xmlns:bqmodel="http://biomodels.net/model-qualifiers/" xmlns:bqbiol="http://biomodels.net/biology-qualifiers/">**

**<rdf:Description rdf:about="ffcf0b88-bdb3-46fb-b196-a50d4b068fde">**

**<bqbiol:is>**

**<rdf:Bag>**

**<rdf:li rdf:resource="http://identifiers.org/PUBCHEM.COMPOUND/778"/></rdf:Bag></bqbiol:is><bqbiol:is>**

**<rdf:Bag>**

**<rdf:li rdf:resource="http://identifiers.org/PUBCHEM.COMPOUND/49791978"/></rdf:Bag></bqbiol:is><bqbiol:is>**

**<rdf:Bag>**

**<rdf:li rdf:resource="http://identifiers.org/PUBCHEM.COMPOUND/91552"/></rdf:Bag></bqbiol:is><bqbiol:is>**

**<rdf:Bag>**

**<rdf:li rdf:resource="http://identifiers.org/PUBCHEM.COMPOUND/6971015"/></rdf:Bag></bqbiol:is><bqbiol:is>**

**<rdf:Bag>**

**<rdf:li rdf:resource="http://identifiers.org/INCHIKEY/FFFHZYDWPBMWHY-VKHMYHEASA-N"/></rdf:Bag></bqbiol:is><bqbiol:is>**

**<rdf:Bag>**

**<rdf:li rdf:resource="http://identifiers.org/KEGG.COMPOUND/C00155"/></rdf:Bag></bqbiol:is><bqbiol:is>**

**<rdf:Bag>**

**<rdf:li rdf:resource="http://identifiers.org/KEGG.COMPOUND/C05330"/></rdf:Bag></bqbiol:is><bqbiol:is>**

**<rdf:Bag>**

**<rdf:li rdf:resource="http://identifiers.org/HMDB/HMDB00742"/></rdf:Bag></bqbiol:is><bqbiol:is>**

**<rdf:Bag>**

**<rdf:li rdf:resource="http://identifiers.org/CHEBI/CHEBI:17588"/></rdf:Bag></bqbiol:is><bqbiol:is>**

**<rdf:Bag>**

**<rdf:li rdf:resource="http://identifiers.org/CHEBI/CHEBI:58065"/></rdf:Bag></bqbiol:is><bqbiol:is>**

**<rdf:Bag>**

**<rdf:li rdf:resource="http://identifiers.org/CHEBI/CHEBI:58199"/></rdf:Bag></bqbiol:is><bqbiol:is>**

**<rdf:Bag>**

**<rdf:li rdf:resource="http://identifiers.org/CHEBI/CHEBI:17230"/></rdf:Bag></bqbiol:is><bqbiol:is>**

**<rdf:Bag>**

**<rdf:li rdf:resource="http://identifiers.org/INCHI/InChI=1S/C4H9NO2S/c5-3(1-2-8)4(6)7/h3,8H,1-2,5H2,(H,6,7)"/></rdf:Bag></bqbiol:is><bqbiol:is>**

**<rdf:Bag>**

**<rdf:li rdf:resource="http://identifiers.org/INCHI/InChI=1S/C4H9NO2S/c5-3(1-2-8)4(6)7/h3,8H,1-2,5H2,(H,6,7)/t3-/m0/s1"/></rdf:Bag></bqbiol:is></rdf:Description></rdf:RDF>**

**</annotation>**

**</species>**

**<species id="M_48dhoxquin" constant="false" hasOnlySubstanceUnits="false" name="quinoline-4,8-diol" metaid="f6416d5e-b461-4f16-9ccd-8bca60fa6c07" boundaryCondition="false" compartment="metaComp">**

**<notes>**

**<body xmlns="http://www.w3.org/1999/xhtml">**

**<p>FORMULA: C9H7NO2</p>**

**<p>CHARGE: 0</p>**

**<p>PUBCHEM.COMPOUND: 440737</p>**

**<p>INCHIKEY: PYELIMVFIITPER-UHFFFAOYSA-N</p>**

**<p>KEGG.COMPOUND: C05637</p>**

**<p>CHEBI: CHEBI:28883</p>**

**<p>INCHI: InChI=1S/C9H7NO2/c11-7-4-5-10-9-6(7)2-1-3-8(9)12/h1-5,12H,(H,10,11)</p>**

**</body>**

**</notes>**

**<annotation>**

**<rdf:RDF xmlns:rdf="http://www.w3.org/1999/02/22-rdf-syntax-ns#" xmlns:bqmodel="http://biomodels.net/model-qualifiers/" xmlns:bqbiol="http://biomodels.net/biology-qualifiers/">**

**<rdf:Description rdf:about="f6416d5e-b461-4f16-9ccd-8bca60fa6c07">**

**<bqbiol:is>**

**<rdf:Bag>**

**<rdf:li rdf:resource="http://identifiers.org/PUBCHEM.COMPOUND/440737"/></rdf:Bag></bqbiol:is><bqbiol:is>**

**<rdf:Bag>**

**<rdf:li rdf:resource="http://identifiers.org/INCHIKEY/PYELIMVFIITPER-UHFFFAOYSA-N"/></rdf:Bag></bqbiol:is><bqbiol:is>**

**<rdf:Bag>**

**<rdf:li rdf:resource="http://identifiers.org/KEGG.COMPOUND/C05637"/></rdf:Bag></bqbiol:is><bqbiol:is>**

**<rdf:Bag>**

**<rdf:li rdf:resource="http://identifiers.org/CHEBI/CHEBI:28883"/></rdf:Bag></bqbiol:is><bqbiol:is>**

**<rdf:Bag>**

**<rdf:li rdf:resource="http://identifiers.org/INCHI/InChI=1S/C9H7NO2/c11-7-4-5-10-9-6(7)2-1-3-8(9)12/h1-5,12H,(H,10,11)"/></rdf:Bag></bqbiol:is></rdf:Description></rdf:RDF>**

**</annotation>**

**</species>**

**<species id="M_fe3" constant="false" hasOnlySubstanceUnits="false" name="Fe3+" metaid="ac9b1a60-6d79-4650-8cb2-50429e34724c" boundaryCondition="false" compartment="metaComp">**

**<notes>**

**<body xmlns="http://www.w3.org/1999/xhtml">**

**<p>FORMULA: FE</p>**

**<p>CHARGE: 0</p>**

**<p>PUBCHEM.COMPOUND: 29936</p>**

**<p>KEGG.COMPOUND: C14819</p>**

**<p>HMDB: HMDB12943</p>**

**<p>CHEBI: CHEBI:29034</p>**

**<p>INCHI: InChI=1S/Fe/q+3</p>**

**</body>**

**</notes>**

**<annotation>**

**<rdf:RDF xmlns:rdf="http://www.w3.org/1999/02/22-rdf-syntax-ns#" xmlns:bqmodel="http://biomodels.net/model-qualifiers/" xmlns:bqbiol="http://biomodels.net/biology-qualifiers/">**

**<rdf:Description rdf:about="ac9b1a60-6d79-4650-8cb2-50429e34724c">**

**<bqbiol:is>**

**<rdf:Bag>**

**<rdf:li rdf:resource="http://identifiers.org/PUBCHEM.COMPOUND/29936"/></rdf:Bag></bqbiol:is><bqbiol:is>**

**<rdf:Bag>**

**<rdf:li rdf:resource="http://identifiers.org/KEGG.COMPOUND/C14819"/></rdf:Bag></bqbiol:is><bqbiol:is>**

**<rdf:Bag>**

**<rdf:li rdf:resource="http://identifiers.org/HMDB/HMDB12943"/></rdf:Bag></bqbiol:is><bqbiol:is>**

**<rdf:Bag>**

**<rdf:li rdf:resource="http://identifiers.org/CHEBI/CHEBI:29034"/></rdf:Bag></bqbiol:is><bqbiol:is>**

**<rdf:Bag>**

**<rdf:li rdf:resource="http://identifiers.org/INCHI/InChI=1S/Fe/q+3"/></rdf:Bag></bqbiol:is></rdf:Description></rdf:RDF>**

**</annotation>**

**</species>**

**<species id="M_g3m8masn" constant="false" hasOnlySubstanceUnits="false" name="(alpha-D-Glucosyl)3-(alpha-D-mannosyl)8-beta-D-mannosyl-diacetylchitobiosyl-L-asparagine (protein)" metaid="df035dd9-ef6b-4f54-b2f8-e2d5ff62381e" boundaryCondition="false" compartment="metaComp">**

**<notes>**

**<body xmlns="http://www.w3.org/1999/xhtml">**

**<p>FORMULA: C88H147N2O70X</p>**

**<p>CHARGE: 0</p>**

**<p>KEGG.COMPOUND: C05873</p>**

**</body>**

**</notes>**

**<annotation>**

**<rdf:RDF xmlns:rdf="http://www.w3.org/1999/02/22-rdf-syntax-ns#" xmlns:bqmodel="http://biomodels.net/model-qualifiers/" xmlns:bqbiol="http://biomodels.net/biology-qualifiers/">**

**<rdf:Description rdf:about="df035dd9-ef6b-4f54-b2f8-e2d5ff62381e">**

**<bqbiol:is>**

**<rdf:Bag>**

**<rdf:li rdf:resource="http://identifiers.org/KEGG.COMPOUND/C05873"/></rdf:Bag></bqbiol:is></rdf:Description></rdf:RDF>**

**</annotation>**

**</species>**

**<species id="M_C14849" constant="false" hasOnlySubstanceUnits="false" name="Benzo[a]pyrene-9,10-oxide" metaid="13b07c39-dc11-4fd6-9c55-2a69b4d8a210" boundaryCondition="false" compartment="metaComp">**

**<notes>**

**<body xmlns="http://www.w3.org/1999/xhtml">**

**<p>FORMULA: C20H12O</p>**

**<p>CHARGE: 0</p>**

**<p>PUBCHEM.COMPOUND: 37456</p>**

**<p>INCHIKEY: GOEJUYABKOTLJA-UHFFFAOYSA-N</p>**

**<p>KEGG.COMPOUND: C14849</p>**

**<p>CHEBI: CHEBI:34564</p>**

**<p>INCHI: InChI=1/C20H12O/c1-2-11-4-5-13-10-14-7-9-16-20(21-16)19(14)15-8-6-12(3-1)17(11)18(13)15/h1-10,16,20H || InChI=1S/C20H12O/c1-2-11-4-5-13-10-14-7-9-16-20(21-16)19(14)15-8-6-12(3-1)17(11)18(13)15/h1-10,16,20H</p>**

**</body>**

**</notes>**

**<annotation>**

**<rdf:RDF xmlns:rdf="http://www.w3.org/1999/02/22-rdf-syntax-ns#" xmlns:bqmodel="http://biomodels.net/model-qualifiers/" xmlns:bqbiol="http://biomodels.net/biology-qualifiers/">**

**<rdf:Description rdf:about="_13b07c39-dc11-4fd6-9c55-2a69b4d8a210">**

**<bqbiol:is>**

**<rdf:Bag>**

**<rdf:li rdf:resource="http://identifiers.org/PUBCHEM.COMPOUND/37456"/></rdf:Bag></bqbiol:is><bqbiol:is>**

**<rdf:Bag>**

**<rdf:li rdf:resource="http://identifiers.org/INCHIKEY/GOEJUYABKOTLJA-UHFFFAOYSA-N"/></rdf:Bag></bqbiol:is><bqbiol:is>**

**<rdf:Bag>**

**<rdf:li rdf:resource="http://identifiers.org/KEGG.COMPOUND/C14849"/></rdf:Bag></bqbiol:is><bqbiol:is>**

**<rdf:Bag>**

**<rdf:li rdf:resource="http://identifiers.org/CHEBI/CHEBI:34564"/></rdf:Bag></bqbiol:is><bqbiol:is>**

**<rdf:Bag>**

**<rdf:li rdf:resource="http://identifiers.org/INCHI/InChI=1/C20H12O/c1-2-11-4-5-13-10-14-7-9-16-20(21-16)19(14)15-8-6-12(3-1)17(11)18(13)15/h1-10,16,20H"/></rdf:Bag></bqbiol:is><bqbiol:is>**

**<rdf:Bag>**

**<rdf:li rdf:resource="http://identifiers.org/INCHI/InChI=1S/C20H12O/c1-2-11-4-5-13-10-14-7-9-16-20(21-16)19(14)15-8-6-12(3-1)17(11)18(13)15/h1-10,16,20H"/></rdf:Bag></bqbiol:is></rdf:Description></rdf:RDF>**

**</annotation>**

**</species>**

**<species id="M_gltcho" constant="false" hasOnlySubstanceUnits="false" name="beta glucan-taurocholic acid complex" metaid="fa8c039c-8736-4b8b-bd81-589f6fa43e56" boundaryCondition="false" compartment="metaComp">**

**<notes>**

**<body xmlns="http://www.w3.org/1999/xhtml">**

**<p>FORMULA: C1200026H2200045NO1100007S</p>**

**<p>CHARGE: 0</p>**

**</body>**

**</notes>**

**<annotation>**

**<rdf:RDF xmlns:rdf="http://www.w3.org/1999/02/22-rdf-syntax-ns#" xmlns:bqmodel="http://biomodels.net/model-qualifiers/" xmlns:bqbiol="http://biomodels.net/biology-qualifiers/">**

**<rdf:Description rdf:about="fa8c039c-8736-4b8b-bd81-589f6fa43e56"/></rdf:RDF>**

**</annotation>**

**</species>**

**<species id="M_gt1alpha_hs" constant="false" hasOnlySubstanceUnits="false" name="GT1aalpha" metaid="b4a69850-0b12-4320-af82-b6d57f1ad18f" boundaryCondition="false" compartment="metaComp">**

**<notes>**

**<body xmlns="http://www.w3.org/1999/xhtml">**

**<p>FORMULA: C77H127N5O46FULLRCO</p>**

**<p>CHARGE: 0</p>**

**</body>**

**</notes>**

**<annotation>**

**<rdf:RDF xmlns:rdf="http://www.w3.org/1999/02/22-rdf-syntax-ns#" xmlns:bqmodel="http://biomodels.net/model-qualifiers/" xmlns:bqbiol="http://biomodels.net/biology-qualifiers/">**

**<rdf:Description rdf:about="b4a69850-0b12-4320-af82-b6d57f1ad18f"/></rdf:RDF>**

**</annotation>**

**</species>**

**<species id="M_HC00695" constant="false" hasOnlySubstanceUnits="false" name="S-Succinyldihydrolipoamide" metaid="518a82d6-e318-4f36-aca0-fe1e85b26cc1" boundaryCondition="false" compartment="metaComp">**

**<notes>**

**<body xmlns="http://www.w3.org/1999/xhtml">**

**<p>FORMULA: C12H20NO4S2</p>**

**<p>CHARGE: 0</p>**

**<p>PUBCHEM.COMPOUND: 439425 || 11953795</p>**

**<p>INCHIKEY: KWKBJWYJJBQOAE-UHFFFAOYSA-N</p>**

**<p>KEGG.COMPOUND: C01169</p>**

**<p>HMDB: HMDB01177</p>**

**<p>CHEBI: CHEBI:17432</p>**

**<p>INCHI: InChI=1S/C12H21NO4S2/c13-10(14)4-2-1-3-9(7-8-18)19-12(17)6-5-11(15)16/h9,18H,1-8H2,(H2,13,14)(H,15,16)/t9-/m1/s1 || InChI=1S/C12H21NO4S2/c13-10(14)4-2-1-3-9(18)7-8-19-12(17)6-5-11(15)16/h9,18H,1-8H2,(H2,13,14)(H,15,16)</p>**

**</body>**

**</notes>**

**<annotation>**

**<rdf:RDF xmlns:rdf="http://www.w3.org/1999/02/22-rdf-syntax-ns#" xmlns:bqmodel="http://biomodels.net/model-qualifiers/" xmlns:bqbiol="http://biomodels.net/biology-qualifiers/">**

**<rdf:Description rdf:about="_518a82d6-e318-4f36-aca0-fe1e85b26cc1">**

**<bqbiol:is>**

**<rdf:Bag>**

**<rdf:li rdf:resource="http://identifiers.org/PUBCHEM.COMPOUND/439425"/></rdf:Bag></bqbiol:is><bqbiol:is>**

**<rdf:Bag>**

**<rdf:li rdf:resource="http://identifiers.org/PUBCHEM.COMPOUND/11953795"/></rdf:Bag></bqbiol:is><bqbiol:is>**

**<rdf:Bag>**

**<rdf:li rdf:resource="http://identifiers.org/INCHIKEY/KWKBJWYJJBQOAE-UHFFFAOYSA-N"/></rdf:Bag></bqbiol:is><bqbiol:is>**

**<rdf:Bag>**

**<rdf:li rdf:resource="http://identifiers.org/KEGG.COMPOUND/C01169"/></rdf:Bag></bqbiol:is><bqbiol:is>**

**<rdf:Bag>**

**<rdf:li rdf:resource="http://identifiers.org/HMDB/HMDB01177"/></rdf:Bag></bqbiol:is><bqbiol:is>**

**<rdf:Bag>**

**<rdf:li rdf:resource="http://identifiers.org/CHEBI/CHEBI:17432"/></rdf:Bag></bqbiol:is><bqbiol:is>**

**<rdf:Bag>**

**<rdf:li rdf:resource="http://identifiers.org/INCHI/InChI=1S/C12H21NO4S2/c13-10(14)4-2-1-3-9(7-8-18)19-12(17)6-5-11(15)16/h9,18H,1-8H2,(H2,13,14)(H,15,16)/t9-/m1/s1"/></rdf:Bag></bqbiol:is><bqbiol:is>**

**<rdf:Bag>**

**<rdf:li rdf:resource="http://identifiers.org/INCHI/InChI=1S/C12H21NO4S2/c13-10(14)4-2-1-3-9(18)7-8-19-12(17)6-5-11(15)16/h9,18H,1-8H2,(H2,13,14)(H,15,16)"/></rdf:Bag></bqbiol:is></rdf:Description></rdf:RDF>**

**</annotation>**

**</species>**

**<species id="M_uacgam" constant="false" hasOnlySubstanceUnits="false" name="UDP-N-acetyl-alpha-D-glucosamine(2-)" metaid="7fcb0f57-c3f5-4633-8824-1e04069138bc" boundaryCondition="false" compartment="metaComp">**

**<notes>**

**<body xmlns="http://www.w3.org/1999/xhtml">**

**<p>FORMULA: C17H25N3O17P2</p>**

**<p>CHARGE: 0</p>**

**<p>PUBCHEM.COMPOUND: 445675</p>**

**<p>KEGG.COMPOUND: C00043</p>**

**<p>HMDB: HMDB00290</p>**

**<p>CHEBI: CHEBI:57705 || CHEBI:16264</p>**

**<p>INCHI: InChI=1S/C17H27N3O17P2/c1-6(22)18-10-13(26)11(24)7(4-21)35-16(10)36-39(31,32)37-38(29,30)33-5-8-12(25)14(27)15(34-8)20-3-2-9(23)19-17(20)28/h2-3,7-8,10-16,21,24-27H,4-5H2,1H3,(H,18,22)(H,29,30)(H,31,32)(H,19,23,28)/t7-,8-,10-,11-,12-,13-,14-,15-,16-/m1/s1 || InChI=1S/C17H27N3O17P2/c1-6(22)18-10-13(26)11(24)7(4-21)35-16(10)36-39(31,32)37-38(29,30)33-5-8-12(25)14(27)15(34-8)20-3-2-9(23)19-17(20)28/h2-3,7-8,10-16,21,24-27H,4-5H2,1H3,(H,18,22)(H,29,30)(H,31,32)(H,19,23,28)/p-2/t7-,8-,10-,11-,12-,13-,14-,15-,16-/m1/s1</p>**

**</body>**

**</notes>**

**<annotation>**

**<rdf:RDF xmlns:rdf="http://www.w3.org/1999/02/22-rdf-syntax-ns#" xmlns:bqmodel="http://biomodels.net/model-qualifiers/" xmlns:bqbiol="http://biomodels.net/biology-qualifiers/">**

**<rdf:Description rdf:about="_7fcb0f57-c3f5-4633-8824-1e04069138bc">**

**<bqbiol:is>**

**<rdf:Bag>**

**<rdf:li rdf:resource="http://identifiers.org/PUBCHEM.COMPOUND/445675"/></rdf:Bag></bqbiol:is><bqbiol:is>**

**<rdf:Bag>**

**<rdf:li rdf:resource="http://identifiers.org/KEGG.COMPOUND/C00043"/></rdf:Bag></bqbiol:is><bqbiol:is>**

**<rdf:Bag>**

**<rdf:li rdf:resource="http://identifiers.org/HMDB/HMDB00290"/></rdf:Bag></bqbiol:is><bqbiol:is>**

**<rdf:Bag>**

**<rdf:li rdf:resource="http://identifiers.org/CHEBI/CHEBI:57705"/></rdf:Bag></bqbiol:is><bqbiol:is>**

**<rdf:Bag>**

**<rdf:li rdf:resource="http://identifiers.org/CHEBI/CHEBI:16264"/></rdf:Bag></bqbiol:is><bqbiol:is>**

**<rdf:Bag>**

**<rdf:li rdf:resource="http://identifiers.org/INCHI/InChI=1S/C17H27N3O17P2/c1-6(22)18-10-13(26)11(24)7(4-21)35-16(10)36-39(31,32)37-38(29,30)33-5-8-12(25)14(27)15(34-8)20-3-2-9(23)19-17(20)28/h2-3,7-8,10-16,21,24-27H,4-5H2,1H3,(H,18,22)(H,29,30)(H,31,32)(H,19,23,28)/t7-,8-,10-,11-,12-,13-,14-,15-,16-/m1/s1"/></rdf:Bag></bqbiol:is><bqbiol:is>**

**<rdf:Bag>**

**<rdf:li rdf:resource="http://identifiers.org/INCHI/InChI=1S/C17H27N3O17P2/c1-6(22)18-10-13(26)11(24)7(4-21)35-16(10)36-39(31,32)37-38(29,30)33-5-8-12(25)14(27)15(34-8)20-3-2-9(23)19-17(20)28/h2-3,7-8,10-16,21,24-27H,4-5H2,1H3,(H,18,22)(H,29,30)(H,31,32)(H,19,23,28)/p-2/t7-,8-,10-,11-,12-,13-,14-,15-,16-/m1/s1"/></rdf:Bag></bqbiol:is></rdf:Description></rdf:RDF>**

**</annotation>**

**</species>**

**<species id="M_HC01335" constant="false" hasOnlySubstanceUnits="false" name="10fthf5glu[c]" metaid="c9393d3f-4434-4ce2-8256-cfb091f0d4cc" boundaryCondition="false" compartment="metaComp">**

**<notes>**

**<body xmlns="http://www.w3.org/1999/xhtml">**

**<p>FORMULA: C40H45N11O19</p>**

**<p>CHARGE: 0</p>**

**</body>**

**</notes>**

**<annotation>**

**<rdf:RDF xmlns:rdf="http://www.w3.org/1999/02/22-rdf-syntax-ns#" xmlns:bqmodel="http://biomodels.net/model-qualifiers/" xmlns:bqbiol="http://biomodels.net/biology-qualifiers/">**

**<rdf:Description rdf:about="c9393d3f-4434-4ce2-8256-cfb091f0d4cc"/></rdf:RDF>**

**</annotation>**

**</species>**

**<species id="M_C09209" constant="false" hasOnlySubstanceUnits="false" name="Harman" metaid="c039b4c1-0208-486b-a829-1dec779b7144" boundaryCondition="false" compartment="metaComp">**

**<notes>**

**<body xmlns="http://www.w3.org/1999/xhtml">**

**<p>FORMULA: C12H10N2</p>**

**<p>CHARGE: 0</p>**

**<p>PUBCHEM.COMPOUND: 5281404</p>**

**<p>INCHIKEY: PSFDQSOCUJVVGF-UHFFFAOYSA-N</p>**

**<p>KEGG.COMPOUND: C09209</p>**

**<p>CHEBI: CHEBI:5623</p>**

**<p>INCHI: InChI=1S/C12H10N2/c1-8-12-10(6-7-13-8)9-4-2-3-5-11(9)14-12/h2-7,14H,1H3 || InChI=1/C12H10N2/c1-8-12-10(6-7-13-8)9-4-2-3-5-11(9)14-12/h2-7,14H,1H3</p>**

**</body>**

**</notes>**

**<annotation>**

**<rdf:RDF xmlns:rdf="http://www.w3.org/1999/02/22-rdf-syntax-ns#" xmlns:bqmodel="http://biomodels.net/model-qualifiers/" xmlns:bqbiol="http://biomodels.net/biology-qualifiers/">**

**<rdf:Description rdf:about="c039b4c1-0208-486b-a829-1dec779b7144">**

**<bqbiol:is>**

**<rdf:Bag>**

**<rdf:li rdf:resource="http://identifiers.org/PUBCHEM.COMPOUND/5281404"/></rdf:Bag></bqbiol:is><bqbiol:is>**

**<rdf:Bag>**

**<rdf:li rdf:resource="http://identifiers.org/INCHIKEY/PSFDQSOCUJVVGF-UHFFFAOYSA-N"/></rdf:Bag></bqbiol:is><bqbiol:is>**

**<rdf:Bag>**

**<rdf:li rdf:resource="http://identifiers.org/KEGG.COMPOUND/C09209"/></rdf:Bag></bqbiol:is><bqbiol:is>**

**<rdf:Bag>**

**<rdf:li rdf:resource="http://identifiers.org/CHEBI/CHEBI:5623"/></rdf:Bag></bqbiol:is><bqbiol:is>**

**<rdf:Bag>**

**<rdf:li rdf:resource="http://identifiers.org/INCHI/InChI=1S/C12H10N2/c1-8-12-10(6-7-13-8)9-4-2-3-5-11(9)14-12/h2-7,14H,1H3"/></rdf:Bag></bqbiol:is><bqbiol:is>**

**<rdf:Bag>**

**<rdf:li rdf:resource="http://identifiers.org/INCHI/InChI=1/C12H10N2/c1-8-12-10(6-7-13-8)9-4-2-3-5-11(9)14-12/h2-7,14H,1H3"/></rdf:Bag></bqbiol:is></rdf:Description></rdf:RDF>**

**</annotation>**

**</species>**

**<species id="M_ttdcea" constant="false" hasOnlySubstanceUnits="false" name="tetradecenoate (n-C14:1)" metaid="d6ceec5a-8de1-467d-b2d4-20b1f24783f6" boundaryCondition="false" compartment="metaComp">**

**<notes>**

**<body xmlns="http://www.w3.org/1999/xhtml">**

**<p>FORMULA: C14H25O2</p>**

**<p>CHARGE: 0</p>**

**<p>PUBCHEM.COMPOUND: 5281119 || 68344</p>**

**<p>KEGG.COMPOUND: C08322</p>**

**<p>HMDB: HMDB02000</p>**

**<p>CHEBI: CHEBI:27781</p>**

**<p>INCHI: InChI=1S/C14H26O2/c1-2-3-4-5-6-7-8-9-10-11-12-13-14(15)16/h5-6H,2-4,7-13H2,1H3,(H,15,16)/b6-5- || InChI=1/C14H26O2/c1-2-3-4-5-6-7-8-9-10-11-12-13-14(15)16/h5-6H,2-4,7-13H2,1H3,(H,15,16)/b6-5-</p>**

**</body>**

**</notes>**

**<annotation>**

**<rdf:RDF xmlns:rdf="http://www.w3.org/1999/02/22-rdf-syntax-ns#" xmlns:bqmodel="http://biomodels.net/model-qualifiers/" xmlns:bqbiol="http://biomodels.net/biology-qualifiers/">**

**<rdf:Description rdf:about="d6ceec5a-8de1-467d-b2d4-20b1f24783f6">**

**<bqbiol:is>**

**<rdf:Bag>**

**<rdf:li rdf:resource="http://identifiers.org/PUBCHEM.COMPOUND/5281119"/></rdf:Bag></bqbiol:is><bqbiol:is>**

**<rdf:Bag>**

**<rdf:li rdf:resource="http://identifiers.org/PUBCHEM.COMPOUND/68344"/></rdf:Bag></bqbiol:is><bqbiol:is>**

**<rdf:Bag>**

**<rdf:li rdf:resource="http://identifiers.org/KEGG.COMPOUND/C08322"/></rdf:Bag></bqbiol:is><bqbiol:is>**

**<rdf:Bag>**

**<rdf:li rdf:resource="http://identifiers.org/HMDB/HMDB02000"/></rdf:Bag></bqbiol:is><bqbiol:is>**

**<rdf:Bag>**

**<rdf:li rdf:resource="http://identifiers.org/CHEBI/CHEBI:27781"/></rdf:Bag></bqbiol:is><bqbiol:is>**

**<rdf:Bag>**

**<rdf:li rdf:resource="http://identifiers.org/INCHI/InChI=1S/C14H26O2/c1-2-3-4-5-6-7-8-9-10-11-12-13-14(15)16/h5-6H,2-4,7-13H2,1H3,(H,15,16)/b6-5-"/></rdf:Bag></bqbiol:is><bqbiol:is>**

**<rdf:Bag>**

**<rdf:li rdf:resource="http://identifiers.org/INCHI/InChI=1/C14H26O2/c1-2-3-4-5-6-7-8-9-10-11-12-13-14(15)16/h5-6H,2-4,7-13H2,1H3,(H,15,16)/b6-5-"/></rdf:Bag></bqbiol:is></rdf:Description></rdf:RDF>**

**</annotation>**

**</species>**

**<species id="M_C14851" constant="false" hasOnlySubstanceUnits="false" name="Benzo[a]pyrene-4,5-oxide" metaid="d6c45ffc-36cc-43e7-8b3c-1b984a792323" boundaryCondition="false" compartment="metaComp">**

**<notes>**

**<body xmlns="http://www.w3.org/1999/xhtml">**

**<p>FORMULA: C20H12O</p>**

**<p>CHARGE: 0</p>**

**<p>PUBCHEM.COMPOUND: 37786</p>**

**<p>INCHIKEY: XGZQLNASOQVQTD-UHFFFAOYSA-N</p>**

**<p>KEGG.COMPOUND: C14851</p>**

**<p>CHEBI: CHEBI:34560</p>**

**<p>INCHI: InChI=1/C20H12O/c1-2-6-13-12(4-1)10-16-18-14(13)9-8-11-5-3-7-15(17(11)18)19-20(16)21-19/h1-10,19-20H || InChI=1S/C20H12O/c1-2-6-13-12(4-1)10-16-18-14(13)9-8-11-5-3-7-15(17(11)18)19-20(16)21-19/h1-10,19-20H</p>**

**</body>**

**</notes>**

**<annotation>**

**<rdf:RDF xmlns:rdf="http://www.w3.org/1999/02/22-rdf-syntax-ns#" xmlns:bqmodel="http://biomodels.net/model-qualifiers/" xmlns:bqbiol="http://biomodels.net/biology-qualifiers/">**

**<rdf:Description rdf:about="d6c45ffc-36cc-43e7-8b3c-1b984a792323">**

**<bqbiol:is>**

**<rdf:Bag>**

**<rdf:li rdf:resource="http://identifiers.org/PUBCHEM.COMPOUND/37786"/></rdf:Bag></bqbiol:is><bqbiol:is>**

**<rdf:Bag>**

**<rdf:li rdf:resource="http://identifiers.org/INCHIKEY/XGZQLNASOQVQTD-UHFFFAOYSA-N"/></rdf:Bag></bqbiol:is><bqbiol:is>**

**<rdf:Bag>**

**<rdf:li rdf:resource="http://identifiers.org/KEGG.COMPOUND/C14851"/></rdf:Bag></bqbiol:is><bqbiol:is>**

**<rdf:Bag>**

**<rdf:li rdf:resource="http://identifiers.org/CHEBI/CHEBI:34560"/></rdf:Bag></bqbiol:is><bqbiol:is>**

**<rdf:Bag>**

**<rdf:li rdf:resource="http://identifiers.org/INCHI/InChI=1/C20H12O/c1-2-6-13-12(4-1)10-16-18-14(13)9-8-11-5-3-7-15(17(11)18)19-20(16)21-19/h1-10,19-20H"/></rdf:Bag></bqbiol:is><bqbiol:is>**

**<rdf:Bag>**

**<rdf:li rdf:resource="http://identifiers.org/INCHI/InChI=1S/C20H12O/c1-2-6-13-12(4-1)10-16-18-14(13)9-8-11-5-3-7-15(17(11)18)19-20(16)21-19/h1-10,19-20H"/></rdf:Bag></bqbiol:is></rdf:Description></rdf:RDF>**

**</annotation>**

**</species>**

**<species id="M_dkmpp" constant="false" hasOnlySubstanceUnits="false" name="2,3-diketo-5-methylthio-1-phosphopentane" metaid="b50a39ff-8d7d-4922-8029-d1da0ffe4308" boundaryCondition="false" compartment="metaComp">**

**<notes>**

**<body xmlns="http://www.w3.org/1999/xhtml">**

**<p>FORMULA: C6H9O6PS</p>**

**<p>CHARGE: 0</p>**

**<p>PUBCHEM.COMPOUND: 561</p>**

**<p>INCHIKEY: HKEAOVFNWRDVAJ-UHFFFAOYSA-L</p>**

**<p>KEGG.COMPOUND: C15650</p>**

**<p>CHEBI: CHEBI:58828 || CHEBI:50604</p>**

**<p>INCHI: InChI=1S/C6H11O6PS/c1-14-3-2-5(7)6(8)4-12-13(9,10)11/h2-4H2,1H3,(H2,9,10,11) || InChI=1S/C6H11O6PS/c1-14-3-2-5(7)6(8)4-12-13(9,10)11/h2-4H2,1H3,(H2,9,10,11)/p-2</p>**

**</body>**

**</notes>**

**<annotation>**

**<rdf:RDF xmlns:rdf="http://www.w3.org/1999/02/22-rdf-syntax-ns#" xmlns:bqmodel="http://biomodels.net/model-qualifiers/" xmlns:bqbiol="http://biomodels.net/biology-qualifiers/">**

**<rdf:Description rdf:about="b50a39ff-8d7d-4922-8029-d1da0ffe4308">**

**<bqbiol:is>**

**<rdf:Bag>**

**<rdf:li rdf:resource="http://identifiers.org/PUBCHEM.COMPOUND/561"/></rdf:Bag></bqbiol:is><bqbiol:is>**

**<rdf:Bag>**

**<rdf:li rdf:resource="http://identifiers.org/INCHIKEY/HKEAOVFNWRDVAJ-UHFFFAOYSA-L"/></rdf:Bag></bqbiol:is><bqbiol:is>**

**<rdf:Bag>**

**<rdf:li rdf:resource="http://identifiers.org/KEGG.COMPOUND/C15650"/></rdf:Bag></bqbiol:is><bqbiol:is>**

**<rdf:Bag>**

**<rdf:li rdf:resource="http://identifiers.org/CHEBI/CHEBI:58828"/></rdf:Bag></bqbiol:is><bqbiol:is>**

**<rdf:Bag>**

**<rdf:li rdf:resource="http://identifiers.org/CHEBI/CHEBI:50604"/></rdf:Bag></bqbiol:is><bqbiol:is>**

**<rdf:Bag>**

**<rdf:li rdf:resource="http://identifiers.org/INCHI/InChI=1S/C6H11O6PS/c1-14-3-2-5(7)6(8)4-12-13(9,10)11/h2-4H2,1H3,(H2,9,10,11)"/></rdf:Bag></bqbiol:is><bqbiol:is>**

**<rdf:Bag>**

**<rdf:li rdf:resource="http://identifiers.org/INCHI/InChI=1S/C6H11O6PS/c1-14-3-2-5(7)6(8)4-12-13(9,10)11/h2-4H2,1H3,(H2,9,10,11)/p-2"/></rdf:Bag></bqbiol:is></rdf:Description></rdf:RDF>**

**</annotation>**

**</species>**

**<species id="M_mi3p_D" constant="false" hasOnlySubstanceUnits="false" name="1D-myo-inositol 3-phosphate(2-)" metaid="255f1d86-a0dc-461b-a369-483082e75df8" boundaryCondition="false" compartment="metaComp">**

**<notes>**

**<body xmlns="http://www.w3.org/1999/xhtml">**

**<p>FORMULA: C6H11O9P</p>**

**<p>CHARGE: 0</p>**

**<p>PUBCHEM.COMPOUND: 440194</p>**

**<p>KEGG.COMPOUND: C04006</p>**

**<p>HMDB: HMDB06814</p>**

**<p>CHEBI: CHEBI:18169 || CHEBI:58401</p>**

**<p>INCHI: InChI=1S/C6H13O9P/c7-1-2(8)4(10)6(5(11)3(1)9)15-16(12,13)14/h1-11H,(H2,12,13,14)/t1-,2-,3+,4-,5-,6-/m0/s1 || InChI=1S/C6H13O9P/c7-1-2(8)4(10)6(5(11)3(1)9)15-16(12,13)14/h1-11H,(H2,12,13,14)/p-2/t1-,2-,3+,4-,5-,6-/m0/s1 || InChI=1S/C6H13O9P/c7-1-2(8)4(10)6(5(11)3(1)9)15-16(12,13)14/h1-11H,(H2,12,13,14)/t1?,2-,3+,4-,5-,6?/m0/s1</p>**

**</body>**

**</notes>**

**<annotation>**

**<rdf:RDF xmlns:rdf="http://www.w3.org/1999/02/22-rdf-syntax-ns#" xmlns:bqmodel="http://biomodels.net/model-qualifiers/" xmlns:bqbiol="http://biomodels.net/biology-qualifiers/">**

**<rdf:Description rdf:about="_255f1d86-a0dc-461b-a369-483082e75df8">**

**<bqbiol:is>**

**<rdf:Bag>**

**<rdf:li rdf:resource="http://identifiers.org/PUBCHEM.COMPOUND/440194"/></rdf:Bag></bqbiol:is><bqbiol:is>**

**<rdf:Bag>**

**<rdf:li rdf:resource="http://identifiers.org/KEGG.COMPOUND/C04006"/></rdf:Bag></bqbiol:is><bqbiol:is>**

**<rdf:Bag>**

**<rdf:li rdf:resource="http://identifiers.org/HMDB/HMDB06814"/></rdf:Bag></bqbiol:is><bqbiol:is>**

**<rdf:Bag>**

**<rdf:li rdf:resource="http://identifiers.org/CHEBI/CHEBI:18169"/></rdf:Bag></bqbiol:is><bqbiol:is>**

**<rdf:Bag>**

**<rdf:li rdf:resource="http://identifiers.org/CHEBI/CHEBI:58401"/></rdf:Bag></bqbiol:is><bqbiol:is>**

**<rdf:Bag>**

**<rdf:li rdf:resource="http://identifiers.org/INCHI/InChI=1S/C6H13O9P/c7-1-2(8)4(10)6(5(11)3(1)9)15-16(12,13)14/h1-11H,(H2,12,13,14)/t1-,2-,3+,4-,5-,6-/m0/s1"/></rdf:Bag></bqbiol:is><bqbiol:is>**

**<rdf:Bag>**

**<rdf:li rdf:resource="http://identifiers.org/INCHI/InChI=1S/C6H13O9P/c7-1-2(8)4(10)6(5(11)3(1)9)15-16(12,13)14/h1-11H,(H2,12,13,14)/p-2/t1-,2-,3+,4-,5-,6-/m0/s1"/></rdf:Bag></bqbiol:is><bqbiol:is>**

**<rdf:Bag>**

**<rdf:li rdf:resource="http://identifiers.org/INCHI/InChI=1S/C6H13O9P/c7-1-2(8)4(10)6(5(11)3(1)9)15-16(12,13)14/h1-11H,(H2,12,13,14)/t1?,2-,3+,4-,5-,6?/m0/s1"/></rdf:Bag></bqbiol:is></rdf:Description></rdf:RDF>**

**</annotation>**

**</species>**

**<species id="M_dmhptcoa" constant="false" hasOnlySubstanceUnits="false" name="2,6 dimethylheptanoyl-CoA" metaid="1a4f8870-d8b7-449a-bab1-12a02793214e" boundaryCondition="false" compartment="metaComp">**

**<notes>**

**<body xmlns="http://www.w3.org/1999/xhtml">**

**<p>FORMULA: C30H52N7O17P3S</p>**

**<p>CHARGE: 0</p>**

**<p>PUBCHEM.COMPOUND: 53477808</p>**

**<p>HMDB: HMDB06258</p>**

**<p>INCHI: InChI=1/C30H52N7O17P3S/c1-17(2)7-6-8-18(3)29(42)58-12-11-32-20(38)9-10-33-27(41)24(40)30(4,5)14-51-57(48,49)54-56(46,47)50-13-19-23(53-55(43,44)45)22(39)28(52-19)37-16-36-21-25(31)34-15-35-26(21)37/h15-19,22-24,28,39-40H,6-14H2,1-5H3,(H,32,38)(H,33,41)(H,46,47)(H,48,49)(H2,31,34,35)(H2,43,44,45)/p-4/t18-,19+,22-,23-,24?,28+/m0/s1 || InChI=1S/C30H52N7O17P3S/c1-17(2)7-6-8-18(3)29(42)58-12-11-32-20(38)9-10-33-27(41)24(40)30(4,5)14-51-57(48,49)54-56(46,47)50-13-19-23(53-55(43,44)45)22(39)28(52-19)37-16-36-21-25(31)34-15-35-26(21)37/h15-19,22-24,28,39-40H,6-14H2,1-5H3,(H,32,38)(H,33,41)(H,46,47)(H,48,49)(H2,31,34,35)(H2,43,44,45)/p-4/t18-,19+,22-,23-,24?,28+/m0/s1</p>**

**</body>**

**</notes>**

**<annotation>**

**<rdf:RDF xmlns:rdf="http://www.w3.org/1999/02/22-rdf-syntax-ns#" xmlns:bqmodel="http://biomodels.net/model-qualifiers/" xmlns:bqbiol="http://biomodels.net/biology-qualifiers/">**

**<rdf:Description rdf:about="_1a4f8870-d8b7-449a-bab1-12a02793214e">**

**<bqbiol:is>**

**<rdf:Bag>**

**<rdf:li rdf:resource="http://identifiers.org/PUBCHEM.COMPOUND/53477808"/></rdf:Bag></bqbiol:is><bqbiol:is>**

**<rdf:Bag>**

**<rdf:li rdf:resource="http://identifiers.org/HMDB/HMDB06258"/></rdf:Bag></bqbiol:is><bqbiol:is>**

**<rdf:Bag>**

**<rdf:li rdf:resource="http://identifiers.org/INCHI/InChI=1/C30H52N7O17P3S/c1-17(2)7-6-8-18(3)29(42)58-12-11-32-20(38)9-10-33-27(41)24(40)30(4,5)14-51-57(48,49)54-56(46,47)50-13-19-23(53-55(43,44)45)22(39)28(52-19)37-16-36-21-25(31)34-15-35-26(21)37/h15-19,22-24,28,39-40H,6-14H2,1-5H3,(H,32,38)(H,33,41)(H,46,47)(H,48,49)(H2,31,34,35)(H2,43,44,45)/p-4/t18-,19+,22-,23-,24?,28+/m0/s1"/></rdf:Bag></bqbiol:is><bqbiol:is>**

**<rdf:Bag>**

**<rdf:li rdf:resource="http://identifiers.org/INCHI/InChI=1S/C30H52N7O17P3S/c1-17(2)7-6-8-18(3)29(42)58-12-11-32-20(38)9-10-33-27(41)24(40)30(4,5)14-51-57(48,49)54-56(46,47)50-13-19-23(53-55(43,44)45)22(39)28(52-19)37-16-36-21-25(31)34-15-35-26(21)37/h15-19,22-24,28,39-40H,6-14H2,1-5H3,(H,32,38)(H,33,41)(H,46,47)(H,48,49)(H2,31,34,35)(H2,43,44,45)/p-4/t18-,19+,22-,23-,24?,28+/m0/s1"/></rdf:Bag></bqbiol:is></rdf:Description></rdf:RDF>**

**</annotation>**

**</species>**

**<species id="M_CE0328" constant="false" hasOnlySubstanceUnits="false" name="docosahexaenoate" metaid="f33277a7-febc-444e-bf42-48bbe4405e2e" boundaryCondition="false" compartment="metaComp">**

**<notes>**

**<body xmlns="http://www.w3.org/1999/xhtml">**

**<p>FORMULA: C22H31O2</p>**

**<p>CHARGE: 0</p>**

**<p>PUBCHEM.COMPOUND: 445580 || 3144</p>**

**<p>KEGG.COMPOUND: C06429</p>**

**<p>HMDB: HMDB02183</p>**

**<p>CHEBI: CHEBI:28125</p>**

**<p>INCHI: InChI=1S/C22H32O2/c1-2-3-4-5-6-7-8-9-10-11-12-13-14-15-16-17-18-19-20-21-22(23)24/h3-4,6-7,9-10,12-13,15-16,18-19H,2,5,8,11,14,17,20-21H2,1H3,(H,23,24) || InChI=1S/C22H32O2/c1-2-3-4-5-6-7-8-9-10-11-12-13-14-15-16-17-18-19-20-21-22(23)24/h3-4,6-7,9-10,12-13,15-16,18-19H,2,5,8,11,14,17,20-21H2,1H3,(H,23,24)/b4-3-,7-6-,10-9-,13-12-,16-15-,19-18-</p>**

**</body>**

**</notes>**

**<annotation>**

**<rdf:RDF xmlns:rdf="http://www.w3.org/1999/02/22-rdf-syntax-ns#" xmlns:bqmodel="http://biomodels.net/model-qualifiers/" xmlns:bqbiol="http://biomodels.net/biology-qualifiers/">**

**<rdf:Description rdf:about="f33277a7-febc-444e-bf42-48bbe4405e2e">**

**<bqbiol:is>**

**<rdf:Bag>**

**<rdf:li rdf:resource="http://identifiers.org/PUBCHEM.COMPOUND/445580"/></rdf:Bag></bqbiol:is><bqbiol:is>**

**<rdf:Bag>**

**<rdf:li rdf:resource="http://identifiers.org/PUBCHEM.COMPOUND/3144"/></rdf:Bag></bqbiol:is><bqbiol:is>**

**<rdf:Bag>**

**<rdf:li rdf:resource="http://identifiers.org/KEGG.COMPOUND/C06429"/></rdf:Bag></bqbiol:is><bqbiol:is>**

**<rdf:Bag>**

**<rdf:li rdf:resource="http://identifiers.org/HMDB/HMDB02183"/></rdf:Bag></bqbiol:is><bqbiol:is>**

**<rdf:Bag>**

**<rdf:li rdf:resource="http://identifiers.org/CHEBI/CHEBI:28125"/></rdf:Bag></bqbiol:is><bqbiol:is>**

**<rdf:Bag>**

**<rdf:li rdf:resource="http://identifiers.org/INCHI/InChI=1S/C22H32O2/c1-2-3-4-5-6-7-8-9-10-11-12-13-14-15-16-17-18-19-20-21-22(23)24/h3-4,6-7,9-10,12-13,15-16,18-19H,2,5,8,11,14,17,20-21H2,1H3,(H,23,24)"/></rdf:Bag></bqbiol:is><bqbiol:is>**

**<rdf:Bag>**

**<rdf:li rdf:resource="http://identifiers.org/INCHI/InChI=1S/C22H32O2/c1-2-3-4-5-6-7-8-9-10-11-12-13-14-15-16-17-18-19-20-21-22(23)24/h3-4,6-7,9-10,12-13,15-16,18-19H,2,5,8,11,14,17,20-21H2,1H3,(H,23,24)/b4-3-,7-6-,10-9-,13-12-,16-15-,19-18-"/></rdf:Bag></bqbiol:is></rdf:Description></rdf:RDF>**

**</annotation>**

**</species>**

**<species id="M_HC01326" constant="false" hasOnlySubstanceUnits="false" name="(R)-3-Hydroxypalmitoyl-ACP" metaid="5bd70fa4-5fb4-4228-8992-2ee225ba38fb" boundaryCondition="false" compartment="metaComp">**

**<notes>**

**<body xmlns="http://www.w3.org/1999/xhtml">**

**<p>CHARGE: 0</p>**

**<p>PUBCHEM.COMPOUND: 7222</p>**

**<p>KEGG.COMPOUND: C04633</p>**

**</body>**

**</notes>**

**<annotation>**

**<rdf:RDF xmlns:rdf="http://www.w3.org/1999/02/22-rdf-syntax-ns#" xmlns:bqmodel="http://biomodels.net/model-qualifiers/" xmlns:bqbiol="http://biomodels.net/biology-qualifiers/">**

**<rdf:Description rdf:about="_5bd70fa4-5fb4-4228-8992-2ee225ba38fb">**

**<bqbiol:is>**

**<rdf:Bag>**

**<rdf:li rdf:resource="http://identifiers.org/PUBCHEM.COMPOUND/7222"/></rdf:Bag></bqbiol:is><bqbiol:is>**

**<rdf:Bag>**

**<rdf:li rdf:resource="http://identifiers.org/KEGG.COMPOUND/C04633"/></rdf:Bag></bqbiol:is></rdf:Description></rdf:RDF>**

**</annotation>**

**</species>**

**<species id="M_odecrn" constant="false" hasOnlySubstanceUnits="false" name="octadecenoyl carnitine" metaid="3ac74ce2-5ecb-4183-a41d-499582af4078" boundaryCondition="false" compartment="metaComp">**

**<notes>**

**<body xmlns="http://www.w3.org/1999/xhtml">**

**<p>FORMULA: C25H47NO4</p>**

**<p>CHARGE: 0</p>**

**</body>**

**</notes>**

**<annotation>**

**<rdf:RDF xmlns:rdf="http://www.w3.org/1999/02/22-rdf-syntax-ns#" xmlns:bqmodel="http://biomodels.net/model-qualifiers/" xmlns:bqbiol="http://biomodels.net/biology-qualifiers/">**

**<rdf:Description rdf:about="_3ac74ce2-5ecb-4183-a41d-499582af4078"/></rdf:RDF>**

**</annotation>**

**</species>**

**<species id="M_cs_b_pre4" constant="false" hasOnlySubstanceUnits="false" name="chondroitin sulfate B (IdoA2S-GalNAc4S), precursor 4" metaid="19f33d2c-b83b-47dc-9078-7a11dea0447f" boundaryCondition="false" compartment="metaComp">**

**<notes>**

**<body xmlns="http://www.w3.org/1999/xhtml">**

**<p>FORMULA: C45H67N2O42S2X</p>**

**<p>CHARGE: 0</p>**

**</body>**

**</notes>**

**<annotation>**

**<rdf:RDF xmlns:rdf="http://www.w3.org/1999/02/22-rdf-syntax-ns#" xmlns:bqmodel="http://biomodels.net/model-qualifiers/" xmlns:bqbiol="http://biomodels.net/biology-qualifiers/">**

**<rdf:Description rdf:about="_19f33d2c-b83b-47dc-9078-7a11dea0447f"/></rdf:RDF>**

**</annotation>**

**</species>**

**<species id="M_HC01321" constant="false" hasOnlySubstanceUnits="false" name="(R)-3-Hydroxybutanoyl-ACP" metaid="7054912c-5fee-4923-9bc8-21bda7d814a5" boundaryCondition="false" compartment="metaComp">**

**<notes>**

**<body xmlns="http://www.w3.org/1999/xhtml">**

**<p>CHARGE: 0</p>**

**<p>PUBCHEM.COMPOUND: 7208</p>**

**<p>KEGG.COMPOUND: C04618</p>**

**</body>**

**</notes>**

**<annotation>**

**<rdf:RDF xmlns:rdf="http://www.w3.org/1999/02/22-rdf-syntax-ns#" xmlns:bqmodel="http://biomodels.net/model-qualifiers/" xmlns:bqbiol="http://biomodels.net/biology-qualifiers/">**

**<rdf:Description rdf:about="_7054912c-5fee-4923-9bc8-21bda7d814a5">**

**<bqbiol:is>**

**<rdf:Bag>**

**<rdf:li rdf:resource="http://identifiers.org/PUBCHEM.COMPOUND/7208"/></rdf:Bag></bqbiol:is><bqbiol:is>**

**<rdf:Bag>**

**<rdf:li rdf:resource="http://identifiers.org/KEGG.COMPOUND/C04618"/></rdf:Bag></bqbiol:is></rdf:Description></rdf:RDF>**

**</annotation>**

**</species>**

**<species id="M_cs_b_pre5" constant="false" hasOnlySubstanceUnits="false" name="chondroitin sulfate B (IdoA2S-GalNAc4s), precursor 5" metaid="f3de8eab-0ad2-4277-b57a-32cb3ef7019d" boundaryCondition="false" compartment="metaComp">**

**<notes>**

**<body xmlns="http://www.w3.org/1999/xhtml">**

**<p>FORMULA: C45H66N2O45S3X</p>**

**<p>CHARGE: 0</p>**

**</body>**

**</notes>**

**<annotation>**

**<rdf:RDF xmlns:rdf="http://www.w3.org/1999/02/22-rdf-syntax-ns#" xmlns:bqmodel="http://biomodels.net/model-qualifiers/" xmlns:bqbiol="http://biomodels.net/biology-qualifiers/">**

**<rdf:Description rdf:about="f3de8eab-0ad2-4277-b57a-32cb3ef7019d"/></rdf:RDF>**

**</annotation>**

**</species>**

**<species id="M_HC01322" constant="false" hasOnlySubstanceUnits="false" name="(R)-3-Hydroxydecanoyl-ACP" metaid="1f755ef8-39f2-4a4f-9652-31431a02eee9" boundaryCondition="false" compartment="metaComp">**

**<notes>**

**<body xmlns="http://www.w3.org/1999/xhtml">**

**<p>CHARGE: 0</p>**

**<p>PUBCHEM.COMPOUND: 7209</p>**

**<p>KEGG.COMPOUND: C04619</p>**

**</body>**

**</notes>**

**<annotation>**

**<rdf:RDF xmlns:rdf="http://www.w3.org/1999/02/22-rdf-syntax-ns#" xmlns:bqmodel="http://biomodels.net/model-qualifiers/" xmlns:bqbiol="http://biomodels.net/biology-qualifiers/">**

**<rdf:Description rdf:about="_1f755ef8-39f2-4a4f-9652-31431a02eee9">**

**<bqbiol:is>**

**<rdf:Bag>**

**<rdf:li rdf:resource="http://identifiers.org/PUBCHEM.COMPOUND/7209"/></rdf:Bag></bqbiol:is><bqbiol:is>**

**<rdf:Bag>**

**<rdf:li rdf:resource="http://identifiers.org/KEGG.COMPOUND/C04619"/></rdf:Bag></bqbiol:is></rdf:Description></rdf:RDF>**

**</annotation>**

**</species>**

**<species id="M_3dodtricoa" constant="false" hasOnlySubstanceUnits="false" name="3,6,9-dodecatrienoylcoa" metaid="6c8db033-95e0-463a-9091-9a55ccb86774" boundaryCondition="false" compartment="metaComp">**

**<notes>**

**<body xmlns="http://www.w3.org/1999/xhtml">**

**<p>FORMULA: C33H48N7O17P3S</p>**

**<p>CHARGE: 0</p>**

**</body>**

**</notes>**

**<annotation>**

**<rdf:RDF xmlns:rdf="http://www.w3.org/1999/02/22-rdf-syntax-ns#" xmlns:bqmodel="http://biomodels.net/model-qualifiers/" xmlns:bqbiol="http://biomodels.net/biology-qualifiers/">**

**<rdf:Description rdf:about="_6c8db033-95e0-463a-9091-9a55ccb86774"/></rdf:RDF>**

**</annotation>**

**</species>**

**<species id="M_thcholst" constant="false" hasOnlySubstanceUnits="false" name="3alpha,7alpha,12alpha-trihydroxy-5beta-cholestan-26-al" metaid="041df103-48f5-4efb-82fb-be85b30cfe67" boundaryCondition="false" compartment="metaComp">**

**<notes>**

**<body xmlns="http://www.w3.org/1999/xhtml">**

**<p>FORMULA: C27H46O4</p>**

**<p>CHARGE: 0</p>**

**<p>PUBCHEM.COMPOUND: 439479 || 24771792</p>**

**<p>INCHIKEY: USFJGINJGUIFSY-XZULNKEGSA-N</p>**

**<p>KEGG.COMPOUND: C01301</p>**

**<p>HMDB: HMDB03533</p>**

**<p>CHEBI: CHEBI:48940 || CHEBI:16466</p>**

**<p>INCHI: InChI=1S/C27H46O4/c1-16(15-28)6-5-7-17(2)20-8-9-21-25-22(14-24(31)27(20,21)4)26(3)11-10-19(29)12-18(26)13-23(25)30/h15-25,29-31H,5-14H2,1-4H3/t16?,17-,18+,19-,20-,21+,22+,23-,24+,25+,26+,27-/m1/s1</p>**

**</body>**

**</notes>**

**<annotation>**

**<rdf:RDF xmlns:rdf="http://www.w3.org/1999/02/22-rdf-syntax-ns#" xmlns:bqmodel="http://biomodels.net/model-qualifiers/" xmlns:bqbiol="http://biomodels.net/biology-qualifiers/">**

**<rdf:Description rdf:about="_041df103-48f5-4efb-82fb-be85b30cfe67">**

**<bqbiol:is>**

**<rdf:Bag>**

**<rdf:li rdf:resource="http://identifiers.org/PUBCHEM.COMPOUND/439479"/></rdf:Bag></bqbiol:is><bqbiol:is>**

**<rdf:Bag>**

**<rdf:li rdf:resource="http://identifiers.org/PUBCHEM.COMPOUND/24771792"/></rdf:Bag></bqbiol:is><bqbiol:is>**

**<rdf:Bag>**

**<rdf:li rdf:resource="http://identifiers.org/INCHIKEY/USFJGINJGUIFSY-XZULNKEGSA-N"/></rdf:Bag></bqbiol:is><bqbiol:is>**

**<rdf:Bag>**

**<rdf:li rdf:resource="http://identifiers.org/KEGG.COMPOUND/C01301"/></rdf:Bag></bqbiol:is><bqbiol:is>**

**<rdf:Bag>**

**<rdf:li rdf:resource="http://identifiers.org/HMDB/HMDB03533"/></rdf:Bag></bqbiol:is><bqbiol:is>**

**<rdf:Bag>**

**<rdf:li rdf:resource="http://identifiers.org/CHEBI/CHEBI:48940"/></rdf:Bag></bqbiol:is><bqbiol:is>**

**<rdf:Bag>**

**<rdf:li rdf:resource="http://identifiers.org/CHEBI/CHEBI:16466"/></rdf:Bag></bqbiol:is><bqbiol:is>**

**<rdf:Bag>**

**<rdf:li rdf:resource="http://identifiers.org/INCHI/InChI=1S/C27H46O4/c1-16(15-28)6-5-7-17(2)20-8-9-21-25-22(14-24(31)27(20,21)4)26(3)11-10-19(29)12-18(26)13-23(25)30/h15-25,29-31H,5-14H2,1-4H3/t16?,17-,18+,19-,20-,21+,22+,23-,24+,25+,26+,27-/m1/s1"/></rdf:Bag></bqbiol:is></rdf:Description></rdf:RDF>**

**</annotation>**

**</species>**

**<species id="M_HC01323" constant="false" hasOnlySubstanceUnits="false" name="(R)-3-Hydroxyoctanoyl-ACP" metaid="3cc9a3d0-362b-4ca8-b701-bedf48ba9644" boundaryCondition="false" compartment="metaComp">**

**<notes>**

**<body xmlns="http://www.w3.org/1999/xhtml">**

**<p>CHARGE: 0</p>**

**<p>PUBCHEM.COMPOUND: 7210</p>**

**<p>KEGG.COMPOUND: C04620</p>**

**</body>**

**</notes>**

**<annotation>**

**<rdf:RDF xmlns:rdf="http://www.w3.org/1999/02/22-rdf-syntax-ns#" xmlns:bqmodel="http://biomodels.net/model-qualifiers/" xmlns:bqbiol="http://biomodels.net/biology-qualifiers/">**

**<rdf:Description rdf:about="_3cc9a3d0-362b-4ca8-b701-bedf48ba9644">**

**<bqbiol:is>**

**<rdf:Bag>**

**<rdf:li rdf:resource="http://identifiers.org/PUBCHEM.COMPOUND/7210"/></rdf:Bag></bqbiol:is><bqbiol:is>**

**<rdf:Bag>**

**<rdf:li rdf:resource="http://identifiers.org/KEGG.COMPOUND/C04620"/></rdf:Bag></bqbiol:is></rdf:Description></rdf:RDF>**

**</annotation>**

**</species>**

**<species id="M_amet" constant="false" hasOnlySubstanceUnits="false" name="S-adenosyl-L-methionine" metaid="3b5c0a8f-ef82-46c6-82d4-27d30e94f6fa" boundaryCondition="false" compartment="metaComp">**

**<notes>**

**<body xmlns="http://www.w3.org/1999/xhtml">**

**<p>FORMULA: C15H23N6O5S</p>**

**<p>CHARGE: 0</p>**

**<p>PUBCHEM.COMPOUND: 34756 || 57417012 || 57416859 || 16757548</p>**

**<p>INCHIKEY: MEFKEPWMEQBLKI-AIRLBKTGSA-N</p>**

**<p>KEGG.COMPOUND: C00019</p>**

**<p>HMDB: HMDB01185</p>**

**<p>CHEBI: CHEBI:15414 || CHEBI:59789 || CHEBI:67040 || CHEBI:33442</p>**

**<p>INCHI: InChI=1S/C15H22N6O5S/c1-27(3-2-7(16)15(24)25)4-8-10(22)11(23)14(26-8)21-6-20-9-12(17)18-5-19-13(9)21/h5-8,10-11,14,22-23H,2-4,16H2,1H3,(H2-,17,18,19,24,25)/t7-,8+,10+,11+,14+,27?/m0/s1 || InChI=1S/C15H22N6O5S/c1-27(3-2-7(16)15(24)25)4-8-10(22)11(23)14(26-8)21-6-20-9-12(17)18-5-19-13(9)21/h5-8,10-11,14,22-23H,2-4,16H2,1H3,(H2-,17,18,19,24,25)/p+1/t7-,8+,10+,11+,14+,27?/m0/s1</p>**

**</body>**

**</notes>**

**<annotation>**

**<rdf:RDF xmlns:rdf="http://www.w3.org/1999/02/22-rdf-syntax-ns#" xmlns:bqmodel="http://biomodels.net/model-qualifiers/" xmlns:bqbiol="http://biomodels.net/biology-qualifiers/">**

**<rdf:Description rdf:about="_3b5c0a8f-ef82-46c6-82d4-27d30e94f6fa">**

**<bqbiol:is>**

**<rdf:Bag>**

**<rdf:li rdf:resource="http://identifiers.org/PUBCHEM.COMPOUND/34756"/></rdf:Bag></bqbiol:is><bqbiol:is>**

**<rdf:Bag>**

**<rdf:li rdf:resource="http://identifiers.org/PUBCHEM.COMPOUND/57417012"/></rdf:Bag></bqbiol:is><bqbiol:is>**

**<rdf:Bag>**

**<rdf:li rdf:resource="http://identifiers.org/PUBCHEM.COMPOUND/57416859"/></rdf:Bag></bqbiol:is><bqbiol:is>**

**<rdf:Bag>**

**<rdf:li rdf:resource="http://identifiers.org/PUBCHEM.COMPOUND/16757548"/></rdf:Bag></bqbiol:is><bqbiol:is>**

**<rdf:Bag>**

**<rdf:li rdf:resource="http://identifiers.org/INCHIKEY/MEFKEPWMEQBLKI-AIRLBKTGSA-N"/></rdf:Bag></bqbiol:is><bqbiol:is>**

**<rdf:Bag>**

**<rdf:li rdf:resource="http://identifiers.org/KEGG.COMPOUND/C00019"/></rdf:Bag></bqbiol:is><bqbiol:is>**

**<rdf:Bag>**

**<rdf:li rdf:resource="http://identifiers.org/HMDB/HMDB01185"/></rdf:Bag></bqbiol:is><bqbiol:is>**

**<rdf:Bag>**

**<rdf:li rdf:resource="http://identifiers.org/CHEBI/CHEBI:15414"/></rdf:Bag></bqbiol:is><bqbiol:is>**

**<rdf:Bag>**

**<rdf:li rdf:resource="http://identifiers.org/CHEBI/CHEBI:59789"/></rdf:Bag></bqbiol:is><bqbiol:is>**

**<rdf:Bag>**

**<rdf:li rdf:resource="http://identifiers.org/CHEBI/CHEBI:67040"/></rdf:Bag></bqbiol:is><bqbiol:is>**

**<rdf:Bag>**

**<rdf:li rdf:resource="http://identifiers.org/CHEBI/CHEBI:33442"/></rdf:Bag></bqbiol:is><bqbiol:is>**

**<rdf:Bag>**

**<rdf:li rdf:resource="http://identifiers.org/INCHI/InChI=1S/C15H22N6O5S/c1-27(3-2-7(16)15(24)25)4-8-10(22)11(23)14(26-8)21-6-20-9-12(17)18-5-19-13(9)21/h5-8,10-11,14,22-23H,2-4,16H2,1H3,(H2-,17,18,19,24,25)/t7-,8+,10+,11+,14+,27?/m0/s1"/></rdf:Bag></bqbiol:is><bqbiol:is>**

**<rdf:Bag>**

**<rdf:li rdf:resource="http://identifiers.org/INCHI/InChI=1S/C15H22N6O5S/c1-27(3-2-7(16)15(24)25)4-8-10(22)11(23)14(26-8)21-6-20-9-12(17)18-5-19-13(9)21/h5-8,10-11,14,22-23H,2-4,16H2,1H3,(H2-,17,18,19,24,25)/p+1/t7-,8+,10+,11+,14+,27?/m0/s1"/></rdf:Bag></bqbiol:is></rdf:Description></rdf:RDF>**

**</annotation>**

**</species>**

**<species id="M_dmhptcrn" constant="false" hasOnlySubstanceUnits="false" name="2,6 dimethylheptanoyl carnitine" metaid="9e548a30-1a40-4358-be96-36aef6da905a" boundaryCondition="false" compartment="metaComp">**

**<notes>**

**<body xmlns="http://www.w3.org/1999/xhtml">**

**<p>FORMULA: C16H35NO4</p>**

**<p>CHARGE: 0</p>**

**<p>PUBCHEM.COMPOUND: 53477823</p>**

**<p>INCHIKEY: QBYXBONNCVATNQ-UHFFFAOYSA-N</p>**

**<p>HMDB: HMDB06320</p>**

**<p>INCHI: InChI=1S/C16H31NO4/c1-12(2)8-7-9-13(3)16(20)21-14(10-15(18)19)11-17(4,5)6/h12-14H,7-11H2,1-6H3 || InChI=1/C16H31NO4/c1-12(2)8-7-9-13(3)16(20)21-14(10-15(18)19)11-17(4,5)6/h12-14H,7-11H2,1-6H3</p>**

**</body>**

**</notes>**

**<annotation>**

**<rdf:RDF xmlns:rdf="http://www.w3.org/1999/02/22-rdf-syntax-ns#" xmlns:bqmodel="http://biomodels.net/model-qualifiers/" xmlns:bqbiol="http://biomodels.net/biology-qualifiers/">**

**<rdf:Description rdf:about="_9e548a30-1a40-4358-be96-36aef6da905a">**

**<bqbiol:is>**

**<rdf:Bag>**

**<rdf:li rdf:resource="http://identifiers.org/PUBCHEM.COMPOUND/53477823"/></rdf:Bag></bqbiol:is><bqbiol:is>**

**<rdf:Bag>**

**<rdf:li rdf:resource="http://identifiers.org/INCHIKEY/QBYXBONNCVATNQ-UHFFFAOYSA-N"/></rdf:Bag></bqbiol:is><bqbiol:is>**

**<rdf:Bag>**

**<rdf:li rdf:resource="http://identifiers.org/HMDB/HMDB06320"/></rdf:Bag></bqbiol:is><bqbiol:is>**

**<rdf:Bag>**

**<rdf:li rdf:resource="http://identifiers.org/INCHI/InChI=1S/C16H31NO4/c1-12(2)8-7-9-13(3)16(20)21-14(10-15(18)19)11-17(4,5)6/h12-14H,7-11H2,1-6H3"/></rdf:Bag></bqbiol:is><bqbiol:is>**

**<rdf:Bag>**

**<rdf:li rdf:resource="http://identifiers.org/INCHI/InChI=1/C16H31NO4/c1-12(2)8-7-9-13(3)16(20)21-14(10-15(18)19)11-17(4,5)6/h12-14H,7-11H2,1-6H3"/></rdf:Bag></bqbiol:is></rdf:Description></rdf:RDF>**

**</annotation>**

**</species>**

**<species id="M_g2m8masn" constant="false" hasOnlySubstanceUnits="false" name="(alpha-D-Glucosyl)2-(alpha-D-mannosyl)8-beta-D-mannosyl-diacetylchitobiosyl-L-asparagine (protein)" metaid="22fa02f3-9d4a-40e3-96ac-f99a2532137a" boundaryCondition="false" compartment="metaComp">**

**<notes>**

**<body xmlns="http://www.w3.org/1999/xhtml">**

**<p>FORMULA: C82H137N2O65X</p>**

**<p>CHARGE: 0</p>**

**<p>KEGG.COMPOUND: C05874</p>**

**</body>**

**</notes>**

**<annotation>**

**<rdf:RDF xmlns:rdf="http://www.w3.org/1999/02/22-rdf-syntax-ns#" xmlns:bqmodel="http://biomodels.net/model-qualifiers/" xmlns:bqbiol="http://biomodels.net/biology-qualifiers/">**

**<rdf:Description rdf:about="_22fa02f3-9d4a-40e3-96ac-f99a2532137a">**

**<bqbiol:is>**

**<rdf:Bag>**

**<rdf:li rdf:resource="http://identifiers.org/KEGG.COMPOUND/C05874"/></rdf:Bag></bqbiol:is></rdf:Description></rdf:RDF>**

**</annotation>**

**</species>**

**<species id="M_inost" constant="false" hasOnlySubstanceUnits="false" name="myo-inositol" metaid="893db0c3-80fa-4dc4-9aca-aa15444be4ac" boundaryCondition="false" compartment="metaComp">**

**<notes>**

**<body xmlns="http://www.w3.org/1999/xhtml">**

**<p>FORMULA: C6H12O6</p>**

**<p>CHARGE: 0</p>**

**<p>PUBCHEM.COMPOUND: 892</p>**

**<p>INCHIKEY: CDAISMWEOUEBRE-GPIVLXJGSA-N</p>**

**<p>KEGG.COMPOUND: C00137</p>**

**<p>HMDB: HMDB00211</p>**

**<p>CHEBI: CHEBI:17268 || CHEBI:24848</p>**

**<p>INCHI: InChI=1S/C6H12O6/c7-1-2(8)4(10)6(12)5(11)3(1)9/h1-12H/t1-,2-,3-,4+,5-,6-</p>**

**</body>**

**</notes>**

**<annotation>**

**<rdf:RDF xmlns:rdf="http://www.w3.org/1999/02/22-rdf-syntax-ns#" xmlns:bqmodel="http://biomodels.net/model-qualifiers/" xmlns:bqbiol="http://biomodels.net/biology-qualifiers/">**

**<rdf:Description rdf:about="_893db0c3-80fa-4dc4-9aca-aa15444be4ac">**

**<bqbiol:is>**

**<rdf:Bag>**

**<rdf:li rdf:resource="http://identifiers.org/PUBCHEM.COMPOUND/892"/></rdf:Bag></bqbiol:is><bqbiol:is>**

**<rdf:Bag>**

**<rdf:li rdf:resource="http://identifiers.org/INCHIKEY/CDAISMWEOUEBRE-GPIVLXJGSA-N"/></rdf:Bag></bqbiol:is><bqbiol:is>**

**<rdf:Bag>**

**<rdf:li rdf:resource="http://identifiers.org/KEGG.COMPOUND/C00137"/></rdf:Bag></bqbiol:is><bqbiol:is>**

**<rdf:Bag>**

**<rdf:li rdf:resource="http://identifiers.org/HMDB/HMDB00211"/></rdf:Bag></bqbiol:is><bqbiol:is>**

**<rdf:Bag>**

**<rdf:li rdf:resource="http://identifiers.org/CHEBI/CHEBI:17268"/></rdf:Bag></bqbiol:is><bqbiol:is>**

**<rdf:Bag>**

**<rdf:li rdf:resource="http://identifiers.org/CHEBI/CHEBI:24848"/></rdf:Bag></bqbiol:is><bqbiol:is>**

**<rdf:Bag>**

**<rdf:li rdf:resource="http://identifiers.org/INCHI/InChI=1S/C6H12O6/c7-1-2(8)4(10)6(12)5(11)3(1)9/h1-12H/t1-,2-,3-,4+,5-,6-"/></rdf:Bag></bqbiol:is></rdf:Description></rdf:RDF>**

**</annotation>**

**</species>**

**<species id="M_dc2coa" constant="false" hasOnlySubstanceUnits="false" name="trans-Dec-2-enoyl-CoA" metaid="40ba9470-1b74-4137-a143-b4cb0208af47" boundaryCondition="false" compartment="metaComp">**

**<notes>**

**<body xmlns="http://www.w3.org/1999/xhtml">**

**<p>FORMULA: C31H48N7O17P3S</p>**

**<p>CHARGE: 0</p>**

**<p>PUBCHEM.COMPOUND: 24883423 || 5280768</p>**

**<p>INCHIKEY: MGNBGCRQQFMNBM-YJHHLLFWSA-N</p>**

**<p>KEGG.COMPOUND: C05275</p>**

**<p>HMDB: HMDB03948</p>**

**<p>CHEBI: CHEBI:10723 || CHEBI:61406</p>**

**<p>INCHI: InChI=1S/C31H52N7O17P3S/c1-4-5-6-7-8-9-10-11-22(40)59-15-14-33-21(39)12-13-34-29(43)26(42)31(2,3)17-52-58(49,50)55-57(47,48)51-16-20-25(54-56(44,45)46)24(41)30(53-20)38-19-37-23-27(32)35-18-36-28(23)38/h10-11,18-20,24-26,30,41-42H,4-9,12-17H2,1-3H3,(H,33,39)(H,34,43)(H,47,48)(H,49,50)(H2,32,35,36)(H2,44,45,46)/b11-10+/t20-,24-,25-,26?,30-/m1/s1 || InChI=1S/C31H52N7O17P3S/c1-4-5-6-7-8-9-10-11-22(40)59-15-14-33-21(39)12-13-34-29(43)26(42)31(2,3)17-52-58(49,50)55-57(47,48)51-16-20-25(54-56(44,45)46)24(41)30(53-20)38-19-37-23-27(32)35-18-36-28(23)38/h10-11,18-20,24-26,30,41-42H,4-9,12-17H2,1-3H3,(H,33,39)(H,34,43)(H,47,48)(H,49,50)(H2,32,35,36)(H2,44,45,46)/b11-10+/t20-,24-,25-,26+,30-/m1/s1 || InChI=1S/C31H52N7O17P3S/c1-4-5-6-7-8-9-10-11-22(40)59-15-14-33-21(39)12-13-34-29(43)26(42)31(2,3)17-52-58(49,50)55-57(47,48)51-16-20-25(54-56(44,45)46)24(41)30(53-20)38-19-37-23-27(32)35-18-36-28(23)38/h10-11,18-20,24-26,30,41-42H,4-9,12-17H2,1-3H3,(H,33,39)(H,34,43)(H,47,48)(H,49,50)(H2,32,35,36)(H2,44,45,46)/p-4/b11-10+/t20-,24-,25-,26+,30-/m1/s1</p>**

**</body>**

**</notes>**

**<annotation>**

**<rdf:RDF xmlns:rdf="http://www.w3.org/1999/02/22-rdf-syntax-ns#" xmlns:bqmodel="http://biomodels.net/model-qualifiers/" xmlns:bqbiol="http://biomodels.net/biology-qualifiers/">**

**<rdf:Description rdf:about="_40ba9470-1b74-4137-a143-b4cb0208af47">**

**<bqbiol:is>**

**<rdf:Bag>**

**<rdf:li rdf:resource="http://identifiers.org/PUBCHEM.COMPOUND/24883423"/></rdf:Bag></bqbiol:is><bqbiol:is>**

**<rdf:Bag>**

**<rdf:li rdf:resource="http://identifiers.org/PUBCHEM.COMPOUND/5280768"/></rdf:Bag></bqbiol:is><bqbiol:is>**

**<rdf:Bag>**

**<rdf:li rdf:resource="http://identifiers.org/INCHIKEY/MGNBGCRQQFMNBM-YJHHLLFWSA-N"/></rdf:Bag></bqbiol:is><bqbiol:is>**

**<rdf:Bag>**

**<rdf:li rdf:resource="http://identifiers.org/KEGG.COMPOUND/C05275"/></rdf:Bag></bqbiol:is><bqbiol:is>**

**<rdf:Bag>**

**<rdf:li rdf:resource="http://identifiers.org/HMDB/HMDB03948"/></rdf:Bag></bqbiol:is><bqbiol:is>**

**<rdf:Bag>**

**<rdf:li rdf:resource="http://identifiers.org/CHEBI/CHEBI:10723"/></rdf:Bag></bqbiol:is><bqbiol:is>**

**<rdf:Bag>**

**<rdf:li rdf:resource="http://identifiers.org/CHEBI/CHEBI:61406"/></rdf:Bag></bqbiol:is><bqbiol:is>**

**<rdf:Bag>**

**<rdf:li rdf:resource="http://identifiers.org/INCHI/InChI=1S/C31H52N7O17P3S/c1-4-5-6-7-8-9-10-11-22(40)59-15-14-33-21(39)12-13-34-29(43)26(42)31(2,3)17-52-58(49,50)55-57(47,48)51-16-20-25(54-56(44,45)46)24(41)30(53-20)38-19-37-23-27(32)35-18-36-28(23)38/h10-11,18-20,24-26,30,41-42H,4-9,12-17H2,1-3H3,(H,33,39)(H,34,43)(H,47,48)(H,49,50)(H2,32,35,36)(H2,44,45,46)/b11-10+/t20-,24-,25-,26?,30-/m1/s1"/></rdf:Bag></bqbiol:is><bqbiol:is>**

**<rdf:Bag>**

**<rdf:li rdf:resource="http://identifiers.org/INCHI/InChI=1S/C31H52N7O17P3S/c1-4-5-6-7-8-9-10-11-22(40)59-15-14-33-21(39)12-13-34-29(43)26(42)31(2,3)17-52-58(49,50)55-57(47,48)51-16-20-25(54-56(44,45)46)24(41)30(53-20)38-19-37-23-27(32)35-18-36-28(23)38/h10-11,18-20,24-26,30,41-42H,4-9,12-17H2,1-3H3,(H,33,39)(H,34,43)(H,47,48)(H,49,50)(H2,32,35,36)(H2,44,45,46)/b11-10+/t20-,24-,25-,26+,30-/m1/s1"/></rdf:Bag></bqbiol:is><bqbiol:is>**

**<rdf:Bag>**

**<rdf:li rdf:resource="http://identifiers.org/INCHI/InChI=1S/C31H52N7O17P3S/c1-4-5-6-7-8-9-10-11-22(40)59-15-14-33-21(39)12-13-34-29(43)26(42)31(2,3)17-52-58(49,50)55-57(47,48)51-16-20-25(54-56(44,45)46)24(41)30(53-20)38-19-37-23-27(32)35-18-36-28(23)38/h10-11,18-20,24-26,30,41-42H,4-9,12-17H2,1-3H3,(H,33,39)(H,34,43)(H,47,48)(H,49,50)(H2,32,35,36)(H2,44,45,46)/p-4/b11-10+/t20-,24-,25-,26+,30-/m1/s1"/></rdf:Bag></bqbiol:is></rdf:Description></rdf:RDF>**

**</annotation>**

**</species>**

**<species id="M_CE1401" constant="false" hasOnlySubstanceUnits="false" name="homocysteine thiolactone" metaid="eeb96f15-0589-449b-b85f-6edb617c99a1" boundaryCondition="false" compartment="metaComp">**

**<notes>**

**<body xmlns="http://www.w3.org/1999/xhtml">**

**<p>FORMULA: C4H8NOS</p>**

**<p>CHARGE: 0</p>**

**<p>PUBCHEM.COMPOUND: 107712 || 134505</p>**

**<p>INCHIKEY: KIWQWJKWBHZMDT-UHFFFAOYSA-N</p>**

**<p>HMDB: HMDB02287</p>**

**<p>CHEBI: CHEBI:60315</p>**

**<p>INCHI: InChI=1S/C4H7NOS/c5-3-1-2-7-4(3)6/h3H,1-2,5H2/t3-/m0/s1 || InChI=1S/C4H7NOS/c5-3-1-2-7-4(3)6/h3H,1-2,5H2</p>**

**</body>**

**</notes>**

**<annotation>**

**<rdf:RDF xmlns:rdf="http://www.w3.org/1999/02/22-rdf-syntax-ns#" xmlns:bqmodel="http://biomodels.net/model-qualifiers/" xmlns:bqbiol="http://biomodels.net/biology-qualifiers/">**

**<rdf:Description rdf:about="eeb96f15-0589-449b-b85f-6edb617c99a1">**

**<bqbiol:is>**

**<rdf:Bag>**

**<rdf:li rdf:resource="http://identifiers.org/PUBCHEM.COMPOUND/107712"/></rdf:Bag></bqbiol:is><bqbiol:is>**

**<rdf:Bag>**

**<rdf:li rdf:resource="http://identifiers.org/PUBCHEM.COMPOUND/134505"/></rdf:Bag></bqbiol:is><bqbiol:is>**

**<rdf:Bag>**

**<rdf:li rdf:resource="http://identifiers.org/INCHIKEY/KIWQWJKWBHZMDT-UHFFFAOYSA-N"/></rdf:Bag></bqbiol:is><bqbiol:is>**

**<rdf:Bag>**

**<rdf:li rdf:resource="http://identifiers.org/HMDB/HMDB02287"/></rdf:Bag></bqbiol:is><bqbiol:is>**

**<rdf:Bag>**

**<rdf:li rdf:resource="http://identifiers.org/CHEBI/CHEBI:60315"/></rdf:Bag></bqbiol:is><bqbiol:is>**

**<rdf:Bag>**

**<rdf:li rdf:resource="http://identifiers.org/INCHI/InChI=1S/C4H7NOS/c5-3-1-2-7-4(3)6/h3H,1-2,5H2/t3-/m0/s1"/></rdf:Bag></bqbiol:is><bqbiol:is>**

**<rdf:Bag>**

**<rdf:li rdf:resource="http://identifiers.org/INCHI/InChI=1S/C4H7NOS/c5-3-1-2-7-4(3)6/h3H,1-2,5H2"/></rdf:Bag></bqbiol:is></rdf:Description></rdf:RDF>**

**</annotation>**

**</species>**

**<species id="M_acglcgal14acglcgalgluside_hs" constant="false" hasOnlySubstanceUnits="false" name="nLc5Cer" metaid="d6f1d7e7-2b95-41fa-a2ec-1133ff524147" boundaryCondition="false" compartment="metaComp">**

**<notes>**

**<body xmlns="http://www.w3.org/1999/xhtml">**

**<p>FORMULA: C52H92N3O27FULLRCO</p>**

**<p>CHARGE: 0</p>**

**</body>**

**</notes>**

**<annotation>**

**<rdf:RDF xmlns:rdf="http://www.w3.org/1999/02/22-rdf-syntax-ns#" xmlns:bqmodel="http://biomodels.net/model-qualifiers/" xmlns:bqbiol="http://biomodels.net/biology-qualifiers/">**

**<rdf:Description rdf:about="d6f1d7e7-2b95-41fa-a2ec-1133ff524147"/></rdf:RDF>**

**</annotation>**

**</species>**

**<species id="M_2octpencoa" constant="false" hasOnlySubstanceUnits="false" name="2,6,9,12,15-octadecapentenoylcoa" metaid="1c3410ba-e76a-4e87-bb2c-8a1649243bb2" boundaryCondition="false" compartment="metaComp">**

**<notes>**

**<body xmlns="http://www.w3.org/1999/xhtml">**

**<p>FORMULA: C39H56N7O17P3S</p>**

**<p>CHARGE: 0</p>**

**</body>**

**</notes>**

**<annotation>**

**<rdf:RDF xmlns:rdf="http://www.w3.org/1999/02/22-rdf-syntax-ns#" xmlns:bqmodel="http://biomodels.net/model-qualifiers/" xmlns:bqbiol="http://biomodels.net/biology-qualifiers/">**

**<rdf:Description rdf:about="_1c3410ba-e76a-4e87-bb2c-8a1649243bb2"/></rdf:RDF>**

**</annotation>**

**</species>**

**<species id="M_mi3456p" constant="false" hasOnlySubstanceUnits="false" name="1D-myo-inositol 3,4,5,6-tetrakisphosphate(8-)" metaid="fd5073d4-619a-447c-b40f-fef9612081f9" boundaryCondition="false" compartment="metaComp">**

**<notes>**

**<body xmlns="http://www.w3.org/1999/xhtml">**

**<p>FORMULA: C6H8O18P4</p>**

**<p>CHARGE: 0</p>**

**<p>PUBCHEM.COMPOUND: 121920</p>**

**<p>KEGG.COMPOUND: C04520</p>**

**<p>HMDB: HMDB03848</p>**

**<p>CHEBI: CHEBI:15844 || CHEBI:57539</p>**

**<p>INCHI: InChI=1S/C6H16O18P4/c7-1-2(8)4(22-26(12,13)14)6(24-28(18,19)20)5(23-27(15,16)17)3(1)21-25(9,10)11/h1-8H,(H2,9,10,11)(H2,12,13,14)(H2,15,16,17)(H2,18,19,20)/t1-,2+,3-,4-,5+,6+/m0/s1 || InChI=1S/C6H16O18P4/c7-1-2(8)4(22-26(12,13)14)6(24-28(18,19)20)5(23-27(15,16)17)3(1)21-25(9,10)11/h1-8H,(H2,9,10,11)(H2,12,13,14)(H2,15,16,17)(H2,18,19,20)/p-8/t1-,2+,3-,4-,5+,6+/m0/s1</p>**

**</body>**

**</notes>**

**<annotation>**

**<rdf:RDF xmlns:rdf="http://www.w3.org/1999/02/22-rdf-syntax-ns#" xmlns:bqmodel="http://biomodels.net/model-qualifiers/" xmlns:bqbiol="http://biomodels.net/biology-qualifiers/">**

**<rdf:Description rdf:about="fd5073d4-619a-447c-b40f-fef9612081f9">**

**<bqbiol:is>**

**<rdf:Bag>**

**<rdf:li rdf:resource="http://identifiers.org/PUBCHEM.COMPOUND/121920"/></rdf:Bag></bqbiol:is><bqbiol:is>**

**<rdf:Bag>**

**<rdf:li rdf:resource="http://identifiers.org/KEGG.COMPOUND/C04520"/></rdf:Bag></bqbiol:is><bqbiol:is>**

**<rdf:Bag>**

**<rdf:li rdf:resource="http://identifiers.org/HMDB/HMDB03848"/></rdf:Bag></bqbiol:is><bqbiol:is>**

**<rdf:Bag>**

**<rdf:li rdf:resource="http://identifiers.org/CHEBI/CHEBI:15844"/></rdf:Bag></bqbiol:is><bqbiol:is>**

**<rdf:Bag>**

**<rdf:li rdf:resource="http://identifiers.org/CHEBI/CHEBI:57539"/></rdf:Bag></bqbiol:is><bqbiol:is>**

**<rdf:Bag>**

**<rdf:li rdf:resource="http://identifiers.org/INCHI/InChI=1S/C6H16O18P4/c7-1-2(8)4(22-26(12,13)14)6(24-28(18,19)20)5(23-27(15,16)17)3(1)21-25(9,10)11/h1-8H,(H2,9,10,11)(H2,12,13,14)(H2,15,16,17)(H2,18,19,20)/t1-,2+,3-,4-,5+,6+/m0/s1"/></rdf:Bag></bqbiol:is><bqbiol:is>**

**<rdf:Bag>**

**<rdf:li rdf:resource="http://identifiers.org/INCHI/InChI=1S/C6H16O18P4/c7-1-2(8)4(22-26(12,13)14)6(24-28(18,19)20)5(23-27(15,16)17)3(1)21-25(9,10)11/h1-8H,(H2,9,10,11)(H2,12,13,14)(H2,15,16,17)(H2,18,19,20)/p-8/t1-,2+,3-,4-,5+,6+/m0/s1"/></rdf:Bag></bqbiol:is></rdf:Description></rdf:RDF>**

**</annotation>**

**</species>**

**<species id="M_atp" constant="false" hasOnlySubstanceUnits="false" name="ATP(4-)" metaid="422acde3-f3a4-431c-a1a2-070041cd79b0" boundaryCondition="false" compartment="metaComp">**

**<notes>**

**<body xmlns="http://www.w3.org/1999/xhtml">**

**<p>FORMULA: C10H12N5O13P3</p>**

**<p>CHARGE: 0</p>**

**<p>PUBCHEM.COMPOUND: 5957</p>**

**<p>KEGG.COMPOUND: C00002</p>**

**<p>HMDB: HMDB00538</p>**

**<p>CHEBI: CHEBI:30616 || CHEBI:15422 || CHEBI:57299</p>**

**<p>INCHI: InChI=1S/C10H16N5O13P3/c11-8-5-9(13-2-12-8)15(3-14-5)10-7(17)6(16)4(26-10)1-25-30(21,22)28-31(23,24)27-29(18,19)20/h2-4,6-7,10,16-17H,1H2,(H,21,22)(H,23,24)(H2,11,12,13)(H2,18,19,20)/p-3/t4-,6-,7-,10-/m1/s1 || InChI=1S/C10H16N5O13P3/c11-8-5-9(13-2-12-8)15(3-14-5)10-7(17)6(16)4(26-10)1-25-30(21,22)28-31(23,24)27-29(18,19)20/h2-4,6-7,10,16-17H,1H2,(H,21,22)(H,23,24)(H2,11,12,13)(H2,18,19,20)/t4-,6-,7-,10-/m1/s1 || InChI=1S/C10H16N5O13P3/c11-8-5-9(13-2-12-8)15(3-14-5)10-7(17)6(16)4(26-10)1-25-30(21,22)28-31(23,24)27-29(18,19)20/h2-4,6-7,10,16-17H,1H2,(H,21,22)(H,23,24)(H2,11,12,13)(H2,18,19,20)/p-4/t4-,6-,7-,10-/m1/s1</p>**

**</body>**

**</notes>**

**<annotation>**

**<rdf:RDF xmlns:rdf="http://www.w3.org/1999/02/22-rdf-syntax-ns#" xmlns:bqmodel="http://biomodels.net/model-qualifiers/" xmlns:bqbiol="http://biomodels.net/biology-qualifiers/">**

**<rdf:Description rdf:about="_422acde3-f3a4-431c-a1a2-070041cd79b0">**

**<bqbiol:is>**

**<rdf:Bag>**

**<rdf:li rdf:resource="http://identifiers.org/PUBCHEM.COMPOUND/5957"/></rdf:Bag></bqbiol:is><bqbiol:is>**

**<rdf:Bag>**

**<rdf:li rdf:resource="http://identifiers.org/KEGG.COMPOUND/C00002"/></rdf:Bag></bqbiol:is><bqbiol:is>**

**<rdf:Bag>**

**<rdf:li rdf:resource="http://identifiers.org/HMDB/HMDB00538"/></rdf:Bag></bqbiol:is><bqbiol:is>**

**<rdf:Bag>**

**<rdf:li rdf:resource="http://identifiers.org/CHEBI/CHEBI:30616"/></rdf:Bag></bqbiol:is><bqbiol:is>**

**<rdf:Bag>**

**<rdf:li rdf:resource="http://identifiers.org/CHEBI/CHEBI:15422"/></rdf:Bag></bqbiol:is><bqbiol:is>**

**<rdf:Bag>**

**<rdf:li rdf:resource="http://identifiers.org/CHEBI/CHEBI:57299"/></rdf:Bag></bqbiol:is><bqbiol:is>**

**<rdf:Bag>**

**<rdf:li rdf:resource="http://identifiers.org/INCHI/InChI=1S/C10H16N5O13P3/c11-8-5-9(13-2-12-8)15(3-14-5)10-7(17)6(16)4(26-10)1-25-30(21,22)28-31(23,24)27-29(18,19)20/h2-4,6-7,10,16-17H,1H2,(H,21,22)(H,23,24)(H2,11,12,13)(H2,18,19,20)/p-3/t4-,6-,7-,10-/m1/s1"/></rdf:Bag></bqbiol:is><bqbiol:is>**

**<rdf:Bag>**

**<rdf:li rdf:resource="http://identifiers.org/INCHI/InChI=1S/C10H16N5O13P3/c11-8-5-9(13-2-12-8)15(3-14-5)10-7(17)6(16)4(26-10)1-25-30(21,22)28-31(23,24)27-29(18,19)20/h2-4,6-7,10,16-17H,1H2,(H,21,22)(H,23,24)(H2,11,12,13)(H2,18,19,20)/t4-,6-,7-,10-/m1/s1"/></rdf:Bag></bqbiol:is><bqbiol:is>**

**<rdf:Bag>**

**<rdf:li rdf:resource="http://identifiers.org/INCHI/InChI=1S/C10H16N5O13P3/c11-8-5-9(13-2-12-8)15(3-14-5)10-7(17)6(16)4(26-10)1-25-30(21,22)28-31(23,24)27-29(18,19)20/h2-4,6-7,10,16-17H,1H2,(H,21,22)(H,23,24)(H2,11,12,13)(H2,18,19,20)/p-4/t4-,6-,7-,10-/m1/s1"/></rdf:Bag></bqbiol:is></rdf:Description></rdf:RDF>**

**</annotation>**

**</species>**

**<species id="M_C05463" constant="false" hasOnlySubstanceUnits="false" name="taurodeoxycholate" metaid="c637494a-e933-4683-a9d6-5e8a6030004a" boundaryCondition="false" compartment="metaComp">**

**<notes>**

**<body xmlns="http://www.w3.org/1999/xhtml">**

**<p>FORMULA: C26H44NO6S</p>**

**<p>CHARGE: 0</p>**

**<p>PUBCHEM.COMPOUND: 2733768</p>**

**<p>INCHIKEY: AWDRATDZQPNJFN-GHMNEPPTSA-N</p>**

**<p>KEGG.COMPOUND: C05463</p>**

**<p>HMDB: HMDB04011 || HMDB00896</p>**

**<p>CHEBI: CHEBI:36261 || CHEBI:9410</p>**

**<p>INCHI: InChI=1S/C26H45NO6S/c1-16(4-9-24(30)27-12-13-34(31,32)33)20-7-8-21-19-6-5-17-14-18(28)10-11-25(17,2)22(19)15-23(29)26(20,21)3/h16-23,28-29H,4-15H2,1-3H3,(H,27,30)(H,31,32,33)/t16?,17?,18-,19?,20?,21?,22?,23+,25+,26-/m1/s1 || InChI=1S/C26H45NO6S/c1-16(4-9-24(30)27-12-13-34(31,32)33)20-7-8-21-19-6-5-17-14-18(28)10-11-25(17,2)22(19)15-23(29)26(20,21)3/h16-23,28-29H,4-15H2,1-3H3,(H,27,30)(H,31,32,33)/p-1/t16-,17-,18-,19+,20-,21+,22+,23+,25+,26-/m1/s1 || InChI=1S/C26H45NO6S/c1-16(4-9-24(30)27-12-13-34(31,32)33)20-7-8-21-19-6-5-17-14-18(28)10-11-25(17,2)22(19)15-23(29)26(20,21)3/h16-23,28-29H,4-15H2,1-3H3,(H,27,30)(H,31,32,33)/t16-,17-,18-,19+,20-,21+,22+,23+,25+,26-/m1/s1</p>**

**</body>**

**</notes>**

**<annotation>**

**<rdf:RDF xmlns:rdf="http://www.w3.org/1999/02/22-rdf-syntax-ns#" xmlns:bqmodel="http://biomodels.net/model-qualifiers/" xmlns:bqbiol="http://biomodels.net/biology-qualifiers/">**

**<rdf:Description rdf:about="c637494a-e933-4683-a9d6-5e8a6030004a">**

**<bqbiol:is>**

**<rdf:Bag>**

**<rdf:li rdf:resource="http://identifiers.org/PUBCHEM.COMPOUND/2733768"/></rdf:Bag></bqbiol:is><bqbiol:is>**

**<rdf:Bag>**

**<rdf:li rdf:resource="http://identifiers.org/INCHIKEY/AWDRATDZQPNJFN-GHMNEPPTSA-N"/></rdf:Bag></bqbiol:is><bqbiol:is>**

**<rdf:Bag>**

**<rdf:li rdf:resource="http://identifiers.org/KEGG.COMPOUND/C05463"/></rdf:Bag></bqbiol:is><bqbiol:is>**

**<rdf:Bag>**

**<rdf:li rdf:resource="http://identifiers.org/HMDB/HMDB04011"/></rdf:Bag></bqbiol:is><bqbiol:is>**

**<rdf:Bag>**

**<rdf:li rdf:resource="http://identifiers.org/HMDB/HMDB00896"/></rdf:Bag></bqbiol:is><bqbiol:is>**

**<rdf:Bag>**

**<rdf:li rdf:resource="http://identifiers.org/CHEBI/CHEBI:36261"/></rdf:Bag></bqbiol:is><bqbiol:is>**

**<rdf:Bag>**

**<rdf:li rdf:resource="http://identifiers.org/CHEBI/CHEBI:9410"/></rdf:Bag></bqbiol:is><bqbiol:is>**

**<rdf:Bag>**

**<rdf:li rdf:resource="http://identifiers.org/INCHI/InChI=1S/C26H45NO6S/c1-16(4-9-24(30)27-12-13-34(31,32)33)20-7-8-21-19-6-5-17-14-18(28)10-11-25(17,2)22(19)15-23(29)26(20,21)3/h16-23,28-29H,4-15H2,1-3H3,(H,27,30)(H,31,32,33)/t16?,17?,18-,19?,20?,21?,22?,23+,25+,26-/m1/s1"/></rdf:Bag></bqbiol:is><bqbiol:is>**

**<rdf:Bag>**

**<rdf:li rdf:resource="http://identifiers.org/INCHI/InChI=1S/C26H45NO6S/c1-16(4-9-24(30)27-12-13-34(31,32)33)20-7-8-21-19-6-5-17-14-18(28)10-11-25(17,2)22(19)15-23(29)26(20,21)3/h16-23,28-29H,4-15H2,1-3H3,(H,27,30)(H,31,32,33)/p-1/t16-,17-,18-,19+,20-,21+,22+,23+,25+,26-/m1/s1"/></rdf:Bag></bqbiol:is><bqbiol:is>**

**<rdf:Bag>**

**<rdf:li rdf:resource="http://identifiers.org/INCHI/InChI=1S/C26H45NO6S/c1-16(4-9-24(30)27-12-13-34(31,32)33)20-7-8-21-19-6-5-17-14-18(28)10-11-25(17,2)22(19)15-23(29)26(20,21)3/h16-23,28-29H,4-15H2,1-3H3,(H,27,30)(H,31,32,33)/t16-,17-,18-,19+,20-,21+,22+,23+,25+,26-/m1/s1"/></rdf:Bag></bqbiol:is></rdf:Description></rdf:RDF>**

**</annotation>**

**</species>**

**<species id="M_ga2_hs" constant="false" hasOnlySubstanceUnits="false" name="GA2" metaid="c34ca589-838e-4daa-95c4-8d5677588c72" boundaryCondition="false" compartment="metaComp">**

**<notes>**

**<body xmlns="http://www.w3.org/1999/xhtml">**

**<p>FORMULA: C38H69N2O17FULLRCO</p>**

**<p>CHARGE: 0</p>**

**<p>CHEBI: CHEBI:465284</p>**

**</body>**

**</notes>**

**<annotation>**

**<rdf:RDF xmlns:rdf="http://www.w3.org/1999/02/22-rdf-syntax-ns#" xmlns:bqmodel="http://biomodels.net/model-qualifiers/" xmlns:bqbiol="http://biomodels.net/biology-qualifiers/">**

**<rdf:Description rdf:about="c34ca589-838e-4daa-95c4-8d5677588c72">**

**<bqbiol:is>**

**<rdf:Bag>**

**<rdf:li rdf:resource="http://identifiers.org/CHEBI/CHEBI:465284"/></rdf:Bag></bqbiol:is></rdf:Description></rdf:RDF>**

**</annotation>**

**</species>**

**<species id="M_ptth" constant="false" hasOnlySubstanceUnits="false" name="pantetheine" metaid="f05f9c67-f644-4edd-b75a-f1b3cfac453a" boundaryCondition="false" compartment="metaComp">**

**<notes>**

**<body xmlns="http://www.w3.org/1999/xhtml">**

**<p>FORMULA: C11H22N2O4S</p>**

**<p>CHARGE: 0</p>**

**<p>PUBCHEM.COMPOUND: 479 || 439322</p>**

**<p>INCHIKEY: ZNXZGRMVNNHPCA-VIFPVBQESA-N</p>**

**<p>KEGG.COMPOUND: C00831</p>**

**<p>HMDB: HMDB03426</p>**

**<p>CHEBI: CHEBI:16753</p>**

**<p>INCHI: InChI=1S/C11H22N2O4S/c1-11(2,7-14)9(16)10(17)13-4-3-8(15)12-5-6-18/h9,14,16,18H,3-7H2,1-2H3,(H,12,15)(H,13,17)/t9-/m0/s1 || InChI=1S/C11H22N2O4S/c1-11(2,7-14)9(16)10(17)13-4-3-8(15)12-5-6-18/h9,14,16,18H,3-7H2,1-2H3,(H,12,15)(H,13,17)</p>**

**</body>**

**</notes>**

**<annotation>**

**<rdf:RDF xmlns:rdf="http://www.w3.org/1999/02/22-rdf-syntax-ns#" xmlns:bqmodel="http://biomodels.net/model-qualifiers/" xmlns:bqbiol="http://biomodels.net/biology-qualifiers/">**

**<rdf:Description rdf:about="f05f9c67-f644-4edd-b75a-f1b3cfac453a">**

**<bqbiol:is>**

**<rdf:Bag>**

**<rdf:li rdf:resource="http://identifiers.org/PUBCHEM.COMPOUND/479"/></rdf:Bag></bqbiol:is><bqbiol:is>**

**<rdf:Bag>**

**<rdf:li rdf:resource="http://identifiers.org/PUBCHEM.COMPOUND/439322"/></rdf:Bag></bqbiol:is><bqbiol:is>**

**<rdf:Bag>**

**<rdf:li rdf:resource="http://identifiers.org/INCHIKEY/ZNXZGRMVNNHPCA-VIFPVBQESA-N"/></rdf:Bag></bqbiol:is><bqbiol:is>**

**<rdf:Bag>**

**<rdf:li rdf:resource="http://identifiers.org/KEGG.COMPOUND/C00831"/></rdf:Bag></bqbiol:is><bqbiol:is>**

**<rdf:Bag>**

**<rdf:li rdf:resource="http://identifiers.org/HMDB/HMDB03426"/></rdf:Bag></bqbiol:is><bqbiol:is>**

**<rdf:Bag>**

**<rdf:li rdf:resource="http://identifiers.org/CHEBI/CHEBI:16753"/></rdf:Bag></bqbiol:is><bqbiol:is>**

**<rdf:Bag>**

**<rdf:li rdf:resource="http://identifiers.org/INCHI/InChI=1S/C11H22N2O4S/c1-11(2,7-14)9(16)10(17)13-4-3-8(15)12-5-6-18/h9,14,16,18H,3-7H2,1-2H3,(H,12,15)(H,13,17)/t9-/m0/s1"/></rdf:Bag></bqbiol:is><bqbiol:is>**

**<rdf:Bag>**

**<rdf:li rdf:resource="http://identifiers.org/INCHI/InChI=1S/C11H22N2O4S/c1-11(2,7-14)9(16)10(17)13-4-3-8(15)12-5-6-18/h9,14,16,18H,3-7H2,1-2H3,(H,12,15)(H,13,17)"/></rdf:Bag></bqbiol:is></rdf:Description></rdf:RDF>**

**</annotation>**

**</species>**

**<species id="M_cs_a_b_pre3" constant="false" hasOnlySubstanceUnits="false" name="chondroitin sulfate A (GalNAc4S-GlcA) and B (IdoA2S-GalNAc4S), precursor 3" metaid="1e182232-bcdf-401e-8514-8ee06f571f03" boundaryCondition="false" compartment="metaComp">**

**<notes>**

**<body xmlns="http://www.w3.org/1999/xhtml">**

**<p>FORMULA: C45H67N2O42S2X</p>**

**<p>CHARGE: 0</p>**

**</body>**

**</notes>**

**<annotation>**

**<rdf:RDF xmlns:rdf="http://www.w3.org/1999/02/22-rdf-syntax-ns#" xmlns:bqmodel="http://biomodels.net/model-qualifiers/" xmlns:bqbiol="http://biomodels.net/biology-qualifiers/">**

**<rdf:Description rdf:about="_1e182232-bcdf-401e-8514-8ee06f571f03"/></rdf:RDF>**

**</annotation>**

**</species>**

**<species id="M_cs_a_b_pre2" constant="false" hasOnlySubstanceUnits="false" name="chondroitin sulfate A (GalNAc4S-GlcA) and B (IdoA2S-GalNAc4S), precursor 2" metaid="e7726680-c907-4b8b-8f56-75d1eadafbcc" boundaryCondition="false" compartment="metaComp">**

**<notes>**

**<body xmlns="http://www.w3.org/1999/xhtml">**

**<p>FORMULA: C37H54NO37S2X</p>**

**<p>CHARGE: 0</p>**

**</body>**

**</notes>**

**<annotation>**

**<rdf:RDF xmlns:rdf="http://www.w3.org/1999/02/22-rdf-syntax-ns#" xmlns:bqmodel="http://biomodels.net/model-qualifiers/" xmlns:bqbiol="http://biomodels.net/biology-qualifiers/">**

**<rdf:Description rdf:about="e7726680-c907-4b8b-8f56-75d1eadafbcc"/></rdf:RDF>**

**</annotation>**

**</species>**

**<species id="M_CE0347" constant="false" hasOnlySubstanceUnits="false" name="12-hydroxyeicosatetraenoate" metaid="5d5032e8-736e-4668-8ef8-c24a8a7f553f" boundaryCondition="false" compartment="metaComp">**

**<notes>**

**<body xmlns="http://www.w3.org/1999/xhtml">**

**<p>FORMULA: C20H31O3</p>**

**<p>CHARGE: 0</p>**

**<p>PUBCHEM.COMPOUND: 5312983</p>**

**<p>HMDB: HMDB06111</p>**

**<p>INCHI: InChI=1S/C20H32O3/c1-2-3-4-5-10-13-16-19(21)17-14-11-8-6-7-9-12-15-18-20(22)23/h7-11,13-14,17,19,21H,2-6,12,15-16,18H2,1H3,(H,22,23) || InChI=1S/C20H32O3/c1-2-3-4-5-10-13-16-19(21)17-14-11-8-6-7-9-12-15-18-20(22)23/h7-11,13-14,17,19,21H,2-6,12,15-16,18H2,1H3,(H,22,23)/b9-7+,11-8-,13-10-,17-14-</p>**

**</body>**

**</notes>**

**<annotation>**

**<rdf:RDF xmlns:rdf="http://www.w3.org/1999/02/22-rdf-syntax-ns#" xmlns:bqmodel="http://biomodels.net/model-qualifiers/" xmlns:bqbiol="http://biomodels.net/biology-qualifiers/">**

**<rdf:Description rdf:about="_5d5032e8-736e-4668-8ef8-c24a8a7f553f">**

**<bqbiol:is>**

**<rdf:Bag>**

**<rdf:li rdf:resource="http://identifiers.org/PUBCHEM.COMPOUND/5312983"/></rdf:Bag></bqbiol:is><bqbiol:is>**

**<rdf:Bag>**

**<rdf:li rdf:resource="http://identifiers.org/HMDB/HMDB06111"/></rdf:Bag></bqbiol:is><bqbiol:is>**

**<rdf:Bag>**

**<rdf:li rdf:resource="http://identifiers.org/INCHI/InChI=1S/C20H32O3/c1-2-3-4-5-10-13-16-19(21)17-14-11-8-6-7-9-12-15-18-20(22)23/h7-11,13-14,17,19,21H,2-6,12,15-16,18H2,1H3,(H,22,23)"/></rdf:Bag></bqbiol:is><bqbiol:is>**

**<rdf:Bag>**

**<rdf:li rdf:resource="http://identifiers.org/INCHI/InChI=1S/C20H32O3/c1-2-3-4-5-10-13-16-19(21)17-14-11-8-6-7-9-12-15-18-20(22)23/h7-11,13-14,17,19,21H,2-6,12,15-16,18H2,1H3,(H,22,23)/b9-7+,11-8-,13-10-,17-14-"/></rdf:Bag></bqbiol:is></rdf:Description></rdf:RDF>**

**</annotation>**

**</species>**

**<species id="M_23doguln" constant="false" hasOnlySubstanceUnits="false" name="(4R,5S)-4,5,6-trihydroxy-2,3-dioxohexanoate" metaid="168dff8b-441c-438d-8669-cb0840b92c63" boundaryCondition="false" compartment="metaComp">**

**<notes>**

**<body xmlns="http://www.w3.org/1999/xhtml">**

**<p>FORMULA: C6H7O7</p>**

**<p>CHARGE: 0</p>**

**<p>PUBCHEM.COMPOUND: 440390</p>**

**<p>INCHIKEY: GJQWCDSAOUMKSE-STHAYSLISA-N</p>**

**<p>KEGG.COMPOUND: C04575</p>**

**<p>HMDB: HMDB06511 || HMDB05971</p>**

**<p>CHEBI: CHEBI:15622 || CHEBI:57441</p>**

**<p>INCHI: InChI=1S/C6H8O7/c7-1-2(8)3(9)4(10)5(11)6(12)13/h2-3,7-9H,1H2,(H,12,13)/p-1/t2-,3+/m0/s1 || InChI=1S/C6H8O7/c7-1-2(8)3(9)4(10)5(11)6(12)13/h2-3,7-9H,1H2,(H,12,13)/t2-,3+/m0/s1</p>**

**</body>**

**</notes>**

**<annotation>**

**<rdf:RDF xmlns:rdf="http://www.w3.org/1999/02/22-rdf-syntax-ns#" xmlns:bqmodel="http://biomodels.net/model-qualifiers/" xmlns:bqbiol="http://biomodels.net/biology-qualifiers/">**

**<rdf:Description rdf:about="_168dff8b-441c-438d-8669-cb0840b92c63">**

**<bqbiol:is>**

**<rdf:Bag>**

**<rdf:li rdf:resource="http://identifiers.org/PUBCHEM.COMPOUND/440390"/></rdf:Bag></bqbiol:is><bqbiol:is>**

**<rdf:Bag>**

**<rdf:li rdf:resource="http://identifiers.org/INCHIKEY/GJQWCDSAOUMKSE-STHAYSLISA-N"/></rdf:Bag></bqbiol:is><bqbiol:is>**

**<rdf:Bag>**

**<rdf:li rdf:resource="http://identifiers.org/KEGG.COMPOUND/C04575"/></rdf:Bag></bqbiol:is><bqbiol:is>**

**<rdf:Bag>**

**<rdf:li rdf:resource="http://identifiers.org/HMDB/HMDB06511"/></rdf:Bag></bqbiol:is><bqbiol:is>**

**<rdf:Bag>**

**<rdf:li rdf:resource="http://identifiers.org/HMDB/HMDB05971"/></rdf:Bag></bqbiol:is><bqbiol:is>**

**<rdf:Bag>**

**<rdf:li rdf:resource="http://identifiers.org/CHEBI/CHEBI:15622"/></rdf:Bag></bqbiol:is><bqbiol:is>**

**<rdf:Bag>**

**<rdf:li rdf:resource="http://identifiers.org/CHEBI/CHEBI:57441"/></rdf:Bag></bqbiol:is><bqbiol:is>**

**<rdf:Bag>**

**<rdf:li rdf:resource="http://identifiers.org/INCHI/InChI=1S/C6H8O7/c7-1-2(8)3(9)4(10)5(11)6(12)13/h2-3,7-9H,1H2,(H,12,13)/p-1/t2-,3+/m0/s1"/></rdf:Bag></bqbiol:is><bqbiol:is>**

**<rdf:Bag>**

**<rdf:li rdf:resource="http://identifiers.org/INCHI/InChI=1S/C6H8O7/c7-1-2(8)3(9)4(10)5(11)6(12)13/h2-3,7-9H,1H2,(H,12,13)/t2-,3+/m0/s1"/></rdf:Bag></bqbiol:is></rdf:Description></rdf:RDF>**

**</annotation>**

**</species>**

**<species id="M_2mcacn" constant="false" hasOnlySubstanceUnits="false" name="(Z)-but-2-ene-1,2,3-tricarboxylate" metaid="e323a27e-b5e1-4a51-abc6-873aa2f4b209" boundaryCondition="false" compartment="metaComp">**

**<notes>**

**<body xmlns="http://www.w3.org/1999/xhtml">**

**<p>FORMULA: C7H5O6</p>**

**<p>CHARGE: 0</p>**

**<p>PUBCHEM.COMPOUND: 3080625</p>**

**<p>INCHIKEY: NUZLRKBHOBPTQV-ARJAWSKDSA-N</p>**

**<p>KEGG.COMPOUND: C04225</p>**

**<p>HMDB: HMDB06357</p>**

**<p>CHEBI: CHEBI:57872 || CHEBI:16717</p>**

**<p>INCHI: InChI=1S/C7H8O6/c1-3(6(10)11)4(7(12)13)2-5(8)9/h2H2,1H3,(H,8,9)(H,10,11)(H,12,13)/p-3/b4-3- || InChI=1S/C7H8O6/c1-3(6(10)11)4(7(12)13)2-5(8)9/h2H2,1H3,(H,8,9)(H,10,11)(H,12,13)/b4-3-</p>**

**</body>**

**</notes>**

**<annotation>**

**<rdf:RDF xmlns:rdf="http://www.w3.org/1999/02/22-rdf-syntax-ns#" xmlns:bqmodel="http://biomodels.net/model-qualifiers/" xmlns:bqbiol="http://biomodels.net/biology-qualifiers/">**

**<rdf:Description rdf:about="e323a27e-b5e1-4a51-abc6-873aa2f4b209">**

**<bqbiol:is>**

**<rdf:Bag>**

**<rdf:li rdf:resource="http://identifiers.org/PUBCHEM.COMPOUND/3080625"/></rdf:Bag></bqbiol:is><bqbiol:is>**

**<rdf:Bag>**

**<rdf:li rdf:resource="http://identifiers.org/INCHIKEY/NUZLRKBHOBPTQV-ARJAWSKDSA-N"/></rdf:Bag></bqbiol:is><bqbiol:is>**

**<rdf:Bag>**

**<rdf:li rdf:resource="http://identifiers.org/KEGG.COMPOUND/C04225"/></rdf:Bag></bqbiol:is><bqbiol:is>**

**<rdf:Bag>**

**<rdf:li rdf:resource="http://identifiers.org/HMDB/HMDB06357"/></rdf:Bag></bqbiol:is><bqbiol:is>**

**<rdf:Bag>**

**<rdf:li rdf:resource="http://identifiers.org/CHEBI/CHEBI:57872"/></rdf:Bag></bqbiol:is><bqbiol:is>**

**<rdf:Bag>**

**<rdf:li rdf:resource="http://identifiers.org/CHEBI/CHEBI:16717"/></rdf:Bag></bqbiol:is><bqbiol:is>**

**<rdf:Bag>**

**<rdf:li rdf:resource="http://identifiers.org/INCHI/InChI=1S/C7H8O6/c1-3(6(10)11)4(7(12)13)2-5(8)9/h2H2,1H3,(H,8,9)(H,10,11)(H,12,13)/p-3/b4-3-"/></rdf:Bag></bqbiol:is><bqbiol:is>**

**<rdf:Bag>**

**<rdf:li rdf:resource="http://identifiers.org/INCHI/InChI=1S/C7H8O6/c1-3(6(10)11)4(7(12)13)2-5(8)9/h2H2,1H3,(H,8,9)(H,10,11)(H,12,13)/b4-3-"/></rdf:Bag></bqbiol:is></rdf:Description></rdf:RDF>**

**</annotation>**

**</species>**

**<species id="M_succoa" constant="false" hasOnlySubstanceUnits="false" name="Succinyl-CoA" metaid="0082d4c9-1092-4b7e-89c4-0ff08d9bc153" boundaryCondition="false" compartment="metaComp">**

**<notes>**

**<body xmlns="http://www.w3.org/1999/xhtml">**

**<p>FORMULA: C25H35N7O19P3S</p>**

**<p>CHARGE: 0</p>**

**<p>PUBCHEM.COMPOUND: 439161</p>**

**<p>INCHIKEY: VNOYUJKHFWYWIR-ITIYDSSPSA-N</p>**

**<p>KEGG.COMPOUND: C00091</p>**

**<p>HMDB: HMDB01022</p>**

**<p>CHEBI: CHEBI:57292 || CHEBI:15380</p>**

**<p>INCHI: InChI=1S/C25H40N7O19P3S/c1-25(2,20(38)23(39)28-6-5-14(33)27-7-8-55-16(36)4-3-15(34)35)10-48-54(45,46)51-53(43,44)47-9-13-19(50-52(40,41)42)18(37)24(49-13)32-12-31-17-21(26)29-11-30-22(17)32/h11-13,18-20,24,37-38H,3-10H2,1-2H3,(H,27,33)(H,28,39)(H,34,35)(H,43,44)(H,45,46)(H2,26,29,30)(H2,40,41,42)/p-5/t13-,18-,19-,20+,24-/m1/s1 || InChI=1S/C25H40N7O19P3S/c1-25(2,20(38)23(39)28-6-5-14(33)27-7-8-55-16(36)4-3-15(34)35)10-48-54(45,46)51-53(43,44)47-9-13-19(50-52(40,41)42)18(37)24(49-13)32-12-31-17-21(26)29-11-30-22(17)32/h11-13,18-20,24,37-38H,3-10H2,1-2H3,(H,27,33)(H,28,39)(H,34,35)(H,43,44)(H,45,46)(H2,26,29,30)(H2,40,41,42)/t13-,18-,19-,20+,24-/m1/s1 || InChI=1S/C25H40N7O19P3S/c1-25(2,20(38)23(39)28-6-5-14(33)27-7-8-55-16(36)4-3-15(34)35)10-48-54(45,46)51-53(43,44)47-9-13-19(50-52(40,41)42)18(37)24(49-13)32-12-31-17-21(26)29-11-30-22(17)32/h11-13,18-20,24,37-38H,3-10H2,1-2H3,(H,27,33)(H,28,39)(H,34,35)(H,43,44)(H,45,46)(H2,26,29,30)(H2,40,41,42)/t13-,18-,19-,20?,24-/m1/s1</p>**

**</body>**

**</notes>**

**<annotation>**

**<rdf:RDF xmlns:rdf="http://www.w3.org/1999/02/22-rdf-syntax-ns#" xmlns:bqmodel="http://biomodels.net/model-qualifiers/" xmlns:bqbiol="http://biomodels.net/biology-qualifiers/">**

**<rdf:Description rdf:about="_0082d4c9-1092-4b7e-89c4-0ff08d9bc153">**

**<bqbiol:is>**

**<rdf:Bag>**

**<rdf:li rdf:resource="http://identifiers.org/PUBCHEM.COMPOUND/439161"/></rdf:Bag></bqbiol:is><bqbiol:is>**

**<rdf:Bag>**

**<rdf:li rdf:resource="http://identifiers.org/INCHIKEY/VNOYUJKHFWYWIR-ITIYDSSPSA-N"/></rdf:Bag></bqbiol:is><bqbiol:is>**

**<rdf:Bag>**

**<rdf:li rdf:resource="http://identifiers.org/KEGG.COMPOUND/C00091"/></rdf:Bag></bqbiol:is><bqbiol:is>**

**<rdf:Bag>**

**<rdf:li rdf:resource="http://identifiers.org/HMDB/HMDB01022"/></rdf:Bag></bqbiol:is><bqbiol:is>**

**<rdf:Bag>**

**<rdf:li rdf:resource="http://identifiers.org/CHEBI/CHEBI:57292"/></rdf:Bag></bqbiol:is><bqbiol:is>**

**<rdf:Bag>**

**<rdf:li rdf:resource="http://identifiers.org/CHEBI/CHEBI:15380"/></rdf:Bag></bqbiol:is><bqbiol:is>**

**<rdf:Bag>**

**<rdf:li rdf:resource="http://identifiers.org/INCHI/InChI=1S/C25H40N7O19P3S/c1-25(2,20(38)23(39)28-6-5-14(33)27-7-8-55-16(36)4-3-15(34)35)10-48-54(45,46)51-53(43,44)47-9-13-19(50-52(40,41)42)18(37)24(49-13)32-12-31-17-21(26)29-11-30-22(17)32/h11-13,18-20,24,37-38H,3-10H2,1-2H3,(H,27,33)(H,28,39)(H,34,35)(H,43,44)(H,45,46)(H2,26,29,30)(H2,40,41,42)/p-5/t13-,18-,19-,20+,24-/m1/s1"/></rdf:Bag></bqbiol:is><bqbiol:is>**

**<rdf:Bag>**

**<rdf:li rdf:resource="http://identifiers.org/INCHI/InChI=1S/C25H40N7O19P3S/c1-25(2,20(38)23(39)28-6-5-14(33)27-7-8-55-16(36)4-3-15(34)35)10-48-54(45,46)51-53(43,44)47-9-13-19(50-52(40,41)42)18(37)24(49-13)32-12-31-17-21(26)29-11-30-22(17)32/h11-13,18-20,24,37-38H,3-10H2,1-2H3,(H,27,33)(H,28,39)(H,34,35)(H,43,44)(H,45,46)(H2,26,29,30)(H2,40,41,42)/t13-,18-,19-,20+,24-/m1/s1"/></rdf:Bag></bqbiol:is><bqbiol:is>**

**<rdf:Bag>**

**<rdf:li rdf:resource="http://identifiers.org/INCHI/InChI=1S/C25H40N7O19P3S/c1-25(2,20(38)23(39)28-6-5-14(33)27-7-8-55-16(36)4-3-15(34)35)10-48-54(45,46)51-53(43,44)47-9-13-19(50-52(40,41)42)18(37)24(49-13)32-12-31-17-21(26)29-11-30-22(17)32/h11-13,18-20,24,37-38H,3-10H2,1-2H3,(H,27,33)(H,28,39)(H,34,35)(H,43,44)(H,45,46)(H2,26,29,30)(H2,40,41,42)/t13-,18-,19-,20?,24-/m1/s1"/></rdf:Bag></bqbiol:is></rdf:Description></rdf:RDF>**

**</annotation>**

**</species>**

**<species id="M_3ivcoa" constant="false" hasOnlySubstanceUnits="false" name="3-hydroxyisovalerylcoa" metaid="cf442f05-8998-4d2b-9a91-85241368c6fd" boundaryCondition="false" compartment="metaComp">**

**<notes>**

**<body xmlns="http://www.w3.org/1999/xhtml">**

**<p>FORMULA: C26H40N7O18P3S</p>**

**<p>CHARGE: 0</p>**

**<p>PUBCHEM.COMPOUND: 11953876 || 11966188</p>**

**<p>KEGG.COMPOUND: C05998</p>**

**<p>HMDB: HMDB06870</p>**

**<p>CHEBI: CHEBI:28291</p>**

**<p>INCHI: InChI=1S/C26H44N7O18P3S/c1-25(2,20(37)23(38)29-6-5-15(34)28-7-8-55-16(35)9-26(3,4)39)11-48-54(45,46)51-53(43,44)47-10-14-19(50-52(40,41)42)18(36)24(49-14)33-13-32-17-21(27)30-12-31-22(17)33/h12-14,18-20,24,36-37,39H,5-11H2,1-4H3,(H,28,34)(H,29,38)(H,43,44)(H,45,46)(H2,27,30,31)(H2,40,41,42)/t14-,18-,19-,20?,24-/m1/s1 || InChI=1S/C26H44N7O18P3S/c1-25(2,20(37)23(38)29-6-5-15(34)28-7-8-55-16(35)9-26(3,4)39)11-48-54(45,46)51-53(43,44)47-10-14-19(50-52(40,41)42)18(36)24(49-14)33-13-32-17-21(27)30-12-31-22(17)33/h12-14,18-20,24,36-37,39H,5-11H2,1-4H3,(H,28,34)(H,29,38)(H,43,44)(H,45,46)(H2,27,30,31)(H2,40,41,42)/t14-,18-,19-,20+,24-/m1/s1</p>**

**</body>**

**</notes>**

**<annotation>**

**<rdf:RDF xmlns:rdf="http://www.w3.org/1999/02/22-rdf-syntax-ns#" xmlns:bqmodel="http://biomodels.net/model-qualifiers/" xmlns:bqbiol="http://biomodels.net/biology-qualifiers/">**

**<rdf:Description rdf:about="cf442f05-8998-4d2b-9a91-85241368c6fd">**

**<bqbiol:is>**

**<rdf:Bag>**

**<rdf:li rdf:resource="http://identifiers.org/PUBCHEM.COMPOUND/11953876"/></rdf:Bag></bqbiol:is><bqbiol:is>**

**<rdf:Bag>**

**<rdf:li rdf:resource="http://identifiers.org/PUBCHEM.COMPOUND/11966188"/></rdf:Bag></bqbiol:is><bqbiol:is>**

**<rdf:Bag>**

**<rdf:li rdf:resource="http://identifiers.org/KEGG.COMPOUND/C05998"/></rdf:Bag></bqbiol:is><bqbiol:is>**

**<rdf:Bag>**

**<rdf:li rdf:resource="http://identifiers.org/HMDB/HMDB06870"/></rdf:Bag></bqbiol:is><bqbiol:is>**

**<rdf:Bag>**

**<rdf:li rdf:resource="http://identifiers.org/CHEBI/CHEBI:28291"/></rdf:Bag></bqbiol:is><bqbiol:is>**

**<rdf:Bag>**

**<rdf:li rdf:resource="http://identifiers.org/INCHI/InChI=1S/C26H44N7O18P3S/c1-25(2,20(37)23(38)29-6-5-15(34)28-7-8-55-16(35)9-26(3,4)39)11-48-54(45,46)51-53(43,44)47-10-14-19(50-52(40,41)42)18(36)24(49-14)33-13-32-17-21(27)30-12-31-22(17)33/h12-14,18-20,24,36-37,39H,5-11H2,1-4H3,(H,28,34)(H,29,38)(H,43,44)(H,45,46)(H2,27,30,31)(H2,40,41,42)/t14-,18-,19-,20?,24-/m1/s1"/></rdf:Bag></bqbiol:is><bqbiol:is>**

**<rdf:Bag>**

**<rdf:li rdf:resource="http://identifiers.org/INCHI/InChI=1S/C26H44N7O18P3S/c1-25(2,20(37)23(38)29-6-5-15(34)28-7-8-55-16(35)9-26(3,4)39)11-48-54(45,46)51-53(43,44)47-10-14-19(50-52(40,41)42)18(36)24(49-14)33-13-32-17-21(27)30-12-31-22(17)33/h12-14,18-20,24,36-37,39H,5-11H2,1-4H3,(H,28,34)(H,29,38)(H,43,44)(H,45,46)(H2,27,30,31)(H2,40,41,42)/t14-,18-,19-,20+,24-/m1/s1"/></rdf:Bag></bqbiol:is></rdf:Description></rdf:RDF>**

**</annotation>**

**</species>**

**<species id="M_fad" constant="false" hasOnlySubstanceUnits="false" name="Flavin adenine dinucleotide oxidized" metaid="4fadf598-846a-4d29-8219-0185422a9e3a" boundaryCondition="false" compartment="metaComp">**

**<notes>**

**<body xmlns="http://www.w3.org/1999/xhtml">**

**<p>FORMULA: C27H31N9O15P2</p>**

**<p>CHARGE: 0</p>**

**<p>PUBCHEM.COMPOUND: 643975</p>**

**<p>KEGG.COMPOUND: C00016</p>**

**<p>HMDB: HMDB01248</p>**

**<p>CHEBI: CHEBI:16238</p>**

**<p>INCHI: InChI=1S/C27H33N9O15P2/c1-10-3-12-13(4-11(10)2)35(24-18(32-12)25(42)34-27(43)33-24)5-14(37)19(39)15(38)6-48-52(44,45)51-53(46,47)49-7-16-20(40)21(41)26(50-16)36-9-31-17-22(28)29-8-30-23(17)36/h3-4,8-9,14-16,19-21,26,37-41H,5-7H2,1-2H3,(H,44,45)(H,46,47)(H2,28,29,30)(H,34,42,43)/t14-,15+,16+,19-,20+,21+,26+/m0/s1</p>**

**</body>**

**</notes>**

**<annotation>**

**<rdf:RDF xmlns:rdf="http://www.w3.org/1999/02/22-rdf-syntax-ns#" xmlns:bqmodel="http://biomodels.net/model-qualifiers/" xmlns:bqbiol="http://biomodels.net/biology-qualifiers/">**

**<rdf:Description rdf:about="_4fadf598-846a-4d29-8219-0185422a9e3a">**

**<bqbiol:is>**

**<rdf:Bag>**

**<rdf:li rdf:resource="http://identifiers.org/PUBCHEM.COMPOUND/643975"/></rdf:Bag></bqbiol:is><bqbiol:is>**

**<rdf:Bag>**

**<rdf:li rdf:resource="http://identifiers.org/KEGG.COMPOUND/C00016"/></rdf:Bag></bqbiol:is><bqbiol:is>**

**<rdf:Bag>**

**<rdf:li rdf:resource="http://identifiers.org/HMDB/HMDB01248"/></rdf:Bag></bqbiol:is><bqbiol:is>**

**<rdf:Bag>**

**<rdf:li rdf:resource="http://identifiers.org/CHEBI/CHEBI:16238"/></rdf:Bag></bqbiol:is><bqbiol:is>**

**<rdf:Bag>**

**<rdf:li rdf:resource="http://identifiers.org/INCHI/InChI=1S/C27H33N9O15P2/c1-10-3-12-13(4-11(10)2)35(24-18(32-12)25(42)34-27(43)33-24)5-14(37)19(39)15(38)6-48-52(44,45)51-53(46,47)49-7-16-20(40)21(41)26(50-16)36-9-31-17-22(28)29-8-30-23(17)36/h3-4,8-9,14-16,19-21,26,37-41H,5-7H2,1-2H3,(H,44,45)(H,46,47)(H2,28,29,30)(H,34,42,43)/t14-,15+,16+,19-,20+,21+,26+/m0/s1"/></rdf:Bag></bqbiol:is></rdf:Description></rdf:RDF>**

**</annotation>**

**</species>**

**<species id="M_pydx" constant="false" hasOnlySubstanceUnits="false" name="Pyridoxal" metaid="64da0c1b-3aa4-43ca-b272-54193f7972e7" boundaryCondition="false" compartment="metaComp">**

**<notes>**

**<body xmlns="http://www.w3.org/1999/xhtml">**

**<p>FORMULA: C8H9NO3</p>**

**<p>CHARGE: 0</p>**

**<p>PUBCHEM.COMPOUND: 1050</p>**

**<p>INCHIKEY: RADKZDMFGJYCBB-UHFFFAOYSA-N</p>**

**<p>KEGG.COMPOUND: C00250</p>**

**<p>HMDB: HMDB01545</p>**

**<p>CHEBI: CHEBI:17310</p>**

**<p>INCHI: InChI=1S/C8H9NO3/c1-5-8(12)7(4-11)6(3-10)2-9-5/h2,4,10,12H,3H2,1H3</p>**

**</body>**

**</notes>**

**<annotation>**

**<rdf:RDF xmlns:rdf="http://www.w3.org/1999/02/22-rdf-syntax-ns#" xmlns:bqmodel="http://biomodels.net/model-qualifiers/" xmlns:bqbiol="http://biomodels.net/biology-qualifiers/">**

**<rdf:Description rdf:about="_64da0c1b-3aa4-43ca-b272-54193f7972e7">**

**<bqbiol:is>**

**<rdf:Bag>**

**<rdf:li rdf:resource="http://identifiers.org/PUBCHEM.COMPOUND/1050"/></rdf:Bag></bqbiol:is><bqbiol:is>**

**<rdf:Bag>**

**<rdf:li rdf:resource="http://identifiers.org/INCHIKEY/RADKZDMFGJYCBB-UHFFFAOYSA-N"/></rdf:Bag></bqbiol:is><bqbiol:is>**

**<rdf:Bag>**

**<rdf:li rdf:resource="http://identifiers.org/KEGG.COMPOUND/C00250"/></rdf:Bag></bqbiol:is><bqbiol:is>**

**<rdf:Bag>**

**<rdf:li rdf:resource="http://identifiers.org/HMDB/HMDB01545"/></rdf:Bag></bqbiol:is><bqbiol:is>**

**<rdf:Bag>**

**<rdf:li rdf:resource="http://identifiers.org/CHEBI/CHEBI:17310"/></rdf:Bag></bqbiol:is><bqbiol:is>**

**<rdf:Bag>**

**<rdf:li rdf:resource="http://identifiers.org/INCHI/InChI=1S/C8H9NO3/c1-5-8(12)7(4-11)6(3-10)2-9-5/h2,4,10,12H,3H2,1H3"/></rdf:Bag></bqbiol:is></rdf:Description></rdf:RDF>**

**</annotation>**

**</species>**

**<species id="M_HC00682" constant="false" hasOnlySubstanceUnits="false" name="S-Acetyldihydrolipoamide" metaid="7a409af6-1c7b-4202-8c85-4f107772f128" boundaryCondition="false" compartment="metaComp">**

**<notes>**

**<body xmlns="http://www.w3.org/1999/xhtml">**

**<p>FORMULA: C10H19NO2S2</p>**

**<p>CHARGE: 0</p>**

**<p>PUBCHEM.COMPOUND: 1076</p>**

**<p>INCHIKEY: ARGXEXVCHMNAQU-UHFFFAOYSA-N</p>**

**<p>KEGG.COMPOUND: C01136</p>**

**<p>HMDB: HMDB01526</p>**

**<p>CHEBI: CHEBI:16807</p>**

**<p>INCHI: InChI=1S/C10H19NO2S2/c1-8(12)15-9(6-7-14)4-2-3-5-10(11)13/h9,14H,2-7H2,1H3,(H2,11,13)</p>**

**</body>**

**</notes>**

**<annotation>**

**<rdf:RDF xmlns:rdf="http://www.w3.org/1999/02/22-rdf-syntax-ns#" xmlns:bqmodel="http://biomodels.net/model-qualifiers/" xmlns:bqbiol="http://biomodels.net/biology-qualifiers/">**

**<rdf:Description rdf:about="_7a409af6-1c7b-4202-8c85-4f107772f128">**

**<bqbiol:is>**

**<rdf:Bag>**

**<rdf:li rdf:resource="http://identifiers.org/PUBCHEM.COMPOUND/1076"/></rdf:Bag></bqbiol:is><bqbiol:is>**

**<rdf:Bag>**

**<rdf:li rdf:resource="http://identifiers.org/INCHIKEY/ARGXEXVCHMNAQU-UHFFFAOYSA-N"/></rdf:Bag></bqbiol:is><bqbiol:is>**

**<rdf:Bag>**

**<rdf:li rdf:resource="http://identifiers.org/KEGG.COMPOUND/C01136"/></rdf:Bag></bqbiol:is><bqbiol:is>**

**<rdf:Bag>**

**<rdf:li rdf:resource="http://identifiers.org/HMDB/HMDB01526"/></rdf:Bag></bqbiol:is><bqbiol:is>**

**<rdf:Bag>**

**<rdf:li rdf:resource="http://identifiers.org/CHEBI/CHEBI:16807"/></rdf:Bag></bqbiol:is><bqbiol:is>**

**<rdf:Bag>**

**<rdf:li rdf:resource="http://identifiers.org/INCHI/InChI=1S/C10H19NO2S2/c1-8(12)15-9(6-7-14)4-2-3-5-10(11)13/h9,14H,2-7H2,1H3,(H2,11,13)"/></rdf:Bag></bqbiol:is></rdf:Description></rdf:RDF>**

**</annotation>**

**</species>**

**<species id="M_thbpt" constant="false" hasOnlySubstanceUnits="false" name="5,6,7,8-tetrahydrobiopterin" metaid="6b19e573-278d-40d1-9f94-6fd3e2b26033" boundaryCondition="false" compartment="metaComp">**

**<notes>**

**<body xmlns="http://www.w3.org/1999/xhtml">**

**<p>FORMULA: C9H15N5O3</p>**

**<p>CHARGE: 0</p>**

**<p>PUBCHEM.COMPOUND: 44257 || 1125 || 169715</p>**

**<p>INCHIKEY: FNKQXYHWGSIFBK-UHFFFAOYSA-N</p>**

**<p>HMDB: HMDB00027</p>**

**<p>KEGG.COMPOUND: C00272</p>**

**<p>CHEBI: CHEBI:59560 || CHEBI:15372</p>**

**<p>INCHI: InChI=1S/C9H15N5O3/c1-3(15)6(16)4-2-11-7-5(12-4)8(17)14-9(10)13-7/h3-4,6,12,15-16H,2H2,1H3,(H4,10,11,13,14,17)</p>**

**</body>**

**</notes>**

**<annotation>**

**<rdf:RDF xmlns:rdf="http://www.w3.org/1999/02/22-rdf-syntax-ns#" xmlns:bqmodel="http://biomodels.net/model-qualifiers/" xmlns:bqbiol="http://biomodels.net/biology-qualifiers/">**

**<rdf:Description rdf:about="_6b19e573-278d-40d1-9f94-6fd3e2b26033">**

**<bqbiol:is>**

**<rdf:Bag>**

**<rdf:li rdf:resource="http://identifiers.org/PUBCHEM.COMPOUND/44257"/></rdf:Bag></bqbiol:is><bqbiol:is>**

**<rdf:Bag>**

**<rdf:li rdf:resource="http://identifiers.org/PUBCHEM.COMPOUND/1125"/></rdf:Bag></bqbiol:is><bqbiol:is>**

**<rdf:Bag>**

**<rdf:li rdf:resource="http://identifiers.org/PUBCHEM.COMPOUND/169715"/></rdf:Bag></bqbiol:is><bqbiol:is>**

**<rdf:Bag>**

**<rdf:li rdf:resource="http://identifiers.org/INCHIKEY/FNKQXYHWGSIFBK-UHFFFAOYSA-N"/></rdf:Bag></bqbiol:is><bqbiol:is>**

**<rdf:Bag>**

**<rdf:li rdf:resource="http://identifiers.org/HMDB/HMDB00027"/></rdf:Bag></bqbiol:is><bqbiol:is>**

**<rdf:Bag>**

**<rdf:li rdf:resource="http://identifiers.org/KEGG.COMPOUND/C00272"/></rdf:Bag></bqbiol:is><bqbiol:is>**

**<rdf:Bag>**

**<rdf:li rdf:resource="http://identifiers.org/CHEBI/CHEBI:59560"/></rdf:Bag></bqbiol:is><bqbiol:is>**

**<rdf:Bag>**

**<rdf:li rdf:resource="http://identifiers.org/CHEBI/CHEBI:15372"/></rdf:Bag></bqbiol:is><bqbiol:is>**

**<rdf:Bag>**

**<rdf:li rdf:resource="http://identifiers.org/INCHI/InChI=1S/C9H15N5O3/c1-3(15)6(16)4-2-11-7-5(12-4)8(17)14-9(10)13-7/h3-4,6,12,15-16H,2H2,1H3,(H4,10,11,13,14,17)"/></rdf:Bag></bqbiol:is></rdf:Description></rdf:RDF>**

**</annotation>**

**</species>**

**<species id="M_cs_pre" constant="false" hasOnlySubstanceUnits="false" name="chondroitin sulfate precursor (GalNAc-GlcA-(Gal)2-Xyl-L-Ser (protein))" metaid="a813fb38-59bb-4e8b-8052-a6ff90339485" boundaryCondition="false" compartment="metaComp">**

**<notes>**

**<body xmlns="http://www.w3.org/1999/xhtml">**

**<p>FORMULA: C31H49NO25X</p>**

**<p>CHARGE: 0</p>**

**<p>KEGG.COMPOUND: C04893</p>**

**</body>**

**</notes>**

**<annotation>**

**<rdf:RDF xmlns:rdf="http://www.w3.org/1999/02/22-rdf-syntax-ns#" xmlns:bqmodel="http://biomodels.net/model-qualifiers/" xmlns:bqbiol="http://biomodels.net/biology-qualifiers/">**

**<rdf:Description rdf:about="a813fb38-59bb-4e8b-8052-a6ff90339485">**

**<bqbiol:is>**

**<rdf:Bag>**

**<rdf:li rdf:resource="http://identifiers.org/KEGG.COMPOUND/C04893"/></rdf:Bag></bqbiol:is></rdf:Description></rdf:RDF>**

**</annotation>**

**</species>**

**<species id="M_mi14p" constant="false" hasOnlySubstanceUnits="false" name="(1R,2R,3R,4R,5R,6S)-2,3,5,6-tetrahydroxy-4-(phosphonatooxy)cyclohexyl phosphate" metaid="bc4150c6-b49b-4f07-b774-8bcc49225be9" boundaryCondition="false" compartment="metaComp">**

**<notes>**

**<body xmlns="http://www.w3.org/1999/xhtml">**

**<p>FORMULA: C6H10O12P2</p>**

**<p>CHARGE: 0</p>**

**<p>KEGG.COMPOUND: C01220</p>**

**<p>HMDB: HMDB00968</p>**

**<p>CHEBI: CHEBI:17816 || CHEBI:58282</p>**

**<p>INCHI: InChI=1S/C6H14O12P2/c7-1-2(8)6(18-20(14,15)16)4(10)3(9)5(1)17-19(11,12)13/h1-10H,(H2,11,12,13)(H2,14,15,16)/t1-,2-,3-,4+,5+,6+/m1/s1 || InChI=1S/C6H14O12P2/c7-1-2(8)6(18-20(14,15)16)4(10)3(9)5(1)17-19(11,12)13/h1-10H,(H2,11,12,13)(H2,14,15,16)/p-4/t1-,2-,3-,4+,5+,6+/m1/s1</p>**

**</body>**

**</notes>**

**<annotation>**

**<rdf:RDF xmlns:rdf="http://www.w3.org/1999/02/22-rdf-syntax-ns#" xmlns:bqmodel="http://biomodels.net/model-qualifiers/" xmlns:bqbiol="http://biomodels.net/biology-qualifiers/">**

**<rdf:Description rdf:about="bc4150c6-b49b-4f07-b774-8bcc49225be9">**

**<bqbiol:is>**

**<rdf:Bag>**

**<rdf:li rdf:resource="http://identifiers.org/KEGG.COMPOUND/C01220"/></rdf:Bag></bqbiol:is><bqbiol:is>**

**<rdf:Bag>**

**<rdf:li rdf:resource="http://identifiers.org/HMDB/HMDB00968"/></rdf:Bag></bqbiol:is><bqbiol:is>**

**<rdf:Bag>**

**<rdf:li rdf:resource="http://identifiers.org/CHEBI/CHEBI:17816"/></rdf:Bag></bqbiol:is><bqbiol:is>**

**<rdf:Bag>**

**<rdf:li rdf:resource="http://identifiers.org/CHEBI/CHEBI:58282"/></rdf:Bag></bqbiol:is><bqbiol:is>**

**<rdf:Bag>**

**<rdf:li rdf:resource="http://identifiers.org/INCHI/InChI=1S/C6H14O12P2/c7-1-2(8)6(18-20(14,15)16)4(10)3(9)5(1)17-19(11,12)13/h1-10H,(H2,11,12,13)(H2,14,15,16)/t1-,2-,3-,4+,5+,6+/m1/s1"/></rdf:Bag></bqbiol:is><bqbiol:is>**

**<rdf:Bag>**

**<rdf:li rdf:resource="http://identifiers.org/INCHI/InChI=1S/C6H14O12P2/c7-1-2(8)6(18-20(14,15)16)4(10)3(9)5(1)17-19(11,12)13/h1-10H,(H2,11,12,13)(H2,14,15,16)/p-4/t1-,2-,3-,4+,5+,6+/m1/s1"/></rdf:Bag></bqbiol:is></rdf:Description></rdf:RDF>**

**</annotation>**

**</species>**

**<species id="M_HC01797" constant="false" hasOnlySubstanceUnits="false" name="Activated_methyl_group" metaid="42c69b53-80bd-4360-8572-5487215d56a7" boundaryCondition="false" compartment="metaComp">**

**<notes>**

**<body xmlns="http://www.w3.org/1999/xhtml">**

**<p>CHARGE: 0</p>**

**<p>PUBCHEM.COMPOUND: 13088</p>**

**<p>KEGG.COMPOUND: C10905</p>**

**</body>**

**</notes>**

**<annotation>**

**<rdf:RDF xmlns:rdf="http://www.w3.org/1999/02/22-rdf-syntax-ns#" xmlns:bqmodel="http://biomodels.net/model-qualifiers/" xmlns:bqbiol="http://biomodels.net/biology-qualifiers/">**

**<rdf:Description rdf:about="_42c69b53-80bd-4360-8572-5487215d56a7">**

**<bqbiol:is>**

**<rdf:Bag>**

**<rdf:li rdf:resource="http://identifiers.org/PUBCHEM.COMPOUND/13088"/></rdf:Bag></bqbiol:is><bqbiol:is>**

**<rdf:Bag>**

**<rdf:li rdf:resource="http://identifiers.org/KEGG.COMPOUND/C10905"/></rdf:Bag></bqbiol:is></rdf:Description></rdf:RDF>**

**</annotation>**

**</species>**

**<species id="M_ebastine" constant="false" hasOnlySubstanceUnits="false" name="ebastine" metaid="1936b890-caa3-41a7-ae69-40924af4e658" boundaryCondition="false" compartment="metaComp">**

**<notes>**

**<body xmlns="http://www.w3.org/1999/xhtml">**

**<p>FORMULA: C32H39NO2</p>**

**<p>CHARGE: 0</p>**

**<p>PUBCHEM.COMPOUND: 3191</p>**

**<p>INCHIKEY: MJJALKDDGIKVBE-UHFFFAOYSA-N</p>**

**<p>CHEBI: CHEBI:211060 || CHEBI:31528</p>**

**<p>INCHI: InChI=1S/C32H39NO2/c1-32(2,3)28-18-16-25(17-19-28)30(34)15-10-22-33-23-20-29(21-24-33)35-31(26-11-6-4-7-12-26)27-13-8-5-9-14-27/h4-9,11-14,16-19,29,31H,10,15,20-24H2,1-3H3</p>**

**</body>**

**</notes>**

**<annotation>**

**<rdf:RDF xmlns:rdf="http://www.w3.org/1999/02/22-rdf-syntax-ns#" xmlns:bqmodel="http://biomodels.net/model-qualifiers/" xmlns:bqbiol="http://biomodels.net/biology-qualifiers/">**

**<rdf:Description rdf:about="_1936b890-caa3-41a7-ae69-40924af4e658">**

**<bqbiol:is>**

**<rdf:Bag>**

**<rdf:li rdf:resource="http://identifiers.org/PUBCHEM.COMPOUND/3191"/></rdf:Bag></bqbiol:is><bqbiol:is>**

**<rdf:Bag>**

**<rdf:li rdf:resource="http://identifiers.org/INCHIKEY/MJJALKDDGIKVBE-UHFFFAOYSA-N"/></rdf:Bag></bqbiol:is><bqbiol:is>**

**<rdf:Bag>**

**<rdf:li rdf:resource="http://identifiers.org/CHEBI/CHEBI:211060"/></rdf:Bag></bqbiol:is><bqbiol:is>**

**<rdf:Bag>**

**<rdf:li rdf:resource="http://identifiers.org/CHEBI/CHEBI:31528"/></rdf:Bag></bqbiol:is><bqbiol:is>**

**<rdf:Bag>**

**<rdf:li rdf:resource="http://identifiers.org/INCHI/InChI=1S/C32H39NO2/c1-32(2,3)28-18-16-25(17-19-28)30(34)15-10-22-33-23-20-29(21-24-33)35-31(26-11-6-4-7-12-26)27-13-8-5-9-14-27/h4-9,11-14,16-19,29,31H,10,15,20-24H2,1-3H3"/></rdf:Bag></bqbiol:is></rdf:Description></rdf:RDF>**

**</annotation>**

**</species>**

**<species id="M_gchola" constant="false" hasOnlySubstanceUnits="false" name="glycocholate" metaid="f4894ef3-6308-4b82-9f47-0e0d1e7c45b5" boundaryCondition="false" compartment="metaComp">**

**<notes>**

**<body xmlns="http://www.w3.org/1999/xhtml">**

**<p>FORMULA: C26H43NO6</p>**

**<p>CHARGE: 0</p>**

**<p>PUBCHEM.COMPOUND: 10140 || 23617285</p>**

**<p>INCHIKEY: RFDAIACWWDREDC-FRVQLJSFSA-N</p>**

**<p>KEGG.COMPOUND: C01921</p>**

**<p>HMDB: HMDB00138</p>**

**<p>CHEBI: CHEBI:17687 || CHEBI:29746</p>**

**<p>INCHI: InChI=1S/C26H43NO6/c1-14(4-7-22(31)27-13-23(32)33)17-5-6-18-24-19(12-21(30)26(17,18)3)25(2)9-8-16(28)10-15(25)11-20(24)29/h14-21,24,28-30H,4-13H2,1-3H3,(H,27,31)(H,32,33)/t14-,15+,16-,17-,18+,19+,20-,21+,24+,25+,26-/m1/s1 || InChI=1S/C26H43NO6/c1-14(4-7-22(31)27-13-23(32)33)17-5-6-18-24-19(12-21(30)26(17,18)3)25(2)9-8-16(28)10-15(25)11-20(24)29/h14-21,24,28-30H,4-13H2,1-3H3,(H,27,31)(H,32,33)/t14-,15?,16-,17-,18+,19+,20-,21+,24+,25+,26-/m1/s1</p>**

**</body>**

**</notes>**

**<annotation>**

**<rdf:RDF xmlns:rdf="http://www.w3.org/1999/02/22-rdf-syntax-ns#" xmlns:bqmodel="http://biomodels.net/model-qualifiers/" xmlns:bqbiol="http://biomodels.net/biology-qualifiers/">**

**<rdf:Description rdf:about="f4894ef3-6308-4b82-9f47-0e0d1e7c45b5">**

**<bqbiol:is>**

**<rdf:Bag>**

**<rdf:li rdf:resource="http://identifiers.org/PUBCHEM.COMPOUND/10140"/></rdf:Bag></bqbiol:is><bqbiol:is>**

**<rdf:Bag>**

**<rdf:li rdf:resource="http://identifiers.org/PUBCHEM.COMPOUND/23617285"/></rdf:Bag></bqbiol:is><bqbiol:is>**

**<rdf:Bag>**

**<rdf:li rdf:resource="http://identifiers.org/INCHIKEY/RFDAIACWWDREDC-FRVQLJSFSA-N"/></rdf:Bag></bqbiol:is><bqbiol:is>**

**<rdf:Bag>**

**<rdf:li rdf:resource="http://identifiers.org/KEGG.COMPOUND/C01921"/></rdf:Bag></bqbiol:is><bqbiol:is>**

**<rdf:Bag>**

**<rdf:li rdf:resource="http://identifiers.org/HMDB/HMDB00138"/></rdf:Bag></bqbiol:is><bqbiol:is>**

**<rdf:Bag>**

**<rdf:li rdf:resource="http://identifiers.org/CHEBI/CHEBI:17687"/></rdf:Bag></bqbiol:is><bqbiol:is>**

**<rdf:Bag>**

**<rdf:li rdf:resource="http://identifiers.org/CHEBI/CHEBI:29746"/></rdf:Bag></bqbiol:is><bqbiol:is>**

**<rdf:Bag>**

**<rdf:li rdf:resource="http://identifiers.org/INCHI/InChI=1S/C26H43NO6/c1-14(4-7-22(31)27-13-23(32)33)17-5-6-18-24-19(12-21(30)26(17,18)3)25(2)9-8-16(28)10-15(25)11-20(24)29/h14-21,24,28-30H,4-13H2,1-3H3,(H,27,31)(H,32,33)/t14-,15+,16-,17-,18+,19+,20-,21+,24+,25+,26-/m1/s1"/></rdf:Bag></bqbiol:is><bqbiol:is>**

**<rdf:Bag>**

**<rdf:li rdf:resource="http://identifiers.org/INCHI/InChI=1S/C26H43NO6/c1-14(4-7-22(31)27-13-23(32)33)17-5-6-18-24-19(12-21(30)26(17,18)3)25(2)9-8-16(28)10-15(25)11-20(24)29/h14-21,24,28-30H,4-13H2,1-3H3,(H,27,31)(H,32,33)/t14-,15?,16-,17-,18+,19+,20-,21+,24+,25+,26-/m1/s1"/></rdf:Bag></bqbiol:is></rdf:Description></rdf:RDF>**

**</annotation>**

**</species>**

**<species id="M_ksi_pre27" constant="false" hasOnlySubstanceUnits="false" name="keratan sulfate I biosynthesis, precursor 27" metaid="92bded93-c535-4ea4-8863-a09a0abea4bc" boundaryCondition="false" compartment="metaComp">**

**<notes>**

**<body xmlns="http://www.w3.org/1999/xhtml">**

**<p>FORMULA: C205H328N14O171S8X</p>**

**<p>CHARGE: 0</p>**

**</body>**

**</notes>**

**<annotation>**

**<rdf:RDF xmlns:rdf="http://www.w3.org/1999/02/22-rdf-syntax-ns#" xmlns:bqmodel="http://biomodels.net/model-qualifiers/" xmlns:bqbiol="http://biomodels.net/biology-qualifiers/">**

**<rdf:Description rdf:about="_92bded93-c535-4ea4-8863-a09a0abea4bc"/></rdf:RDF>**

**</annotation>**

**</species>**

**<species id="M_ksi_pre28" constant="false" hasOnlySubstanceUnits="false" name="keratan sulfate I biosynthesis, precursor 28" metaid="a959ce83-229c-4076-801f-0a847356adb4" boundaryCondition="false" compartment="metaComp">**

**<notes>**

**<body xmlns="http://www.w3.org/1999/xhtml">**

**<p>FORMULA: C213H341N15O176S8X</p>**

**<p>CHARGE: 0</p>**

**</body>**

**</notes>**

**<annotation>**

**<rdf:RDF xmlns:rdf="http://www.w3.org/1999/02/22-rdf-syntax-ns#" xmlns:bqmodel="http://biomodels.net/model-qualifiers/" xmlns:bqbiol="http://biomodels.net/biology-qualifiers/">**

**<rdf:Description rdf:about="a959ce83-229c-4076-801f-0a847356adb4"/></rdf:RDF>**

**</annotation>**

**</species>**

**<species id="M_HC01377" constant="false" hasOnlySubstanceUnits="false" name="S-(3-Methylbutanoyl)-dihydrolipoamide" metaid="0f9ab2fd-ce65-4944-b60a-06387c957cfd" boundaryCondition="false" compartment="metaComp">**

**<notes>**

**<body xmlns="http://www.w3.org/1999/xhtml">**

**<p>FORMULA: C13H25NO2S2</p>**

**<p>CHARGE: 0</p>**

**<p>PUBCHEM.COMPOUND: 440566</p>**

**<p>INCHIKEY: KMUSXGCRMMQDBP-UHFFFAOYSA-N</p>**

**<p>KEGG.COMPOUND: C05119</p>**

**<p>HMDB: HMDB06867</p>**

**<p>CHEBI: CHEBI:27462</p>**

**<p>INCHI: InChI=1S/C13H25NO2S2/c1-10(2)9-13(16)18-8-7-11(17)5-3-4-6-12(14)15/h10-11,17H,3-9H2,1-2H3,(H2,14,15)</p>**

**</body>**

**</notes>**

**<annotation>**

**<rdf:RDF xmlns:rdf="http://www.w3.org/1999/02/22-rdf-syntax-ns#" xmlns:bqmodel="http://biomodels.net/model-qualifiers/" xmlns:bqbiol="http://biomodels.net/biology-qualifiers/">**

**<rdf:Description rdf:about="_0f9ab2fd-ce65-4944-b60a-06387c957cfd">**

**<bqbiol:is>**

**<rdf:Bag>**

**<rdf:li rdf:resource="http://identifiers.org/PUBCHEM.COMPOUND/440566"/></rdf:Bag></bqbiol:is><bqbiol:is>**

**<rdf:Bag>**

**<rdf:li rdf:resource="http://identifiers.org/INCHIKEY/KMUSXGCRMMQDBP-UHFFFAOYSA-N"/></rdf:Bag></bqbiol:is><bqbiol:is>**

**<rdf:Bag>**

**<rdf:li rdf:resource="http://identifiers.org/KEGG.COMPOUND/C05119"/></rdf:Bag></bqbiol:is><bqbiol:is>**

**<rdf:Bag>**

**<rdf:li rdf:resource="http://identifiers.org/HMDB/HMDB06867"/></rdf:Bag></bqbiol:is><bqbiol:is>**

**<rdf:Bag>**

**<rdf:li rdf:resource="http://identifiers.org/CHEBI/CHEBI:27462"/></rdf:Bag></bqbiol:is><bqbiol:is>**

**<rdf:Bag>**

**<rdf:li rdf:resource="http://identifiers.org/INCHI/InChI=1S/C13H25NO2S2/c1-10(2)9-13(16)18-8-7-11(17)5-3-4-6-12(14)15/h10-11,17H,3-9H2,1-2H3,(H2,14,15)"/></rdf:Bag></bqbiol:is></rdf:Description></rdf:RDF>**

**</annotation>**

**</species>**

**<species id="M_ksi_pre29" constant="false" hasOnlySubstanceUnits="false" name="keratan sulfate I biosynthesis, precursor 29" metaid="e6baeb60-aedd-4ec6-abbb-bc6c8e9791a6" boundaryCondition="false" compartment="metaComp">**

**<notes>**

**<body xmlns="http://www.w3.org/1999/xhtml">**

**<p>FORMULA: C213H340N15O179S9X</p>**

**<p>CHARGE: 0</p>**

**</body>**

**</notes>**

**<annotation>**

**<rdf:RDF xmlns:rdf="http://www.w3.org/1999/02/22-rdf-syntax-ns#" xmlns:bqmodel="http://biomodels.net/model-qualifiers/" xmlns:bqbiol="http://biomodels.net/biology-qualifiers/">**

**<rdf:Description rdf:about="e6baeb60-aedd-4ec6-abbb-bc6c8e9791a6"/></rdf:RDF>**

**</annotation>**

**</species>**

**<species id="M_HC01376" constant="false" hasOnlySubstanceUnits="false" name="S-(2-Methylbutanoyl)-dihydrolipoamide" metaid="82332e0a-fa83-4223-ba59-6b13fa522693" boundaryCondition="false" compartment="metaComp">**

**<notes>**

**<body xmlns="http://www.w3.org/1999/xhtml">**

**<p>FORMULA: C13H25NO2S2</p>**

**<p>CHARGE: 0</p>**

**<p>PUBCHEM.COMPOUND: 440565</p>**

**<p>INCHIKEY: UFNCWFSSEGPJNL-UHFFFAOYSA-N</p>**

**<p>KEGG.COMPOUND: C05118</p>**

**<p>HMDB: HMDB06869</p>**

**<p>CHEBI: CHEBI:28692</p>**

**<p>INCHI: InChI=1S/C13H25NO2S2/c1-3-10(2)13(16)18-9-8-11(17)6-4-5-7-12(14)15/h10-11,17H,3-9H2,1-2H3,(H2,14,15)</p>**

**</body>**

**</notes>**

**<annotation>**

**<rdf:RDF xmlns:rdf="http://www.w3.org/1999/02/22-rdf-syntax-ns#" xmlns:bqmodel="http://biomodels.net/model-qualifiers/" xmlns:bqbiol="http://biomodels.net/biology-qualifiers/">**

**<rdf:Description rdf:about="_82332e0a-fa83-4223-ba59-6b13fa522693">**

**<bqbiol:is>**

**<rdf:Bag>**

**<rdf:li rdf:resource="http://identifiers.org/PUBCHEM.COMPOUND/440565"/></rdf:Bag></bqbiol:is><bqbiol:is>**

**<rdf:Bag>**

**<rdf:li rdf:resource="http://identifiers.org/INCHIKEY/UFNCWFSSEGPJNL-UHFFFAOYSA-N"/></rdf:Bag></bqbiol:is><bqbiol:is>**

**<rdf:Bag>**

**<rdf:li rdf:resource="http://identifiers.org/KEGG.COMPOUND/C05118"/></rdf:Bag></bqbiol:is><bqbiol:is>**

**<rdf:Bag>**

**<rdf:li rdf:resource="http://identifiers.org/HMDB/HMDB06869"/></rdf:Bag></bqbiol:is><bqbiol:is>**

**<rdf:Bag>**

**<rdf:li rdf:resource="http://identifiers.org/CHEBI/CHEBI:28692"/></rdf:Bag></bqbiol:is><bqbiol:is>**

**<rdf:Bag>**

**<rdf:li rdf:resource="http://identifiers.org/INCHI/InChI=1S/C13H25NO2S2/c1-3-10(2)13(16)18-9-8-11(17)6-4-5-7-12(14)15/h10-11,17H,3-9H2,1-2H3,(H2,14,15)"/></rdf:Bag></bqbiol:is></rdf:Description></rdf:RDF>**

**</annotation>**

**</species>**

**<species id="M_ksi_pre23" constant="false" hasOnlySubstanceUnits="false" name="keratan sulfate I biosynthesis, precursor 23" metaid="32828aca-97ef-4cbe-ae79-0da0d99973c2" boundaryCondition="false" compartment="metaComp">**

**<notes>**

**<body xmlns="http://www.w3.org/1999/xhtml">**

**<p>FORMULA: C185H296N13O153S7X</p>**

**<p>CHARGE: 0</p>**

**</body>**

**</notes>**

**<annotation>**

**<rdf:RDF xmlns:rdf="http://www.w3.org/1999/02/22-rdf-syntax-ns#" xmlns:bqmodel="http://biomodels.net/model-qualifiers/" xmlns:bqbiol="http://biomodels.net/biology-qualifiers/">**

**<rdf:Description rdf:about="_32828aca-97ef-4cbe-ae79-0da0d99973c2"/></rdf:RDF>**

**</annotation>**

**</species>**

**<species id="M_ksi_pre24" constant="false" hasOnlySubstanceUnits="false" name="keratan sulfate I biosynthesis, precursor 24" metaid="1eae1213-faba-4da8-9f4a-8742d1a3c26f" boundaryCondition="false" compartment="metaComp">**

**<notes>**

**<body xmlns="http://www.w3.org/1999/xhtml">**

**<p>FORMULA: C191H306N13O158S7X</p>**

**<p>CHARGE: 0</p>**

**</body>**

**</notes>**

**<annotation>**

**<rdf:RDF xmlns:rdf="http://www.w3.org/1999/02/22-rdf-syntax-ns#" xmlns:bqmodel="http://biomodels.net/model-qualifiers/" xmlns:bqbiol="http://biomodels.net/biology-qualifiers/">**

**<rdf:Description rdf:about="_1eae1213-faba-4da8-9f4a-8742d1a3c26f"/></rdf:RDF>**

**</annotation>**

**</species>**

**<species id="M_ksi_pre25" constant="false" hasOnlySubstanceUnits="false" name="keratan sulfate I biosynthesis, precursor 25" metaid="760489ae-f9a2-4485-8171-64af9cffcba3" boundaryCondition="false" compartment="metaComp">**

**<notes>**

**<body xmlns="http://www.w3.org/1999/xhtml">**

**<p>FORMULA: C199H319N14O163S7X</p>**

**<p>CHARGE: 0</p>**

**</body>**

**</notes>**

**<annotation>**

**<rdf:RDF xmlns:rdf="http://www.w3.org/1999/02/22-rdf-syntax-ns#" xmlns:bqmodel="http://biomodels.net/model-qualifiers/" xmlns:bqbiol="http://biomodels.net/biology-qualifiers/">**

**<rdf:Description rdf:about="_760489ae-f9a2-4485-8171-64af9cffcba3"/></rdf:RDF>**

**</annotation>**

**</species>**

**<species id="M_mi1345p" constant="false" hasOnlySubstanceUnits="false" name="1D-myo-inositol 1,3,4,5-tetrakisphosphate(8-)" metaid="25f9fb14-97ae-4cf9-8846-943a5947d226" boundaryCondition="false" compartment="metaComp">**

**<notes>**

**<body xmlns="http://www.w3.org/1999/xhtml">**

**<p>FORMULA: C6H8O18P4</p>**

**<p>CHARGE: 0</p>**

**<p>PUBCHEM.COMPOUND: 107758</p>**

**<p>KEGG.COMPOUND: C01272</p>**

**<p>HMDB: HMDB01059</p>**

**<p>CHEBI: CHEBI:16783 || CHEBI:57895</p>**

**<p>INCHI: InChI=1S/C6H16O18P4/c7-1-3(21-25(9,10)11)2(8)5(23-27(15,16)17)6(24-28(18,19)20)4(1)22-26(12,13)14/h1-8H,(H2,9,10,11)(H2,12,13,14)(H2,15,16,17)(H2,18,19,20)/t1-,2-,3?,4-,5+,6?/m0/s1 || InChI=1S/C6H16O18P4/c7-1-3(21-25(9,10)11)2(8)5(23-27(15,16)17)6(24-28(18,19)20)4(1)22-26(12,13)14/h1-8H,(H2,9,10,11)(H2,12,13,14)(H2,15,16,17)(H2,18,19,20)/t1-,2-,3-,4+,5-,6-/m0/s1 || InChI=1S/C6H16O18P4/c7-1-3(21-25(9,10)11)2(8)5(23-27(15,16)17)6(24-28(18,19)20)4(1)22-26(12,13)14/h1-8H,(H2,9,10,11)(H2,12,13,14)(H2,15,16,17)(H2,18,19,20)/p-8/t1-,2-,3-,4+,5-,6-/m0/s1</p>**

**</body>**

**</notes>**

**<annotation>**

**<rdf:RDF xmlns:rdf="http://www.w3.org/1999/02/22-rdf-syntax-ns#" xmlns:bqmodel="http://biomodels.net/model-qualifiers/" xmlns:bqbiol="http://biomodels.net/biology-qualifiers/">**

**<rdf:Description rdf:about="_25f9fb14-97ae-4cf9-8846-943a5947d226">**

**<bqbiol:is>**

**<rdf:Bag>**

**<rdf:li rdf:resource="http://identifiers.org/PUBCHEM.COMPOUND/107758"/></rdf:Bag></bqbiol:is><bqbiol:is>**

**<rdf:Bag>**

**<rdf:li rdf:resource="http://identifiers.org/KEGG.COMPOUND/C01272"/></rdf:Bag></bqbiol:is><bqbiol:is>**

**<rdf:Bag>**

**<rdf:li rdf:resource="http://identifiers.org/HMDB/HMDB01059"/></rdf:Bag></bqbiol:is><bqbiol:is>**

**<rdf:Bag>**

**<rdf:li rdf:resource="http://identifiers.org/CHEBI/CHEBI:16783"/></rdf:Bag></bqbiol:is><bqbiol:is>**

**<rdf:Bag>**

**<rdf:li rdf:resource="http://identifiers.org/CHEBI/CHEBI:57895"/></rdf:Bag></bqbiol:is><bqbiol:is>**

**<rdf:Bag>**

**<rdf:li rdf:resource="http://identifiers.org/INCHI/InChI=1S/C6H16O18P4/c7-1-3(21-25(9,10)11)2(8)5(23-27(15,16)17)6(24-28(18,19)20)4(1)22-26(12,13)14/h1-8H,(H2,9,10,11)(H2,12,13,14)(H2,15,16,17)(H2,18,19,20)/t1-,2-,3?,4-,5+,6?/m0/s1"/></rdf:Bag></bqbiol:is><bqbiol:is>**

**<rdf:Bag>**

**<rdf:li rdf:resource="http://identifiers.org/INCHI/InChI=1S/C6H16O18P4/c7-1-3(21-25(9,10)11)2(8)5(23-27(15,16)17)6(24-28(18,19)20)4(1)22-26(12,13)14/h1-8H,(H2,9,10,11)(H2,12,13,14)(H2,15,16,17)(H2,18,19,20)/t1-,2-,3-,4+,5-,6-/m0/s1"/></rdf:Bag></bqbiol:is><bqbiol:is>**

**<rdf:Bag>**

**<rdf:li rdf:resource="http://identifiers.org/INCHI/InChI=1S/C6H16O18P4/c7-1-3(21-25(9,10)11)2(8)5(23-27(15,16)17)6(24-28(18,19)20)4(1)22-26(12,13)14/h1-8H,(H2,9,10,11)(H2,12,13,14)(H2,15,16,17)(H2,18,19,20)/p-8/t1-,2-,3-,4+,5-,6-/m0/s1"/></rdf:Bag></bqbiol:is></rdf:Description></rdf:RDF>**

**</annotation>**

**</species>**

**<species id="M_ksi_pre26" constant="false" hasOnlySubstanceUnits="false" name="keratan sulfate I biosynthesis, precursor 26" metaid="f0386ac0-44ef-49cc-8158-63bad6e172d4" boundaryCondition="false" compartment="metaComp">**

**<notes>**

**<body xmlns="http://www.w3.org/1999/xhtml">**

**<p>FORMULA: C199H318N14O166S8X</p>**

**<p>CHARGE: 0</p>**

**</body>**

**</notes>**

**<annotation>**

**<rdf:RDF xmlns:rdf="http://www.w3.org/1999/02/22-rdf-syntax-ns#" xmlns:bqmodel="http://biomodels.net/model-qualifiers/" xmlns:bqbiol="http://biomodels.net/biology-qualifiers/">**

**<rdf:Description rdf:about="f0386ac0-44ef-49cc-8158-63bad6e172d4"/></rdf:RDF>**

**</annotation>**

**</species>**

**<species id="M_5HPET" constant="false" hasOnlySubstanceUnits="false" name="5(S)-HPETE(1-)" metaid="cb295104-5e37-4aa3-9127-c248767caf19" boundaryCondition="false" compartment="metaComp">**

**<notes>**

**<body xmlns="http://www.w3.org/1999/xhtml">**

**<p>FORMULA: C20H31O4</p>**

**<p>CHARGE: 0</p>**

**<p>PUBCHEM.COMPOUND: 5280778</p>**

**<p>KEGG.COMPOUND: C05356</p>**

**<p>HMDB: HMDB01193 || HMDB11135</p>**

**<p>CHEBI: CHEBI:15632 || CHEBI:57450</p>**

**<p>INCHI: InChI=1S/C20H32O4/c1-2-3-4-5-6-7-8-9-10-11-12-13-14-16-19(24-23)17-15-18-20(21)22/h6-7,9-10,12-14,16,19,23H,2-5,8,11,15,17-18H2,1H3,(H,21,22)/b7-6-,10-9-,13-12-,16-14+/t19-/m1/s1 || InChI=1S/C20H32O4/c1-2-3-4-5-6-7-8-9-10-11-12-13-14-16-19(24-23)17-15-18-20(21)22/h6-7,9-10,12-14,16,19,23H,2-5,8,11,15,17-18H2,1H3,(H,21,22)/p-1/b7-6-,10-9-,13-12-,16-14+/t19-/m1/s1</p>**

**</body>**

**</notes>**

**<annotation>**

**<rdf:RDF xmlns:rdf="http://www.w3.org/1999/02/22-rdf-syntax-ns#" xmlns:bqmodel="http://biomodels.net/model-qualifiers/" xmlns:bqbiol="http://biomodels.net/biology-qualifiers/">**

**<rdf:Description rdf:about="cb295104-5e37-4aa3-9127-c248767caf19">**

**<bqbiol:is>**

**<rdf:Bag>**

**<rdf:li rdf:resource="http://identifiers.org/PUBCHEM.COMPOUND/5280778"/></rdf:Bag></bqbiol:is><bqbiol:is>**

**<rdf:Bag>**

**<rdf:li rdf:resource="http://identifiers.org/KEGG.COMPOUND/C05356"/></rdf:Bag></bqbiol:is><bqbiol:is>**

**<rdf:Bag>**

**<rdf:li rdf:resource="http://identifiers.org/HMDB/HMDB01193"/></rdf:Bag></bqbiol:is><bqbiol:is>**

**<rdf:Bag>**

**<rdf:li rdf:resource="http://identifiers.org/HMDB/HMDB11135"/></rdf:Bag></bqbiol:is><bqbiol:is>**

**<rdf:Bag>**

**<rdf:li rdf:resource="http://identifiers.org/CHEBI/CHEBI:15632"/></rdf:Bag></bqbiol:is><bqbiol:is>**

**<rdf:Bag>**

**<rdf:li rdf:resource="http://identifiers.org/CHEBI/CHEBI:57450"/></rdf:Bag></bqbiol:is><bqbiol:is>**

**<rdf:Bag>**

**<rdf:li rdf:resource="http://identifiers.org/INCHI/InChI=1S/C20H32O4/c1-2-3-4-5-6-7-8-9-10-11-12-13-14-16-19(24-23)17-15-18-20(21)22/h6-7,9-10,12-14,16,19,23H,2-5,8,11,15,17-18H2,1H3,(H,21,22)/b7-6-,10-9-,13-12-,16-14+/t19-/m1/s1"/></rdf:Bag></bqbiol:is><bqbiol:is>**

**<rdf:Bag>**

**<rdf:li rdf:resource="http://identifiers.org/INCHI/InChI=1S/C20H32O4/c1-2-3-4-5-6-7-8-9-10-11-12-13-14-16-19(24-23)17-15-18-20(21)22/h6-7,9-10,12-14,16,19,23H,2-5,8,11,15,17-18H2,1H3,(H,21,22)/p-1/b7-6-,10-9-,13-12-,16-14+/t19-/m1/s1"/></rdf:Bag></bqbiol:is></rdf:Description></rdf:RDF>**

**</annotation>**

**</species>**

**<species id="M_malt" constant="false" hasOnlySubstanceUnits="false" name="maltose" metaid="57b620d4-91b2-479b-93e8-70b0eb5e0780" boundaryCondition="false" compartment="metaComp">**

**<notes>**

**<body xmlns="http://www.w3.org/1999/xhtml">**

**<p>FORMULA: C12H22O11</p>**

**<p>CHARGE: 0</p>**

**<p>PUBCHEM.COMPOUND: 6255 || 45109807 || 10991489 || 294 || 439341 || 439186 || 303614 || 181526</p>**

**<p>INCHIKEY: GUBGYTABKSRVRQ-PICCSMPSSA-N</p>**

**<p>KEGG.COMPOUND: C01971 || C00897 || C00208</p>**

**<p>HMDB: HMDB00163</p>**

**<p>CHEBI: CHEBI:18167 || CHEBI:47937 || CHEBI:18147 || CHEBI:17306</p>**

**<p>INCHI: InChI=1S/C12H22O11/c13-1-3-5(15)6(16)9(19)12(22-3)23-10-4(2-14)21-11(20)8(18)7(10)17/h3-20H,1-2H2/t3-,4-,5-,6+,7-,8-,9-,10-,11?,12-/m1/s1 || InChI=1S/C12H22O11/c13-1-3-5(15)6(16)9(19)12(22-3)23-10-4(2-14)21-11(20)8(18)7(10)17/h3-20H,1-2H2/t3-,4-,5-,6+,7-,8+,9-,10-,11+,12-/m1/s1</p>**

**</body>**

**</notes>**

**<annotation>**

**<rdf:RDF xmlns:rdf="http://www.w3.org/1999/02/22-rdf-syntax-ns#" xmlns:bqmodel="http://biomodels.net/model-qualifiers/" xmlns:bqbiol="http://biomodels.net/biology-qualifiers/">**

**<rdf:Description rdf:about="_57b620d4-91b2-479b-93e8-70b0eb5e0780">**

**<bqbiol:is>**

**<rdf:Bag>**

**<rdf:li rdf:resource="http://identifiers.org/PUBCHEM.COMPOUND/6255"/></rdf:Bag></bqbiol:is><bqbiol:is>**

**<rdf:Bag>**

**<rdf:li rdf:resource="http://identifiers.org/PUBCHEM.COMPOUND/45109807"/></rdf:Bag></bqbiol:is><bqbiol:is>**

**<rdf:Bag>**

**<rdf:li rdf:resource="http://identifiers.org/PUBCHEM.COMPOUND/10991489"/></rdf:Bag></bqbiol:is><bqbiol:is>**

**<rdf:Bag>**

**<rdf:li rdf:resource="http://identifiers.org/PUBCHEM.COMPOUND/294"/></rdf:Bag></bqbiol:is><bqbiol:is>**

**<rdf:Bag>**

**<rdf:li rdf:resource="http://identifiers.org/PUBCHEM.COMPOUND/439341"/></rdf:Bag></bqbiol:is><bqbiol:is>**

**<rdf:Bag>**

**<rdf:li rdf:resource="http://identifiers.org/PUBCHEM.COMPOUND/439186"/></rdf:Bag></bqbiol:is><bqbiol:is>**

**<rdf:Bag>**

**<rdf:li rdf:resource="http://identifiers.org/PUBCHEM.COMPOUND/303614"/></rdf:Bag></bqbiol:is><bqbiol:is>**

**<rdf:Bag>**

**<rdf:li rdf:resource="http://identifiers.org/PUBCHEM.COMPOUND/181526"/></rdf:Bag></bqbiol:is><bqbiol:is>**

**<rdf:Bag>**

**<rdf:li rdf:resource="http://identifiers.org/INCHIKEY/GUBGYTABKSRVRQ-PICCSMPSSA-N"/></rdf:Bag></bqbiol:is><bqbiol:is>**

**<rdf:Bag>**

**<rdf:li rdf:resource="http://identifiers.org/KEGG.COMPOUND/C01971"/></rdf:Bag></bqbiol:is><bqbiol:is>**

**<rdf:Bag>**

**<rdf:li rdf:resource="http://identifiers.org/KEGG.COMPOUND/C00897"/></rdf:Bag></bqbiol:is><bqbiol:is>**

**<rdf:Bag>**

**<rdf:li rdf:resource="http://identifiers.org/KEGG.COMPOUND/C00208"/></rdf:Bag></bqbiol:is><bqbiol:is>**

**<rdf:Bag>**

**<rdf:li rdf:resource="http://identifiers.org/HMDB/HMDB00163"/></rdf:Bag></bqbiol:is><bqbiol:is>**

**<rdf:Bag>**

**<rdf:li rdf:resource="http://identifiers.org/CHEBI/CHEBI:18167"/></rdf:Bag></bqbiol:is><bqbiol:is>**

**<rdf:Bag>**

**<rdf:li rdf:resource="http://identifiers.org/CHEBI/CHEBI:47937"/></rdf:Bag></bqbiol:is><bqbiol:is>**

**<rdf:Bag>**

**<rdf:li rdf:resource="http://identifiers.org/CHEBI/CHEBI:18147"/></rdf:Bag></bqbiol:is><bqbiol:is>**

**<rdf:Bag>**

**<rdf:li rdf:resource="http://identifiers.org/CHEBI/CHEBI:17306"/></rdf:Bag></bqbiol:is><bqbiol:is>**

**<rdf:Bag>**

**<rdf:li rdf:resource="http://identifiers.org/INCHI/InChI=1S/C12H22O11/c13-1-3-5(15)6(16)9(19)12(22-3)23-10-4(2-14)21-11(20)8(18)7(10)17/h3-20H,1-2H2/t3-,4-,5-,6+,7-,8-,9-,10-,11?,12-/m1/s1"/></rdf:Bag></bqbiol:is><bqbiol:is>**

**<rdf:Bag>**

**<rdf:li rdf:resource="http://identifiers.org/INCHI/InChI=1S/C12H22O11/c13-1-3-5(15)6(16)9(19)12(22-3)23-10-4(2-14)21-11(20)8(18)7(10)17/h3-20H,1-2H2/t3-,4-,5-,6+,7-,8+,9-,10-,11+,12-/m1/s1"/></rdf:Bag></bqbiol:is></rdf:Description></rdf:RDF>**

**</annotation>**

**</species>**

**<species id="M_ksi_pre20" constant="false" hasOnlySubstanceUnits="false" name="keratan sulfate I biosynthesis, precursor 20" metaid="b57e1e14-1fb9-4f9d-9ce2-44204e6fddb5" boundaryCondition="false" compartment="metaComp">**

**<notes>**

**<body xmlns="http://www.w3.org/1999/xhtml">**

**<p>FORMULA: C171H274N12O140S6X</p>**

**<p>CHARGE: 0</p>**

**</body>**

**</notes>**

**<annotation>**

**<rdf:RDF xmlns:rdf="http://www.w3.org/1999/02/22-rdf-syntax-ns#" xmlns:bqmodel="http://biomodels.net/model-qualifiers/" xmlns:bqbiol="http://biomodels.net/biology-qualifiers/">**

**<rdf:Description rdf:about="b57e1e14-1fb9-4f9d-9ce2-44204e6fddb5"/></rdf:RDF>**

**</annotation>**

**</species>**

**<species id="M_ksi_pre21" constant="false" hasOnlySubstanceUnits="false" name="keratan sulfate I biosynthesis, precursor 21" metaid="3d58b0ee-18f8-4d47-87fd-8632b2f86a76" boundaryCondition="false" compartment="metaComp">**

**<notes>**

**<body xmlns="http://www.w3.org/1999/xhtml">**

**<p>FORMULA: C177H284N12O145S6X</p>**

**<p>CHARGE: 0</p>**

**</body>**

**</notes>**

**<annotation>**

**<rdf:RDF xmlns:rdf="http://www.w3.org/1999/02/22-rdf-syntax-ns#" xmlns:bqmodel="http://biomodels.net/model-qualifiers/" xmlns:bqbiol="http://biomodels.net/biology-qualifiers/">**

**<rdf:Description rdf:about="_3d58b0ee-18f8-4d47-87fd-8632b2f86a76"/></rdf:RDF>**

**</annotation>**

**</species>**

**<species id="M_ksi_pre22" constant="false" hasOnlySubstanceUnits="false" name="keratan sulfate I biosynthesis, precursor 22" metaid="08c96a89-1d26-4dc3-8435-78f41258c39f" boundaryCondition="false" compartment="metaComp">**

**<notes>**

**<body xmlns="http://www.w3.org/1999/xhtml">**

**<p>FORMULA: C185H297N13O150S6X</p>**

**<p>CHARGE: 0</p>**

**</body>**

**</notes>**

**<annotation>**

**<rdf:RDF xmlns:rdf="http://www.w3.org/1999/02/22-rdf-syntax-ns#" xmlns:bqmodel="http://biomodels.net/model-qualifiers/" xmlns:bqbiol="http://biomodels.net/biology-qualifiers/">**

**<rdf:Description rdf:about="_08c96a89-1d26-4dc3-8435-78f41258c39f"/></rdf:RDF>**

**</annotation>**

**</species>**

**<species id="M_mi13p" constant="false" hasOnlySubstanceUnits="false" name="1D-myo-Inositol 1,3-bisphosphate" metaid="1bf7936f-d9bc-4a50-95eb-6d0b131c7c77" boundaryCondition="false" compartment="metaComp">**

**<notes>**

**<body xmlns="http://www.w3.org/1999/xhtml">**

**<p>FORMULA: C6H10O12P2</p>**

**<p>CHARGE: 0</p>**

**<p>INCHIKEY: PUVHMWJJTITUGO-FICORBCRSA-N</p>**

**<p>HMDB: HMDB06234</p>**

**<p>KEGG.COMPOUND: C04062</p>**

**<p>CHEBI: CHEBI:18225</p>**

**<p>INCHI: InChI=1S/C6H14O12P2/c7-1-2(8)5(17-19(11,12)13)4(10)6(3(1)9)18-20(14,15)16/h1-10H,(H2,11,12,13)(H2,14,15,16)/t1-,2-,3+,4+,5+,6-</p>**

**</body>**

**</notes>**

**<annotation>**

**<rdf:RDF xmlns:rdf="http://www.w3.org/1999/02/22-rdf-syntax-ns#" xmlns:bqmodel="http://biomodels.net/model-qualifiers/" xmlns:bqbiol="http://biomodels.net/biology-qualifiers/">**

**<rdf:Description rdf:about="_1bf7936f-d9bc-4a50-95eb-6d0b131c7c77">**

**<bqbiol:is>**

**<rdf:Bag>**

**<rdf:li rdf:resource="http://identifiers.org/INCHIKEY/PUVHMWJJTITUGO-FICORBCRSA-N"/></rdf:Bag></bqbiol:is><bqbiol:is>**

**<rdf:Bag>**

**<rdf:li rdf:resource="http://identifiers.org/HMDB/HMDB06234"/></rdf:Bag></bqbiol:is><bqbiol:is>**

**<rdf:Bag>**

**<rdf:li rdf:resource="http://identifiers.org/KEGG.COMPOUND/C04062"/></rdf:Bag></bqbiol:is><bqbiol:is>**

**<rdf:Bag>**

**<rdf:li rdf:resource="http://identifiers.org/CHEBI/CHEBI:18225"/></rdf:Bag></bqbiol:is><bqbiol:is>**

**<rdf:Bag>**

**<rdf:li rdf:resource="http://identifiers.org/INCHI/InChI=1S/C6H14O12P2/c7-1-2(8)5(17-19(11,12)13)4(10)6(3(1)9)18-20(14,15)16/h1-10H,(H2,11,12,13)(H2,14,15,16)/t1-,2-,3+,4+,5+,6-"/></rdf:Bag></bqbiol:is></rdf:Description></rdf:RDF>**

**</annotation>**

**</species>**

**<species id="M_aact" constant="false" hasOnlySubstanceUnits="false" name="ammonioacetone" metaid="06913ea6-b1c3-42a8-869f-40a76147c585" boundaryCondition="false" compartment="metaComp">**

**<notes>**

**<body xmlns="http://www.w3.org/1999/xhtml">**

**<p>FORMULA: C3H8NO</p>**

**<p>CHARGE: 0</p>**

**<p>PUBCHEM.COMPOUND: 215</p>**

**<p>KEGG.COMPOUND: C01888</p>**

**<p>HMDB: HMDB02134</p>**

**<p>CHEBI: CHEBI:58320 || CHEBI:17906</p>**

**<p>INCHI: InChI=1S/C3H7NO/c1-3(5)2-4/h2,4H2,1H3 || InChI=1S/C3H7NO/c1-3(5)2-4/h2,4H2,1H3/p+1</p>**

**</body>**

**</notes>**

**<annotation>**

**<rdf:RDF xmlns:rdf="http://www.w3.org/1999/02/22-rdf-syntax-ns#" xmlns:bqmodel="http://biomodels.net/model-qualifiers/" xmlns:bqbiol="http://biomodels.net/biology-qualifiers/">**

**<rdf:Description rdf:about="_06913ea6-b1c3-42a8-869f-40a76147c585">**

**<bqbiol:is>**

**<rdf:Bag>**

**<rdf:li rdf:resource="http://identifiers.org/PUBCHEM.COMPOUND/215"/></rdf:Bag></bqbiol:is><bqbiol:is>**

**<rdf:Bag>**

**<rdf:li rdf:resource="http://identifiers.org/KEGG.COMPOUND/C01888"/></rdf:Bag></bqbiol:is><bqbiol:is>**

**<rdf:Bag>**

**<rdf:li rdf:resource="http://identifiers.org/HMDB/HMDB02134"/></rdf:Bag></bqbiol:is><bqbiol:is>**

**<rdf:Bag>**

**<rdf:li rdf:resource="http://identifiers.org/CHEBI/CHEBI:58320"/></rdf:Bag></bqbiol:is><bqbiol:is>**

**<rdf:Bag>**

**<rdf:li rdf:resource="http://identifiers.org/CHEBI/CHEBI:17906"/></rdf:Bag></bqbiol:is><bqbiol:is>**

**<rdf:Bag>**

**<rdf:li rdf:resource="http://identifiers.org/INCHI/InChI=1S/C3H7NO/c1-3(5)2-4/h2,4H2,1H3"/></rdf:Bag></bqbiol:is><bqbiol:is>**

**<rdf:Bag>**

**<rdf:li rdf:resource="http://identifiers.org/INCHI/InChI=1S/C3H7NO/c1-3(5)2-4/h2,4H2,1H3/p+1"/></rdf:Bag></bqbiol:is></rdf:Description></rdf:RDF>**

**</annotation>**

**</species>**

**<species id="M_3dsphgn" constant="false" hasOnlySubstanceUnits="false" name="(2S)-1-hydroxy-3-oxooctadecan-2-aminium" metaid="3f9d81c6-3504-4d12-826b-b63ebcf681e9" boundaryCondition="false" compartment="metaComp">**

**<notes>**

**<body xmlns="http://www.w3.org/1999/xhtml">**

**<p>FORMULA: C18H38NO2</p>**

**<p>CHARGE: 0</p>**

**<p>PUBCHEM.COMPOUND: 439853</p>**

**<p>INCHIKEY: KBUNOSOGGAARKZ-KRWDZBQOSA-O</p>**

**<p>KEGG.COMPOUND: C02934</p>**

**<p>HMDB: HMDB01480</p>**

**<p>CHEBI: CHEBI:58299 || CHEBI:17862</p>**

**<p>INCHI: InChI=1S/C18H37NO2/c1-2-3-4-5-6-7-8-9-10-11-12-13-14-15-18(21)17(19)16-20/h17,20H,2-16,19H2,1H3/p+1/t17-/m0/s1 || InChI=1S/C18H37NO2/c1-2-3-4-5-6-7-8-9-10-11-12-13-14-15-18(21)17(19)16-20/h17,20H,2-16,19H2,1H3/t17-/m0/s1</p>**

**</body>**

**</notes>**

**<annotation>**

**<rdf:RDF xmlns:rdf="http://www.w3.org/1999/02/22-rdf-syntax-ns#" xmlns:bqmodel="http://biomodels.net/model-qualifiers/" xmlns:bqbiol="http://biomodels.net/biology-qualifiers/">**

**<rdf:Description rdf:about="_3f9d81c6-3504-4d12-826b-b63ebcf681e9">**

**<bqbiol:is>**

**<rdf:Bag>**

**<rdf:li rdf:resource="http://identifiers.org/PUBCHEM.COMPOUND/439853"/></rdf:Bag></bqbiol:is><bqbiol:is>**

**<rdf:Bag>**

**<rdf:li rdf:resource="http://identifiers.org/INCHIKEY/KBUNOSOGGAARKZ-KRWDZBQOSA-O"/></rdf:Bag></bqbiol:is><bqbiol:is>**

**<rdf:Bag>**

**<rdf:li rdf:resource="http://identifiers.org/KEGG.COMPOUND/C02934"/></rdf:Bag></bqbiol:is><bqbiol:is>**

**<rdf:Bag>**

**<rdf:li rdf:resource="http://identifiers.org/HMDB/HMDB01480"/></rdf:Bag></bqbiol:is><bqbiol:is>**

**<rdf:Bag>**

**<rdf:li rdf:resource="http://identifiers.org/CHEBI/CHEBI:58299"/></rdf:Bag></bqbiol:is><bqbiol:is>**

**<rdf:Bag>**

**<rdf:li rdf:resource="http://identifiers.org/CHEBI/CHEBI:17862"/></rdf:Bag></bqbiol:is><bqbiol:is>**

**<rdf:Bag>**

**<rdf:li rdf:resource="http://identifiers.org/INCHI/InChI=1S/C18H37NO2/c1-2-3-4-5-6-7-8-9-10-11-12-13-14-15-18(21)17(19)16-20/h17,20H,2-16,19H2,1H3/p+1/t17-/m0/s1"/></rdf:Bag></bqbiol:is><bqbiol:is>**

**<rdf:Bag>**

**<rdf:li rdf:resource="http://identifiers.org/INCHI/InChI=1S/C18H37NO2/c1-2-3-4-5-6-7-8-9-10-11-12-13-14-15-18(21)17(19)16-20/h17,20H,2-16,19H2,1H3/t17-/m0/s1"/></rdf:Bag></bqbiol:is></rdf:Description></rdf:RDF>**

**</annotation>**

**</species>**

**<species id="M_CE5308" constant="false" hasOnlySubstanceUnits="false" name="3-oxo-6(R)-hydroxy-tetradec-8-cis-enoate" metaid="5e8d746b-1de2-4313-a0d1-43b79997db86" boundaryCondition="false" compartment="metaComp">**

**<notes>**

**<body xmlns="http://www.w3.org/1999/xhtml">**

**<p>FORMULA: C14H23O4</p>**

**<p>CHARGE: 0</p>**

**</body>**

**</notes>**

**<annotation>**

**<rdf:RDF xmlns:rdf="http://www.w3.org/1999/02/22-rdf-syntax-ns#" xmlns:bqmodel="http://biomodels.net/model-qualifiers/" xmlns:bqbiol="http://biomodels.net/biology-qualifiers/">**

**<rdf:Description rdf:about="_5e8d746b-1de2-4313-a0d1-43b79997db86"/></rdf:RDF>**

**</annotation>**

**</species>**

**<species id="M_CE5309" constant="false" hasOnlySubstanceUnits="false" name="3-oxo-8(R)-hydroxy-hexadeca-6E,10Z-dienoate" metaid="6e24a1d3-7cbe-4d67-a270-2f0bc40acc99" boundaryCondition="false" compartment="metaComp">**

**<notes>**

**<body xmlns="http://www.w3.org/1999/xhtml">**

**<p>FORMULA: C16H25O4</p>**

**<p>CHARGE: 0</p>**

**</body>**

**</notes>**

**<annotation>**

**<rdf:RDF xmlns:rdf="http://www.w3.org/1999/02/22-rdf-syntax-ns#" xmlns:bqmodel="http://biomodels.net/model-qualifiers/" xmlns:bqbiol="http://biomodels.net/biology-qualifiers/">**

**<rdf:Description rdf:about="_6e24a1d3-7cbe-4d67-a270-2f0bc40acc99"/></rdf:RDF>**

**</annotation>**

**</species>**

**<species id="M_CE5306" constant="false" hasOnlySubstanceUnits="false" name="3(S),8(R)-dihydroxy-6E,10Z-hexadecadienoate" metaid="e1533d32-adac-4217-a65d-f37cd5bf0c46" boundaryCondition="false" compartment="metaComp">**

**<notes>**

**<body xmlns="http://www.w3.org/1999/xhtml">**

**<p>FORMULA: C16H27O4</p>**

**<p>CHARGE: 0</p>**

**</body>**

**</notes>**

**<annotation>**

**<rdf:RDF xmlns:rdf="http://www.w3.org/1999/02/22-rdf-syntax-ns#" xmlns:bqmodel="http://biomodels.net/model-qualifiers/" xmlns:bqbiol="http://biomodels.net/biology-qualifiers/">**

**<rdf:Description rdf:about="e1533d32-adac-4217-a65d-f37cd5bf0c46"/></rdf:RDF>**

**</annotation>**

**</species>**

**<species id="M_CE5307" constant="false" hasOnlySubstanceUnits="false" name="3-oxo-10(R)-hydroxy-octadeca-6E,8E,12Z-trienoate" metaid="8ba2c272-8c20-4c7e-9733-4783bbd22dc7" boundaryCondition="false" compartment="metaComp">**

**<notes>**

**<body xmlns="http://www.w3.org/1999/xhtml">**

**<p>FORMULA: C18H27O4</p>**

**<p>CHARGE: 0</p>**

**</body>**

**</notes>**

**<annotation>**

**<rdf:RDF xmlns:rdf="http://www.w3.org/1999/02/22-rdf-syntax-ns#" xmlns:bqmodel="http://biomodels.net/model-qualifiers/" xmlns:bqbiol="http://biomodels.net/biology-qualifiers/">**

**<rdf:Description rdf:about="_8ba2c272-8c20-4c7e-9733-4783bbd22dc7"/></rdf:RDF>**

**</annotation>**

**</species>**

**<species id="M_CE5304" constant="false" hasOnlySubstanceUnits="false" name="15-deoxy-PGD2" metaid="046d37fb-cbef-400e-bf4d-c09b8a67817f" boundaryCondition="false" compartment="metaComp">**

**<notes>**

**<body xmlns="http://www.w3.org/1999/xhtml">**

**<p>FORMULA: C20H29O4</p>**

**<p>CHARGE: 0</p>**

**<p>PUBCHEM.COMPOUND: 5283052</p>**

**<p>INCHI: InChI=1/C20H30O4/c1-2-3-4-5-6-9-12-16-17(19(22)15-18(16)21)13-10-7-8-11-14-20(23)24/h6-7,9-10,12,17,19,22H,2-5,8,11,13-15H2,1H3,(H,23,24)/p-1/b9-6-,10-7-,16-12+/t17-,19+/m0/s1</p>**

**</body>**

**</notes>**

**<annotation>**

**<rdf:RDF xmlns:rdf="http://www.w3.org/1999/02/22-rdf-syntax-ns#" xmlns:bqmodel="http://biomodels.net/model-qualifiers/" xmlns:bqbiol="http://biomodels.net/biology-qualifiers/">**

**<rdf:Description rdf:about="_046d37fb-cbef-400e-bf4d-c09b8a67817f">**

**<bqbiol:is>**

**<rdf:Bag>**

**<rdf:li rdf:resource="http://identifiers.org/PUBCHEM.COMPOUND/5283052"/></rdf:Bag></bqbiol:is><bqbiol:is>**

**<rdf:Bag>**

**<rdf:li rdf:resource="http://identifiers.org/INCHI/InChI=1/C20H30O4/c1-2-3-4-5-6-9-12-16-17(19(22)15-18(16)21)13-10-7-8-11-14-20(23)24/h6-7,9-10,12,17,19,22H,2-5,8,11,13-15H2,1H3,(H,23,24)/p-1/b9-6-,10-7-,16-12+/t17-,19+/m0/s1"/></rdf:Bag></bqbiol:is></rdf:Description></rdf:RDF>**

**</annotation>**

**</species>**

**<species id="M_CE5305" constant="false" hasOnlySubstanceUnits="false" name="3(S),6(R)-dihydroxy-tetradec-8Z-enoate" metaid="c7a4950e-4d84-4653-a649-268cd4d7baad" boundaryCondition="false" compartment="metaComp">**

**<notes>**

**<body xmlns="http://www.w3.org/1999/xhtml">**

**<p>FORMULA: C14H25O4</p>**

**<p>CHARGE: 0</p>**

**</body>**

**</notes>**

**<annotation>**

**<rdf:RDF xmlns:rdf="http://www.w3.org/1999/02/22-rdf-syntax-ns#" xmlns:bqmodel="http://biomodels.net/model-qualifiers/" xmlns:bqbiol="http://biomodels.net/biology-qualifiers/">**

**<rdf:Description rdf:about="c7a4950e-4d84-4653-a649-268cd4d7baad"/></rdf:RDF>**

**</annotation>**

**</species>**

**<species id="M_odecoa" constant="false" hasOnlySubstanceUnits="false" name="Octadecenoyl-CoA (n-C18:1CoA)" metaid="d3f5683e-b258-4551-b9ec-8ea9620c1ea6" boundaryCondition="false" compartment="metaComp">**

**<notes>**

**<body xmlns="http://www.w3.org/1999/xhtml">**

**<p>FORMULA: C39H64N7O17P3S</p>**

**<p>CHARGE: 0</p>**

**<p>PUBCHEM.COMPOUND: 92160 || 5280355 || 3793</p>**

**<p>KEGG.COMPOUND: C00510</p>**

**<p>HMDB: HMDB01322</p>**

**<p>CHEBI: CHEBI:15534 || CHEBI:57387</p>**

**<p>INCHI: InChI=1S/C39H68N7O17P3S/c1-4-5-6-7-8-9-10-11-12-13-14-15-16-17-18-19-30(48)67-23-22-41-29(47)20-21-42-37(51)34(50)39(2,3)25-60-66(57,58)63-65(55,56)59-24-28-33(62-64(52,53)54)32(49)38(61-28)46-27-45-31-35(40)43-26-44-36(31)46/h11-12,26-28,32-34,38,49-50H,4-10,13-25H2,1-3H3,(H,41,47)(H,42,51)(H,55,56)(H,57,58)(H2,40,43,44)(H2,52,53,54)/p-4/b12-11-/t28-,32-,33-,34+,38-/m1/s1 || InChI=1S/C39H68N7O17P3S/c1-4-5-6-7-8-9-10-11-12-13-14-15-16-17-18-19-30(48)67-23-22-41-29(47)20-21-42-37(51)34(50)39(2,3)25-60-66(57,58)63-65(55,56)59-24-28-33(62-64(52,53)54)32(49)38(61-28)46-27-45-31-35(40)43-26-44-36(31)46/h11-12,26-28,32-34,38,49-50H,4-10,13-25H2,1-3H3,(H,41,47)(H,42,51)(H,55,56)(H,57,58)(H2,40,43,44)(H2,52,53,54)/b12-11-/t28-,32-,33-,34+,38-/m1/s1 || InChI=1S/C39H68N7O17P3S/c1-4-5-6-7-8-9-10-11-12-13-14-15-16-17-18-19-30(48)67-23-22-41-29(47)20-21-42-37(51)34(50)39(2,3)25-60-66(57,58)63-65(55,56)59-24-28-33(62-64(52,53)54)32(49)38(61-28)46-27-45-31-35(40)43-26-44-36(31)46/h11-12,26-28,32-34,38,49-50H,4-10,13-25H2,1-3H3,(H,41,47)(H,42,51)(H,55,56)(H,57,58)(H2,40,43,44)(H2,52,53,54)/b12-11-/t28-,32-,33-,34?,38-/m1/s1</p>**

**</body>**

**</notes>**

**<annotation>**

**<rdf:RDF xmlns:rdf="http://www.w3.org/1999/02/22-rdf-syntax-ns#" xmlns:bqmodel="http://biomodels.net/model-qualifiers/" xmlns:bqbiol="http://biomodels.net/biology-qualifiers/">**

**<rdf:Description rdf:about="d3f5683e-b258-4551-b9ec-8ea9620c1ea6">**

**<bqbiol:is>**

**<rdf:Bag>**

**<rdf:li rdf:resource="http://identifiers.org/PUBCHEM.COMPOUND/92160"/></rdf:Bag></bqbiol:is><bqbiol:is>**

**<rdf:Bag>**

**<rdf:li rdf:resource="http://identifiers.org/PUBCHEM.COMPOUND/5280355"/></rdf:Bag></bqbiol:is><bqbiol:is>**

**<rdf:Bag>**

**<rdf:li rdf:resource="http://identifiers.org/PUBCHEM.COMPOUND/3793"/></rdf:Bag></bqbiol:is><bqbiol:is>**

**<rdf:Bag>**

**<rdf:li rdf:resource="http://identifiers.org/KEGG.COMPOUND/C00510"/></rdf:Bag></bqbiol:is><bqbiol:is>**

**<rdf:Bag>**

**<rdf:li rdf:resource="http://identifiers.org/HMDB/HMDB01322"/></rdf:Bag></bqbiol:is><bqbiol:is>**

**<rdf:Bag>**

**<rdf:li rdf:resource="http://identifiers.org/CHEBI/CHEBI:15534"/></rdf:Bag></bqbiol:is><bqbiol:is>**

**<rdf:Bag>**

**<rdf:li rdf:resource="http://identifiers.org/CHEBI/CHEBI:57387"/></rdf:Bag></bqbiol:is><bqbiol:is>**

**<rdf:Bag>**

**<rdf:li rdf:resource="http://identifiers.org/INCHI/InChI=1S/C39H68N7O17P3S/c1-4-5-6-7-8-9-10-11-12-13-14-15-16-17-18-19-30(48)67-23-22-41-29(47)20-21-42-37(51)34(50)39(2,3)25-60-66(57,58)63-65(55,56)59-24-28-33(62-64(52,53)54)32(49)38(61-28)46-27-45-31-35(40)43-26-44-36(31)46/h11-12,26-28,32-34,38,49-50H,4-10,13-25H2,1-3H3,(H,41,47)(H,42,51)(H,55,56)(H,57,58)(H2,40,43,44)(H2,52,53,54)/p-4/b12-11-/t28-,32-,33-,34+,38-/m1/s1"/></rdf:Bag></bqbiol:is><bqbiol:is>**

**<rdf:Bag>**

**<rdf:li rdf:resource="http://identifiers.org/INCHI/InChI=1S/C39H68N7O17P3S/c1-4-5-6-7-8-9-10-11-12-13-14-15-16-17-18-19-30(48)67-23-22-41-29(47)20-21-42-37(51)34(50)39(2,3)25-60-66(57,58)63-65(55,56)59-24-28-33(62-64(52,53)54)32(49)38(61-28)46-27-45-31-35(40)43-26-44-36(31)46/h11-12,26-28,32-34,38,49-50H,4-10,13-25H2,1-3H3,(H,41,47)(H,42,51)(H,55,56)(H,57,58)(H2,40,43,44)(H2,52,53,54)/b12-11-/t28-,32-,33-,34+,38-/m1/s1"/></rdf:Bag></bqbiol:is><bqbiol:is>**

**<rdf:Bag>**

**<rdf:li rdf:resource="http://identifiers.org/INCHI/InChI=1S/C39H68N7O17P3S/c1-4-5-6-7-8-9-10-11-12-13-14-15-16-17-18-19-30(48)67-23-22-41-29(47)20-21-42-37(51)34(50)39(2,3)25-60-66(57,58)63-65(55,56)59-24-28-33(62-64(52,53)54)32(49)38(61-28)46-27-45-31-35(40)43-26-44-36(31)46/h11-12,26-28,32-34,38,49-50H,4-10,13-25H2,1-3H3,(H,41,47)(H,42,51)(H,55,56)(H,57,58)(H2,40,43,44)(H2,52,53,54)/b12-11-/t28-,32-,33-,34?,38-/m1/s1"/></rdf:Bag></bqbiol:is></rdf:Description></rdf:RDF>**

**</annotation>**

**</species>**

**<species id="M_lyxnt" constant="false" hasOnlySubstanceUnits="false" name="L-lyxonate" metaid="263e340b-a539-4aee-86d2-f94a77420f10" boundaryCondition="false" compartment="metaComp">**

**<notes>**

**<body xmlns="http://www.w3.org/1999/xhtml">**

**<p>FORMULA: C5H9O6</p>**

**<p>CHARGE: 0</p>**

**<p>PUBCHEM.COMPOUND: 7778 || 644110</p>**

**<p>INCHIKEY: QXKAIJAYHKCRRA-PZGQECOJSA-N</p>**

**<p>KEGG.COMPOUND: C05412</p>**

**<p>CHEBI: CHEBI:6268</p>**
[truncated: 8,656,360 more chars]
